# Increased nutrient diversity can induce loss of microbial diversity through enhanced resource uptake

**DOI:** 10.1038/s41559-026-03111-4

**Published:** 2026-07-20

**Authors:** Or Shalev, Xiaozhou Ye, Joachim Kilian, Mark Stahl, Christoph Ratzke

**Affiliations:** 1 Interfaculty Institute for Microbiology and Infection Medicine Tübingen (IMIT), Cluster of Excellence EXC 2124 “Controlling Microbes to Fight Infections” (CMFI), University of Tübingen, Tübingen, Germany; 2 Zentrum für Molekularbiologie der Pflanzen (ZMBP), University of Tübingen, Tübingen, Germany

**Keywords:** Microbial ecology, Emergence

## Abstract

The origin of biodiversity is a central question in ecology, particularly how numerous microbial species co-exist within a single community. A prevailing hypothesis holds that microbial species co-exist by specializing on different resources, thereby reducing competition. Accordingly, increasing resource diversity is expected to promote species co-existence and boost biodiversity. By integrating high-throughput experiments, ecological analysis of global microbiome data, metabolic profiling and theoretical modelling, we find that the relationship between resource diversity and microbial biodiversity is not consistently positive and can even decline with increasing resource diversity. This unexpected result emerges from a widespread physiological response across diverse microbial taxa, in which more complex environments trigger higher overall resource uptake. This intensifies competition and can lead to biodiversity loss. Updating a central ecological model to include this physiological trait accurately reproduces the observed decline. Our findings suggest that physiological changes at the individual level can substantially alter predicted diversity patterns and scale up to influence community structure.

## Abstract (web summary)

Increasing nutrient diversity is expected to promote microbial diversity. This study shows that more complex environments can enhance resource uptake, intensify competition and lead to microbial biodiversity loss, challenging a central ecological assumption.

## Main

**Fig. 1:**
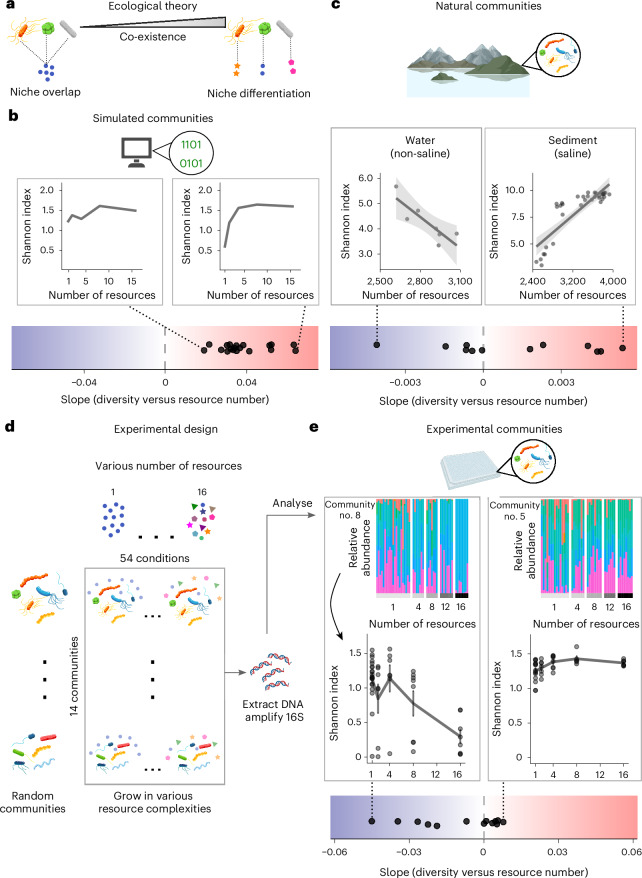
Microbial biodiversity often declines with increasing resource diversity across natural and experimental communities. **a**, When microbial species prefer different resources, higher resource diversity is expected to promote co-existence by reducing competition. This schematic is simplified for illustrative purposes; in reality, microbial communities also exhibit cross-feeding interactions and varying niche breadths, which are not depicted here. **b**, This expectation is supported by simulations using a consumer–resource model (Supplementary Text 1, Section 1; 20 simulated communities). Top: example communities showing the weakest and strongest biodiversity responses to increasing resource number. Bottom: for each community, biodiversity response was quantified as the slope (*β*_1_) of a linear model (Shannon index = *β*_0_ + *β*_1_ × number of resources + *ε*), fitted using the two extreme conditions: single-resource (1) and maximal-resource (16) environments. The mean slope across all simulated communities is shown in Supplementary Fig. 1a (Shannon index) and Supplementary Fig. 1b (richness). **c**, In natural microbial communities, biodiversity does not consistently increase with resource diversity. Data from the EMP^19^ (462 samples across 13 ecosystems) combine 16S-based diversity estimates with mass spectrometry-derived resource profiles (Methods). Top: example ecosystems showing positive and negative relationships between resource diversity and microbial biodiversity (full results in Supplementary Fig. 1c). A similar pattern is observed using richness instead of Shannon diversity (Supplementary Fig. 1d). Notably, the animal distal gut (non-saline) environment contains the most samples in the EMP dataset and shows a significant negative correlation (Supplementary Fig. 1d). Bottom: extending this analysis to all 13 ecosystems, we derive the slope (*β*_1_) of Shannon diversity index versus resource number as described in **b**. **d**, To experimentally test the effect of resource diversity on microbial diversity, we used 14 bacterial strains (Supplementary Data 2) to assemble 14 random synthetic communities—7 communities with 6 members and 7 communities with 8 members. Each community was cultured across five levels of resource diversity (1, 2, 4, 8 or 16 carbon sources), totalling 54 conditions per community. **e**, In our controlled experiments, microbial biodiversity generally decreased with increasing resource diversity, whereas the magnitude of this effect varied across communities. Top: communities with the strongest biodiversity decrease and increase in response to resource diversity are shown, along with relative abundances of their member strains. Shading indicates mean ± s.e.m. For the single-resource condition *n* = 21 and *n* = 8 for mixed-resource conditions (>1 resource). Results using richness are shown in Supplementary Fig. 1f. Bottom: extending this analysis to all 14 communities, we derive the slope (*β*_1_) of Shannon diversity index versus resource number as described in **b**. Illustrations in **b, c** and **e** partially created in BioRender; Ratzke, C. https://biorender.com/zrfhfyl (2026). Source data

Microbial life flourishes in astonishing biodiversity. A single gram of soil can harbour over ten thousand bacterial species^1^. Yet how so many species co-exist has remained a central question in ecology for nearly a century^2–4^. Classic theory holds that when species compete for the same resource, the most efficient consumer prevails, driving others to extinction through competitive exclusion^2^. Co-existence, therefore, requires niche differentiation: species avoid direct competition by exploiting different resources^5^. By this logic, greater resource diversity should support more co-existing species^6,7^ (Fig. 1a; hereafter, resource ‘diversity’ is used interchangeably with resource ‘number’).

This principle is formalized in the consumer–resource model^8^. In this model, species compete for limited resources by consuming them and converting them into biomass, which reduces the amount of resources available to others. When species are assigned random metabolic traits and uptake efficiencies, using a realistic framework that also allows metabolic byproduct secretion (cross-feeding, which is a common mechanism in microbial communities^9^), the model predicts that increasing resource diversity allows more species to persist—as naively expected and previously shown by others^10^ (Fig. 1b and Supplementary Fig. 1a,b). This relationship saturates at high-resource numbers because increasing niche overlap limits how many species can stably co-exist. The positive relationship between resource diversity and biodiversity may be further reinforced by diauxic shifts, which can promote species co-existence in mixed-resource environments^11^. Although additional mechanisms such as stochastic processes^12^, viral infections^13^, metabolic interdependencies^14^ and environmental fluctuations^15,16^ have also been proposed to sustain microbial biodiversity, the concept that resource diversity drives species biodiversity remains foundational. Yet despite decades of theoretical support, empirical evidence for this link remains scarce^17,18^, raising the question of whether this long-standing theory truly holds in microbial ecosystems.

As a first step towards exploring this question, we re-analysed data from the Earth Microbiome Project (EMP)^19^ that contained both the species diversity (measured by DNA sequencing) and nutrient diversity (measured by mass spectrometry) for the same samples, allowing us to look at the connection between both. We found that resource diversity does not consistently correlate with biodiversity (Fig. 1c and Supplementary Fig. 1c,d): in some ecosystems biodiversity increases with higher resource diversity, whereas in others, it decreases. But, interpreting these data is difficult, as it is not clear whether the detected nutrients were supplied by the environment or synthesized by the bacteria, complicating efforts to link species composition to nutrient richness. More broadly, natural systems are inherently uncontrolled and confounded by several unmeasured factors, which makes data interpretation challenging and calls for controlled laboratory experiments. Nonetheless, only a few controlled experiments have directly addressed this question^17,18^. Others^17^ found a positive relationship between resource and microbial diversity in microcosms, although the increase in biodiversity was weaker than expected. However, these experiments involved a single, complex and undefined soil-derived community containing thousands of taxa. When transferred to laboratory conditions, such diverse natural communities often collapse to a few dominant species, as observed in this study, because most natural bacteria cannot survive under laboratory conditions. Such strong environmental filtering can make it difficult to disentangle how species interactions change with increasing resource diversity. By contrast, Pacheco et al.^18^ examined how increasing resource diversity influences diversity in a defined microbial community. Here the biodiversity increase was substantially weaker than predicted by classical consumer–resource models and even smaller than that observed by Dal Bello et al.^17^, a result that was central to their analysis. Similarly to Dal Bello et al.^17^, the use of a single community constrains the generality of their conclusions.

Motivated by the difficulty of drawing causal conclusions from the EMP patterns and by the limitations of previous laboratory studies, we systematically tested several defined microbial communities and thousands of interactions, and found that—contrary to expectations—microbial biodiversity can decline as resource diversity increases.

## Results

### Greater resource diversity often reduces microbial biodiversity

To test the fundamental relationship between resource diversity and microbial biodiversity, we assembled random microbial communities across a range of resource diversities (Fig. 1d; Methods). Communities were cultivated for 9 days under a daily dilution regime to allow them to stabilize (Supplementary Fig. 2), and final community composition was assessed using 16S ribosomal RNA amplicon sequencing. This yielded 701 sequenced samples across different experimental conditions (Supplementary Data 1).

Contrary to predictions from the consumer–resource model (Fig. 1b), microbial biodiversity declined in a notable fraction of communities as resource diversity increased (7 of 14 communities showed declines in both Shannon diversity and richness), while increases in biodiversity were rare and typically modest (Fig. 1e and Supplementary Figs. 1e,f, 3 and 4). The patterns remained when controlling for potential contamination and the exclusion of low-abundance taxa (Supplementary Fig. 3). These findings challenge the ecological expectation that higher resource diversity promotes co-existence and raise the question of what drives this widespread biodiversity loss.

### Resource-diversity fuels competition, which reduces co-existence

**Fig. 2:**
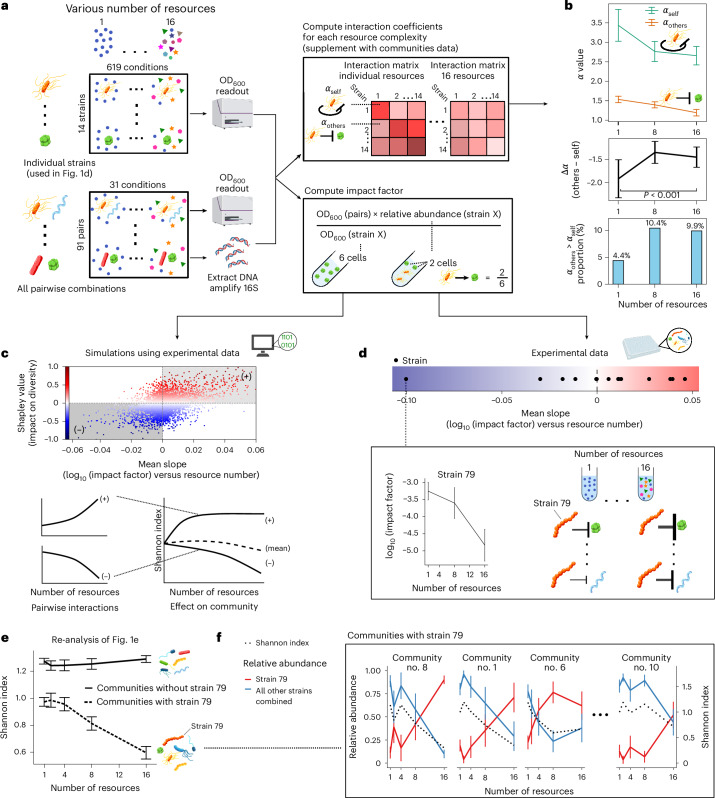
Increasing pairwise competitiveness reduces community biodiversity as resource diversity increases. **a**, Overview of the experimental and modelling workflow. We studied pairwise interactions among 14 bacterial strains across 1, 8 and 16 carbon-source conditions. Each pair was tested in an average of 31 conditions and individual strains were profiled in all their respective conditions, totalling 619 measurements (Supplementary Data 3). OD_600_ and 16S rRNA sequencing were used to quantify growth and composition. Interaction matrices were inferred by fitting a generalized Lotka–Volterra model with Bayesian regression to our data (Methods), using observed data to estimate intraspecies (*α*_self_) and interspecies (*α*_others_) interaction strengths (Supplementary Text 2; results in **b**). Interaction matrices were inferred by a Bayesian regression of a generalized Lotka–Volterra model and the mean interaction coefficients for 1, 8 and 16 carbon sources were calculated (Supplementary Fig. 6 and Supplementary Text 2), allowing comparison of *α*_self_ and *α*_others_ interactions across resource levels (**b**). To assess strain-level contributions to diversity loss, we defined an impact factor on the basis of the change in the absolute abundance (estimated from OD_600_ and 16S data) of the partner of a strain in co-culture versus monoculture. For example, a drop from 6 to 2 yields an impact factor of 0.33. **b**, Resource complexity shifted communities towards competitive exclusion. On average, *α*_self_ decreased more than *α*_others_, reducing co-existence potential (top and middle). The proportion of interactions where *α*_others_ > *α*_self_ increased with resource number (bottom). Trend lines show mean ± s.e.m. **c**, Simulations using noise-perturbed experimental data showed that strains with more negative impact factor slopes (1 versus 16 resources) contributed more to biodiversity loss. The cartoon illustrates how increased pairwise suppression by a single strain can scale up to reduce overall community diversity. **d**, Strain 79 showed the steepest increase in suppressiveness among experimentally tested strains. For each strain, impact factors were averaged across all pairwise interaction partners for each resource condition and we fitted log_10_(impact factor) = *β*_0_ + *β*_1_ × number of resources + *ε* across 1-, 8- and 16-resource conditions (3,398 values total). Strain 79 showed the most negative *β*_1_. The cartoon illustrates how its suppressive effect increased consistently across interaction partners with resource diversity. **e**, Communities containing strain 79 (*n* = 8) showed a sharp biodiversity decline with increasing resource diversity, whereas those without it (*n* = 6) slightly increased. Trend lines show the mean ± s.e.m. **f**, In these same communities, strain 79 increased in relative abundance whereas other strains declined, along with a drop in Shannon diversity. Trend lines show the mean ± s.e.m. Shown are four representative examples (full data in Supplementary Fig. 14). Illustrations in **a**, **c** and **d** partially created in BioRender; Ratzke, C. https://biorender.com/zrfhfyl (2026). Source data

Microbial communities are strongly shaped by interactions between their constituent strains. We hypothesized that the observed loss of biodiversity might result from a fundamental shift in these interactions triggered by increasing resource diversity. To test this, we systematically co-incubated all pairwise combinations of the 14 strains used in our synthetic communities under a wide range of resource conditions, grouped into three levels of resource diversity: 1, 8 and 16 resources. For each pairwise co-culture, we measured total biomass (optical density OD_600_) and relative abundances by 16S amplicon sequencing after incubating the interaction pairs for 5 days under daily dilution. The total abundance of each species in the final community was then estimated by multiplying the total biomass by its relative abundance. We also measured how each strain grew in isolation in each environment (Supplementary Fig. 5). These steady-state abundances are determined by microbial interactions and therefore allow us to get insights into those. A simple way to connect interactions within a community with its steady-state composition is offered by generalized Lotka–Volterra models:

1 $$\displaystyle\frac{{\rm{d}}{x}_{i}}{{\rm{d}}t}={{k}_{i}x}_{i}\left(1-\sum _{j}{\alpha }_{{ij}}{x}_{j}\right)$$

where *x*_i_ and *k*_*i*_ are the population density and per capita growth rate of species *i* and *α*_*ij*_ is the impact of species *j* on species *i* by interaction. By fitting the steady-state solution of these equations to our individual and pairwise data, along with community data (Fig. 1e), we obtained estimates of the interactions (*α*_*ij*_) between the microbes for different nutrient diversities. Since we fit the same equations with data obtained from many nutrient conditions, we would expect a certain variability of the underlying *α*_*ij*_. Therefore, we used a Bayesian regression that naturally allows for its parameters to be distributed (Methods; Supplementary Text 2), in line with similar previous approaches^20,21^. This yielded interaction matrices for the three levels of resource diversity (illustrated in Fig. 2a; Supplementary Fig. 6), which, when used to simulate community assembly, reproduced the observed decline in biodiversity (Supplementary Fig. 7), suggesting the validity of the inferred interactions. In these matrices, diagonal terms represent self-inhibition (how much a strain limits its own growth; *α*_self_) and off-diagonal terms capture how strongly a strain inhibits others (*α*_others_). The *α* values represent per capita interaction strengths, meaning they are independent of population densities. According to theory, when interspecies inhibition exceeds self-inhibition (*α*_others_ > *α*_self_), competitive exclusion will occur, where one strain will outcompete the other^22^. When a strain inhibits itself stronger than its interaction partner (*α*_others_ < *α*_self_) co-existence is possible. Therefore, if biodiversity loss is driven by pairwise interactions, we would expect increasing resource diversity to shift this balance in favour of exclusion. Interestingly, both self-inhibition and interspecies inhibition became weaker as resource diversity increased (Fig. 2b (top) and Supplementary Figs. 8 and 7). However, self-inhibition (*α*_self_) decreased more rapidly than interspecies inhibition (*α*_others_; Fig. 2b (middle)), leading to a greater number of predicted competitive exclusions as resource complexity (that is, resource diversity) increased (Fig. 2b (bottom) and Supplementary Figs. 8c and 9). Counterintuitively, it is therefore not stronger per capita inhibition between competitors, but greater individual growth—driven by reduced self-inhibition—that drives competitive exclusion. Increased growth leads to larger population sizes, which exert stronger total inhibitory effects on competitors, even as per capita interspecies inhibition becomes weaker with increasing resource diversity.

The above findings explain the average biodiversity loss with increasing resource diversity, but the strength of this effect varied across communities (Fig. 1e). This suggests that community composition influenced the outcome, with some strains playing a larger role than others. To identify which strains drive biodiversity loss, we defined an ‘impact factor’—which equals the growth of a strain in the presence of an interaction partner divided by the growth without it. This metric quantifies how the population of one species affects that of another and thus characterizes the influence of a given species, and is therefore defined for the strain exerting the effect. An impact factor of 1 indicates neutrality, values below 1 indicate suppression and values above 1 indicate facilitation. In the context of a generalized Lotka–Volterra framework these impact factors depend on all the four alphas of a pairwise interaction (two *α*_self_ and two *α*_other_) that we estimated above (Fig. 2a; Methods). We hypothesized that strains whose impact factors tend to decrease—that is, became more suppressive—as resource diversity increased would be key drivers of biodiversity loss. To test this, we extensively simulated communities using synthetic strain sets generated by adding random noise to the experimentally derived interaction matrices for 1, 8 and 16 carbon sources with generalized Lotka–Volterra equations (Methods). This approach allowed us to generalize beyond the limited statistical power of the experimental dataset. For each simulated community, we calculated biodiversity under low- and high-resource diversity, allowing us to estimate the change of biodiversity with resource number. By calculating Shapley values for each strain within the community, we could assess the contribution of each strain to this change in biodiversity and plot it against strain-specific changes in impact factor (Fig. 2c and Supplementary Fig. 11). Indeed, strains that became more suppressive with increasing resource diversity consistently drove greater biodiversity loss, validating our hypothesis.

To confirm these predictions experimentally, we examined our experimental dataset and found that strains becoming more suppressive with increasing resource diversity contributed more strongly to biodiversity loss (Supplementary Fig. 12). One strain in particular, ‘79’, stood out—showing a pronounced decrease in impact factor (that is, increased suppression) as resource diversity rose (Fig. 2d and Supplementary Fig. 13). Comparing communities with and without strain 79 revealed a striking pattern: those containing it exhibited sharp biodiversity declines with increasing resource diversity, whereas those without it showed slight biodiversity gains (Fig. 2e). In communities with strain 79, its relative abundance increased steadily with resource diversity, eventually dominating at 16 resources as the total abundance of other strains declined (Fig. 2f and Supplementary Fig. 14). Shannon diversity mirrored this trend, decreasing in parallel with the collapse of the remaining community members.

We tested whether the mechanism proposed by Pacheco et al.^18^ could map onto our results. Their work suggested that higher resource generalism leads to stronger competitive advantage and dominance at elevated resource diversity, reducing co-existence through increased niche overlap. Strain 79, despite its strong biodiversity impact, exhibited only moderate niche breadth compared with the other community members, indicating that its dominance cannot be explained by generalism alone (Supplementary Fig. 15).

Taken together, our findings show that increasing resource diversity can amplify the suppressive potential of specific strains, which in turn drives biodiversity loss. This raises two key questions: how common is it for strains to become more competitive as resource diversity increases and what physiological mechanisms underlie this shift?

### Increased microbial competitiveness with resource diversity is widespread across our strain collection

**Fig. 3:**
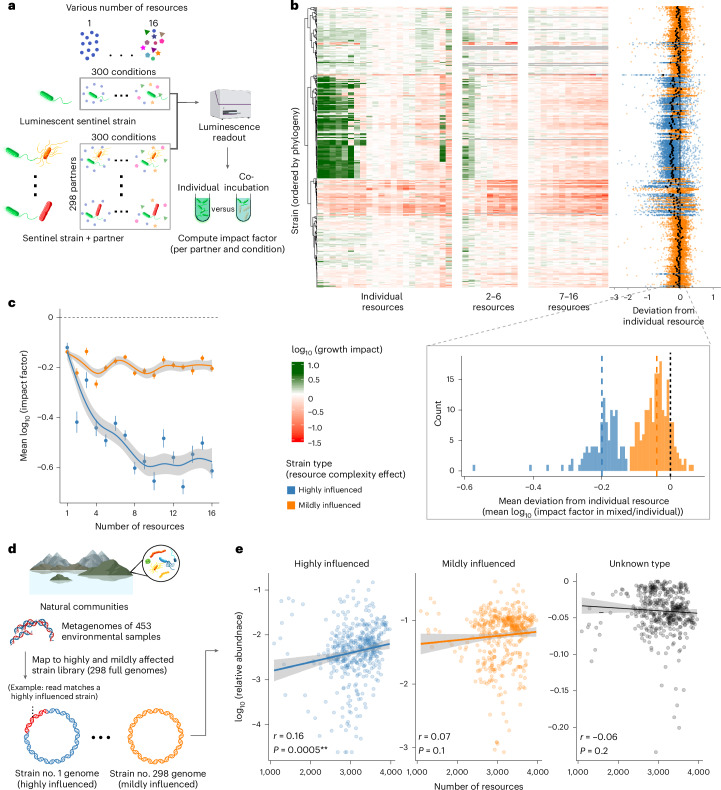
Resource complexity consistently increases microbial competitiveness, particularly among a subset of strains. **a**, We performed a high-throughput experiment using a luminescent sentinel strain (*P. aeruginosa* PA01) to quantify microbial interactions. Its growth was measured alone and in co-culture with each of 298 partner strains across 16 levels of resource diversity (1–16 resources), including 22 individual resources and 278 random mixtures (Supplementary Data 4). This yielded 32,216 impact factor measurements quantifying the effect of each partner across conditions. **b**, *K*-means clustering (Supplementary Fig. 17) classified strains into two types on the basis of their impact factor response: highly influenced (109 strains) and mildly influenced (189 strains). Both types became more suppressive on average with increased carbon complexity, but highly influenced strains showed a much stronger shift. Top: heatmap shows log_10_(impact factor) across individual resources and selected mixtures (grey, missing data). Strains are ordered by core-genome phylogeny inferred with panX (95% core-gene threshold; Methods). Right: the deviation of each strain in resource mixtures (≥2 resources) from its mean impact across individual resources. Black points represent the average deviation of each strain. In total, 6,556 impact factor values were calculated for individual resources (all shown in the heatmap) and 26,660 for resource mixtures (heatmap and plot on the right). Bottom: histogram shows the distribution of mean log_10_(impact factor) differences between individual resources and mixed resources (2–16 resources) for highly and mildly influenced strains. The bimodal pattern reflects the distinct groupings, with dashed lines indicating the mean of each group. **c**, Highly influenced strains show a sharp decline in impact factor, indicating greater suppression, with increasing resource complexity; mildly influenced strains shift only slightly. Lines show mean ± s.e.m., with smoothed generalized additive model fit and 95% confidence intervals. Value *n* denotes the number of independent strain–resource condition observations per group (Supplementary Table 1 gives exact sample sizes). **d**, Metagenomic reads from 462 EMP samples across 18 ecosystems were mapped to the full genomes of the 298 tested strains, grouped by their response to resource complexity (highly or mildly influenced; **b** and Methods). Read counts per group were calculated and converted to relative abundances. Illustration, a metagenomic read mapping to a highly influenced strain. **e**, The relative abundance of highly influenced strains increases with resource complexity across all samples, in contrast to mildly influenced or unmapped reads. Pearson correlation (*r*) and *P* values are shown. Raw (unadjusted) *P* values are shown in each panel. Statistical significance was assessed after Benjamini–Hochberg correction for several comparisons and asterisks denote adjusted *P* values (**P < 0.01). Illustrations in **a** and **d** partially created in BioRender; Ratzke, C. https://biorender.com/zrfhfyl (2026). Source data

To assess how often strains become more competitive with increasing resource diversity, we measured our established impact factor metric (illustrated in Fig. 2a) for 298 strains against a sentinel strain across a wide range of resource conditions, generating ~32,000 data points (Fig. 3a; Methods). As resource complexity increased, most strains became more suppressive (Fig. 3b (top) and Supplementary Fig. 16). However, within this trend, we identified two distinct response types: mildly influenced strains (189) and highly influenced strains (109), the latter showing a much sharper gain in suppression under complex conditions (Fig. 3b (bottom) and Supplementary Figs. 17 and 18). Both types were phylogenetically conserved (Fig. 3b (top); Mantel test, *r* = 0.145, *P* = 0.0001, 9,999 permutations; for taxonomic information see Supplementary Fig. 19 and Supplementary Data 5), suggesting that evolutionary constraints shape competitive strategies. Consistent with this, strain 79 (Fig. 2e,f) belongs to the genus *Pseudomonas* (based on 16S rRNA classification), which is well represented among suppressive highly influenced strains. Notably, these response types were already distinguishable on the basis of how strains behaved in individual-resource environments (Supplementary Figs. 20 and 21), with highly influenced strains forming two groups: facultative strains, whose effects depended on the resource type (typically facilitative in sugars but suppressive in acids) and consistently suppressive strains. By contrast, mildly influenced strains constituted a third, largely neutral group that exerted weaker effects on the sentinel strain. The responses of highly and mildly influenced strains were strongly quantitative, scaling with the number of available resources: highly influenced strains started out less suppressive than mildly influenced strains under simple conditions, but became more suppressive as resource complexity increased (Fig. 3c). Strikingly, these strains also lost the facilitative effects that were common in individual carbon sources (Fig. 3b (top) and Supplementary Fig. 18). Thus, overall, we observed an increase of competitiveness with increasing nutrient diversity across many species.

Because a previous study^18^ linked resource generalism to higher competitiveness under increasing resource diversity, we next examined whether generalism could underlie the enhanced competitiveness of highly influenced strains. We defined niche breadth as the proportion of tested carbon resources on which a strain showed measurable growth (OD_600_ > 0.15 after 72 h). Generalist strains were moderately enriched among highly influenced types (*r* = 0.36, *P* < 0.001; *κ* = 0.36; Jaccard = 0.43; Supplementary Fig. 22), suggesting that broad niche breadth may facilitate competitive dominance at higher resource numbers. However, the overlap between generalist strains and highly influenced strains is only partial. For example, only 67 strains are both generalists and highly influenced, whereas 89 strains fall exclusively into one category or the other (Supplementary Fig. 22). This incomplete correspondence indicates that generalism alone cannot explain the pronounced increase in suppression observed under higher resource diversity, suggesting that additional mechanisms must contribute to the stronger competitive responses of highly influenced strains.

Given the increasing dominance of more competitive strains in our experiments (Fig. 2f), we hypothesized that highly influenced strains would also become more abundant in natural ecosystems as resource diversity increased. To test this and extend our findings beyond the laboratory, we mapped metagenomic data from environmental samples in the EMP^19^ (Fig. 1c) to the genomes of the 298 strains tested in the sentinel assay (Fig. 3d). Because highly influenced strains show a phylogenetic signal (Fig. 3b), their close relatives are expected to share similar traits. Indeed, only reads mapping to highly influenced strains increased significantly with resource diversity, unlike mildly influenced strains or unmapped reads (Fig. 3e). A similar directional tendency is observed in most environments (Supplementary Fig. 23), albeit with variability in strength and significance. These findings suggest that highly influenced strains not only become more competitive in the laboratory, but also tend to become more abundant in natural ecosystems as resource complexity increases—suggesting the ecological relevance of our experimental results.

### Emergent resource uptake raises microbial competitiveness

**Fig. 4:**
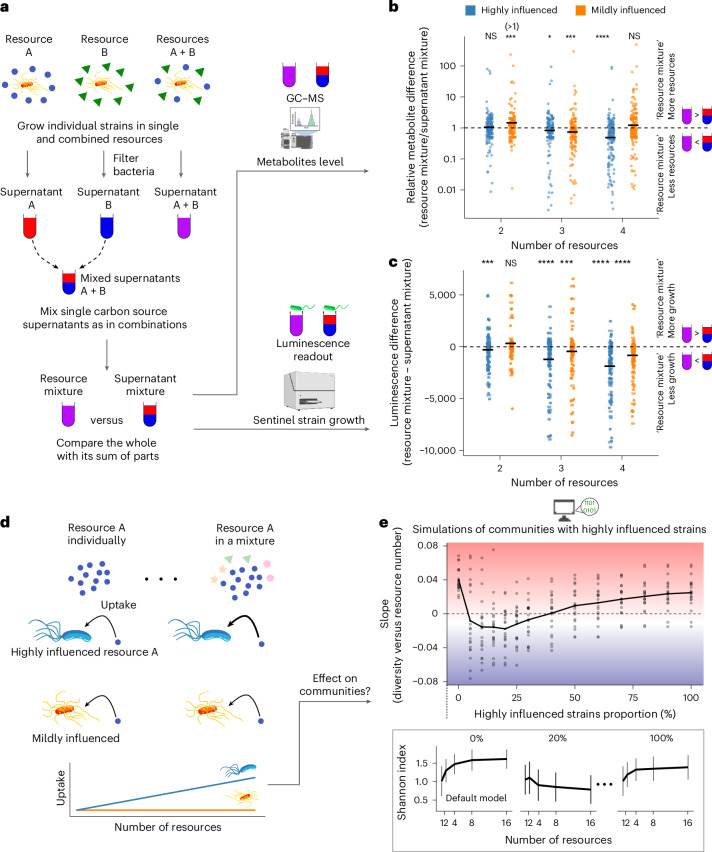
Emergent resource uptake with increasing resource complexity enhances microbial competitiveness. **a**, We tested whether highly influenced strains increase their resource uptake in mixtures beyond what is expected from independent uptake. Strains were grown in individual resources and in mixtures. Supernatants were then extracted and individual supernatants were combined to create supernatant mixtures (main text and Methods). Metabolic depletion was analysed by GC–MS and interaction effects were assessed via sentinel strain luminescence after 24 h. **b**, Highly influenced strains show greater metabolite depletion in resource mixtures than in their corresponding supernatant mixtures and this effect intensifies with increasing resource complexity. Shown is the relative change in metabolite peak area (normalized to an internal standard) for 96 identified metabolites, comparing each resource mixture to its matched supernatant mixture. Mann-Whitney *U*-tests assessed whether the median relative change deviated from 1. For mildly influenced strains in two-resource conditions, values >1 indicate less depletion (lower uptake) in the resource mixture. NS, not significant, *P* > 0.05, *P ≤ 0.05, **P ≤ 0.01, ***P ≤ 0.001, ****P ≤ 0.0001. **c**, Highly influenced strains suppress the sentinel strain more strongly in resource mixtures than in supernatant mixtures and this effect increases with resource complexity. Mildly influenced strains show the same trend, but less pronounced. Shown is the change in sentinel strain luminescence after 24 h. Significance levels as in **b**; *n* = 192. **d**, Our results indicate that highly influenced strains increase carbon uptake as resource diversity increases. For example, when a focal resource (A) is supplied alone, uptake is similar between strain types. But in a mixture, only highly influenced strains show increased uptake of A. **e**, Highly influenced strain behaviour can reduce microbial diversity as resource complexity increases in a modified consumer–resource model. We introduced a strain-specific parameter *γ* that increases uptake affinity (reduces *K*_m_) as resource diversity increases (equations in Supplementary Text 1, Section 2). We simulated communities of randomly generated strains across resource complexities using 14 highly influenced strain fractions (mean across three independent replicates, each consisting of 20 communities per fraction). Shown is the slope (*β*_1_) from a linear model of Shannon diversity versus resource number, plotted against the fraction of highly influenced strains. The 0% case corresponds to the default consumer–resource model (Fig. 1a). The trend line shows the mean ± s.d. Results are robust for a range of *γ* values (*γ* = 1 shown; Supplementary Fig. 31), where *γ* is a sensitivity parameter that controls how much highly influenced strains increase uptake as resource complexity increases (Supplementary Text 1, Section 2). Parameters were set to *ρ* = 0.4 and *e*_mean_ = 0.4 to reflect the data (Supplementary Fig. 30 and Supplementary Text 1, Sections 1.3 and 2.2.1). Illustrations in **a** and **e** partially created in BioRender; Ratzke, C. https://biorender.com/zrfhfyl (2026). Source data

The observed widespread increase in negative interactions with nutrient diversity raises the question of what drives this effect. Microbes often interact by chemically modifying their environment—for instance, by depleting shared resources. If nutrient diversity changes interactions, we might detect those changes in how the microbes alter their chemical environment. Therefore, we examined whether microbial alterations to the growth medium—capturing their metabolic footprint—could reveal the mechanism underlying the observed increase in competitiveness with resource diversity. Specifically, we grew a subset of highly and mildly influenced strains in individual- and mixed-resource settings, filtered the cultures and collected supernatants. We then compared true mixed-resource supernatants to their equivalent mixtures of single-resource supernatants (illustrated in Fig. 4a). If metabolism were unaffected by resource number, both would show similar chemical profiles. However, gas chromatography–mass spectrometry (GC–MS) showed that residual key nutrients were lower in true mixed-resource supernatants, indicating increased carbon uptake and reduced excretion of metabolic byproducts with greater resource diversity (Fig. 4c and Supplementary Fig. 24)—also providing a probable explanation for the stronger microbial growth observed in mixed resources (Fig. 2b). This effect was particularly pronounced in highly influenced strains and was observed for both supplied metabolites that were taken up during growth and produced metabolites that were secreted as byproducts (Supplementary Fig. 25).

To assess whether this enhanced uptake leads to stronger competition, we compared sentinel strain growth in true mixed-resource supernatants to its growth in corresponding mixtures of single-resource supernatants. In line with the GC–MS results, the sentinel strain grew markedly less in true mixed-resource supernatants, especially those from highly influenced strains and under higher resource diversity (Fig. 4c). This reduction was not due to toxicity, as nutrient replenishment restored growth in nearly all cases (Supplementary Fig. 26). Moreover, increasing resource number weakened facilitative effects and intensified suppressive effects, regardless of whether communities were composed exclusively of facilitative or suppressive interactions (Supplementary Fig. 27). Together, these results show that highly influenced strains increase resource uptake as resource diversity rises, causing stronger bacterial growth (Fig. 2b and Supplementary Fig. 5), leaving fewer nutrients available and creating a more competitive environment for other microbes (Fig. 4d).

As both the sentinel-independent (GC–MS results; Fig. 4b) and sentinel-dependent (suppression assays; Fig. 4c) analyses yield consistent results, this indicates that our key findings (Fig. 4c and Supplementary Figs. 16–23) are generally sentinel-independent.

We experimentally found that more nutrient diversity leads to a stronger nutrient uptake especially by a subgroup of bacteria. To test whether this stronger uptake is sufficient to explain the observed biodiversity loss (Fig. 1d), we incorporated the increasing resource consumption with nutrient diversity of highly influenced strains into the classical consumer–resource model (Supplementary Text 1; Methods). Specifically, a subset of strains was allowed to disproportionately increase resource uptake as nutrient diversity increased, reflecting the experimentally observed behaviour. Across simulations varying resource complexity and the proportion of highly influenced strains, the modified model frequently predicted a decline in biodiversity with increasing resource complexity—unlike the original model—with maximal biodiversity loss at small but non-zero fractions of highly influenced strains and a gradual shift towards positive diversity–resource relationships at higher fractions (~50%; Fig. 4e and Supplementary Fig. 28). Notably, this maximal biodiversity loss at low fractions of highly influenced strains coincided with the emergence of strong dominance by a small subset of strains (Supplementary Fig. 29). This outcome closely aligns with our experimental results, where the decrease in diversity under high-resource conditions was largely driven by a single strain (strain 79; Fig. 2a–c).

To identify when increasing resource diversity leads to biodiversity loss, we systematically explored the behaviour of the consumer–resource model across a wide range of conditions (Supplementary Text 1, Section 3). Biodiversity loss does not occur across all parameter combinations, but emerges under specific ecological conditions that we identify below. The analysis revealed three main factors that strongly shape the likelihood of biodiversity decline. Biodiversity loss is most likely when only a small but non-zero fraction of strains disproportionately benefits from nutrient diversity, allowing these strains to dominate at high-resource complexity (Fig. 4e and Supplementary Fig. 30). It is further promoted when the cross-feeding rate is high and communities already sustain high diversity under a single resource (Supplementary Fig. 30, compare across rows) and when strains that benefit most from nutrient diversity are ecological generalists rather than specialists (Supplementary Fig. 30, compare across columns). In contrast, many other model assumptions had little qualitative effect on the biodiversity pattern: the results were robust to how strongly and how heterogeneously strains responded to increasing nutrient diversity (Supplementary Figs. 31 and 32), to changes in the size of the species pool (Supplementary Fig. 34) and to the specific physiological mechanism by which resource diversity benefits highly affected strains—whether by allowing access to lower resource concentrations (reduced *K*_m_; Supplementary Fig. 30) or by increasing resource-use efficiency (Supplementary Fig. 33). The latter reflects our limited knowledge of the exact mechanism by which these strains achieve higher carbon uptake; however, our results demonstrate that, regardless of the underlying mechanism, the ecological outcome remains the same. Larger species pools mainly narrowed the conditions under which biodiversity loss occurred, without altering the underlying mechanism.

Collectively, our results show that bacteria, in particular a distinct subset of strains, become more efficient in taking up nutrients as nutrient diversity increases. This leads to more growth and stronger competition with other strains, which in turn causes more competitive exclusion in interaction pairs and loss of biodiversity in communities. An emergent physiological trait at the individual strain level thus drives the unexpected biodiversity loss at the community scale.

## Discussion

Our findings show that, contrary to ecological intuition and widely used models, increasing resource diversity in microbial communities can reduce biodiversity. This extends previous experimental work, which reported weaker-than-expected diversity increases with additional resources but not a decline^17,18^. Compared with Pacheco et al.^18^, who used a similar defined-community design, our inclusion of several communities uncovered a broader range of outcomes, including consistent diversity reductions. The much lower-than-expected increase observed in ref. ^18^ already hinted that the relationship between microbial biodiversity and resource diversity is not as simple or positive as classical consumer–resource theory suggests. The progression from weakly positive to negative responses observed here underscores that this relationship is not universal but instead depends strongly on community composition and interaction structure. While it is inherently difficult to generalize from laboratory experiments to all natural microbial communities, our results emerge from a large and phylogenetically diverse collection of isolates, suggesting that the mechanism we identify is not restricted to a particular species combination. Our results thus highlight that resource enrichment can intensify competitive exclusion, challenging the assumption of a general positive link between resource and species diversity. This interpretation is further supported by a recent study showing that increasing resource diversity synergistically intensifies competition among microbes^23^.

Since biodiversity is closely linked to ecosystem resilience^24^, our results have important implications. One particularly striking example is in the context of dietary guidelines, which often associate food variety with greater gut microbial richness^25^—yet our results suggest that this strategy may backfire. More broadly, biodiversity loss due to nutrient enrichment (for example, fertilization) is frequently observed in forest^26,27^, agricultural^28^ and aquatic ecosystems^29,30^, although these cases may involve increases in nutrient quantity rather than diversity—depending on soil composition and fertilizer type. These outcomes are typically explained by limiting-factor trade-offs, such as shifts in competition towards light^30,31^. Our study isolated resource diversity alone, keeping total nutrient concentration constant and still observed systematic diversity loss. This demonstrates that resource complexity itself can be sufficient to drive higher competition, although whether such a mechanism operates in macroscopic ecosystems remains unknown.

Conventional ecological models often ignore microbial physiology^4,32^. We show that accounting for it can radically alter predicted outcomes and improve model accuracy, as physiological shifts can cascade to reshape entire ecosystems. This finding is surprising within an ecological framework, yet the underlying physiological pattern—emergent changes in microbial resource uptake—is consistent with mounting evidence: microbes exhibit markedly different behaviour in mixed-resource environments, including diauxic shifts and lag-phase dynamics^33^ and distinct, unexpected gene expression profiles^34^. We speculate that the observed non-additive effects in mixed-resource environments may arise from such enhanced activation of genes involved in nutrient catabolism under mixed-resource conditions, a hypothesis we plan to investigate in future work. Although microbial physiology is undeniably complex, we find that viewing it at a coarse-grained level reveals a surprisingly simple and measurable link to competitive behaviour—one that is sufficient to explain community-level patterns. This echoes previous work showing that even rough measures of cellular regulation can strongly predict bacterial growth rates^35^. Together, this argues for tighter integration between microbial physiology and ecological modelling. Doing so offers a more realistic view of how microbial ecosystems behave—and why they sometimes defy expectations.

Finally, our finding revisits a fundamental question: how is microbial co-existence maintained despite competition for the same resources? While recent theories offer new perspectives on this question^12–16^, it remains unclear whether existing frameworks—alone or in combination—are sufficient to explain the diversity patterns observed in nature, or whether additional, undiscovered mechanisms shape microbial ecosystems in ways we have yet to uncover.

## Methods

### Media and bacterial culture

Bacteria were precultured from frozen stocks in nutrient medium (NM) composed of 1% yeast extract and 1% soytone (both Sigma-Aldrich) for 24 h at 30 °C in shaking (225 rpm). Following preculturing, cultures were diluted 1:5 in PBS medium and inoculated into M9 medium supplemented with the relevant carbon sources (Supplementary Table 1 gives a full list and vendor details) and 1:100 diluted NM. The NM supplement was included on the basis of preliminary tests (Supplementary Fig. 35), which showed that this small addition substantially increased the number of strains reaching measurable optical density, whereas only a few strains exhibited limited growth on the NM supplement alone in the absence of added carbon sources. This indicates a synergistic effect with defined carbon sources, probably through the alleviation of micronutrient or other abiotic limitations that would otherwise restrict growth. Inoculation was performed using an acoustic liquid handler (Echo 525, Beckman Coulter) by transferring 25 nl of 1:5 diluted culture into 40 µl of media per well (corresponding to a dilution of 1:8,000). For both community and pairwise experiments (Figs. 1 and 2), cultures were passaged daily by diluting 1:27 into fresh media (1.5 µl into 39 µl). Communities were sampled on days 9 and 10 (after eight and nine passages, respectively) and pairwise interactions were sampled on day 5 (after four passages). All experiments were conducted in 384-well plates. Community and pairwise experiments were carried out in transparent plates with lids (VWR), while sentinel strain experiments were performed in black plates with lids (Sarstedt, Lumox model) optimized for luminescence readouts.

### Resources and their mixtures

A total of 22 individual resources were used (Supplementary Table 1). These resources were selected to encompass a broad spectrum of microbial growth substrates, including sugars, amino acids, organic acids and alcohol sugars, thereby capturing the main metabolic classes that distinguish ‘sugar specialists’ and ‘acid specialists’ commonly observed among heterotrophic bacteria^36^. Resources were dispensed into 384-well plates containing M9 media without carbon sources using an acoustic liquid handler (Echo 525, Beckman) before microbial inoculation. For all resource conditions (individual or mixtures), a final concentration of 0.2% (mass/volume) was maintained. We used concentration (mass/volume) rather than molarity to keep the total available energy comparable across resources of different molecular weights, consistent with previous resource-diversity studies^17,18,37^. Mixture conditions ranged from 2 to 16 resources and were assigned randomly.

### Strain library and whole-genome sequencing

#### Community and pairwise experiments

For all community and pairwise experiments, we used 14 bacterial strains with distinct 16S rRNA gene sequences, enabling unambiguous taxonomic identification (strain names and 16S sequences listed in Supplementary Data 2). These strains are part of a *Caenorhabditis elegans*-associated isolate collection of the Ratzke laboratory and represent a subset of the 16 strains previously used in a similar context^38^.

#### Strain collection in sentinel strain experiments

For the sentinel strain experiments, we used 298 bacterial strains isolated from the gut of *C. elegans* collected from diverse natural environments, including rotting fruits, soil and compost (Supplementary Data 5). Strains were provided by C. Ratzke and H. Schulenburg. This collection spans a broad range of bacterial taxa (Supplementary Data 5 and Supplementary Fig. 19) and represents a highly diverse and ecologically relevant set of *C.* *elegans*-associated microbes suitable for controlled experimental studies.

#### Bacterial whole-genome sequencing

Of the 298 strains used in this study, 92 had been previously sequenced and assembled (Supplementary Data 5; courtesy of H. Schulenburg, collected by Johnke et al.^39^). The remaining 206 strains were sequenced as part of this work. Genomic DNA was extracted using the peqGOLD Bacteria DNA Kit (VWR). Whole-genome libraries were prepared with the NEBNext Ultra II FS DNA Library Prep Kit for Illumina (New England Biolabs), using dual index oligos for barcoding. To reduce reagent use and cost, the protocol was miniaturized to 1:7 of the standard volume. This scaled-down protocol was benchmarked against the standard 1:1 protocol and produced comparable results. Libraries were sequenced on an Illumina NovaSeq platform to a mean depth of ~120×. Raw reads were trimmed using fastp (v.0.23.2)^40^ and assembled de novo with SPAdes (v.3.15.5)^41^ using the --isolate flag and default error correction. Assemblies were generated using 24 threads per sample. Assembly quality was assessed with QUAST (v.5.2)^42^ and metrics were summarized across strains. Contamination was evaluated at the contig level using DIAMOND^43^ followed by MEGAN^44^ with GTDB taxonomy. Genomes were excluded if ≥10% of contigs were assigned to taxa inconsistent with the expected lineage, based on contradictions at or above the genus level. Sequencing statistics are in Supplementary Data 6.

#### Strain phylogeny calculation

Core-genome phylogeny was constructed using panX^45^, which clusters orthologous genes, aligns core-gene families and infers a maximum-likelihood tree from the concatenated alignment. We used default bacterial parameters and defined core genes as those present in ≥95% of strains. Taxonomy classification was done as described next.

#### Taxonomic classification of bacterial strains

Taxonomic classification of all bacterial strains was performed on the basis of their assembled genomes using a similarity-based approach against the AnnoTree reference database (v.2021). Each genome was queried with DIAMOND v.2.1.6 (blastx mode) against the AnnoTree protein database to identify the closest reference taxa, using a maximum of 25 target sequences per query and default-sensitive alignment parameters. Resulting alignment files (.daa) were filtered to retain only the top 90% of high-confidence matches. For each strain, the corresponding RefSeq or GenBank assembly accession (GCF_/GCA_) most frequently detected across all hits was selected as the best-supported taxonomic representative. These assembly identifiers were then mapped to National Center for Biotechnology Information (NCBI) taxonomy identifiers (TaxIDs) and organism names using official NCBI assembly summary files from both RefSeq and GenBank. To ensure consistency and completeness of the taxonomic hierarchy, TaxIDs were further resolved into full seven-rank lineages (kingdom, phylum, class, order, family, genus and species) using TaxonKit v.0.16.0 with the most recent NCBI taxdump. The resulting consensus table provides, for each strain, its genome accession, matched assembly reference, NCBI TaxID, organism name and the complete hierarchical taxonomic lineage.

### Luciferase tagging of the sentinel strain

*P. aeruginosa* PAO1 was genomically transformed with the luxCDABE operon using the mini-Tn7 insertion system as previously described^38^. Briefly, the plasmids pUC18T-mini-Tn7T-Gm-lux (Addgene no. 64953) and pTNS2 (Addgene no. 64968) were curated from *Escherichia coli* DH5a using a plasmid extraction kit (peqGOLD Plasmid MiniPrep Kit II; VWR). PAO1 cells were made competent for transformation by treatment with a sucrose 300 mM solution and were then electroporated with 50 ng of the curated pUC18T-mini-Tn7T-Gm-lux and pTNS2 plasmids. After recovery in 1 ml of LB medium at 30 °C, the electroporated cells were plated on an LB-agar+Gm30 plate. Plates were incubated for 24 h at 30 °C and colonies were tested for their bioluminescence activity using a plate reader. Positive colonies were then further grown in a selective medium (LB+Gm30) and were stocked in 25% glycerol at −80 °C.

### Community composition based on 16S rRNA gene sequencing

Amplicon-based 16S rRNA gene sequencing was used to profile bacterial community composition in pairwise and multispecies experiments.

#### DNA extraction

Genomic DNA was extracted using a miniaturized protocol in 384-well plates. Pellets were resuspended in TES buffer and 10 µl was transferred to fresh PCR plates. Lysis was initiated by adding metapolyzyme (in PBS) and ReadyLyse (Lucigen, 1:5 in TES), dispensed via an ECHO acoustic liquid handler, followed by overnight incubation at 30 °C with shaking (1,350 rpm). The next day, Proteinase K (New England Biolabs, 1:2 dilution of 20 mg ml^−1^ stock) and 20% SDS (1% final) were added, followed by incubation at 55 °C for 30 min. RNase A (Omega Bio-Tek, 1:10 dilution) was added and incubated for 5 min at room temperature. Proteinase K was inactivated at 95 °C for 5 min. Samples were centrifuged (2,800*g*, 10 min) and supernatants were collected. Lysates were diluted 1:6 and then 1:3 in nuclease-free water. DNA concentrations were measured using the PicoGreen assay and 0.1–10 ng was used as template for 16S amplification.

#### 16S library preparation and sequencing

Full-length 16S rRNA genes were amplified using a barcoded PCR protocol with primers 27 F and 1492 R (barcode sequences listed in Supplementary Data 7), followed by sequencing on an Oxford Nanopore platform. PCR-grade water and 2× Hot-Start PCR Master Mix (Biotechrabbit) were combined in ECHO-qualified plates and 4.4 µl of reaction mix was dispensed into 384-well PCR plates using an ECHO acoustic liquid handler (Beckman Coulter). After centrifugation, 50 nl of barcode-specific primers and 500 nl of genomic DNA were added to each well using the same system. Thermocycling was performed as follows: 94 °C for 5 min; 29 cycles of 94 °C for 60 s, 55 °C for 60 s and 72 °C for 2.5 min; followed by 72 °C for 10 min and a final hold at 4 °C. Barcoded amplicons were pooled per tube and libraries were prepared using Oxford Nanopore short-read amplicon workflow according to the manufacturer’s protocol, with halved reaction and bead-cleanup volumes. Sequencing was performed on a MinION device (Oxford Nanopore Technologies).

#### Read mapping and relative abundance estimation

Raw nanopore reads were split into 100 parts using seqkit^46^ and filtered by quality (*Q* ≥ 15) and length (1,500–1,700 base pairs) using NanoFilt (v.2.8.0)^47^. Demultiplexing was performed with a custom barcode set using minibar (https://github.com/calacademy-research/minibar), allowing edit distances up to six bases. Reverse complementation, where needed, was performed using a custom Python script (rev_cmplt_minibar_output.py). Strain-specific 16S consensus sequences were generated by pooling reads across replicates, filtering (*Q* ≥ 18) and aligning the top 1,000 reads per strain with MAFFT (v.7.508)^48^. Consensus sequences were computed using a custom script applying a 90% agreement threshold and IUPAC ambiguity codes and then combined into a FASTA file to serve as a custom reference database. Chimaeric reads were removed using vsearch (v.2.21.1)^49^ with --uchime_ref against this custom reference database. Non-chimaeric reads were mapped to the database using minimap2 (v.2.26)^50^ with -ax map-ont. Alignment files were sorted and indexed using samtools (v.1.17)^51^ and per sample read counts were generated using samtools idxstats. The final count matrix was compiled with a custom script (make_count_table_updated.py). All custom scripts and workflow code are available at https://github.com/orshalevsk/Emergent_Biodiv_loss.

### OD_600_ and bioluminescence measurement

#### OD_600_

A 384-well plate containing bacterial cultures was mixed for 15 s at 2,000 rpm using a Mixmate plate shaker (Eppendorf), then transferred to a FLUOstar Omega plate reader (BMG Labtech) to measure absorbance at 600 nm.

#### Bioluminescence

Bacterial cultures were transferred to a Lumox 384-well plate (Sarstedt) for bioluminescence measurement. First, 2.5 µl of NM solution was dispensed into each well. Then, 20 µl of bacterial culture was reverse-dispensed into the same wells using a VIAFLO 384 liquid handler (Integra) and mixed with NM for 30 s at 1,500 rpm using a Mixmate plate shaker (Eppendorf). The Lumox plate was incubated at 30 °C in a FLUOstar Omega plate reader (BMG Labtech) for 2 min to allow NM to stimulate bacterial metabolism (notably in *P. aeruginosa*), enhancing bioluminescence signal consistency. Bioluminescence was then recorded using the luminescence emission filter of the plate reader, with a gain setting of 3,600 and an exposure time of 1 s.

### Experimental design

#### Communities, pairwise and individual strains experiments

Precultures were prepared as described above (section ‘Media and bacterial culture’), then dispensed into 384-well plates containing M9 media with the appropriate carbon sources and 1:100 NM. Metadata for all conditions and strain combinations used in the community experiment are provided in Supplementary Data 1; metadata for pairwise and single-strain experiments are provided in Supplementary Data 3. All experimental conditions were fully randomized across wells and plates. At the end of each experiment, bacterial density (OD_600_) was measured for all wells, including individual strains, pairwise combinations and communities. Only pairwise and community samples were further processed for 16S sequencing: samples were centrifuged at 2,800*g* for 5 min, supernatants were removed and pellets were stored at −80 °C. DNA extraction and 16S rRNA gene sequencing procedures are described below (DNA extraction and 16S sequencing). Individual strain growth was measured after 72 h (carrying capacity, OD_600_), pairwise cultures were serially passaged for 5 days, followed by a final 24-h incubation before OD_600_ measurement and 16S profiling, and communities were serially passaged for 9 days, followed by a final 24-h incubation before OD_600_ measurement. The impact factor was computed from experimental OD_600_ data as an empirical measure of total inhibition acting on the sentinel strain. It was defined as the relative reduction in OD_600_ compared with monoculture controls (Fig. 2a). In the framework of generalized Lotka–Volterra model this impact factor corresponds to the steady-state population density of species 2 in the presence of species 1 divided by the steady-state population density of species 2 grown alone, which equals ${\alpha }_{22}\left({\alpha }_{11}-{\alpha }_{21}\right)/\left({\alpha }_{11}{\alpha }_{22}-{\alpha }_{12}{\alpha }_{21}\right)$. Accordingly, the impact factor depends on all four alphas of a pairwise interaction.

#### Sentinel strain selection rationale

We selected *P. aeruginosa* PAO1 as the sentinel because it supports stable, single-copy chromosomal integration of the luxCDABE reporter via the mini-Tn7 system—ensuring consistent reporter dosage, avoiding plasmid-borne variability and yielding a strong, reliable bioluminescence signal compatible with our plate-reader workflow. PAO1 grows robustly at 30 °C—matching the growth range of our strain collection—and exhibits broad metabolic niche breadth (generalist physiology), so its growth is sensitive to a wide range of changes in metabolic space. These practical and biological properties make PAO1 a reliable readout strain for quantifying competitive balance across diverse resource conditions. We note that any single sentinel may introduce bias.

#### Sentinel strain experiments

The full experimental setup for sentinel strain co-cultures is described above (sections ‘Media and Bacterial Culture‘ and ‘Resources and their mixtures’). Briefly, the sentinel strain and 298 co-cultured strains were grown overnight in NM medium and diluted 1:5 in PBS (without washing). Cultures were then dispensed into 384-well plates containing the appropriate media and resource conditions. Six experimental batches were performed: three using individual resources (biological replicates) and three using distinct mixed-resource conditions (no replicates). In the individual-resource experiments, all 298 strains were co-incubated with the sentinel strain across 22 single-resource conditions. The sentinel strain was also grown alone with 16 replicates per condition. In the mixed-resource experiments, one batch included all 298 strains co-incubated with the sentinel strain in all conditions. In the other two batches, 80 strains were randomly selected per condition to maximize the number of unique resource combinations tested. In these batches, the sentinel strain alone was included with eight replicates per condition. All conditions were fully randomized across wells and plates, with each experiment distributed over 20–24 separate 384-well plates per batch. Plates were incubated at 30 °C for 24 h before luminescence was measured. The average luminescence of the sentinel strain grown alone in each condition was used to calculate the impact factor of co-cultured strains, as shown in Figs. 2a and 3a. Impact factor values for individual resources represent the average across three replicate experiments (‘5’, ‘6’ and ‘7’). Full experimental conditions and metadata are provided in Supplementary Data 4.

#### Supernatant experiments

The full setup for supernatant experiments is described above (sections ‘Media and Bacterial Culture‘ and ‘Resources and their mixtures’). Briefly, selected highly influenced and mildly influenced strains were grown overnight in NM medium and diluted 1:5 in PBS (without washing). Cultures were dispensed into 384-well plates containing M9 medium supplemented with either individual- or mixed-carbon sources, specifically: sucrose, glutamic acid, citric acid, maltose, mannose, xylose, lysine, ribose, proline, fructose, histidine and asparagine. Each strain inoculated alone into four adjacent wells per condition (for example, A1, A2, B1 and B2) to ensure sufficient volume for supernatant extraction. Plates were incubated at 30 °C for 24 h. To extract supernatants, technical replicates (four wells per condition) were pooled into 96-well plates using a VIAFLO 384 liquid handler (Integra). Each set of four wells (40 µl per well) yielded a combined volume of 160 µl. The pooled cultures were filtered through 0.2-µm filter plates (PALL) into a new sterile 96-well plate, removing cells and leaving only the cell-free supernatant. This design enabled combinatorial mixing of supernatants to reconstruct mixed-carbon environments. Mixing was performed using a VIAFLO 96 liquid handler by combining supernatants from wells on different plates (for example, A1 from plate 1 with carbon A and A1 from plate 2 with carbon B were combined 1:1 to generate A + B mixtures). For the sentinel growth assay (Fig. 4c), eight strains were randomly selected—four highly influenced and four mildly influenced—were tested across 66 mixed-resource conditions. For the MS experiment (Fig. 4b), a subset of four of these strains (two highly influenced and two mildly influenced) was tested across nine mixed-resource conditions. Full strain lists and conditions are provided in Supplementary Data 8.

### GC–MS sample preparation and analysis

A total of 72 samples (20 µl each) were thawed on ice and mixed with 50 µl of internal standard solution containing 60 µM 13 C_6_-glucose and 60 µM 3-hydroxybenzoic acid (equivalent to 3,000 pmol each). Samples were dried overnight using a vacuum concentrator (Eppendorf Concentrator) to remove water, then placed in a desiccator over phosphorus pentoxide for 1 h. Derivatization was performed following a protocol adapted from ref. ^52^. Briefly, 50 µl of methoxylamine hydrochloride in pyridine (20 mg ml^−1^, freshly prepared) was added to each sample. Samples were incubated in an ultrasonic bath for 10 min, followed by shaking at 30 °C for 90 min at 1,400 rpm. After a brief centrifugation, 70 µl of *N*-methyl-*N*-trimethylsilyltrifluoroacetamide (Sigma-Aldrich) was added. Samples were then incubated at 40 °C for 60 min at 1,200 rpm and left at room temperature for an additional 2 h. Final centrifugation was performed at 15,000*g* for 10 min at 4 °C (Hettich tabletop centrifuge, swing-out rotor) and 60 µl of supernatant was transferred to GC vials for injection. GC–MS analysis was carried out on a Shimadzu TQ 8040 triple quadrupole mass spectrometer coupled to a high-performance liquid chromatography system. Chromatographic separation was achieved on a Restek Rxi-5Sil MS column. Detailed GC and MS parameters are provided in Supplementary Data 9. Data acquisition and peak integration were performed using LabSolutions Insight GC–MS software, with manual curation of peak boundaries.

### EMP data handling

#### Alpha diversity versus resource complexity analysis

Data were retrieved from the EMP study by ref. ^19^ via Qiita (study ID 13114: https://qiita.ucsd.edu/study/description/13114). We used both 16S-based alpha diversity and predicted microbially derived metabolite richness, as previously computed in ref. ^19^ (Fig. 2c and Supplementary Fig. 1c,d). Alpha diversity was taken from the column alpha_16s_deblur_nosingletons_rar5k_shannon, which reports Shannon diversity after singleton removal and rarefaction to 5,000 sequences per sample. This depth was identified by the authors as optimal for cross-sample comparison. Resource diversity was taken from alpha_lcms_fbmn_microbial_richness, defined as the number of HPLC–MS peaks >0 in each sample following feature-based molecular networking and interpreted as microbially associated metabolite richness. Samples were filtered to include only those with non-missing alpha diversity values (477 of 618 total). To ensure statistical robustness, we retained only environments (as defined by EMP level empo_4) with ≥6 samples, resulting in 462 samples across 13 distinct ecosystems. We used the empo_4 level of environmental classification, which provides the most specific ecosystem labels available in the EMP dataset and avoids confounding that can arise at broader levels (for example, empo_1), where ecologically distinct environments—such as animal guts and plants—may be grouped together.

#### Mapping metagenomic reads to highly and mildly influenced strains and abundance estimation

To assess the environmental representation of highly and mildly influenced strains, we mapped metagenomic shotgun reads from the EMP^19^ to our full genome collection of 298 experimentally characterized strains. Genome assemblies were merged into a single reference database and a minimap2 index (v.2.26)^50^ was created. Reads were aligned using minimap2 with the -ax sr --secondary=no parameters to exclude secondary alignments (pipeline: minimap2_pipline_no_multi_match.sh). Alignment files (SAM) were processed using a custom script (make_count_table_updated_with_mistmatch_filter_db.py) to filter for high-quality matches (--min_mapq 50) and compute per strain read counts, along with unmapped reads (Supplementary Data 10). To ensure sufficient coverage and minimize noise, only samples with a total mapped read count ≥10,000 were retained. This filtering yielded 453 metagenomes spanning 18 distinct ecosystems. For each sample, raw counts were normalized to relative abundances. Strains were categorized as highly or mildly influenced based on their experimentally measured impact on biodiversity and reads were binned accordingly into highly influenced, mildly influenced or unmapped groups. Relative abundances for each category were then calculated (Supplementary Data 11). These values were integrated with matched metabolomic profiles from the EMP^19^ for downstream ecological analyses. All custom scripts used in this workflow are available at https://github.com/orshalevsk/Emergent_Biodiv_loss.

### Consumer–resource model

For the classic consumer–resource model, we consider a community of 20 microbial species competing for 30 resources in a continuous dilution (chemostat-like) environment. Species take up available resources and either turn them into biomass or transform them into secreted metabolic byproducts (cross-feeding) and die through dilution. Resources are depleted through species uptake, replenished through secretion from species and are subject to dilution, which replenishes supplied resources and washes out others. For each of 20 independently parameterized communities, we varied resource complexity across 1, 2, 4, 8, 16 supplied resources (equal total supply split evenly among the chosen resources). Initial abundances were uniform (*N*_0_ = 1/20 per species). Simulations used scipy.integrate.solve_ivp v.1.16.3 in Python v.3.14.0 as ODE solver (rtol=1e-3, atol=1e-6, method = ”RK45”), over *t* = 0–800 (a.u.) with dilution of 0.1. Cross-feeding is modelled as a metabolic transformation tensor (*bᵢ*,*β* → *α*) which allows a fraction of consumed resource *β* to be secreted as resource *α* by species *i*, thereby generating new metabolites available to other species. This formulation follows standard extensions of MacArthur-type models incorporating metabolic byproduct exchange and saturating resource uptake kinetics^8,17,53^. For full details, see Supplementary Text 1, Section 1 and the full code (under ‘CR_model_simulations’).

For the highly influenced strains modified model version see Supplementary Text 1, Section 2. In this updated model, we introduce a species-specific sensitivity parameter *γ*. When *γ* > 0, the species increases its resource uptake affinity (reduces *K*ₘ) as resource diversity increases. We set *γ* = 1 for highly influenced strains (HIS) and *γ* = 0 for mildly influenced strains (MIS) by default. For each of the 20 independently parameterized communities, we vary the HIS fraction *f* = 0, 0.05, …, 1.0 (14 fractions) (Fig. 4e and Supplementary Figs. 28–34). To assign the species as either HIS or MIS, we first assigned a tendency to be highly influenced for each species *i*, which is correlated with resource generalism. We then rank the species by their tendency to be highly influenced and assign the ones with highest values as HIS according to the preassigned fraction *f* and the rest as MIS. We simulated the 20 communities under 14 HIS fractions, across the same five resource complexities (1–16) with equal total supply, *t* = 0–800, dilution of 0.1 and uniform initial abundances.

We further performed robustness tests by systematically varying key parameters and assumptions. These include: (1) cross-feeding rate; (2) correlation between highly influenced behaviour and resource generalism; (3) *γ* value for HISs; (4) a continuous instead of binary distribution of MIS and HIS behaviour; (5) an alternative mechanism where resource complexity influences HISs by increasing assimilation efficiency instead of resource affinity; and (6) the number of initial species for each community. For full details see Supplementary Text 1, Section 3 and Supplementary Figs. 30–34 and the full code (https://github.com/orshalevsk/Emergent_Biodiv_loss/).

### Inferring interaction matrices from experimental data using the generalized Lotka–Volterra model

Data from single-species growth (Supplementary Fig. 9), pairwise interactions (Fig. 2a) and community compositions (Fig. 1d,e and Supplementary Fig. 3) at steady state were used to infer Lotka–Volterra interaction matrices of the 14 strains comprising these communities. Interaction matrices were constructed for three datasets corresponding to 1, 8 and 16 available carbon sources. Since each of these three datasets contains data for several different nutrient compositions, we treated interaction strength as a random variable by using Bayesian regression to infer their values.

The steady state of the generalized Lotka–Volterra system is obtained by setting equation (1) to zero, taking into account only those species whose population densities remain non-zero. The obtained equations were fit to the data with Bayesian regression (Monte Carlo Markov Chain sampling, NUTS solver in pyro, python). As priors we used Gaussian distributions with a mean of 1 for the off-diagonal entries and a mean equal to the inverse carrying capacity for the diagonal entries. This is based on the fact that the self-interaction coefficient (*α*_self) corresponds to the inverse of the carrying capacity (*K*), a standard notation of generalized Lotka–Volterra models^32,54^. Posterior sampling was performed with 30 chains, 400 warm-up steps and 200 sampling steps. For full details, see Supplementary Text 2. Note that error bars for *α*_self_ in Fig. 2b (middle) may be overestimated as the interaction values are not independent (Supplementary Fig. 6a).

### Estimating single-species Shapley contributions to biodiversity loss

We used the interaction matrices obtained from experimental data for 1 and 16 carbon sources (methodology described in Supplementary Text 2; results in Supplementary Fig. 4) and added Gaussian noise (mean of 0, variance of 0.7) to each entry to generate a synthetic set of 14 species resembling the 14 experimentally measured strains. We produced in total 800 such sets of 14 species and took from each set randomly six or eight species, respectively (400× six species and 400× eight species). We then simulated all possible subcommunities of these six or eight species using the generalized Lotka–Volterra model under both 1- and 16-resource conditions. Since the interaction matrices were obtained from steady-state data, we could not estimate the per capita growth rates of the single species and set them to be one instead. Since the growth rate may decide for multistable cases in which state the system ends up, this choice may result in different steady states compared with experimentally parametrized or randomized growth rates. (Supplementary Fig. 10). For each simulated subcommunity, we computed the steady-state Shannon diversity index under both conditions and calculated the change in diversity as: *Δ*Shannon = Shannon_16_ − Shannon_1_. We then computed Shapley values, representing the contribution of each strain in a six- or eight-species community to the overall change in Shannon diversity between 1 and 16 resources. Next, using the same interaction matrices (Supplementary Fig. 4), we calculated the mean impact factor for each species on the other members of its community under both resource conditions—analogous to the analysis shown in Fig. 2d. Finally, we computed the change in mean impact factor with increasing resource diversity and plotted these values against the corresponding Shapley values for each species (Fig. 2c).

### Reporting summary

Further information on research design is available in the Nature Portfolio Reporting Summary linked to this article.

## Supplementary information

Supplementary Information Supplementary Figs. 1–35, Tables 1–3 and Texts 1 and 2.

Reporting Summary

Peer Review File

Supplementary Data 1 Metadata for all experimental microbial communities used in the resource diversity experiments (strain composition and conditions), underlying Fig. 1d,e and Supplementary Figs. 2–4.

Supplementary Data 2 Metadata and strain information for the 14 bacterial isolates used to construct synthetic communities, underlying Fig. 1d and Fig. 2.

Supplementary Data 3 Metadata for pairwise interaction experiments used to infer interaction coefficients and construct interaction matrices, underlying Fig. 2a,b and Supplementary Figs. 6–9.

Supplementary Data 4 High-throughput sentinel strain interaction dataset (~32,000 measurements) used to quantify impact factors across resource conditions, underlying Fig. 3a–c and Supplementary Figs. 16–18.

Supplementary Data 5 Complete strain collection with genome accessions and taxonomic annotations used for phylogenetic and environmental mapping analyses, underlying Fig. 3b,d and Supplementary Figs. 19 and 22.

Supplementary Data 6 Sequencing quality metrics and assembly statistics for all genomes generated in this study, supporting Methods (genome sequencing and assembly quality control).

Supplementary Data 7 Primer sequences used for full-length 16S rRNA gene amplification and barcoding, supporting Methods (16S sequencing workflow).

Supplementary Data 8 Supernatant experiment data quantifying metabolic effects and interaction outcomes in individual versus mixed-resource conditions, underlying Fig. 4a–c and Supplementary Figs. 24–27.

Supplementary Data 9 Detailed chromatographic and mass spectrometry acquisition parameters used for metabolomic profiling, supporting Fig. 4b and Methods (GC–MS analysis).

Supplementary Data 10 Binned relative abundance data from EMP samples used for ecological correlation analysis, underlying Figs. 1c and 3e.

Supplementary Data 11 Raw relative abundance data from EMP samples before processing, underlying Figs. 1c and 3e.

## Source data

Source Data Fig. 1 Statistical source data.

Source Data Fig. 2 Statistical source data.

Source Data Fig. 3 Statistical source data.

Source Data Fig. 4 Statistical source data.

## Data Availability

All whole-genome sequencing data generated in this study have been deposited in the European Nucleotide Archive (ENA) under accession number PRJEB112140 and are publicly available. The raw and processed datasets supporting the findings of this study are provided as Supplementary Data 1–11. Source data are provided with this paper.

## Code availability

The code used in this study is available via GitHub at https://github.com/orshalevsk/Emergent_Biodiv_loss.
