# Supplementary material for: Increased nutrient diversity can induce loss of microbial diversity through enhanced resource uptake
Source: Nat Ecol Evol. 2026 Jul 20;10(8):1449–63. doi: 10.1038/s41559-026-03111-4 (PMC13441979; doi:10.1038/s41559-026-03111-4)
Supplement: Supplementary file 1 [file 41559_2026_3111_MOESM12_ESM.pdf]

Method

[Comment]

===== Analytical Line 1 =====

[GC-2010]

Column Oven Temp.	:100.0 °C
Injection Temp.	:280.00 °C
Injection Mode	:Splitless
Sampling Time	:1.00 min
Flow Control Mode	:Linear Velocity
Pressure	:51.0 kPa
Total Flow	:15.1 mL/min
Column Flow	:0.92 mL/min
Linear Velocity	:39.0 cm/sec
Purge Flow	:5.0 mL/min
Split Ratio	:10.0
High Pressure Injection	:OFF
Carrier Gas Saver	:OFF

Oven Temp. Program

Rate	Temperature(°C)	Hold Time(min)
-	100.0	4.00
10.00	320.0	15.00

< Ready Check Heat Unit >

Column Oven	: Yes
SPL1	: Yes
MS	: Yes
SPL2	: Yes
MS	: Yes

< Ready Check Detector(FTD/BID) >

< Ready Check Baseline Drift >

< Ready Check Injection Flow >

SPL1 Carrier	: Yes
SPL1 Purge	: Yes
SPL2 Carrier	: Yes
SPL2 Purge	: Yes

< Ready Check APC Flow >

< Ready Check Detector APC Flow >

External Wait	:No
Equilibrium Time	:1.0 min

[GC Program]

===== Analytical Line 2 =====

[AOC-20i+s]

# of Rinses with Presolvent	:3
# of Rinses with Solvent(post)	:3
# of Rinses with Sample	:0
Plunger Speed(Suction)	:High
Viscosity Comp. Time	:0.2 sec
Plunger Speed(Injection)	:High
Syringe Insertion Speed	:High
Injection Mode	:Normal
Pumping Times	:0
Inj. Port Dwell Time	:0.3 sec
Terminal Air Gap	:No
Plunger Washing Speed	:High
Washing Volume	:8uL
Syringe Suction Position	:0.0 mm
Syringe Injection Position	:0.0 mm
Solvent Selection	:All A,B,C

[GC-2010]

Injection Temp.	:280.00 °C
Injection Mode	:Split
Flow Control Mode	:Linear Velocity
Pressure	:68.1 kPa
Total Flow	:16.2 mL/min
Column Flow	:1.02 mL/min
Linear Velocity	:39.0 cm/sec
Purge Flow	:5.0 mL/min
Split Ratio	:10.0
High Pressure Injection	:OFF
Carrier Gas Saver	:OFF
Splitter Hold	:OFF

Oven Temp. Program

Rate	Temperature(°C)	Hold Time(min)
-	100.0	4.00
10.00	320.0	15.00

[GCMS-TQ8040]
IonSourceTemp :200.00 °C
Interface Temp. :280.00 °C
Solvent Cut Time :1.65 min
Detector Gain Mode :Relative to the Tuning Result
Detector Gain :+0.00 kV
Threshold :0
Acquire Data without Using CID Gas(Q3Scan) :ON

[MS Table]
--Group 1 - Event 1--
Compound Name :
Start Time :2.36min
End Time :3.92min
Acq. Mode :Q3 Scan
Event Time :0.300sec
Scan Speed :2000
Start m/z :45.00
End m/z :600.00
Q1 Resolution : -
Q3 Resolution : -
--Group 1 - Event 2--
Compound Name :Pyruvic acid-meto-TMS
Start Time :2.36min
End Time :3.92min
Acq. Mode :MRM
Event Time :0.150sec
Q1 Resolution :Unit
Q3 Resolution :Low
Ch1-m/z :(Precursor)174.00 (Product)74.00 (CE)21.00V
Ch2-m/z :(Precursor)174.00 (Product)89.00 (CE)9.00V
--Group 1 - Event 3--
Compound Name :Lactic acid-2TMS
Start Time :2.36min
End Time :3.92min
Acq. Mode :MRM
Event Time :0.150sec
Q1 Resolution :Unit
Q3 Resolution :Low
Ch1-m/z :(Precursor)191.10 (Product)147.10 (CE)9.00V
Ch2-m/z :(Precursor)219.00 (Product)147.10 (CE)15.00V
--Group 2 - Event 1--
Compound Name :
Start Time :3.92min
End Time :5.52min
Acq. Mode :Q3 Scan
Event Time :0.300sec
Scan Speed :2000
Start m/z :45.00
End m/z :600.00
Q1 Resolution : -
Q3 Resolution : -
--Group 2 - Event 2--
Compound Name :Alanine-2TMS
Start Time :3.92min
End Time :5.52min
Acq. Mode :MRM
Event Time :0.150sec
Q1 Resolution :Unit
Q3 Resolution :Low
Ch1-m/z :(Precursor)116.10 (Product)73.00 (CE)15.00V
Ch2-m/z :(Precursor)190.10 (Product)147.10 (CE)9.00V
--Group 2 - Event 3--
Compound Name :Oxalic acid-2TMS
Start Time :3.92min
End Time :5.52min
Acq. Mode :MRM
Event Time :0.150sec
Q1 Resolution :Unit
Q3 Resolution :Low
Ch1-m/z :(Precursor)190.10 (Product)147.10 (CE)9.00V
Ch2-m/z :(Precursor)219.10 (Product)147.10 (CE)9.00V
--Group 3 - Event 1--
Compound Name :
Start Time :5.52min
End Time :6.94min

Acq. Mode	:Q3 Scan		
Event Time	:0.300sec		
Scan Speed	:2000		
Start m/z	:45.00		
End m/z	:600.00		
Q1 Resolution	: -		
Q3 Resolution	: -		
--Group 3 - Event 2--			
Compound Name	:Malonic acid-2TMS		
Start Time	:5.52min		
End Time	:6.94min		
Acq. Mode	:MRM		
Event Time	:0.150sec		
Q1 Resolution	:Unit		
Q3 Resolution	:Low		
Ch1-m/z	:(Precursor)233.10	(Product)147.10	(CE)9.00V
Ch2-m/z	:(Precursor)133.10	(Product)115.00	(CE)15.00V
--Group 3 - Event 3--			
Compound Name	:Valine-2TMS		
Start Time	:5.52min		
End Time	:6.94min		
Acq. Mode	:MRM		
Event Time	:0.150sec		
Q1 Resolution	:Unit		
Q3 Resolution	:Low		
Ch1-m/z	:(Precursor)144.10	(Product)73.00	(CE)18.00V
Ch2-m/z	:(Precursor)218.10	(Product)147.10	(CE)9.00V
--Group 4 - Event 1--			
Compound Name	:		
Start Time	:6.94min		
End Time	:8.03min		
Acq. Mode	:Q3 Scan		
Event Time	:0.160sec		
Scan Speed	: 51		
Start m/z	:314.00		
End m/z	:315.00		
Q1 Resolution	: -		
Q3 Resolution	: -		
--Group 4 - Event 2--			
Compound Name	:Leucine-2TMS		
Start Time	:6.94min		
End Time	:8.03min		
Acq. Mode	:MRM		
Event Time	:0.047sec		
Q1 Resolution	:Unit		
Q3 Resolution	:Low		
Ch1-m/z	:(Precursor)158.10	(Product)73.00	(CE)15.00V
Ch2-m/z	:(Precursor)158.10	(Product)102.10	(CE)9.00V
--Group 4 - Event 3--			
Compound Name	:Isoleucine-2TMS		
Start Time	:6.94min		
End Time	:8.03min		
Acq. Mode	:MRM		
Event Time	:0.046sec		
Q1 Resolution	:Unit		
Q3 Resolution	:Low		
Ch1-m/z	:(Precursor)158.20	(Product)73.00	(CE)12.00V
Ch2-m/z	:(Precursor)218.10	(Product)73.00	(CE)15.00V
--Group 4 - Event 4--			
Compound Name	:Proline-2TMS		
Start Time	:6.94min		
End Time	:8.03min		
Acq. Mode	:MRM		
Event Time	:0.046sec		
Q1 Resolution	:Unit		
Q3 Resolution	:Low		
Ch1-m/z	:(Precursor)142.10	(Product)73.00	(CE)18.00V
Ch2-m/z	:(Precursor)216.10	(Product)147.10	(CE)18.00V
--Group 4 - Event 5--			
Compound Name	:C13		
Start Time	:6.94min		
End Time	:8.03min		
Acq. Mode	:Q3 SIM		
Event Time	:0.069sec		
Q1 Resolution	: -		
Q3 Resolution	:High		
Ch1-m/z	:57.10		
Ch2-m/z	:184.25		
Ch3-m/z	:71.10		
--Group 4 - Event 6--			
Compound Name	:Glycine-3TMS		

Start Time	:6.94min		
End Time	:8.03min		
Acq. Mode	:MRM		
Event Time	:0.046sec		
Q1 Resolution	:Unit		
Q3 Resolution	:Low		
Ch1-m/z	:(Precursor)174.10	(Product)73.00	(CE)15.00V
Ch2-m/z	:(Precursor)174.10	(Product)86.00	(CE)12.00V
--Group 4 - Event 7--			
Compound Name	:Succinic acid-2TMS		
Start Time	:6.94min		
End Time	:8.03min		
Acq. Mode	:MRM		
Event Time	:0.046sec		
Q1 Resolution	:Unit		
Q3 Resolution	:Low		
Ch1-m/z	:(Precursor)247.10	(Product)147.10	(CE)6.00V
Ch2-m/z	:(Precursor)172.10	(Product)73.00	(CE)15.00V
--Group 5 - Event 1--			
Compound Name	:		
Start Time	:8.03min		
End Time	:9.09min		
Acq. Mode	:Q3 Scan		
Event Time	:0.300sec		
Scan Speed	:2000		
Start m/z	:45.00		
End m/z	:600.00		
Q1 Resolution	: -		
Q3 Resolution	: -		
--Group 5 - Event 2--			
Compound Name	:Isoleucine-2TMS		
Start Time	:8.03min		
End Time	:9.09min		
Acq. Mode	:MRM		
Event Time	:0.036sec		
Q1 Resolution	:Unit		
Q3 Resolution	:Low		
Ch1-m/z	:(Precursor)158.20	(Product)73.00	(CE)12.00V
Ch2-m/z	:(Precursor)218.10	(Product)73.00	(CE)15.00V
--Group 5 - Event 3--			
Compound Name	:Proline-2TMS		
Start Time	:8.03min		
End Time	:9.09min		
Acq. Mode	:MRM		
Event Time	:0.036sec		
Q1 Resolution	:Unit		
Q3 Resolution	:Low		
Ch1-m/z	:(Precursor)142.10	(Product)73.00	(CE)18.00V
Ch2-m/z	:(Precursor)216.10	(Product)147.10	(CE)18.00V
--Group 5 - Event 4--			
Compound Name	:C13		
Start Time	:8.03min		
End Time	:9.09min		
Acq. Mode	:Q3 SIM		
Event Time	:0.053sec		
Q1 Resolution	: -		
Q3 Resolution	:High		
Ch1-m/z	:57.10		
Ch2-m/z	:184.25		
Ch3-m/z	:71.10		
--Group 5 - Event 5--			
Compound Name	:Glycine-3TMS		
Start Time	:8.03min		
End Time	:9.09min		
Acq. Mode	:MRM		
Event Time	:0.035sec		
Q1 Resolution	:Unit		
Q3 Resolution	:Low		
Ch1-m/z	:(Precursor)174.10	(Product)73.00	(CE)15.00V
Ch2-m/z	:(Precursor)174.10	(Product)86.00	(CE)12.00V
--Group 5 - Event 6--			
Compound Name	:Succinic acid-2TMS		
Start Time	:8.03min		
End Time	:9.09min		
Acq. Mode	:MRM		
Event Time	:0.035sec		
Q1 Resolution	:Unit		
Q3 Resolution	:Low		
Ch1-m/z	:(Precursor)247.10	(Product)147.10	(CE)6.00V
Ch2-m/z	:(Precursor)172.10	(Product)73.00	(CE)15.00V
--Group 5 - Event 7--			

Compound Name	:Fumaric acid-2TMS		
Start Time	:8.03min		
End Time	:9.09min		
Acq. Mode	:MRM		
Event Time	:0.035sec		
Q1 Resolution	:Unit		
Q3 Resolution	:Low		
Ch1-m/z	:(Precursor)245.00	(Product)147.10	(CE)15.00V
Ch2-m/z	:(Precursor)143.10	(Product)75.00	(CE)18.00V
--Group 5 - Event 8--			
Compound Name	:Serine-3TMS		
Start Time	:8.03min		
End Time	:9.09min		
Acq. Mode	:MRM		
Event Time	:0.035sec		
Q1 Resolution	:Unit		
Q3 Resolution	:Low		
Ch1-m/z	:(Precursor)204.10	(Product)73.00	(CE)18.00V
Ch2-m/z	:(Precursor)218.10	(Product)73.00	(CE)18.00V
--Group 5 - Event 9--			
Compound Name	:Threonine-3TMS		
Start Time	:8.03min		
End Time	:9.09min		
Acq. Mode	:MRM		
Event Time	:0.035sec		
Q1 Resolution	:Unit		
Q3 Resolution	:Low		
Ch1-m/z	:(Precursor)218.10	(Product)73.00	(CE)18.00V
Ch2-m/z	:(Precursor)291.00	(Product)101.10	(CE)18.00V
--Group 6 - Event 1--			
Compound Name	:		
Start Time	:9.09min		
End Time	:10.18min		
Acq. Mode	:Q3 Scan		
Event Time	:0.300sec		
Scan Speed	:2000		
Start m/z	:45.00		
End m/z	:600.00		
Q1 Resolution	: -		
Q3 Resolution	: -		
--Group 6 - Event 2--			
Compound Name	:Serine-3TMS		
Start Time	:9.09min		
End Time	:10.18min		
Acq. Mode	:MRM		
Event Time	:0.100sec		
Q1 Resolution	:Unit		
Q3 Resolution	:Low		
Ch1-m/z	:(Precursor)204.10	(Product)73.00	(CE)18.00V
Ch2-m/z	:(Precursor)218.10	(Product)73.00	(CE)18.00V
--Group 6 - Event 3--			
Compound Name	:Threonine-3TMS		
Start Time	:9.09min		
End Time	:10.18min		
Acq. Mode	:MRM		
Event Time	:0.100sec		
Q1 Resolution	:Unit		
Q3 Resolution	:Low		
Ch1-m/z	:(Precursor)218.10	(Product)73.00	(CE)18.00V
Ch2-m/z	:(Precursor)291.00	(Product)101.10	(CE)18.00V
--Group 6 - Event 4--			
Compound Name	:Hydroquinone-2TMS		
Start Time	:9.09min		
End Time	:10.18min		
Acq. Mode	:MRM		
Event Time	:0.100sec		
Q1 Resolution	:Unit		
Q3 Resolution	:Low		
Ch1-m/z	:(Precursor)254.10	(Product)239.10	(CE)12.00V
Ch2-m/z	:(Precursor)239.10	(Product)73.00	(CE)21.00V
--Group 7 - Event 1--			
Compound Name	:		
Start Time	:10.18min		
End Time	:11.45min		
Acq. Mode	:Q3 Scan		
Event Time	:0.300sec		
Scan Speed	:2000		
Start m/z	:45.00		
End m/z	:600.00		
Q1 Resolution	: -		
Q3 Resolution	: -		

--Group 7 - Event 2--

Compound Name	:Malic acid-3TMS		
Start Time	:10.18min		
End Time	:11.45min		
Acq. Mode	:MRM		
Event Time	:0.050sec		
Q1 Resolution	:Unit		
Q3 Resolution	:Low		
Ch1-m/z	:(Precursor)335.00	(Product)147.10	(CE)15.00V
Ch2-m/z	:(Precursor)307.00	(Product)147.10	(CE)27.00V

--Group 7 - Event 3--

Compound Name	:C15
Start Time	:10.18min
End Time	:11.45min
Acq. Mode	:Q3 SIM
Event Time	:0.075sec
Q1 Resolution	: -
Q3 Resolution	:High
Ch1-m/z	:57.05
Ch2-m/z	:212.30
Ch3-m/z	:71.10

--Group 7 - Event 4--

Compound Name	:Salicylic acid-2TMS		
Start Time	:10.18min		
End Time	:11.45min		
Acq. Mode	:MRM		
Event Time	:0.075sec		
Q1 Resolution	:Unit		
Q3 Resolution	:Low		
Ch1-m/z	:(Precursor)267.00	(Product)73.00	(CE)18.00V
Ch2-m/z	:(Precursor)209.00	(Product)91.00	(CE)15.00V
Ch3-m/z	:(Precursor)267.00	(Product)149.00	(CE)9.00V

--Group 7 - Event 5--

Compound Name	:Methionine-2TMS		
Start Time	:10.18min		
End Time	:11.45min		
Acq. Mode	:MRM		
Event Time	:0.050sec		
Q1 Resolution	:Unit		
Q3 Resolution	:Low		
Ch1-m/z	:(Precursor)250.10	(Product)147.10	(CE)15.00V
Ch2-m/z	:(Precursor)293.10	(Product)232.10	(CE)9.00V

--Group 7 - Event 6--

Compound Name	:Cysteine-3TMS		
Start Time	:10.18min		
End Time	:11.45min		
Acq. Mode	:MRM		
Event Time	:0.050sec		
Q1 Resolution	:Unit		
Q3 Resolution	:Low		
Ch1-m/z	:(Precursor)220.10	(Product)73.00	(CE)24.00V
Ch2-m/z	:(Precursor)218.10	(Product)73.00	(CE)24.00V

--Group 8 - Event 1--

Compound Name	:
Start Time	:11.45min
End Time	:12.19min
Acq. Mode	:Q3 Scan
Event Time	:0.300sec
Scan Speed	:2000
Start m/z	:45.00
End m/z	:600.00
Q1 Resolution	: -
Q3 Resolution	: -

--Group 8 - Event 2--

Compound Name	:Methionine-2TMS		
Start Time	:11.45min		
End Time	:12.19min		
Acq. Mode	:MRM		
Event Time	:0.086sec		
Q1 Resolution	:Unit		
Q3 Resolution	:Low		
Ch1-m/z	:(Precursor)250.10	(Product)147.10	(CE)15.00V
Ch2-m/z	:(Precursor)293.10	(Product)232.10	(CE)9.00V

--Group 8 - Event 3--

Compound Name	:Cysteine-3TMS
Start Time	:11.45min
End Time	:12.19min
Acq. Mode	:MRM
Event Time	:0.086sec
Q1 Resolution	:Unit
Q3 Resolution	:Low

Ch1-m/z	:(Precursor)220.10	(Product)73.00	(CE)24.00V
Ch2-m/z	:(Precursor)218.10	(Product)73.00	(CE)24.00V
--Group 8 - Event 4--			
Compound Name	:3-Hydroxybenzoic acid-2TMS		
Start Time	:11.45min		
End Time	:12.19min		
Acq. Mode	:MRM		
Event Time	:0.128sec		
Q1 Resolution	:Unit		
Q3 Resolution	:Low		
Ch1-m/z	:(Precursor)267.00	(Product)193.00	(CE)15.00V
Ch2-m/z	:(Precursor)267.00	(Product)73.00	(CE)27.00V
Ch3-m/z	:(Precursor)267.00	(Product)223.00	(CE)9.00V
--Group 9 - Event 1--			
Compound Name	:		
Start Time	:12.19min		
End Time	:12.66min		
Acq. Mode	:Q3 Scan		
Event Time	:0.300sec		
Scan Speed	:2000		
Start m/z	:45.00		
End m/z	:600.00		
Q1 Resolution	: -		
Q3 Resolution	: -		
--Group 9 - Event 2--			
Compound Name	:Glutamic acid-3TMS		
Start Time	:12.19min		
End Time	:12.66min		
Acq. Mode	:MRM		
Event Time	:0.050sec		
Q1 Resolution	:Unit		
Q3 Resolution	:Low		
Ch1-m/z	:(Precursor)246.10	(Product)73.00	(CE)24.00V
Ch2-m/z	:(Precursor)246.10	(Product)128.10	(CE)15.00V
--Group 9 - Event 3--			
Compound Name	:Phenylalanine-2TMS		
Start Time	:12.19min		
End Time	:12.66min		
Acq. Mode	:MRM		
Event Time	:0.050sec		
Q1 Resolution	:Unit		
Q3 Resolution	:Low		
Ch1-m/z	:(Precursor)218.10	(Product)73.00	(CE)15.00V
Ch2-m/z	:(Precursor)192.10	(Product)73.00	(CE)15.00V
--Group 9 - Event 4--			
Compound Name	:4-Hydroxybenzoic acid-2TMS		
Start Time	:12.19min		
End Time	:12.66min		
Acq. Mode	:MRM		
Event Time	:0.050sec		
Q1 Resolution	:Unit		
Q3 Resolution	:Low		
Ch1-m/z	:(Precursor)267.10	(Product)193.10	(CE)18.00V
Ch2-m/z	:(Precursor)267.10	(Product)223.10	(CE)9.00V
--Group 9 - Event 5--			
Compound Name	:Asparagine-4TMS		
Start Time	:12.19min		
End Time	:12.66min		
Acq. Mode	:MRM		
Event Time	:0.050sec		
Q1 Resolution	:Unit		
Q3 Resolution	:Low		
Ch1-m/z	:(Precursor)231.10	(Product)216.10	(CE)6.00V
Ch2-m/z	:(Precursor)303.10	(Product)73.00	(CE)24.00V
--Group 9 - Event 6--			
Compound Name	:Tartaric acid-4TMS		
Start Time	:12.19min		
End Time	:12.66min		
Acq. Mode	:MRM		
Event Time	:0.050sec		
Q1 Resolution	:Unit		
Q3 Resolution	:Low		
Ch1-m/z	:(Precursor)292.10	(Product)73.00	(CE)24.00V
Ch2-m/z	:(Precursor)292.10	(Product)102.10	(CE)15.00V
--Group 9 - Event 7--			
Compound Name	:Xylose-meto-4TMS(1)		
Start Time	:12.19min		
End Time	:12.66min		
Acq. Mode	:MRM		
Event Time	:0.050sec		
Q1 Resolution	:Unit		

Q3 Resolution	:Low		
Ch1-m/z	:(Precursor)307.10	(Product)217.10	(CE)6.00V
Ch2-m/z	:(Precursor)307.10	(Product)73.00	(CE)30.00V
--Group 10 - Event 1--			
Compound Name	:		
Start Time	:12.66min		
End Time	:13.30min		
Acq. Mode	:Q3 Scan		
Event Time	:0.300sec		
Scan Speed	:2000		
Start m/z	:45.00		
End m/z	:600.00		
Q1 Resolution	: -		
Q3 Resolution	: -		
--Group 10 - Event 2--			
Compound Name	:Glutamic acid-3TMS		
Start Time	:12.66min		
End Time	:13.30min		
Acq. Mode	:MRM		
Event Time	:0.034sec		
Q1 Resolution	:Unit		
Q3 Resolution	:Low		
Ch1-m/z	:(Precursor)246.10	(Product)73.00	(CE)24.00V
Ch2-m/z	:(Precursor)246.10	(Product)128.10	(CE)15.00V
--Group 10 - Event 3--			
Compound Name	:Phenylalanine-2TMS		
Start Time	:12.66min		
End Time	:13.30min		
Acq. Mode	:MRM		
Event Time	:0.034sec		
Q1 Resolution	:Unit		
Q3 Resolution	:Low		
Ch1-m/z	:(Precursor)218.10	(Product)73.00	(CE)15.00V
Ch2-m/z	:(Precursor)192.10	(Product)73.00	(CE)15.00V
--Group 10 - Event 4--			
Compound Name	:4-Hydroxybenzoic acid-2TMS		
Start Time	:12.66min		
End Time	:13.30min		
Acq. Mode	:MRM		
Event Time	:0.034sec		
Q1 Resolution	:Unit		
Q3 Resolution	:Low		
Ch1-m/z	:(Precursor)267.10	(Product)193.10	(CE)18.00V
Ch2-m/z	:(Precursor)267.10	(Product)223.10	(CE)9.00V
--Group 10 - Event 5--			
Compound Name	:Asparagine-4TMS		
Start Time	:12.66min		
End Time	:13.30min		
Acq. Mode	:MRM		
Event Time	:0.033sec		
Q1 Resolution	:Unit		
Q3 Resolution	:Low		
Ch1-m/z	:(Precursor)231.10	(Product)216.10	(CE)6.00V
Ch2-m/z	:(Precursor)303.10	(Product)73.00	(CE)24.00V
--Group 10 - Event 6--			
Compound Name	:Tartaric acid-4TMS		
Start Time	:12.66min		
End Time	:13.30min		
Acq. Mode	:MRM		
Event Time	:0.033sec		
Q1 Resolution	:Unit		
Q3 Resolution	:Low		
Ch1-m/z	:(Precursor)292.10	(Product)73.00	(CE)24.00V
Ch2-m/z	:(Precursor)292.10	(Product)102.10	(CE)15.00V
--Group 10 - Event 7--			
Compound Name	:Xylose-meto-4TMS(1)		
Start Time	:12.66min		
End Time	:13.30min		
Acq. Mode	:MRM		
Event Time	:0.033sec		
Q1 Resolution	:Unit		
Q3 Resolution	:Low		
Ch1-m/z	:(Precursor)307.10	(Product)217.10	(CE)6.00V
Ch2-m/z	:(Precursor)307.10	(Product)73.00	(CE)30.00V
--Group 10 - Event 8--			
Compound Name	:4-Aminobenzoic acid-TMS		
Start Time	:12.66min		
End Time	:13.30min		
Acq. Mode	:MRM		
Event Time	:0.033sec		
Q1 Resolution	:Unit		

Q3 Resolution	:Low		
Ch1-m/z	:(Precursor)194.10	(Product)120.10	(CE)18.00V
Ch2-m/z	:(Precursor)194.10	(Product)150.10	(CE)9.00V
--Group 10 - Event 9--			
Compound Name	:Asparagine-3TMS		
Start Time	:12.66min		
End Time	:13.30min		
Acq. Mode	:MRM		
Event Time	:0.033sec		
Q1 Resolution	:Unit		
Q3 Resolution	:Low		
Ch1-m/z	:(Precursor)188.10	(Product)73.00	(CE)24.00V
Ch2-m/z	:(Precursor)231.10	(Product)132.10	(CE)12.00V
--Group 10 - Event 10--			
Compound Name	:Fucose-meto-4TMS(1)		
Start Time	:12.66min		
End Time	:13.30min		
Acq. Mode	:MRM		
Event Time	:0.033sec		
Q1 Resolution	:Unit		
Q3 Resolution	:Low		
Ch1-m/z	:(Precursor)160.00	(Product)73.00	(CE)18.00V
Ch2-m/z	:(Precursor)277.00	(Product)73.00	(CE)24.00V
--Group 11 - Event 1--			
Compound Name	:		
Start Time	:13.30min		
End Time	:14.05min		
Acq. Mode	:Q3 Scan		
Event Time	:0.300sec		
Scan Speed	:2000		
Start m/z	:45.00		
End m/z	:600.00		
Q1 Resolution	: -		
Q3 Resolution	: -		
--Group 11 - Event 2--			
Compound Name	:4-Aminobenzoic acid-TMS		
Start Time	:13.30min		
End Time	:14.05min		
Acq. Mode	:MRM		
Event Time	:0.043sec		
Q1 Resolution	:Unit		
Q3 Resolution	:Low		
Ch1-m/z	:(Precursor)194.10	(Product)120.10	(CE)18.00V
Ch2-m/z	:(Precursor)194.10	(Product)150.10	(CE)9.00V
--Group 11 - Event 3--			
Compound Name	:Asparagine-3TMS		
Start Time	:13.30min		
End Time	:14.05min		
Acq. Mode	:MRM		
Event Time	:0.043sec		
Q1 Resolution	:Unit		
Q3 Resolution	:Low		
Ch1-m/z	:(Precursor)188.10	(Product)73.00	(CE)24.00V
Ch2-m/z	:(Precursor)231.10	(Product)132.10	(CE)12.00V
--Group 11 - Event 4--			
Compound Name	:Fucose-meto-4TMS(1)		
Start Time	:13.30min		
End Time	:14.05min		
Acq. Mode	:MRM		
Event Time	:0.043sec		
Q1 Resolution	:Unit		
Q3 Resolution	:Low		
Ch1-m/z	:(Precursor)160.00	(Product)73.00	(CE)18.00V
Ch2-m/z	:(Precursor)277.00	(Product)73.00	(CE)24.00V
--Group 11 - Event 5--			
Compound Name	:Fucose-meto-4TMS(2)		
Start Time	:13.30min		
End Time	:14.05min		
Acq. Mode	:MRM		
Event Time	:0.043sec		
Q1 Resolution	:Unit		
Q3 Resolution	:Low		
Ch1-m/z	:(Precursor)160.00	(Product)73.00	(CE)12.00V
Ch2-m/z	:(Precursor)277.00	(Product)73.00	(CE)24.00V
--Group 11 - Event 6--			
Compound Name	:Cysteic acid-3TMS		
Start Time	:13.30min		
End Time	:14.05min		
Acq. Mode	:MRM		
Event Time	:0.043sec		
Q1 Resolution	:Unit		

Q3 Resolution	:Low		
Ch1-m/z	:(Precursor)268.00	(Product)73.00	(CE)21.00V
Ch2-m/z	:(Precursor)268.00	(Product)106.10	(CE)12.00V
--Group 11 - Event 7--			
Compound Name	:Glutamine-4TMS		
Start Time	:13.30min		
End Time	:14.05min		
Acq. Mode	:MRM		
Event Time	:0.043sec		
Q1 Resolution	:Unit		
Q3 Resolution	:Low		
Ch1-m/z	:(Precursor)203.10	(Product)147.10	(CE)18.00V
Ch2-m/z	:(Precursor)227.10	(Product)73.00	(CE)21.00V
--Group 11 - Event 8--			
Compound Name	:Glutamine-3TMS		
Start Time	:13.30min		
End Time	:14.05min		
Acq. Mode	:MRM		
Event Time	:0.042sec		
Q1 Resolution	:Unit		
Q3 Resolution	:Low		
Ch1-m/z	:(Precursor)245.10	(Product)156.10	(CE)9.00V
Ch2-m/z	:(Precursor)245.10	(Product)73.00	(CE)21.00V
--Group 12 - Event 1--			
Compound Name	:		
Start Time	:14.05min		
End Time	:14.79min		
Acq. Mode	:Q3 Scan		
Event Time	:0.300sec		
Scan Speed	:2000		
Start m/z	:45.00		
End m/z	:600.00		
Q1 Resolution	: -		
Q3 Resolution	: -		
--Group 12 - Event 2--			
Compound Name	:Cysteic acid-3TMS		
Start Time	:14.05min		
End Time	:14.79min		
Acq. Mode	:MRM		
Event Time	:0.034sec		
Q1 Resolution	:Unit		
Q3 Resolution	:Low		
Ch1-m/z	:(Precursor)268.00	(Product)73.00	(CE)21.00V
Ch2-m/z	:(Precursor)268.00	(Product)106.10	(CE)12.00V
--Group 12 - Event 3--			
Compound Name	:Glutamine-4TMS		
Start Time	:14.05min		
End Time	:14.79min		
Acq. Mode	:MRM		
Event Time	:0.034sec		
Q1 Resolution	:Unit		
Q3 Resolution	:Low		
Ch1-m/z	:(Precursor)203.10	(Product)147.10	(CE)18.00V
Ch2-m/z	:(Precursor)227.10	(Product)73.00	(CE)21.00V
--Group 12 - Event 4--			
Compound Name	:Glutamine-3TMS		
Start Time	:14.05min		
End Time	:14.79min		
Acq. Mode	:MRM		
Event Time	:0.033sec		
Q1 Resolution	:Unit		
Q3 Resolution	:Low		
Ch1-m/z	:(Precursor)245.10	(Product)156.10	(CE)9.00V
Ch2-m/z	:(Precursor)245.10	(Product)73.00	(CE)21.00V
--Group 12 - Event 5--			
Compound Name	:Arginine-3TMS		
Start Time	:14.05min		
End Time	:14.79min		
Acq. Mode	:MRM		
Event Time	:0.033sec		
Q1 Resolution	:Unit		
Q3 Resolution	:Low		
Ch1-m/z	:(Precursor)157.20	(Product)73.00	(CE)18.00V
Ch2-m/z	:(Precursor)157.20	(Product)141.10	(CE)9.00V
--Group 12 - Event 6--			
Compound Name	:Isocitric acid-4TMS		
Start Time	:14.05min		
End Time	:14.79min		
Acq. Mode	:MRM		
Event Time	:0.033sec		
Q1 Resolution	:Unit		

Q3 Resolution	:Low		
Ch1-m/z	:(Precursor)245.10	(Product)73.00	(CE)21.00V
Ch2-m/z	:(Precursor)245.10	(Product)83.00	(CE)9.00V
--Group 12 - Event 7--			
Compound Name	:Citric acid-4TMS		
Start Time	:14.05min		
End Time	:14.79min		
Acq. Mode	:MRM		
Event Time	:0.033sec		
Q1 Resolution	:Unit		
Q3 Resolution	:Low		
Ch1-m/z	:(Precursor)347.10	(Product)147.10	(CE)24.00V
Ch2-m/z	:(Precursor)347.10	(Product)257.10	(CE)12.00V
--Group 12 - Event 8--			
Compound Name	:3-OMG_1		
Start Time	:14.05min		
End Time	:14.79min		
Acq. Mode	:MRM		
Event Time	:0.050sec		
Q1 Resolution	:Unit		
Q3 Resolution	:Low		
Ch1-m/z	:(Precursor)205.10	(Product)73.10	(CE)24.00V
Ch2-m/z	:(Precursor)261.15	(Product)73.10	(CE)24.00V
Ch3-m/z	:(Precursor)205.10	(Product)147.10	(CE)6.00V
--Group 12 - Event 9--			
Compound Name	:3-OMG_2		
Start Time	:14.05min		
End Time	:14.79min		
Acq. Mode	:MRM		
Event Time	:0.050sec		
Q1 Resolution	:Unit		
Q3 Resolution	:Low		
Ch1-m/z	:(Precursor)205.10	(Product)73.10	(CE)21.00V
Ch2-m/z	:(Precursor)205.10	(Product)147.10	(CE)6.00V
Ch3-m/z	:(Precursor)157.00	(Product)73.10	(CE)15.00V
--Group 13 - Event 1--			
Compound Name	:		
Start Time	:14.79min		
End Time	:15.47min		
Acq. Mode	:Q3 Scan		
Event Time	:0.300sec		
Scan Speed	:2000		
Start m/z	:45.00		
End m/z	:600.00		
Q1 Resolution	: -		
Q3 Resolution	: -		
--Group 13 - Event 2--			
Compound Name	:Arginine-3TMS		
Start Time	:14.79min		
End Time	:15.47min		
Acq. Mode	:MRM		
Event Time	:0.026sec		
Q1 Resolution	:Unit		
Q3 Resolution	:Low		
Ch1-m/z	:(Precursor)157.20	(Product)73.00	(CE)18.00V
Ch2-m/z	:(Precursor)157.20	(Product)141.10	(CE)9.00V
--Group 13 - Event 3--			
Compound Name	:Isocitric acid-4TMS		
Start Time	:14.79min		
End Time	:15.47min		
Acq. Mode	:MRM		
Event Time	:0.025sec		
Q1 Resolution	:Unit		
Q3 Resolution	:Low		
Ch1-m/z	:(Precursor)245.10	(Product)73.00	(CE)21.00V
Ch2-m/z	:(Precursor)245.10	(Product)83.00	(CE)9.00V
--Group 13 - Event 4--			
Compound Name	:Citric acid-4TMS		
Start Time	:14.79min		
End Time	:15.47min		
Acq. Mode	:MRM		
Event Time	:0.025sec		
Q1 Resolution	:Unit		
Q3 Resolution	:Low		
Ch1-m/z	:(Precursor)347.10	(Product)147.10	(CE)24.00V
Ch2-m/z	:(Precursor)347.10	(Product)257.10	(CE)12.00V
--Group 13 - Event 5--			
Compound Name	:3-OMG_1		
Start Time	:14.79min		
End Time	:15.47min		
Acq. Mode	:MRM		

Event Time	:0.037sec		
Q1 Resolution	:Unit		
Q3 Resolution	:Low		
Ch1-m/z	:(Precursor)205.10	(Product)73.10	(CE)24.00V
Ch2-m/z	:(Precursor)261.15	(Product)73.10	(CE)24.00V
Ch3-m/z	:(Precursor)205.10	(Product)147.10	(CE)6.00V
--Group 13 - Event 6--			
Compound Name	:3-OMG_2		
Start Time	:14.79min		
End Time	:15.47min		
Acq. Mode	:MRM		
Event Time	:0.037sec		
Q1 Resolution	:Unit		
Q3 Resolution	:Low		
Ch1-m/z	:(Precursor)205.10	(Product)73.10	(CE)21.00V
Ch2-m/z	:(Precursor)205.10	(Product)147.10	(CE)6.00V
Ch3-m/z	:(Precursor)157.00	(Product)73.10	(CE)15.00V
--Group 13 - Event 7--			
Compound Name	:Fructose-meto-5TMS(1)		
Start Time	:14.79min		
End Time	:15.47min		
Acq. Mode	:MRM		
Event Time	:0.025sec		
Q1 Resolution	:Unit		
Q3 Resolution	:Low		
Ch1-m/z	:(Precursor)307.00	(Product)73.00	(CE)24.00V
Ch2-m/z	:(Precursor)307.00	(Product)217.10	(CE)6.00V
--Group 13 - Event 8--			
Compound Name	:Fructose-meto-5TMS(2)		
Start Time	:14.79min		
End Time	:15.47min		
Acq. Mode	:MRM		
Event Time	:0.025sec		
Q1 Resolution	:Unit		
Q3 Resolution	:Low		
Ch1-m/z	:(Precursor)307.10	(Product)217.20	(CE)6.00V
Ch2-m/z	:(Precursor)307.10	(Product)73.00	(CE)27.00V
--Group 13 - Event 9--			
Compound Name	:Mannose-meto-5TMS(1)		
Start Time	:14.79min		
End Time	:15.47min		
Acq. Mode	:MRM		
Event Time	:0.025sec		
Q1 Resolution	:Unit		
Q3 Resolution	:Low		
Ch1-m/z	:(Precursor)319.00	(Product)73.00	(CE)18.00V
Ch2-m/z	:(Precursor)205.10	(Product)147.10	(CE)9.00V
--Group 13 - Event 10--			
Compound Name	:Glucose-meto-5TMS(1)		
Start Time	:14.79min		
End Time	:15.47min		
Acq. Mode	:MRM		
Event Time	:0.025sec		
Q1 Resolution	:Unit		
Q3 Resolution	:Low		
Ch1-m/z	:(Precursor)319.10	(Product)73.00	(CE)24.00V
Ch2-m/z	:(Precursor)319.10	(Product)129.10	(CE)12.00V
--Group 13 - Event 11--			
Compound Name	:Glucose 13C6-meto-5TMS(1)		
Start Time	:14.79min		
End Time	:15.47min		
Acq. Mode	:MRM		
Event Time	:0.025sec		
Q1 Resolution	:Unit		
Q3 Resolution	:Low		
Ch1-m/z	:(Precursor)323.20	(Product)73.10	(CE)27.00V
Ch2-m/z	:(Precursor)323.20	(Product)132.20	(CE)9.00V
--Group 13 - Event 12--			
Compound Name	:Mannose-meto-5TMS(2)		
Start Time	:14.79min		
End Time	:15.47min		
Acq. Mode	:MRM		
Event Time	:0.025sec		
Q1 Resolution	:Unit		
Q3 Resolution	:Low		
Ch1-m/z	:(Precursor)319.00	(Product)73.00	(CE)27.00V
Ch2-m/z	:(Precursor)205.10	(Product)147.10	(CE)6.00V
--Group 14 - Event 1--			
Compound Name	:		
Start Time	:15.47min		
End Time	:15.75min		

Acq. Mode	:Q3 Scan		
Event Time	:0.300sec		
Scan Speed	:2000		
Start m/z	:45.00		
End m/z	:600.00		
Q1 Resolution	: -		
Q3 Resolution	: -		
--Group 14 - Event 2--			
Compound Name	:Fructose-meto-5TMS(1)		
Start Time	:15.47min		
End Time	:15.75min		
Acq. Mode	:MRM		
Event Time	:0.034sec		
Q1 Resolution	:Unit		
Q3 Resolution	:Low		
Ch1-m/z	:(Precursor)307.00	(Product)73.00	(CE)24.00V
Ch2-m/z	:(Precursor)307.00	(Product)217.10	(CE)6.00V
--Group 14 - Event 3--			
Compound Name	:Fructose-meto-5TMS(2)		
Start Time	:15.47min		
End Time	:15.75min		
Acq. Mode	:MRM		
Event Time	:0.034sec		
Q1 Resolution	:Unit		
Q3 Resolution	:Low		
Ch1-m/z	:(Precursor)307.10	(Product)217.20	(CE)6.00V
Ch2-m/z	:(Precursor)307.10	(Product)73.00	(CE)27.00V
--Group 14 - Event 4--			
Compound Name	:Mannose-meto-5TMS(1)		
Start Time	:15.47min		
End Time	:15.75min		
Acq. Mode	:MRM		
Event Time	:0.034sec		
Q1 Resolution	:Unit		
Q3 Resolution	:Low		
Ch1-m/z	:(Precursor)319.00	(Product)73.00	(CE)18.00V
Ch2-m/z	:(Precursor)205.10	(Product)147.10	(CE)9.00V
--Group 14 - Event 5--			
Compound Name	:Glucose-meto-5TMS(1)		
Start Time	:15.47min		
End Time	:15.75min		
Acq. Mode	:MRM		
Event Time	:0.033sec		
Q1 Resolution	:Unit		
Q3 Resolution	:Low		
Ch1-m/z	:(Precursor)319.10	(Product)73.00	(CE)24.00V
Ch2-m/z	:(Precursor)319.10	(Product)129.10	(CE)12.00V
--Group 14 - Event 6--			
Compound Name	:Glucose 13C6-meto-5TMS(1)		
Start Time	:15.47min		
End Time	:15.75min		
Acq. Mode	:MRM		
Event Time	:0.033sec		
Q1 Resolution	:Unit		
Q3 Resolution	:Low		
Ch1-m/z	:(Precursor)323.20	(Product)73.10	(CE)27.00V
Ch2-m/z	:(Precursor)323.20	(Product)132.20	(CE)9.00V
--Group 14 - Event 7--			
Compound Name	:Mannose-meto-5TMS(2)		
Start Time	:15.47min		
End Time	:15.75min		
Acq. Mode	:MRM		
Event Time	:0.033sec		
Q1 Resolution	:Unit		
Q3 Resolution	:Low		
Ch1-m/z	:(Precursor)319.00	(Product)73.00	(CE)27.00V
Ch2-m/z	:(Precursor)205.10	(Product)147.10	(CE)6.00V
--Group 14 - Event 8--			
Compound Name	:Glucose 13C6-meto-5TMS(2)		
Start Time	:15.47min		
End Time	:15.75min		
Acq. Mode	:MRM		
Event Time	:0.033sec		
Q1 Resolution	:Unit		
Q3 Resolution	:Low		
Ch1-m/z	:(Precursor)323.20	(Product)73.10	(CE)21.00V
Ch2-m/z	:(Precursor)323.20	(Product)132.10	(CE)9.00V
--Group 14 - Event 9--			
Compound Name	:Lysine-4TMS		
Start Time	:15.47min		
End Time	:15.75min		

Acq. Mode	:MRM		
Event Time	:0.033sec		
Q1 Resolution	:Unit		
Q3 Resolution	:Low		
Ch1-m/z	:(Precursor)317.20	(Product)156.20	(CE)9.00V
Ch2-m/z	:(Precursor)317.20	(Product)73.00	(CE)24.00V
--Group 14 - Event 10--			
Compound Name	:Histidine-3TMS		
Start Time	:15.47min		
End Time	:15.75min		
Acq. Mode	:MRM		
Event Time	:0.033sec		
Q1 Resolution	:Unit		
Q3 Resolution	:Low		
Ch1-m/z	:(Precursor)154.10	(Product)73.00	(CE)18.00V
Ch2-m/z	:(Precursor)254.20	(Product)155.20	(CE)15.00V
--Group 15 - Event 1--			
Compound Name	:		
Start Time	:15.75min		
End Time	:16.05min		
Acq. Mode	:Q3 Scan		
Event Time	:0.300sec		
Scan Speed	:2000		
Start m/z	:45.00		
End m/z	:600.00		
Q1 Resolution	: -		
Q3 Resolution	: -		
--Group 15 - Event 2--			
Compound Name	:Mannose-meto-5TMS(1)		
Start Time	:15.75min		
End Time	:16.05min		
Acq. Mode	:MRM		
Event Time	:0.028sec		
Q1 Resolution	:Unit		
Q3 Resolution	:Low		
Ch1-m/z	:(Precursor)319.00	(Product)73.00	(CE)18.00V
Ch2-m/z	:(Precursor)205.10	(Product)147.10	(CE)9.00V
--Group 15 - Event 3--			
Compound Name	:Glucose-meto-5TMS(1)		
Start Time	:15.75min		
End Time	:16.05min		
Acq. Mode	:MRM		
Event Time	:0.028sec		
Q1 Resolution	:Unit		
Q3 Resolution	:Low		
Ch1-m/z	:(Precursor)319.10	(Product)73.00	(CE)24.00V
Ch2-m/z	:(Precursor)319.10	(Product)129.10	(CE)12.00V
--Group 15 - Event 4--			
Compound Name	:Glucose 13C6-meto-5TMS(1)		
Start Time	:15.75min		
End Time	:16.05min		
Acq. Mode	:MRM		
Event Time	:0.028sec		
Q1 Resolution	:Unit		
Q3 Resolution	:Low		
Ch1-m/z	:(Precursor)323.20	(Product)73.10	(CE)27.00V
Ch2-m/z	:(Precursor)323.20	(Product)132.20	(CE)9.00V
--Group 15 - Event 5--			
Compound Name	:Mannose-meto-5TMS(2)		
Start Time	:15.75min		
End Time	:16.05min		
Acq. Mode	:MRM		
Event Time	:0.027sec		
Q1 Resolution	:Unit		
Q3 Resolution	:Low		
Ch1-m/z	:(Precursor)319.00	(Product)73.00	(CE)27.00V
Ch2-m/z	:(Precursor)205.10	(Product)147.10	(CE)6.00V
--Group 15 - Event 6--			
Compound Name	:Glucose 13C6-meto-5TMS(2)		
Start Time	:15.75min		
End Time	:16.05min		
Acq. Mode	:MRM		
Event Time	:0.027sec		
Q1 Resolution	:Unit		
Q3 Resolution	:Low		
Ch1-m/z	:(Precursor)323.20	(Product)73.10	(CE)21.00V
Ch2-m/z	:(Precursor)323.20	(Product)132.10	(CE)9.00V
--Group 15 - Event 7--			
Compound Name	:Lysine-4TMS		
Start Time	:15.75min		
End Time	:16.05min		

Acq. Mode	:MRM		
Event Time	:0.027sec		
Q1 Resolution	:Unit		
Q3 Resolution	:Low		
Ch1-m/z	:(Precursor)317.20	(Product)156.20	(CE)9.00V
Ch2-m/z	:(Precursor)317.20	(Product)73.00	(CE)24.00V
--Group 15 - Event 8--			
Compound Name	:Histidine-3TMS		
Start Time	:15.75min		
End Time	:16.05min		
Acq. Mode	:MRM		
Event Time	:0.027sec		
Q1 Resolution	:Unit		
Q3 Resolution	:Low		
Ch1-m/z	:(Precursor)154.10	(Product)73.00	(CE)18.00V
Ch2-m/z	:(Precursor)254.20	(Product)155.20	(CE)15.00V
--Group 15 - Event 9--			
Compound Name	:Tyrosine-3TMS		
Start Time	:15.75min		
End Time	:16.05min		
Acq. Mode	:MRM		
Event Time	:0.027sec		
Q1 Resolution	:Unit		
Q3 Resolution	:Low		
Ch1-m/z	:(Precursor)218.10	(Product)73.00	(CE)18.00V
Ch2-m/z	:(Precursor)218.10	(Product)100.10	(CE)9.00V
--Group 15 - Event 10--			
Compound Name	:Galacturonic acid-meto-5TMS(1)		
Start Time	:15.75min		
End Time	:16.05min		
Acq. Mode	:MRM		
Event Time	:0.027sec		
Q1 Resolution	:Unit		
Q3 Resolution	:Low		
Ch1-m/z	:(Precursor)333.10	(Product)73.00	(CE)27.00V
Ch2-m/z	:(Precursor)333.10	(Product)143.10	(CE)15.00V
--Group 15 - Event 11--			
Compound Name	:Galactitol-6TMS		
Start Time	:15.75min		
End Time	:16.05min		
Acq. Mode	:MRM		
Event Time	:0.027sec		
Q1 Resolution	:Unit		
Q3 Resolution	:Low		
Ch1-m/z	:(Precursor)319.20	(Product)73.00	(CE)24.00V
Ch2-m/z	:(Precursor)307.20	(Product)73.00	(CE)24.00V
--Group 15 - Event 12--			
Compound Name	:Galacturonic acid-meto-5TMS(2)		
Start Time	:15.75min		
End Time	:16.05min		
Acq. Mode	:MRM		
Event Time	:0.027sec		
Q1 Resolution	:Unit		
Q3 Resolution	:Low		
Ch1-m/z	:(Precursor)333.10	(Product)73.00	(CE)24.00V
Ch2-m/z	:(Precursor)333.10	(Product)143.10	(CE)15.00V
--Group 16 - Event 1--			
Compound Name	:		
Start Time	:16.05min		
End Time	:16.71min		
Acq. Mode	:Q3 Scan		
Event Time	:0.300sec		
Scan Speed	:2000		
Start m/z	:45.00		
End m/z	:600.00		
Q1 Resolution	: -		
Q3 Resolution	: -		
--Group 16 - Event 2--			
Compound Name	:Glucose 13C6-meto-5TMS(2)		
Start Time	:16.05min		
End Time	:16.71min		
Acq. Mode	:MRM		
Event Time	:0.034sec		
Q1 Resolution	:Unit		
Q3 Resolution	:Low		
Ch1-m/z	:(Precursor)323.20	(Product)73.10	(CE)21.00V
Ch2-m/z	:(Precursor)323.20	(Product)132.10	(CE)9.00V
--Group 16 - Event 3--			
Compound Name	:Lysine-4TMS		
Start Time	:16.05min		
End Time	:16.71min		

Acq. Mode	:MRM		
Event Time	:0.034sec		
Q1 Resolution	:Unit		
Q3 Resolution	:Low		
Ch1-m/z	:(Precursor)317.20	(Product)156.20	(CE)9.00V
Ch2-m/z	:(Precursor)317.20	(Product)73.00	(CE)24.00V
--Group 16 - Event 4--			
Compound Name	:Histidine-3TMS		
Start Time	:16.05min		
End Time	:16.71min		
Acq. Mode	:MRM		
Event Time	:0.034sec		
Q1 Resolution	:Unit		
Q3 Resolution	:Low		
Ch1-m/z	:(Precursor)154.10	(Product)73.00	(CE)18.00V
Ch2-m/z	:(Precursor)254.20	(Product)155.20	(CE)15.00V
--Group 16 - Event 5--			
Compound Name	:Tyrosine-3TMS		
Start Time	:16.05min		
End Time	:16.71min		
Acq. Mode	:MRM		
Event Time	:0.033sec		
Q1 Resolution	:Unit		
Q3 Resolution	:Low		
Ch1-m/z	:(Precursor)218.10	(Product)73.00	(CE)18.00V
Ch2-m/z	:(Precursor)218.10	(Product)100.10	(CE)9.00V
--Group 16 - Event 6--			
Compound Name	:Galacturonic acid-meto-5TMS(1)		
Start Time	:16.05min		
End Time	:16.71min		
Acq. Mode	:MRM		
Event Time	:0.033sec		
Q1 Resolution	:Unit		
Q3 Resolution	:Low		
Ch1-m/z	:(Precursor)333.10	(Product)73.00	(CE)27.00V
Ch2-m/z	:(Precursor)333.10	(Product)143.10	(CE)15.00V
--Group 16 - Event 7--			
Compound Name	:Galactitol-6TMS		
Start Time	:16.05min		
End Time	:16.71min		
Acq. Mode	:MRM		
Event Time	:0.033sec		
Q1 Resolution	:Unit		
Q3 Resolution	:Low		
Ch1-m/z	:(Precursor)319.20	(Product)73.00	(CE)24.00V
Ch2-m/z	:(Precursor)307.20	(Product)73.00	(CE)24.00V
--Group 16 - Event 8--			
Compound Name	:Galacturonic acid-meto-5TMS(2)		
Start Time	:16.05min		
End Time	:16.71min		
Acq. Mode	:MRM		
Event Time	:0.033sec		
Q1 Resolution	:Unit		
Q3 Resolution	:Low		
Ch1-m/z	:(Precursor)333.10	(Product)73.00	(CE)24.00V
Ch2-m/z	:(Precursor)333.10	(Product)143.10	(CE)15.00V
--Group 16 - Event 9--			
Compound Name	:Pantothenic acid-3TMS		
Start Time	:16.05min		
End Time	:16.71min		
Acq. Mode	:MRM		
Event Time	:0.033sec		
Q1 Resolution	:Unit		
Q3 Resolution	:Low		
Ch1-m/z	:(Precursor)291.20	(Product)201.10	(CE)9.00V
Ch2-m/z	:(Precursor)291.20	(Product)159.10	(CE)15.00V
--Group 16 - Event 10--			
Compound Name	:Inositol-6TMS(1)		
Start Time	:16.05min		
End Time	:16.71min		
Acq. Mode	:MRM		
Event Time	:0.033sec		
Q1 Resolution	:Unit		
Q3 Resolution	:Low		
Ch1-m/z	:(Precursor)305.10	(Product)73.00	(CE)21.00V
Ch2-m/z	:(Precursor)318.10	(Product)147.10	(CE)21.00V
--Group 17 - Event 1--			
Compound Name	:		
Start Time	:16.71min		
End Time	:18.24min		
Acq. Mode	:Q3 Scan		

Event Time	:0.300sec		
Scan Speed	:2000		
Start m/z	:45.00		
End m/z	:600.00		
Q1 Resolution	: -		
Q3 Resolution	: -		
--Group 17 - Event 2--			
Compound Name	:Pantothenic acid-3TMS		
Start Time	:16.71min		
End Time	:18.24min		
Acq. Mode	:MRM		
Event Time	:0.075sec		
Q1 Resolution	:Unit		
Q3 Resolution	:Low		
Ch1-m/z	:(Precursor)291.20	(Product)201.10	(CE)9.00V
Ch2-m/z	:(Precursor)291.20	(Product)159.10	(CE)15.00V
--Group 17 - Event 3--			
Compound Name	:Inositol-6TMS(1) / Inositol-6TMS(2)		
Start Time	:16.71min		
End Time	:18.24min		
Acq. Mode	:MRM		
Event Time	:0.075sec		
Q1 Resolution	:Unit		
Q3 Resolution	:Low		
Ch1-m/z	:(Precursor)305.10	(Product)73.00	(CE)21.00V
Ch2-m/z	:(Precursor)318.10	(Product)147.10	(CE)21.00V
--Group 17 - Event 4--			
Compound Name	:Palmitic acid-TMS		
Start Time	:16.71min		
End Time	:18.24min		
Acq. Mode	:MRM		
Event Time	:0.075sec		
Q1 Resolution	:Unit		
Q3 Resolution	:Low		
Ch1-m/z	:(Precursor)313.30	(Product)75.10	(CE)21.00V
Ch2-m/z	:(Precursor)145.10	(Product)129.10	(CE)6.00V
--Group 17 - Event 5--			
Compound Name	:Glucaric acid-6TMS		
Start Time	:16.71min		
End Time	:18.24min		
Acq. Mode	:MRM		
Event Time	:0.075sec		
Q1 Resolution	:Unit		
Q3 Resolution	:Low		
Ch1-m/z	:(Precursor)333.10	(Product)73.00	(CE)24.00V
Ch2-m/z	:(Precursor)333.10	(Product)143.10	(CE)15.00V
--Group 18 - Event 1--			
Compound Name	:		
Start Time	:18.24min		
End Time	:20.52min		
Acq. Mode	:Q3 Scan		
Event Time	:0.300sec		
Scan Speed	:2000		
Start m/z	:45.00		
End m/z	:600.00		
Q1 Resolution	: -		
Q3 Resolution	: -		
--Group 18 - Event 2--			
Compound Name	:Stearic acid-TMS		
Start Time	:18.24min		
End Time	:20.52min		
Acq. Mode	:MRM		
Event Time	:0.300sec		
Q1 Resolution	:Unit		
Q3 Resolution	:Low		
Ch1-m/z	:(Precursor)145.10	(Product)129.10	(CE)6.00V
Ch2-m/z	:(Precursor)341.30	(Product)75.00	(CE)21.00V
--Group 19 - Event 1--			
Compound Name	:		
Start Time	:20.52min		
End Time	:29.89min		
Acq. Mode	:Q3 Scan		
Event Time	:0.300sec		
Scan Speed	:2000		
Start m/z	:45.00		
End m/z	:600.00		
Q1 Resolution	: -		
Q3 Resolution	: -		
--Group 19 - Event 2--			
Compound Name	:Sucrose-8TMS		
Start Time	:20.52min		

End Time	:29.89min		
Acq. Mode	:MRM		
Event Time	:0.300sec		
Q1 Resolution	:Unit		
Q3 Resolution	:Low		
Ch1-m/z	:(Precursor)361.10	(Product)73.00	(CE)24.00V
Ch2-m/z	:(Precursor)361.10	(Product)169.10	(CE)12.00V

--Group 20 - Event 1--

Compound Name	:
Start Time	:29.89min
End Time	:40.00min
Acq. Mode	:Q3 Scan
Event Time	:0.300sec
Scan Speed	:2000
Start m/z	:45.00
End m/z	:600.00
Q1 Resolution	: -
Q3 Resolution	: -

Sample Inlet Unit :GC
Inlet Line :Line 2

[MS Program]
Use MS Program :OFF
