# Supplementary material for: Increased nutrient diversity can induce loss of microbial diversity through enhanced resource uptake
Source: Nat Ecol Evol. 2026 Jul 20;10(8):1449–63. doi: 10.1038/s41559-026-03111-4 (PMC13441979; doi:10.1038/s41559-026-03111-4)
Supplement: Supplementary file 2 [file 41559_2026_3111_MOESM1_ESM.pdf]

nature ecology & evolution

Supplementary information https://doi.org/10.1038/s41559-026-03111-4

# Increased nutrient diversity can induce loss of microbial diversity through enhanced resource uptake

In the format provided by the authors and unedited

# Supplementary Information

## Emergent loss of microbial biodiversity with increasing resource diversity

Or Shalev[1*], Xiaozhou Ye[1], Joachim Kilian[2], Mark Stahl[2], Christoph Ratzke[1*]

[1]Interfaculty Institute for Microbiology and Infection Medicine Tübingen (IMIT), Cluster of Excellence EXC 2124 "Controlling Microbes to Fight Infections" (CMFI), University of Tübingen, Calwerstrasse 7/1, 72076 Tübingen

[2]Zentrum für Molekularbiologie der Pflanzen (ZMBP), University of Tübingen, Auf der Morgenstelle 32, 72076 Tübingen

*Correspondence to: or.shalev@uni-tuebingen.de, christoph.ratzke@uni-tuebingen.de

## Table of Contents

***Supplementary Figures*** ....................................................................................... 4

**Supplementary Figure 1: Microbial diversity rarely increases with resource complexity, challenging the intuitive consumer-resource model.** ........................................................ 4

**Supplementary Figure 2: Communities reach a steady state by day 10.** ................................... 6

**Supplementary Figure 3: Richness trends across resource number are robust to contamination and low-abundance taxa, and strongly correlate with the Shannon index.** ............................... 7

**Supplementary Figure 4: Strain relative abundances across resource complexity in the 14 communities. Supplementary results for Fig. 2B.** ...................................................... 9

**Supplementary Figure 5: $OD_{600}$ at carrying capacity for 14 strains grown on individual, 8-resource, and 16-resource mixtures.** ........................................................................ 10

**Supplementary Fig. 7: Simulating microbial communities using interaction matrices derived from experimental data.** ........................................................................................ 12

**Supplementary Figure 8: Individual αself and αother values obtained via Bayesian regression, and their difference, plotted as a function of the number of resources.** ................................. 13

**Supplementary Figure 9: Interaction matrix directly derived from single-species and pairwise growth data.** ................................................................................................ 14

**Supplementary Figure 10: Validation of steady state in gLV simulations.** ............................... 15

**Supplementary Figure 11: Shapley analysis under varying resource conditions.** ...................... 16

**Supplementary Figure 12: Our experimental results support the same conclusion as the simulations: strains with increasingly negative impact factor slopes drive biodiversity loss, while those with positive slopes have the opposite effect.** ............................................................ 18

**Supplementary Figure 13: Impact factor of all 14 strains across resource complexity.** ............. 19

**Supplementary Figure 14: Strain '79' dominance increases with resource complexity, reducing diversity.** ............................................................................................................ 20

**Supplementary Figure 16: Impact factor on the sentinel strain is more suppressive in mixed resources.** ........................................................................................................ 22

**Supplementary Figure 17: K-means clustering was used to classify strains into two types (highly and mildly influenced).** .................................................................................... 23

**Supplementary Figure 18: Impact factor on the sentinel strain is more suppressive in mixed resources for both highly and mildly influenced strains.** ....................................................... 24

Supplementary Figure 20: K-means clustering was used to classify strains into three types based on their interactions in individual resources. ........ 26

Supplementary Figure 21: Highly and mildly influenced strains are categorized into three interaction-type clusters based on their behavior in individual resources. ........ 27

Supplementary Figure 23: The relative abundance of highly influenced strains increases with resource complexity across environments. ........ 30

Supplementary Figure 24: Highly influenced strains show greater metabolic uptake in resource mixtures than in their supernatant mixtures for specific metabolites, with this effect intensifying as resource complexity increases. ........ 32

Supplementary Figure 25: Highly influenced strains show greater metabolic uptake in resource mixtures than in their supernatant mixtures, for both supplied and not supplied resources, with this effect intensifying as resource complexity increases. ........ 33

Supplementary Figure 26: Media replenishment restored normal growth of the sentinel strain in most supernatants, ruling out toxicity as a primary competition driver. ........ 34

Supplementary Figure 27: Facilitation declines and suppression intensifies with increasing resource number, even within exclusively facilitative or suppressive subsets. ........ 35

Supplementary Figure 28: Highly influenced strain behavior can reduce microbial diversity as resource complexity increases in a consumer-resource model. ........ 36

Supplementary Figure 29: Highly influenced strain behavior reduces microbial diversity as resource complexity increases in a consumer-resource model, with highly influenced strains increasingly dominating. ........ 37

Supplementary Figure 30: Robustness of microbial diversity-resource complexity slopes across cross-feeding levels and correlation levels between niche width (generalism) and highly influenced behavior. ........ 39

Supplementary Figure 31: Robustness of microbial diversity-resource complexity slopes across varying levels of highly influenced strain sensitivity (γ). ........ 41

Supplementary Figure 32: Robustness of microbial diversity-resource complexity slopes across a transition from binary to continuous highly influenced and mildly influenced strain behavior distributions. ........ 42

Supplementary Figure 33: Robustness of microbial diversity-resource complexity slopes when resource complexity impacts highly influenced strain behavior by an alternative mechanism. . 43

Supplementary Figure 34: Robustness of microbial diversity-resource complexity slopes across communities with different numbers of initial species (nS). ........ 44

Supplementary Figure 35: 1:100 dilution of rich medium (NM) promotes synergistic growth by alleviating micronutrient limitations without supplying significant carbon. ........ 45

*Supplementary Tables ........ 46*

Supplementary Table 1: Sample sizes per condition for Fig. 3c. ........ 46

Supplementary Table 2: Statistical analysis for Fig. 4b,c. ........ 47

Supplementary Table 3: Resources used in this study. ........ 48

*Supplementary Text ........ 49*

Supplementary Text 1: Supplementary methods to the Consumer-Resource model and highly influenced strain extensions ........ 49

**Supplementary Text 2: Inferring interactions matrices from experimental data using the generalized Lotka-Volterra model....................................................................................... 56**

## Supplementary Figures

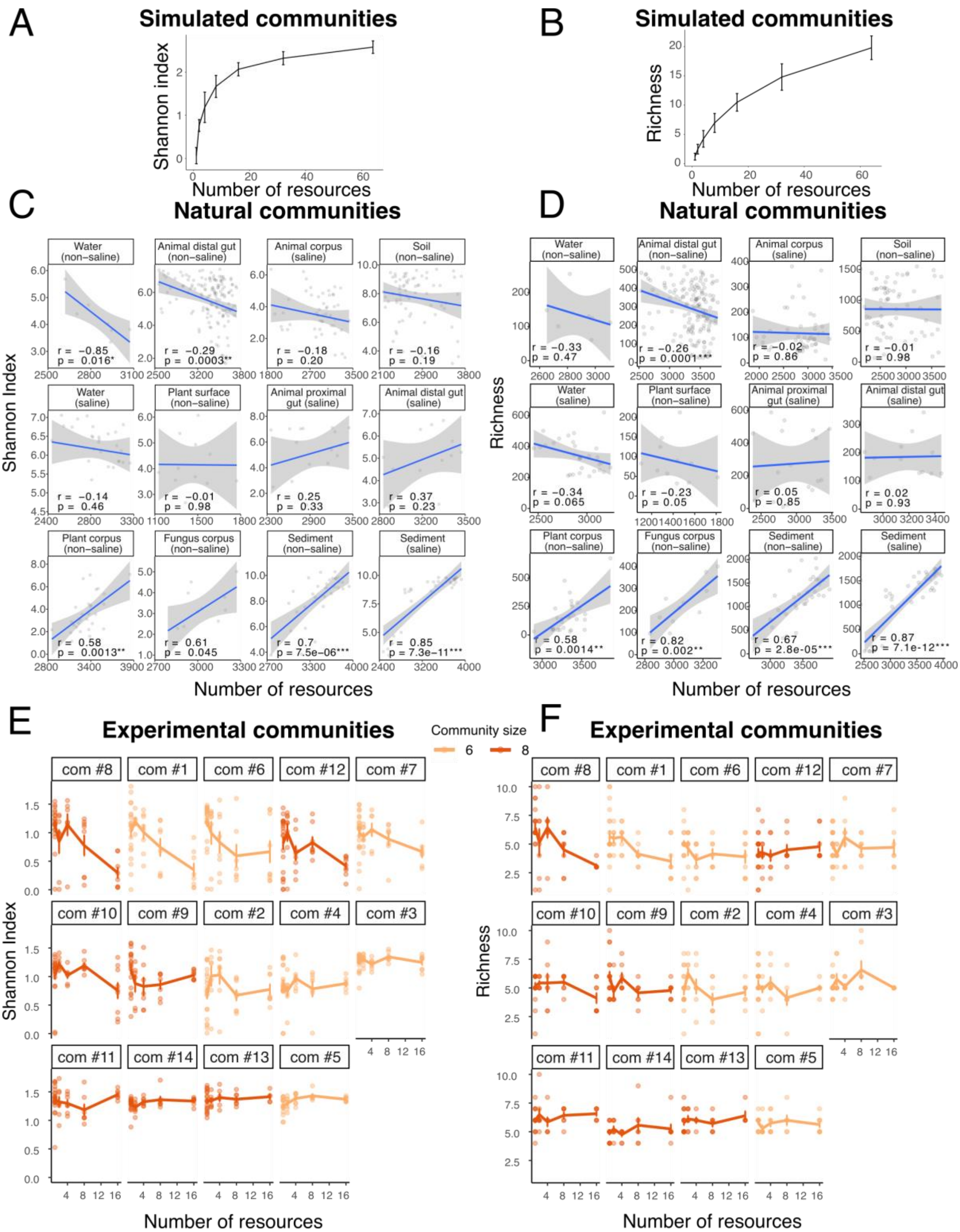


**Supplementary Figure 1: Microbial diversity rarely increases with resource complexity, challenging the intuitive consumer-resource model.** Supplementary results for Fig. 1. **A.** Simulations with the consumer-resource model predict an increase in alpha diversity with niche expansion, shown here using Shannon's diversity index. The trend line represents the average of 21

communities, with error bars indicating ± SD. **B.** Same as panel A but using richness instead of Shannon's diversity index. **C.** In natural communities, the relationship between microbial diversity and resource complexity varies, displaying both positive (consistent with the model) and negative slopes (contrary to it). Data are from the Earth Microbiome Project[1] ('EMP'); 16S metagenomics was used to assess diversity, while mass spectrometry determined the number of resources ('Methods'). Shown are 13 ecosystems at the highest binning level ('empo_4' in EMP nomenclature[1]). The slope of diversity vs. resource number ($\beta_1$) is derived from the linear model: diversity = $\beta_0 + \beta_1 \times$ number of resources + $\varepsilon$. The trend line represents the fitted linear model, with shading indicating the 95% CI. Pearson's correlation analysis (r: correlation coefficient, p: p-value) is shown at the bottom of each plot. Communities are ordered by their slope ($\beta_1$) using Shannon's diversity index (from negative to positive). **D.** Same as panel C but using richness instead of Shannon's diversity index. **E.** In the controlled experiment, microbial diversity responses to resource complexity varied across communities, showing both positive (as predicted by the consumer-resource model) and negative correlations (contrary to it). Data are from 16S sequencing after nine passages (one every 24 hours), followed by 24 h incubation. In total, alpha diversity was calculated for 701 successfully sequenced samples spanning all communities and carbon conditions. The trend line represents the average, with error bars indicating ± SEM. **F.** Same as panel E but using richness instead of Shannon's diversity index. Richness was calculated using a 0.05% relative-abundance threshold to exclude sequencing noise and extremely low-abundance taxa (species below this level were omitted). Results are robust to this cutoff choice, yielding similar qualitative trends when using no threshold (0%) or a higher threshold (1%; Supplementary Fig. 3). Raw (unadjusted) p-values are displayed in each panel. Statistical significance was assessed after Benjamini–Hochberg correction for multiple comparisons, and asterisks denote adjusted p-values (*p_adj < 0.05, **p_adj < 0.01, ***p_adj < 0.001).

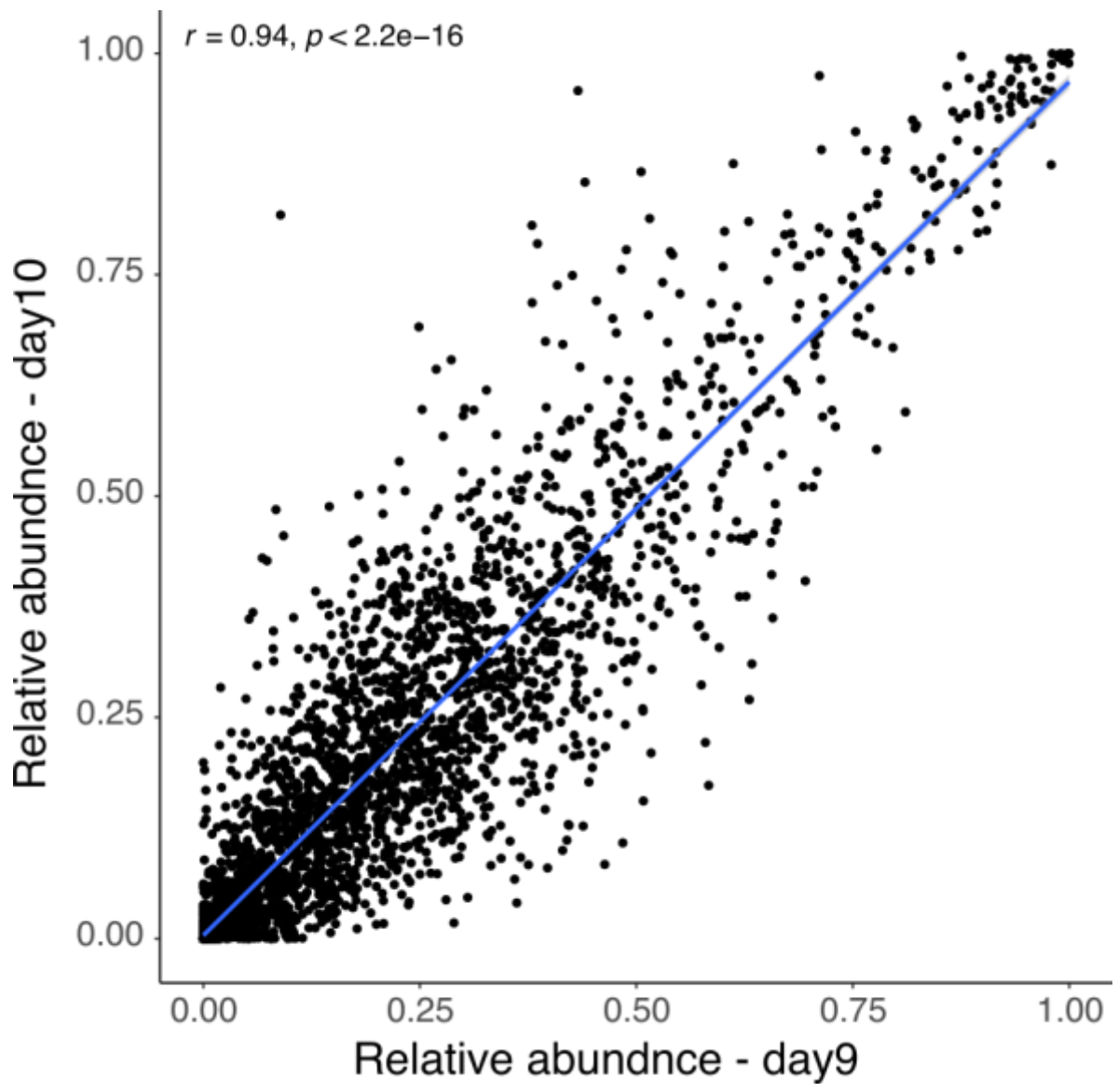


**Supplementary Figure 2: Communities reach a steady state by day 10.** Relative abundances of the same strains within the same sample (strain, community, condition, plate, and well) are shown for day 9 (after eight daily passages into fresh media, followed by 24 h incubation) and day 10 (after nine daily passages and 24 h incubation). Pearson's correlation analysis (r: correlation coefficient, p: p-value) is presented. Data from day 10 were used for all subsequent analyses in this experiment.

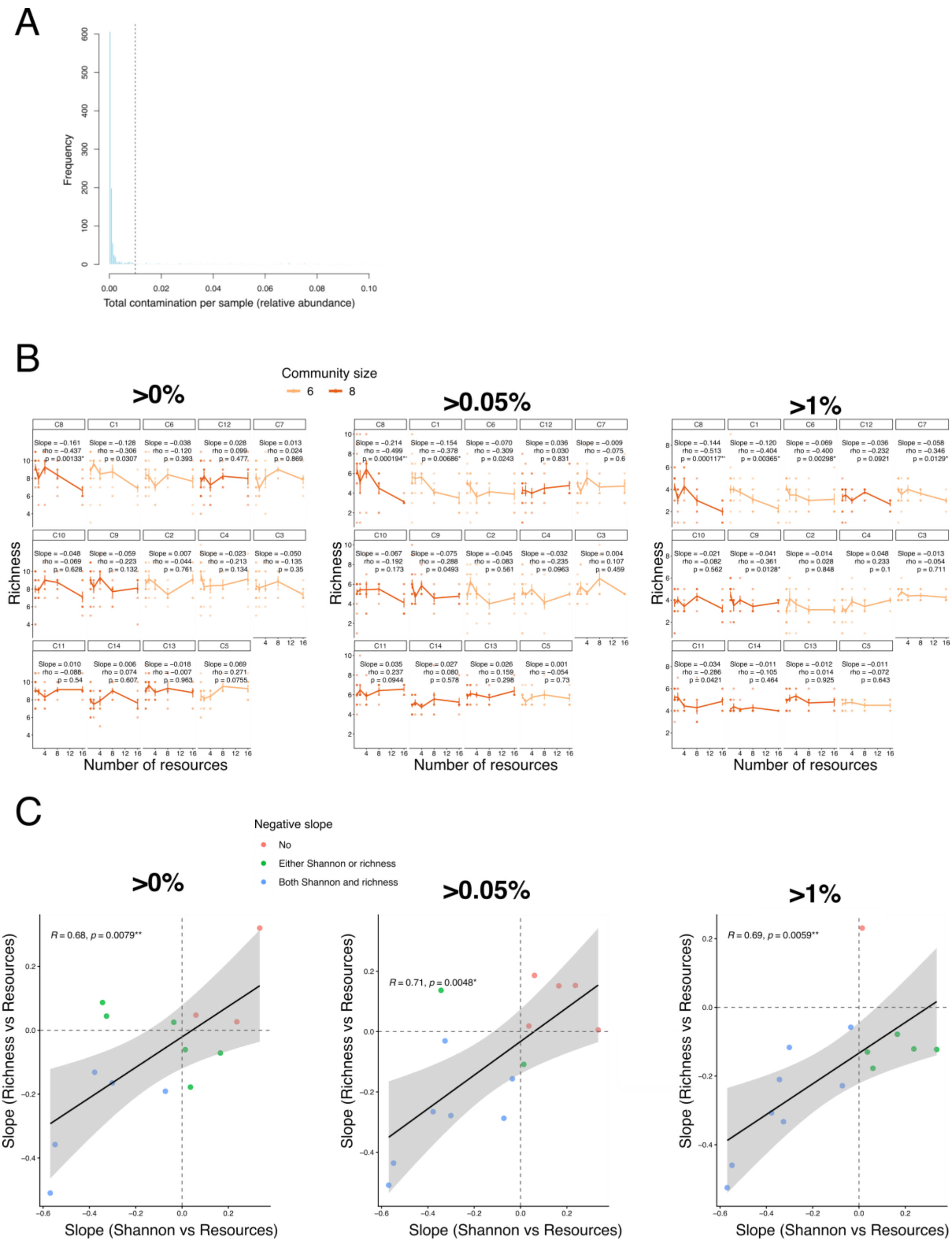


**Supplementary Figure 3: Richness trends across resource number are robust to contamination and low-abundance taxa, and strongly correlate with the Shannon index.** Supplementary results for Fig. 1E **A.** Representation of potential contaminations based on sequencing data (reads mapped to strains not inoculated in a given community). The vertical dashed line marks a relative abundance of

0.01 (1%). Most samples showed no detectable contaminants (0%), and when present, these were typically very low (~0.001, or 0.1%). All communities were assembled from the same 14-strain pool (used for 6- and 8-member communities), and reads were mapped against the full 14-strain reference database. Any additional taxa detected are therefore likely true low-level contaminants or artefacts from PCR, library preparation, sequencing, or mapping. **B.** Richness–resource number relationships were reanalyzed using different abundance thresholds (0%, 0.05%, and 1%), compared with the 0.05% threshold used in the main analysis (Fig. 1E). Results were highly consistent across thresholds. Slopes (from linear models) and corresponding Spearman correlations and p-values are indicated. **C.** Comparison of slopes from Shannon and richness indices (both versus resource number). The two metrics are strongly correlated across thresholds. Moreover, 7 of 14 communities show diversity reductions with increasing resource number for both indices, and only 2 show opposing trends (all values refer to the plot using the 0.05% relative abundance threshold, as in the main Fig. 1; 12 of 14 communities show the same overall direction). Raw (unadjusted) p-values are displayed in each panel. Statistical significance was assessed after Benjamini–Hochberg correction for multiple comparisons, and asterisks denote adjusted p-values (*p_adj < 0.05, **p_adj < 0.01, ***p_adj < 0.001).

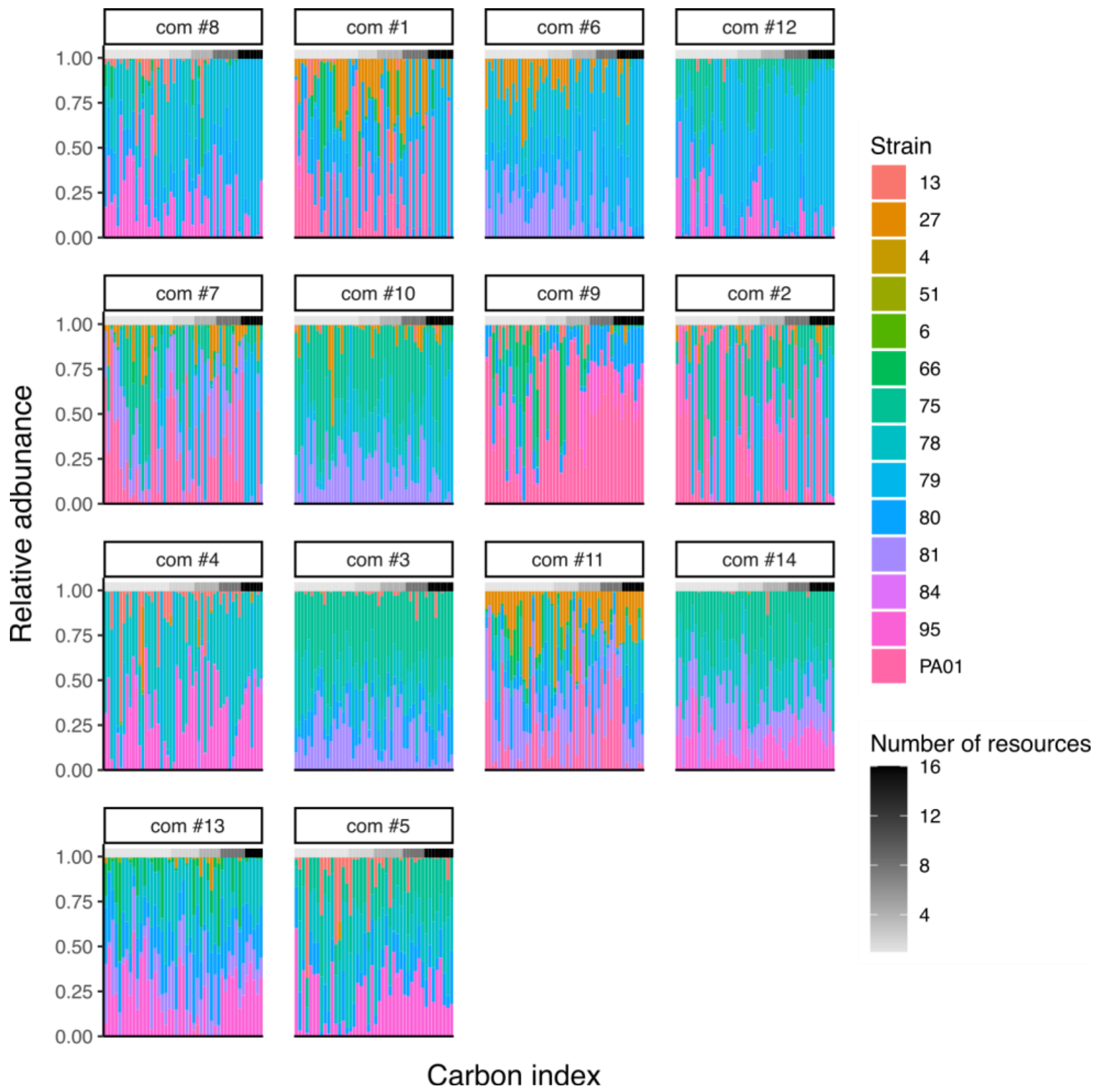


**Supplementary Figure 4: Strain relative abundances across resource complexity in the 14 communities. Supplementary results for Fig. 2B.**

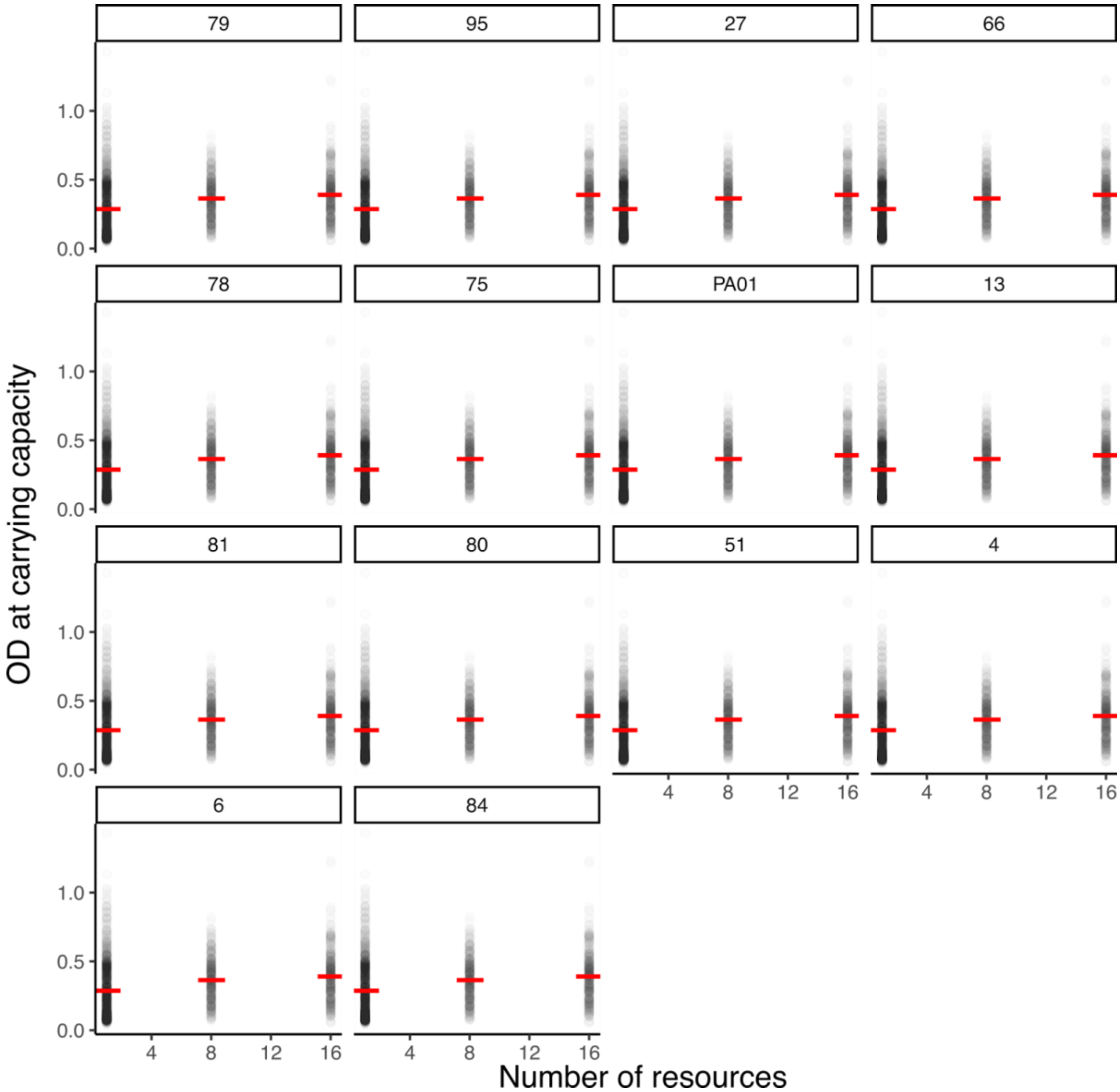


**Supplementary Figure 5: $OD_{600}$ at carrying capacity for 14 strains grown on individual, 8-resource, and 16-resource mixtures.** Supplementary results for Fig. 2B. $OD_{600}$ was measured after 72 h at carrying capacity (K) for each strain across all conditions used in the pairwise experiments. The red line indicates the mean. An increase in mean OD with the number of carbon sources is observed, consistent with a reduction in α self (self-regulation; α self = 1/K), as shown in Fig. 2B.

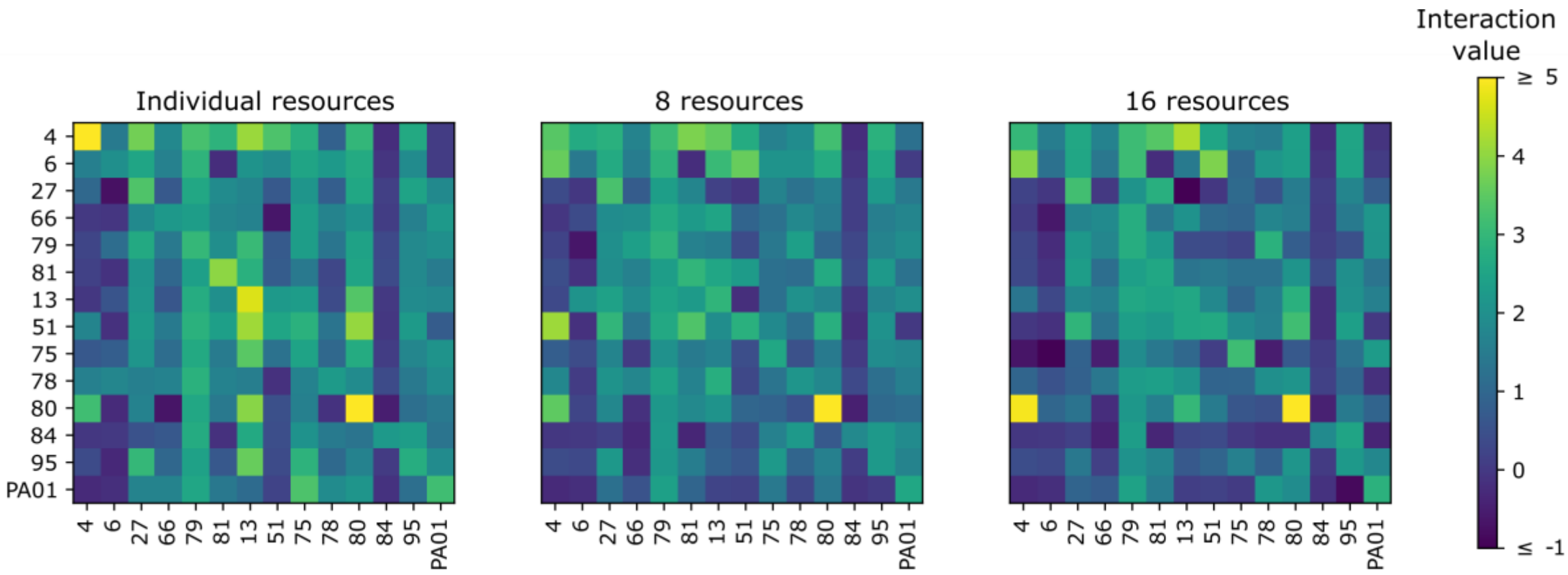


**Supplementary Figure 6: Interaction matrices for 1, 8, and 16 carbon sources, obtained by fitting the experimental data using Bayesian regression (see Supplementary Text 2).** As resource number increases, interaction coefficients decrease for both intra-species (diagonal) and inter-species interactions (off-diagonal). See also Fig. 2B and Supplementary Fig. 7.

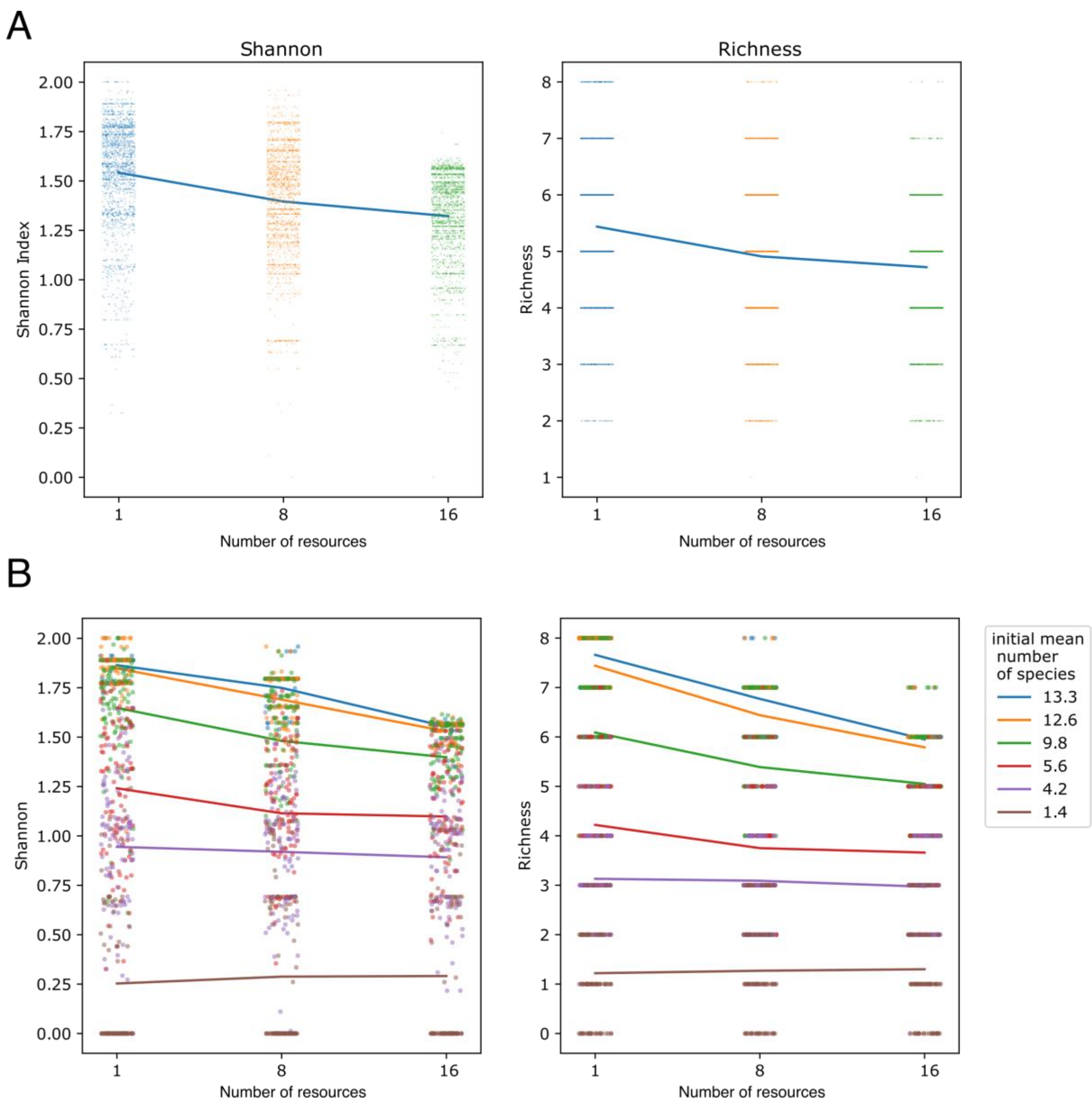


**Supplementary Fig. 7: Simulating microbial communities using interaction matrices derived from experimental data. A.** Simulating 2,000 communities with an average of 8.4 initial species (drawn from the 14 experimental strains) using the interaction matrices shown in Supplementary Fig. 4 reproduces the decline in entropy and richness observed in experimentally measured communities. **B.** Simulations as in panel A but using 100 communities for each fixed initial community size. A reduction in diversity emerges from a certain complexity threshold (approximately 4.2 species) and becomes more pronounced with increasing community size.

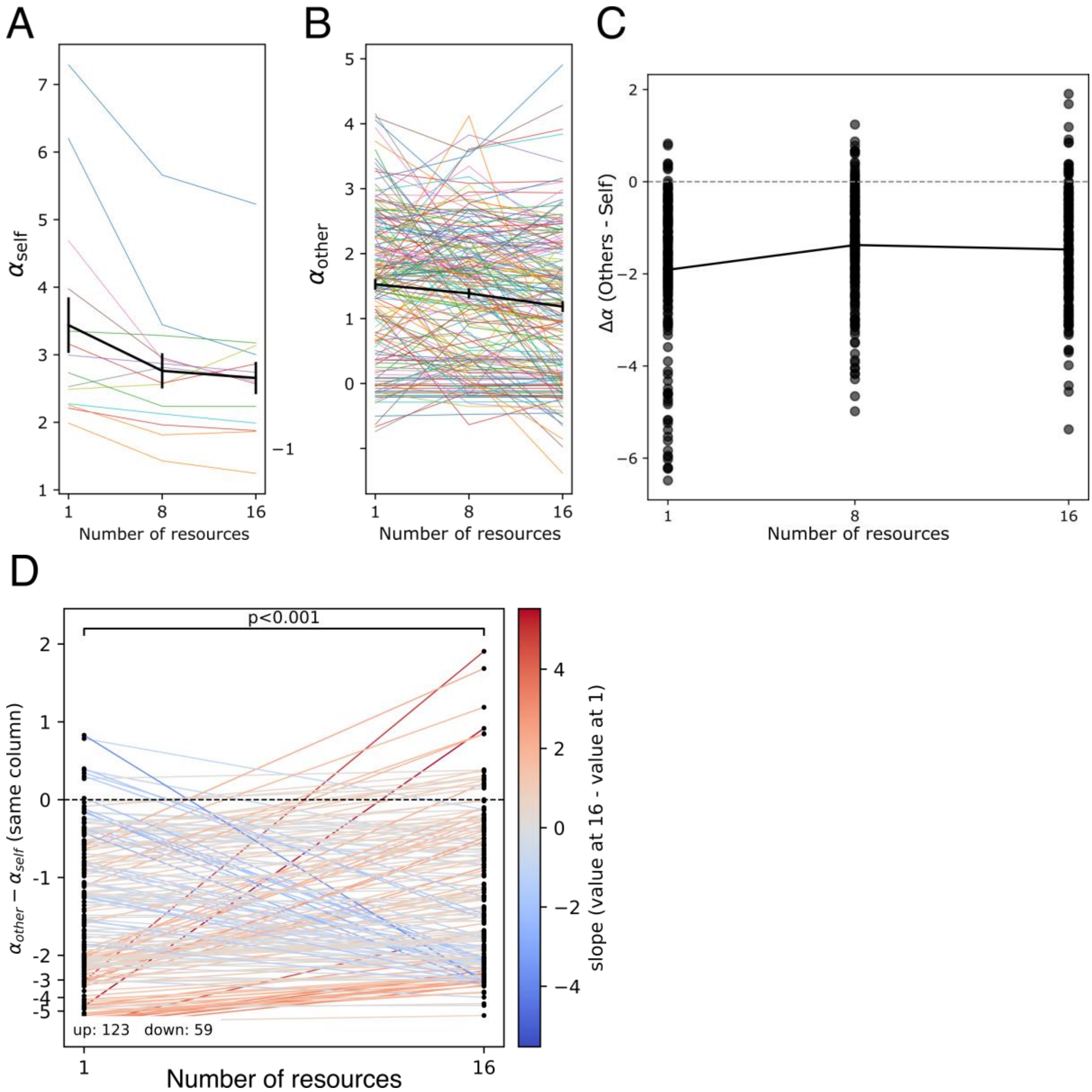


**Supplementary Figure 8: Individual αself and αother values obtained via Bayesian regression, and their difference, plotted as a function of the number of resources. A.** Nearly all $\alpha_{self}$ values decrease with increasing resource number. **B.** $\alpha_{other}$ shows a more complex pattern. Nonetheless, a decreasing trend is also observed on average for $\alpha_{other}$. Black line indicates the mean ± SEM. **C.** Supplement to the bottom panel of Fig. 2B. The system shifts on average toward a competitive exclusion regime at the pairwise level as resource complexity increases. The difference $\alpha_{other} - \alpha_{self}$ decreases with increasing resource number, and the proportion of pairwise interactions where $\alpha_{other} > \alpha_{self}$ (points above the dashed line) increases—conditions under which competitive exclusion occurs. **D.** Pairwise changes in $\alpha$ other $-\alpha$ self between low (1 resource) and high (16 resources) complexity conditions for individual strain pairs. Each line represents a pairwise interaction, colored by the slope (value at 16 minus value at 1). Most interactions show an increase (up: 123) rather than a decrease (down: 59), indicating a shift toward competitive exclusion with increasing resource complexity. Statistical significance was assessed using a paired two-sided Wilcoxon signed-rank test; p-value is indicated in the figure ($p < 0.001$).

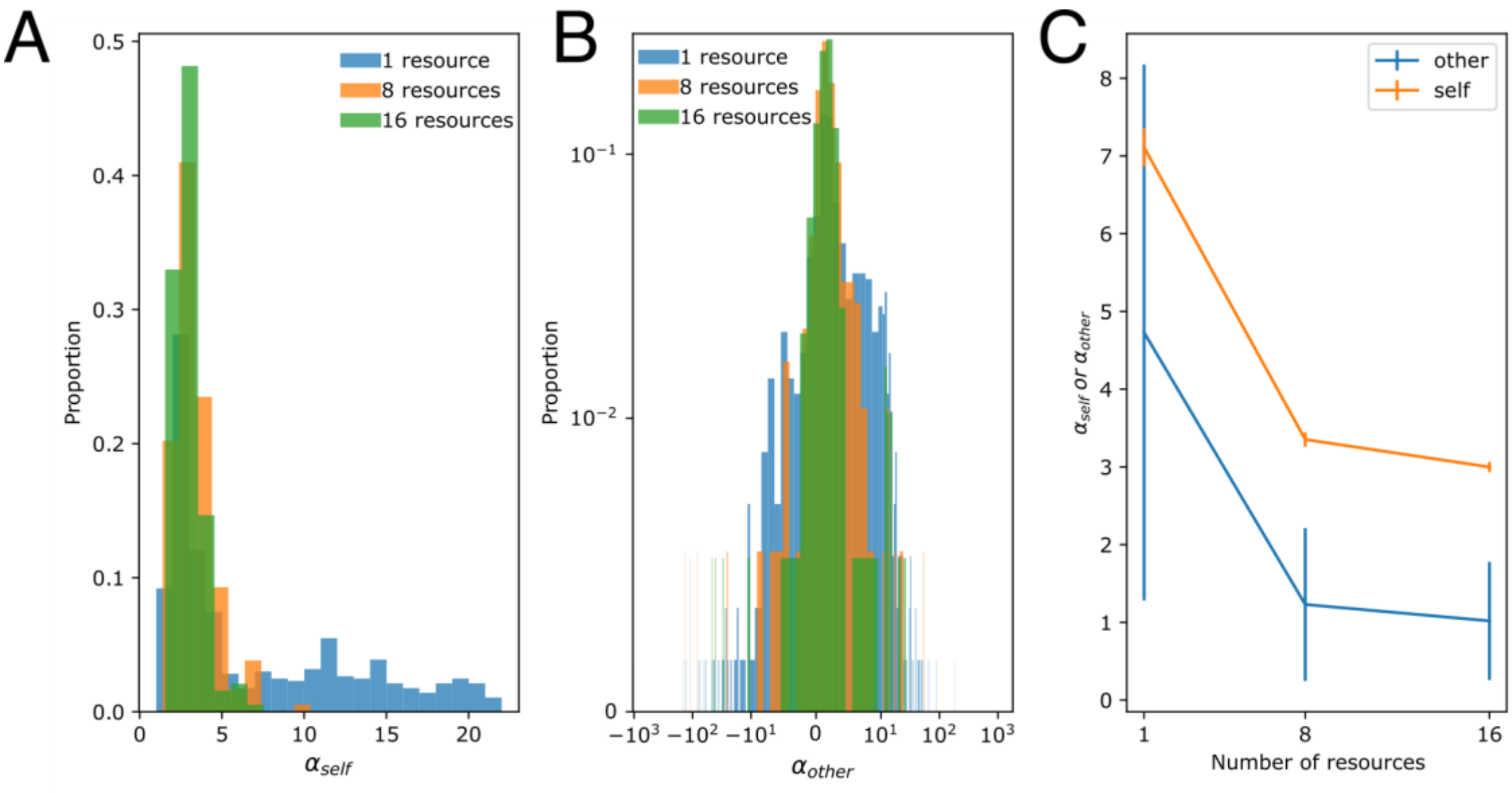


**Supplementary Figure 9: Interaction matrix directly derived from single-species and pairwise growth data.** $\alpha_{self}$ values were estimated as the inverse of carrying capacity, calculated from the final $OD_{600}$ after 72 hours of monoculture growth. $\alpha_{other}$ values were estimated by solving the two-species gLV model under steady-state assumptions: $\alpha_{12} = (1 - \alpha_{11}x_1)/x_2$ and $\alpha_{21} = (1 - \alpha_{22}x_2)/x_1$, using population densities ($x_1$, $x_2$) inferred from each strain's relative abundance multiplied by total community $OD_{600}$ (measured 24 h after five daily passages). To avoid division by near-zero values, strain pairs where either species had a relative abundance ≤ 0.001 were excluded. **A.** Distribution of $\alpha_{self}$ values for 1, 8, and 16 carbon sources. Low population densities in single-resource conditions occasionally produced large, noise-sensitive $\alpha_{self}$ estimates. **B.** This issue was more pronounced for $\alpha_{other}$ values, where small denominators led to extreme values and a broad distribution. **C.** Mean ± SEM of $\alpha_{self}$ and $\alpha_{other}$ across resource richness. The widespread in $\alpha_{other}$ values (panel B) inflated uncertainty in the means. To address this, we adopted a Bayesian regression framework, which allowed us to fit a unified model across all data points while reducing the influence of outliers. Despite the limitations of direct α estimation, the overall trends in panel C are consistent with those obtained using Bayesian regression (Main Fig. 2B).

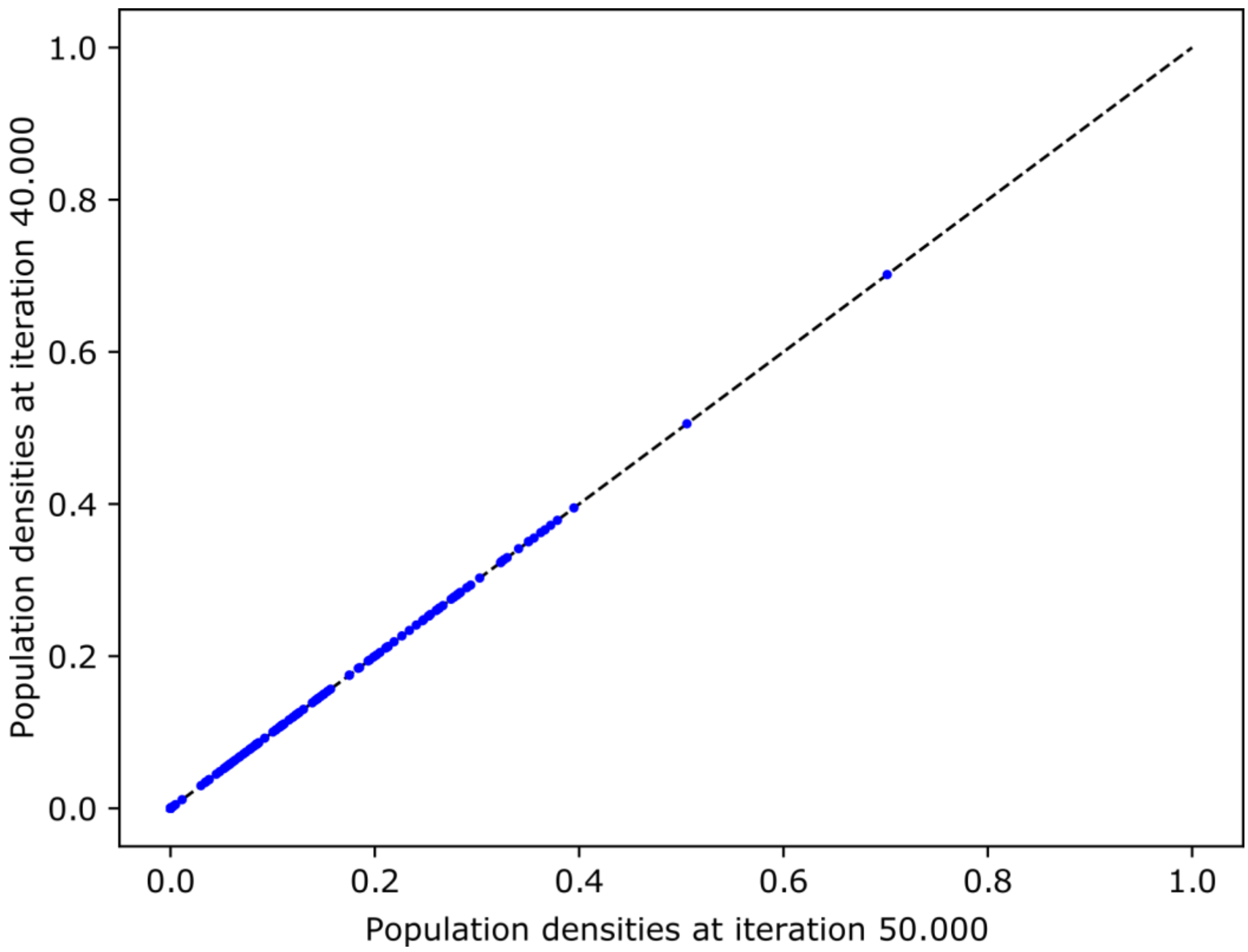


**Supplementary Figure 10: Validation of steady state in gLV simulations.** Related to Methods, chapter '*Estimating single-species Shapley contributions to biodiversity loss'*. Generalized Lotka–Volterra simulations were performed using the same parameters as in Fig. 2C and Supplementary Fig. 5, with interaction matrices from Supplementary Fig. 4. Each simulation run began with six species randomly selected from the full pool of 14. For each matrix, 30 simulations were run for 50,000 iterations. To confirm convergence, species densities at iteration 40,000 were plotted against their densities at iteration 50,000. The overlap between time points indicates that the system had reached steady state. This validation is important because the interaction matrices were derived from steady-state experimental data, but the per capita growth rates (k) could not be inferred and were set to 1. As a result, the model can reliably capture steady-state community structure, but not necessarily population dynamics, which are sensitive to the specific k values.

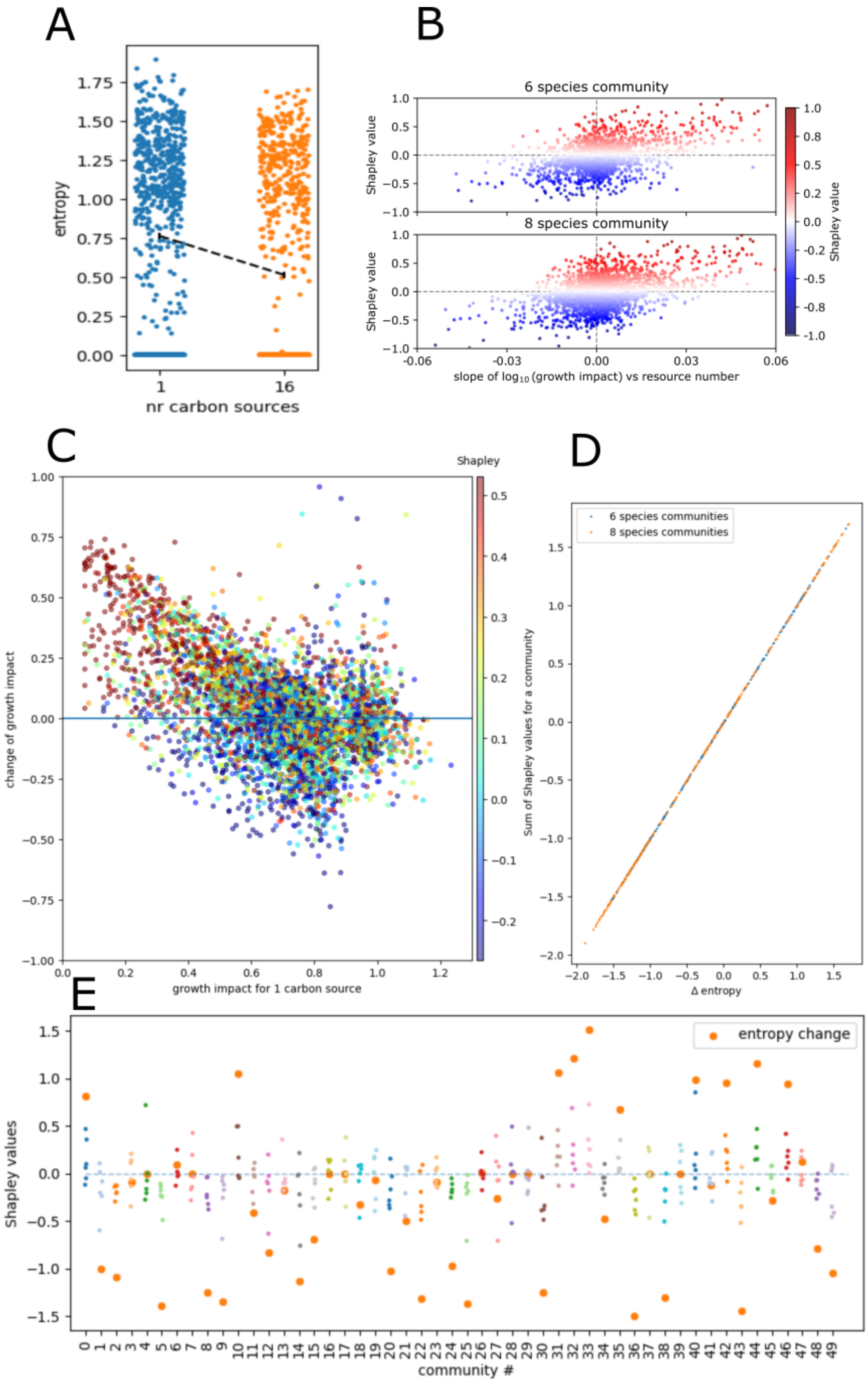


**Supplementary Figure 11: Shapley analysis under varying resource conditions. A.** Effect of resource number on community entropy, interpreted through a Shapley-value lens. Each point represents the entropy of a simulated community under either 1 or 16 resources. The dashed arrow shows the average marginal contribution of increasing resource number to entropy, analogous to a Shapley value. The downward trend reflects a consistent negative effect of resource richness on diversity. **B.** Same analysis as in Main Fig. 2C, shown separately for communities with six and eight species. No significant difference is observed between the two cases. **C.** Both the change in impact factor with increasing resource number and the initial impact factor at one resource influence community diversity. Microbes that are initially weak suppressors but become more suppressive as resource

complexity increases tend to reduce community entropy. However, some strains with little or no change in impact factor—and weak initial suppressiveness—still show high Shapley values (blue dots), likely due to interactions with more dominant strains. We speculate that biodiversity loss may result from a combination of increasingly suppressive strains and others that are outcompeted by them. While impact factor is a useful single-strain trait, many strains deviate from this trend, underscoring the emergent and context-dependent nature of community-level properties. **D.** As expected from theory, Shapley values across all species sum to the total change in the selected outcome—in this case, the change in Shannon entropy with increasing resource richness. **E.** Shapley values for the first 50 communities, along with their total change in entropy (sum of individual species' Shapley values; see panel D). In some communities, entropy change is driven by a single species, while in others it results from the combined effects of multiple strains with large absolute Shapley values.

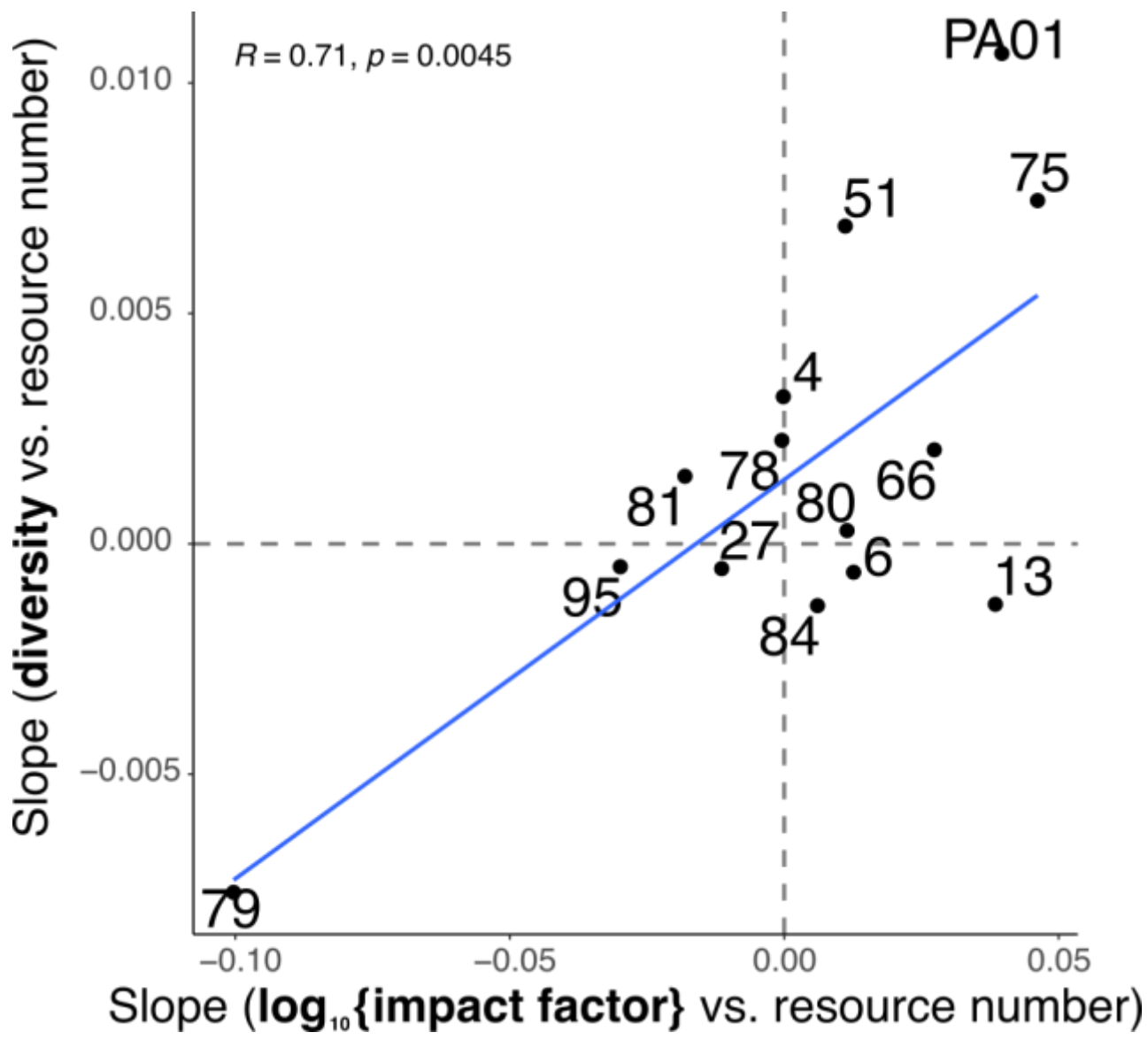


**Supplementary Figure 12: Our experimental results support the same conclusion as the simulations: strains with increasingly negative impact factor slopes drive biodiversity loss, while those with positive slopes have the opposite effect.** X-axis: Slope ($\beta_1$) of $\log_{10}$(impact factor) vs. number of resources, derived from a fitted linear model: $\log_{10}$(impact factor) = $\beta_0 + \beta_1 \times$ number of resources + $\varepsilon$. Y-axis: Slope ($\beta_1$) of Shannon diversity index vs. number of resources, derived from a fitted linear model: diversity = $\beta_0 + \beta_1 \times$ number of resources + $\varepsilon$. The relationship between the two slopes is shown as a fitted linear model. Pearson's correlation coefficient (r) and p-value (p) are indicated in the top left. Strain names are indicated next to each point.

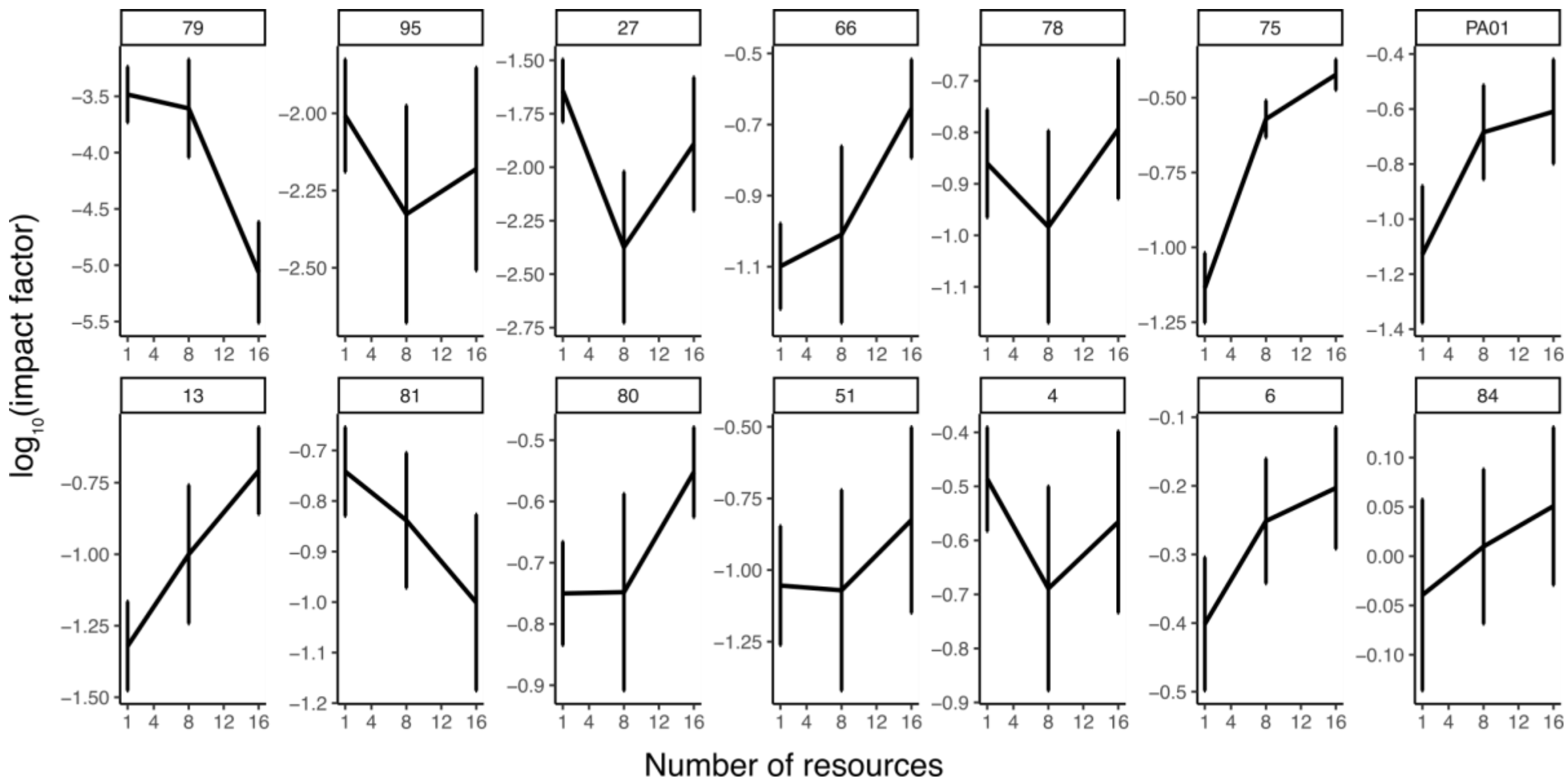


**Supplementary Figure 13: Impact factor of all 14 strains across resource complexity.** Supplementary results for Fig. 2D. Shown is the mean impact factor across 1, 8, and 16 resources for all strains, with error bars indicating ± SEM. Impact factor for each strain was averaged across its interactions with all other 13 strains (i.e., all pairwise interactions) and its growth in isolation (formula and illustration in Fig. 2A).

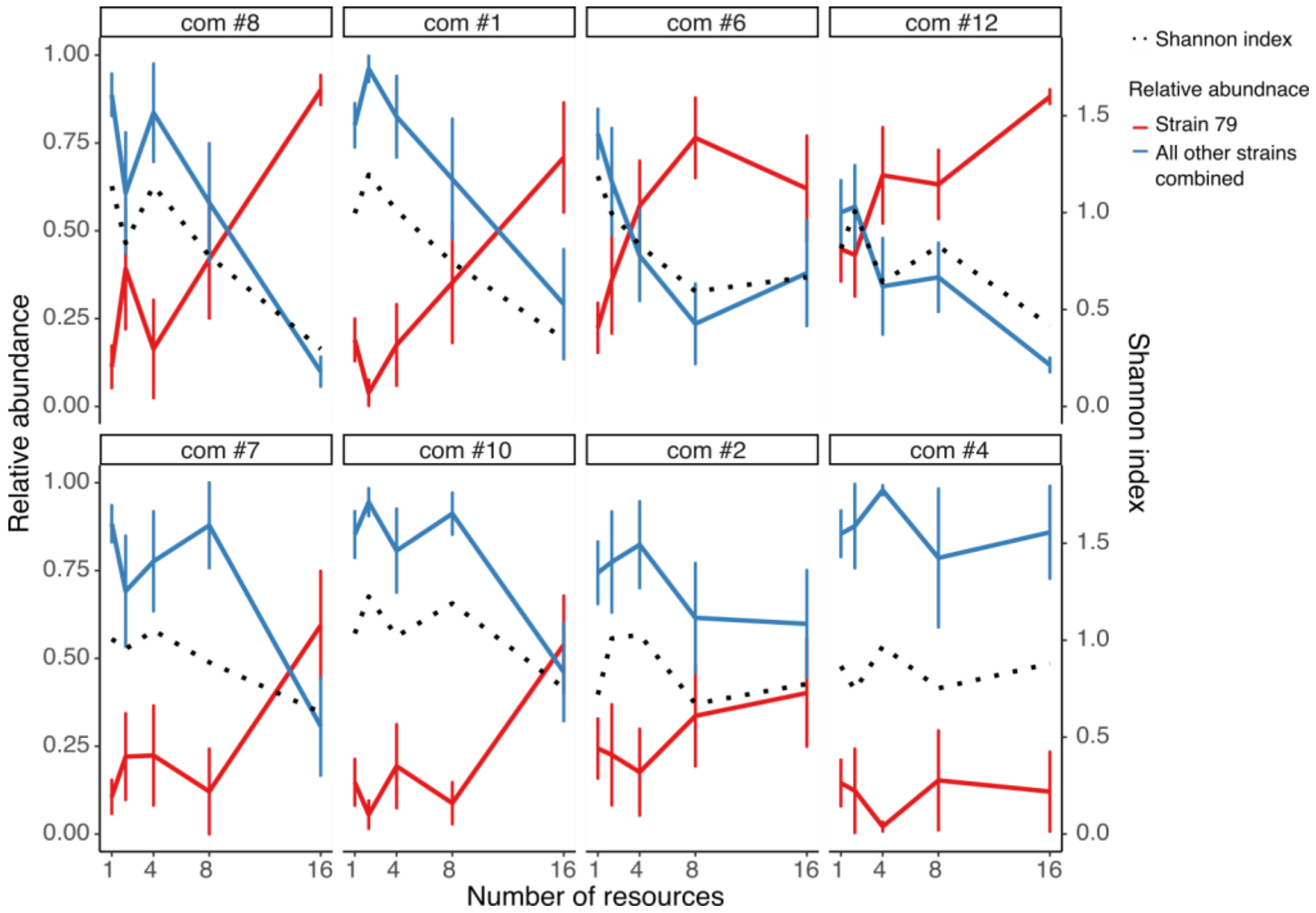


**Supplementary Figure 14: Strain '79' dominance increases with resource complexity, reducing diversity.** Supplementary results for Fig. 2F. Strain '79' exhibits a sharp increase in relative abundance with resource complexity, while the combined relative abundance of all other strains declines. Shannon's diversity index follows this trend, decreasing in parallel with the overall reduction of other strains. Shown are all eight communities containing strain '79', ordered by the slope of Shannon diversity decline between individual resources and 16-resource conditions. The trend line represents the average, with error bars indicating ± SEM.

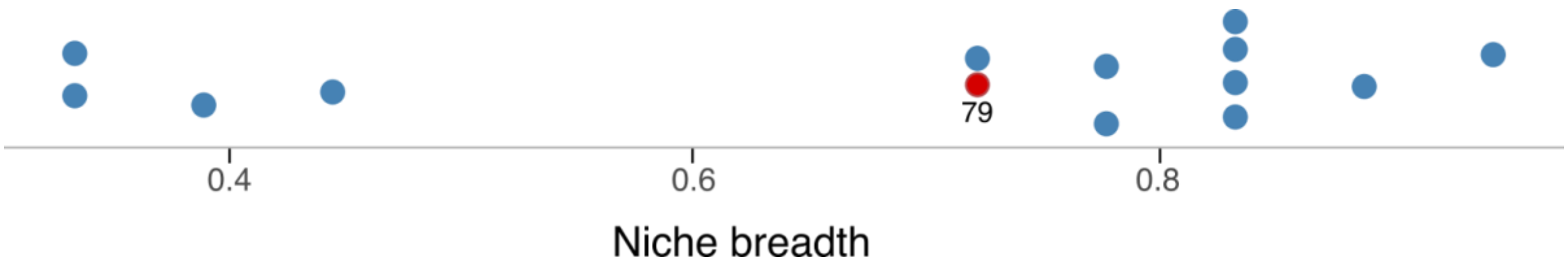


**Supplementary Figure 15: Strain 79 does not exhibit an exceptionally large niche breadth despite its strong biodiversity effect.** Shown is the niche breadth of strain 79 compared with the other 13 strains used in the pairwise and community experiments (e.g., Fig. 1D and Fig. 2E). Niche breadth was calculated as the proportion of resources on which each strain showed growth above an $OD_{600}$ of 0.15—a conservative threshold exceeding the maximum OD observed in any control well.

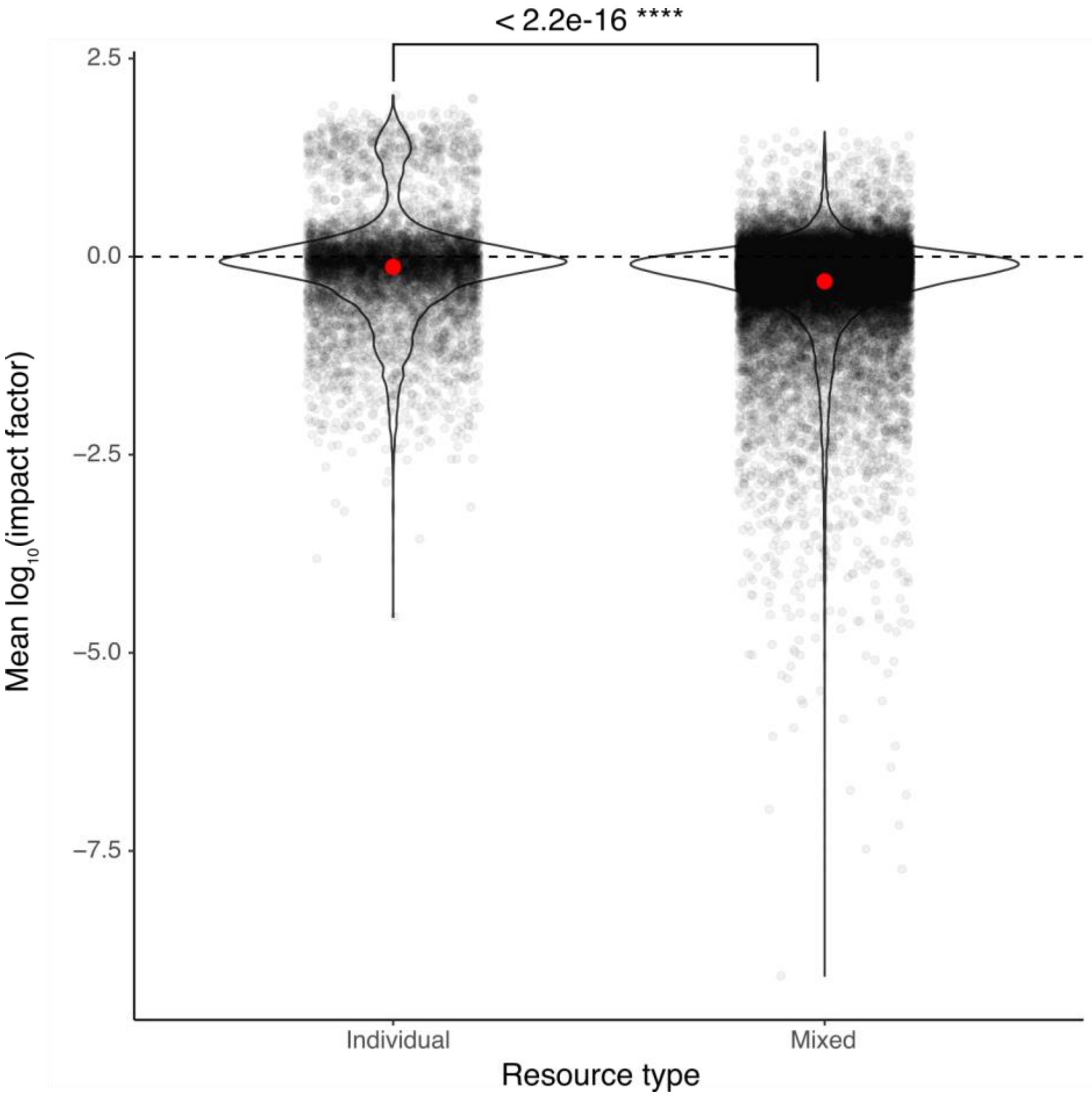


**Supplementary Figure 16: Impact factor on the sentinel strain is more suppressive in mixed resources.** The red dot represents the mean, and the dashed line marks zero impact, indicating no effect on sentinel strain growth. Statistical significance was assessed using a Mann-Whitney U test. In total, 6,556 impact factor values were calculated for individual resources and 26,660 for resource mixtures.

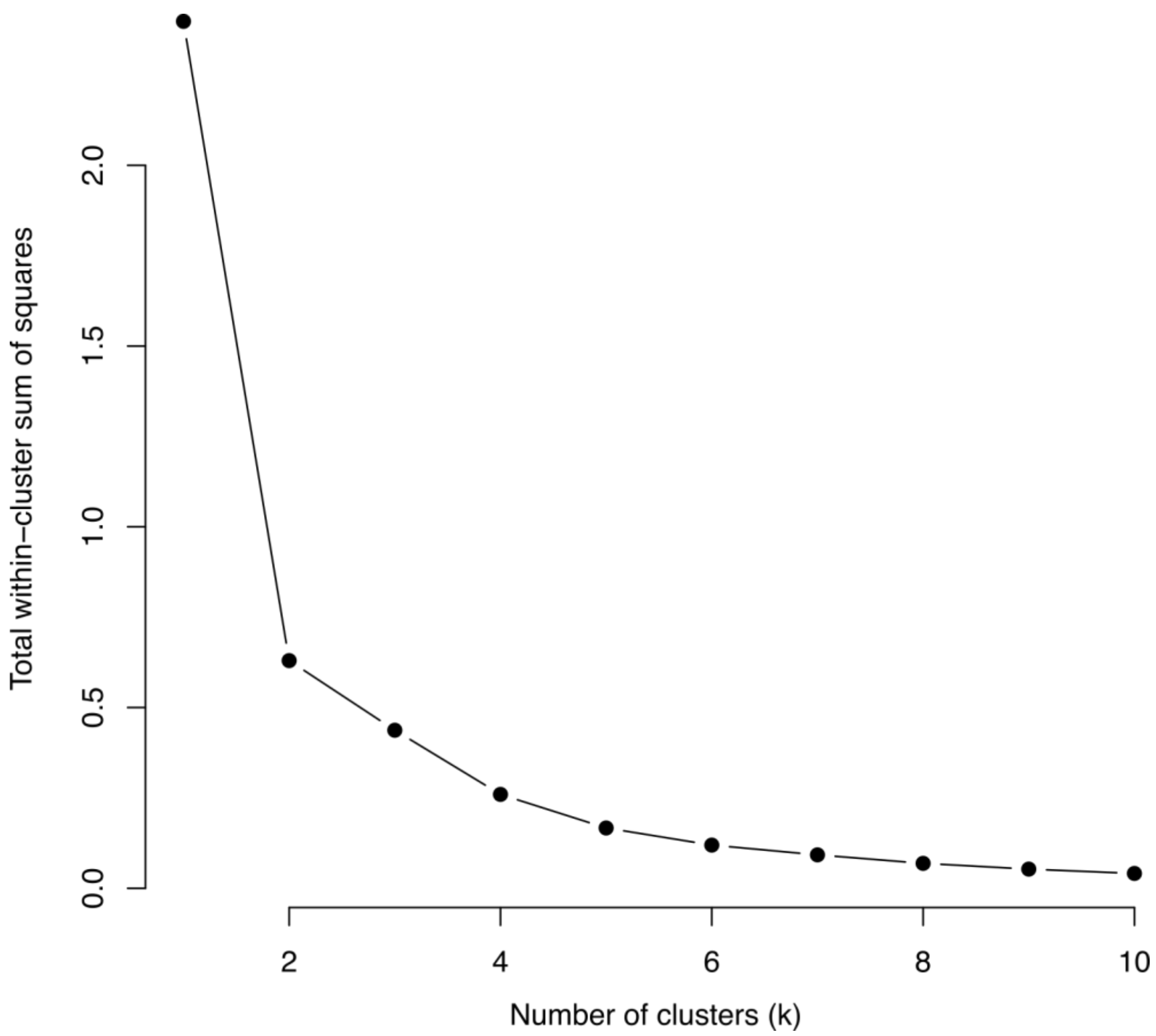


**Supplementary Figure 17: K-means clustering was used to classify strains into two types (highly and mildly influenced).** The optimal number of clusters was determined using the elbow method, where the 'elbow' represents the point at which adding more clusters results in only a minimal reduction in clustering error. As shown, the optimal number of clusters is two.

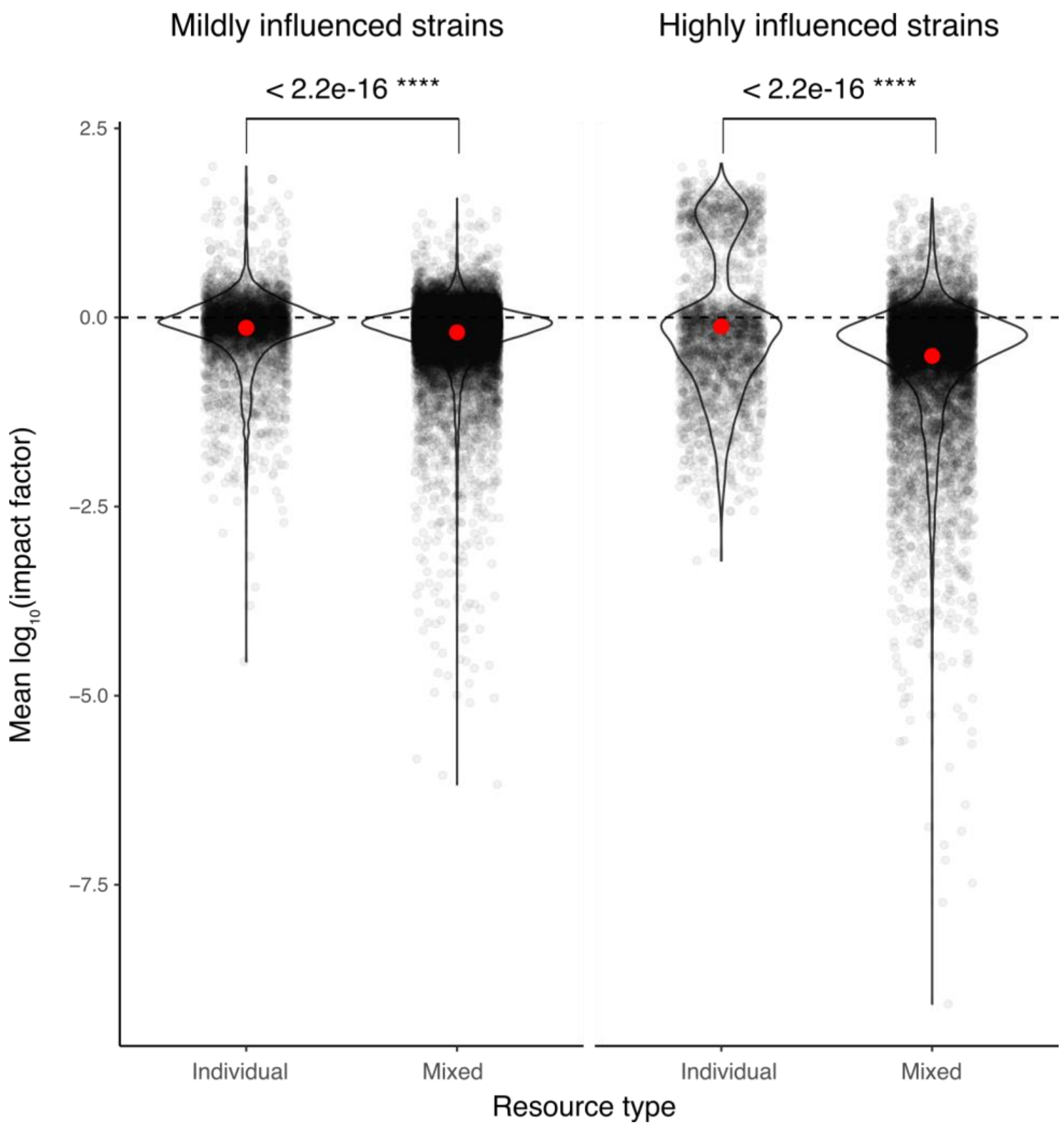


**Supplementary Figure 18: Impact factor on the sentinel strain is more suppressive in mixed resources for both highly and mildly influenced strains.** The red dot represents the mean, and the dashed line marks zero impact, indicating no effect on sentinel strain growth. Statistical significance was assessed using a Mann-Whitney U test. In total, 6,556 impact factor values were calculated for individual resources and 26,660 for resource mixtures.

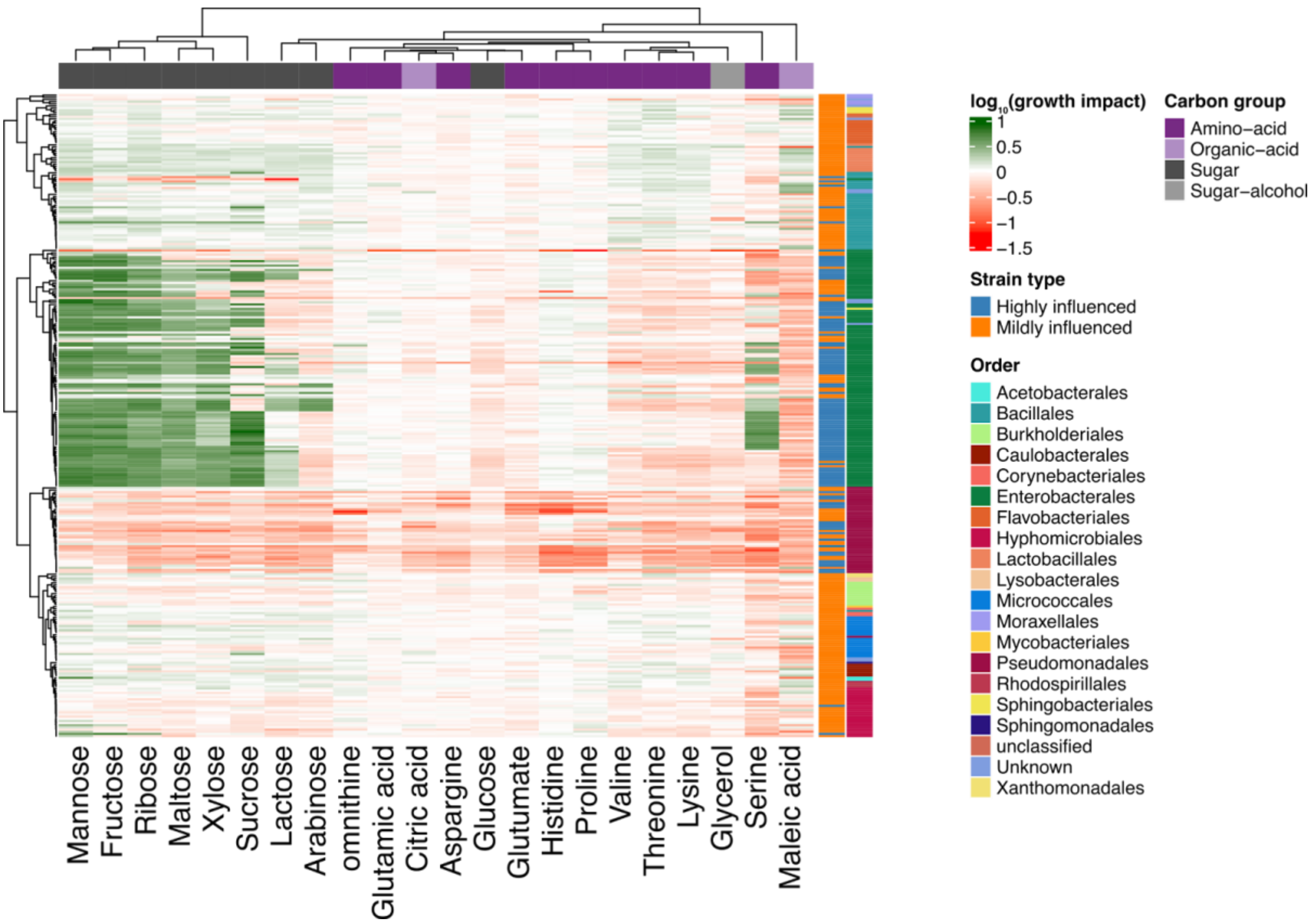


**Supplementary Figure 19: Taxonomy of the 298 strains and their growth impact on the sentinel strain across individual carbon sources.** Supplementary results for Fig. 3B. Taxonomic classification of all 298 strains is shown at the order level, together with their phylogeny (see Methods for details). Also displayed are the strain types (highly or mildly affected), their growth impact on the sentinel strain across the 22 individual carbon sources used in this study, and the corresponding chemical group of each carbon source (amino acid, organic acid, sugar, or sugar alcohol).

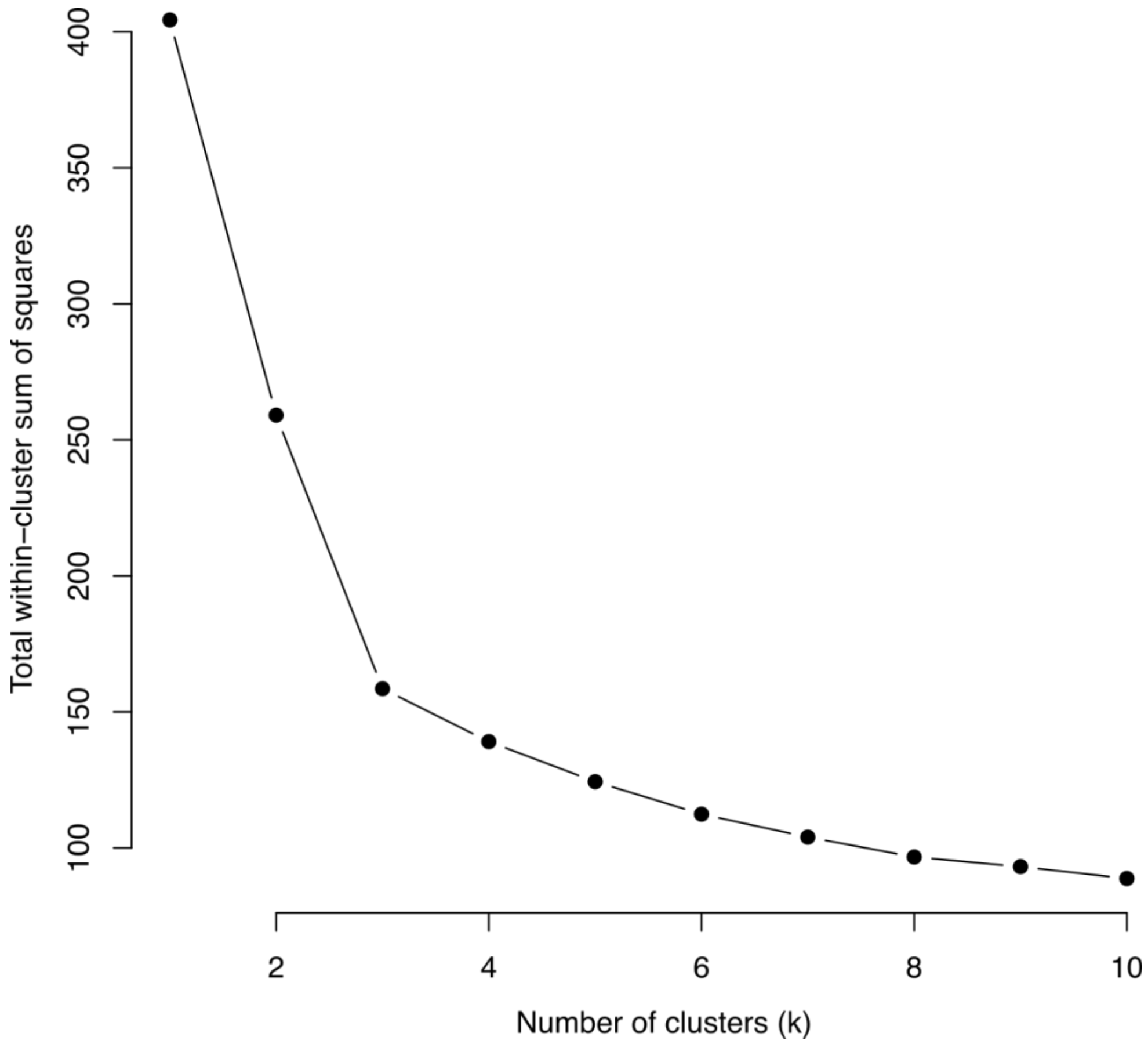


**Supplementary Figure 20: K-means clustering was used to classify strains into three types based on their interactions in individual resources.** The optimal number of clusters was determined using the elbow method, where the 'elbow' represents the point at which adding more clusters results in only a minimal reduction in clustering error. As shown, the optimal number of clusters is three.

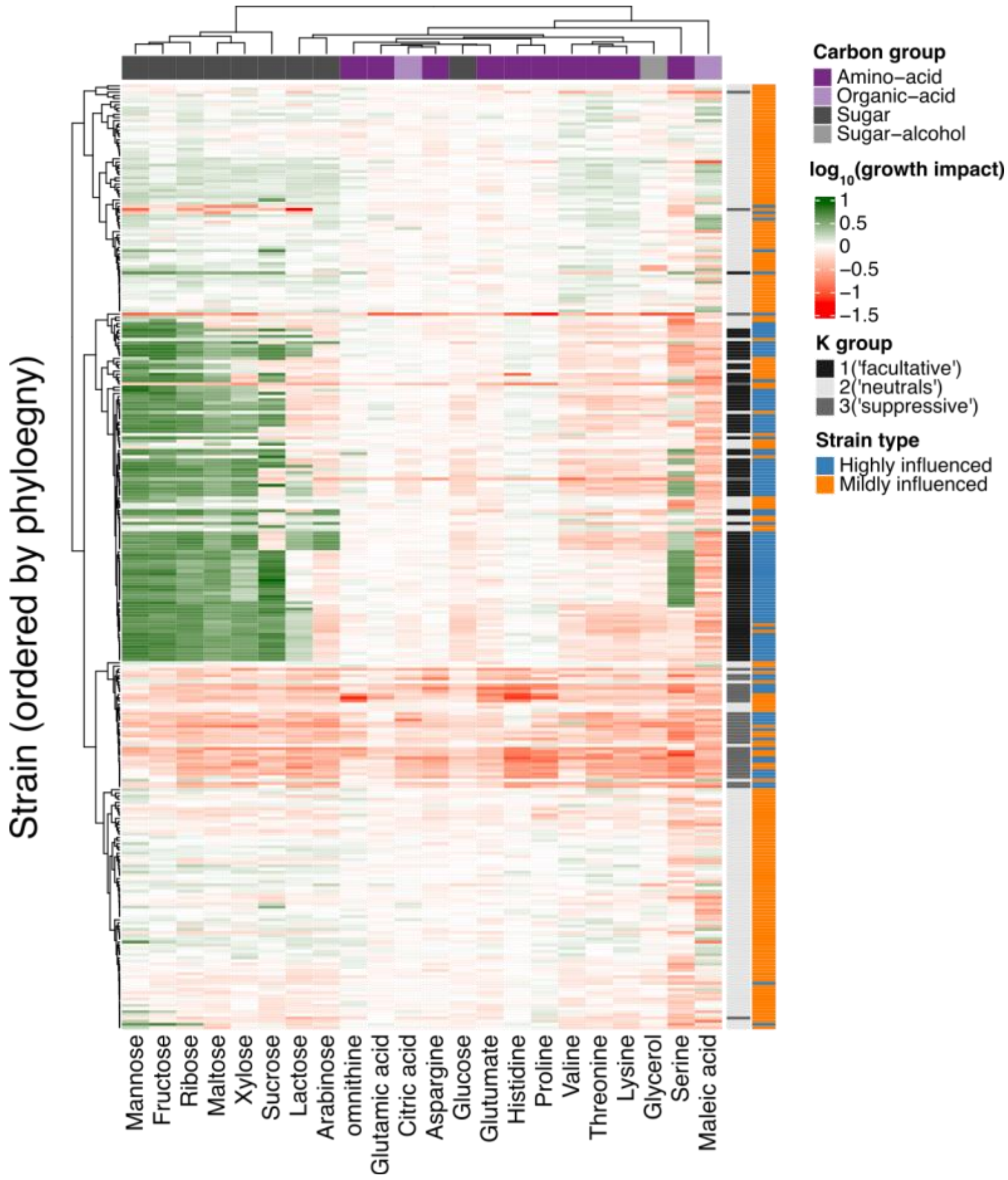


**Supplementary Figure 21: Highly and mildly influenced strains are categorized into three interaction-type clusters based on their behavior in individual resources.** clustering to three groups decided based on supplementary Fig. 16. Highly influenced strains primarily fall into the 'facultative' group (K-group 1; shifting from facilitation in sugars to suppression in acids) and the 'suppressive' group (K-group 3; consistently suppressive). In contrast, mildly influenced strains mainly belong to the 'neutral' group (K-group 2; exerting only mild effects on the sentinel strain without significantly impacting its growth). The same data is presented in Fig. 3B ('individual resources' heatmap).

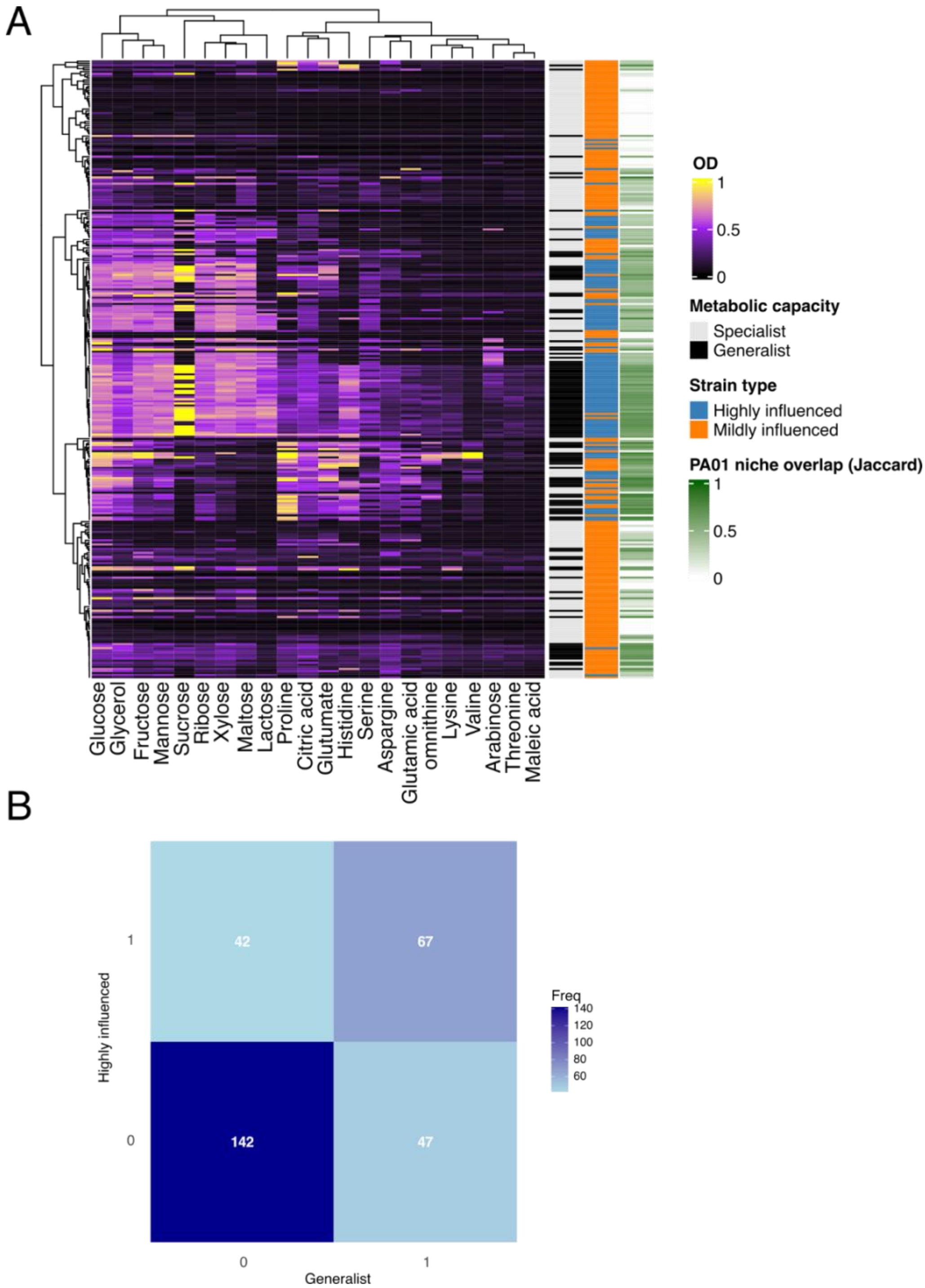


**Supplementary Figure 22: Highly- and mildly-affected strains are only weakly linked with resource generalism. A.** The full collection of 298 strains used in this study was grown across the 22 carbon resources included throughout the work. $OD_{600}$ was measured after 72 h (carrying capacity). Results are shown as a heatmap, with strains classified as generalists (niche breadth > 0.5) or specialists (niche breadth ≤ 0.5). Niche breadth was calculated as the number of resources supporting growth above control level ($OD_{600}$ > 0.15; this conservative threshold exceeds the maximum $OD_{600}$ observed in any control well). PAO1 niche overlap was calculated using the Jaccard distance. The classification of strains as highly or mildly affected is overlaid for comparison, and strains are ordered by core-genome phylogeny (see Methods). **B.** Confusion matrix comparing strain classifications as

highly or mildly affected (binary: 1 = highly affected, 0 = mildly affected) with their classification as generalists or specialists (binary: 1 = generalist, 0 = specialist). While 67 strains are both highly affected and generalists, 42 strains are highly affected specialists, and 47 are mildly affected generalists—indicating only a weak correlation between the two features ($r = 0.36$, $p < 0.001$; $\kappa = 0.36$; Jaccard = 0.43).

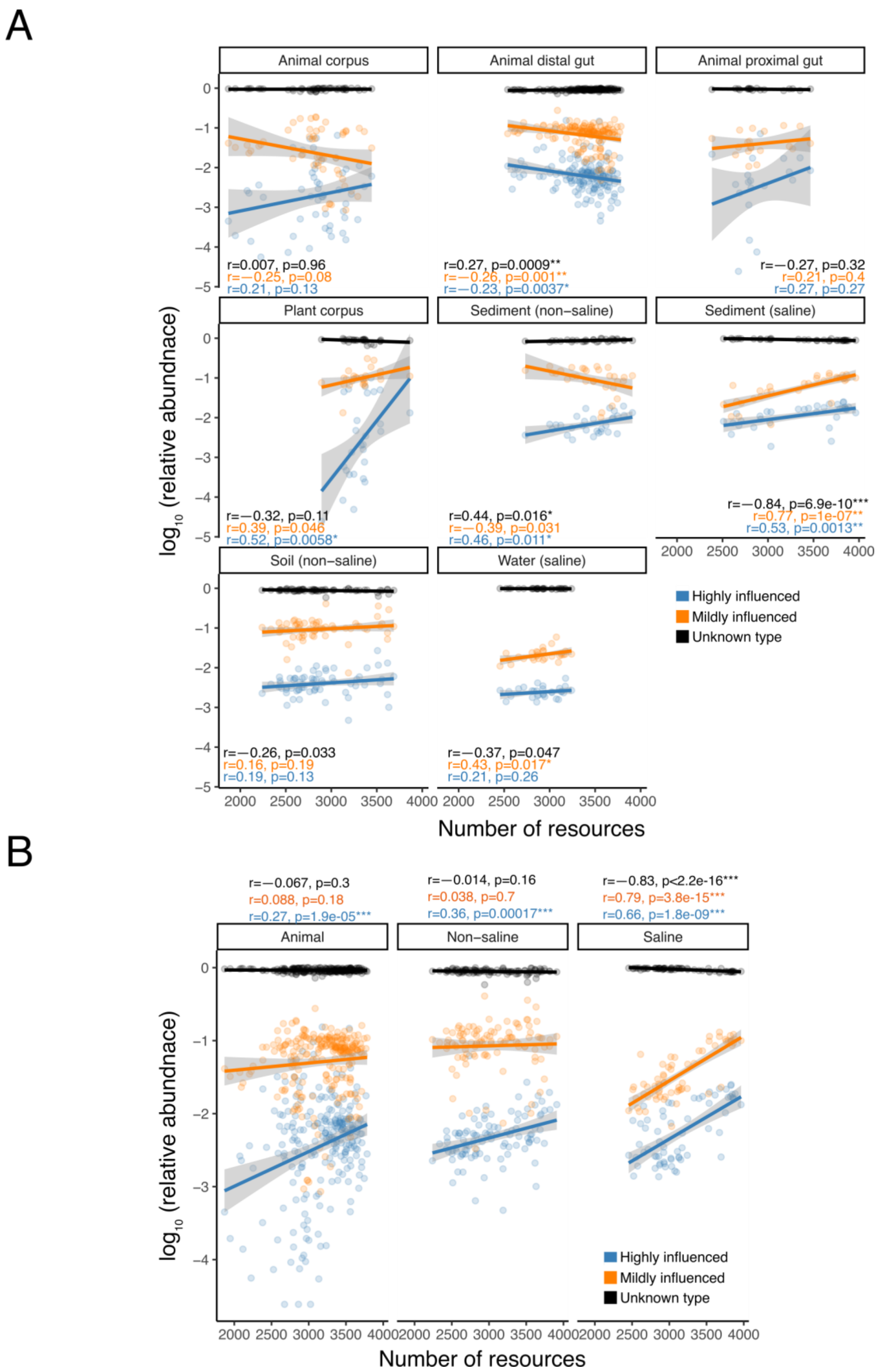


**Supplementary Figure 23: The relative abundance of highly influenced strains increases with resource complexity across environments.** Supplementary results for Fig. 3E**.** A mixed-effects

model was fitted to account for variation among environments (model: relative abundance ~ metabolite richness × strain type + (1 + metabolite richness | environment); $n = 1{,}224$). The relationship between metabolite richness and strain abundance differed significantly among strain types ($p = 2.5 \times 10^{-9}$). Highly influenced strains showed the strongest positive association with metabolite richness (slope = 0.165 ± 0.051), significantly greater than both mildly influenced (0.073 ± 0.051; $p = 0.0011$) and unmapped reads (0.001 ± 0.051; $p < 0.0001$); mildly influenced strains also differed modestly from unmapped reads ($p = 0.016$). Environment was included as a random effect, allowing both intercepts and slopes to vary. Random-effect variances for the intercept (0.015, SD = 0.12) and slope (0.013, SD = 0.11) were small compared with the residual variance (0.136, SD = 0.37), indicating that while environments differ slightly in their mean abundance and slope, the overall pattern—especially for highly influenced strains—is consistent across environments and not driven by any single ecosystem type. **A.** Relationships between metabolite richness and the abundance of highly influenced, mildly influenced, and unmapped strains within each environment, using the EMPO3 classification applied throughout the manuscript. Despite finer resolution, the overall pattern recapitulates Fig. 3E: the relative abundance of highly influenced strains increases with resource complexity, whereas mildly influenced and unmapped reads do not. **B.** Same analysis as in panel A but using the coarser EMPO2 environmental classification. The same general trend is observed, with only highly influenced strains showing significant positive slopes in all three broad environments. Raw (unadjusted) p-values are displayed in each panel. Statistical significance was assessed after Benjamini–Hochberg correction for multiple comparisons, and asterisks denote adjusted p-values (*$p_{adj} < 0.05$, **$p_{adj} < 0.01$, ***$p_{adj} < 0.001$).

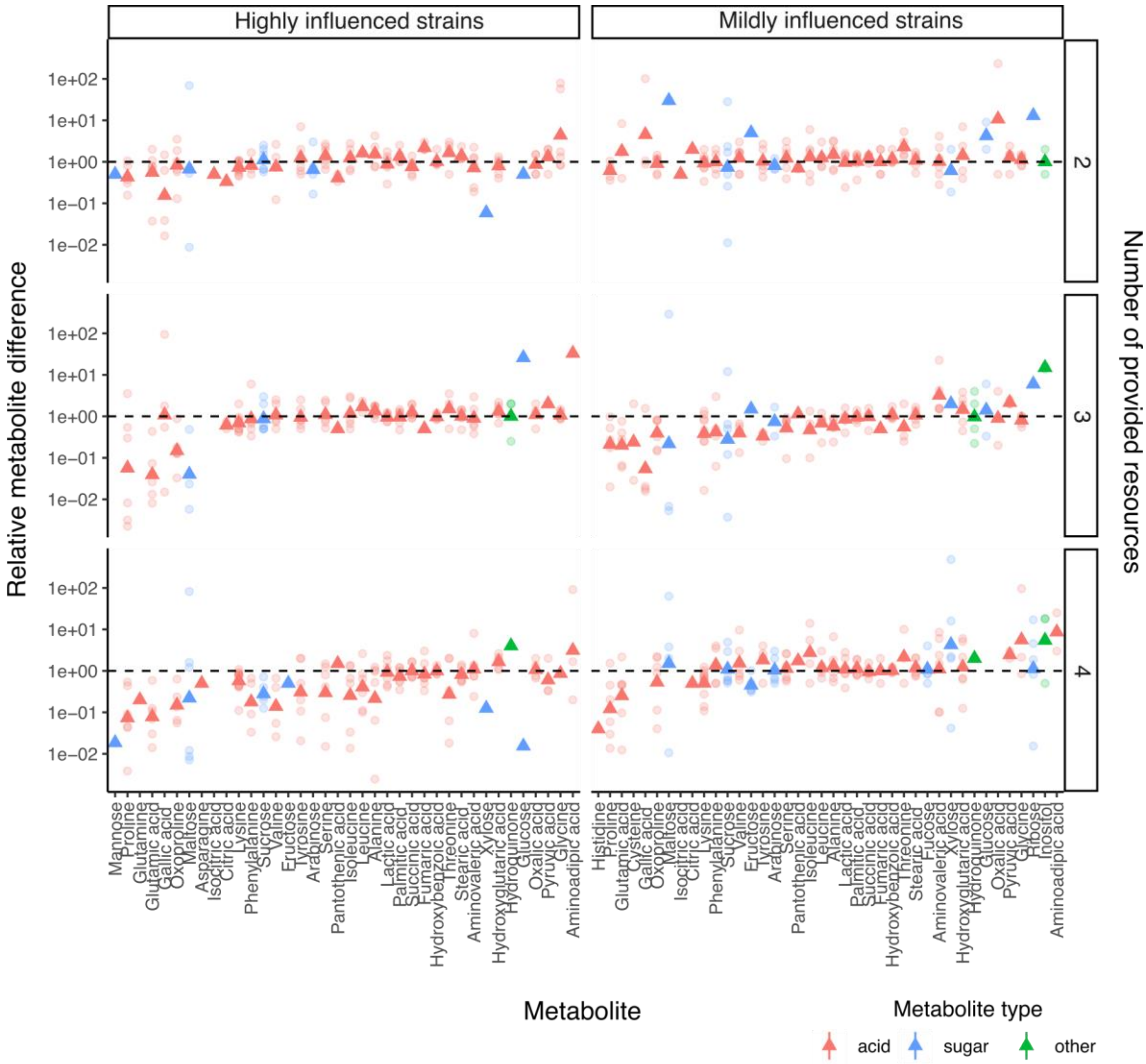


**Supplementary Figure 24: Highly influenced strains show greater metabolic uptake in resource mixtures than in their supernatant mixtures for specific metabolites, with this effect intensifying as resource complexity increases.** Supplementary results for Fig. 4B. Shown is the relative change in metabolite peak area (normalized to an internal control) for 96 identified metabolites, comparing resource mixtures to their matching supernatants. Points represent individual metabolites within a specific sample, while triangles indicate the mean. The y-axis is displayed on a $\log_{10}$ scale.

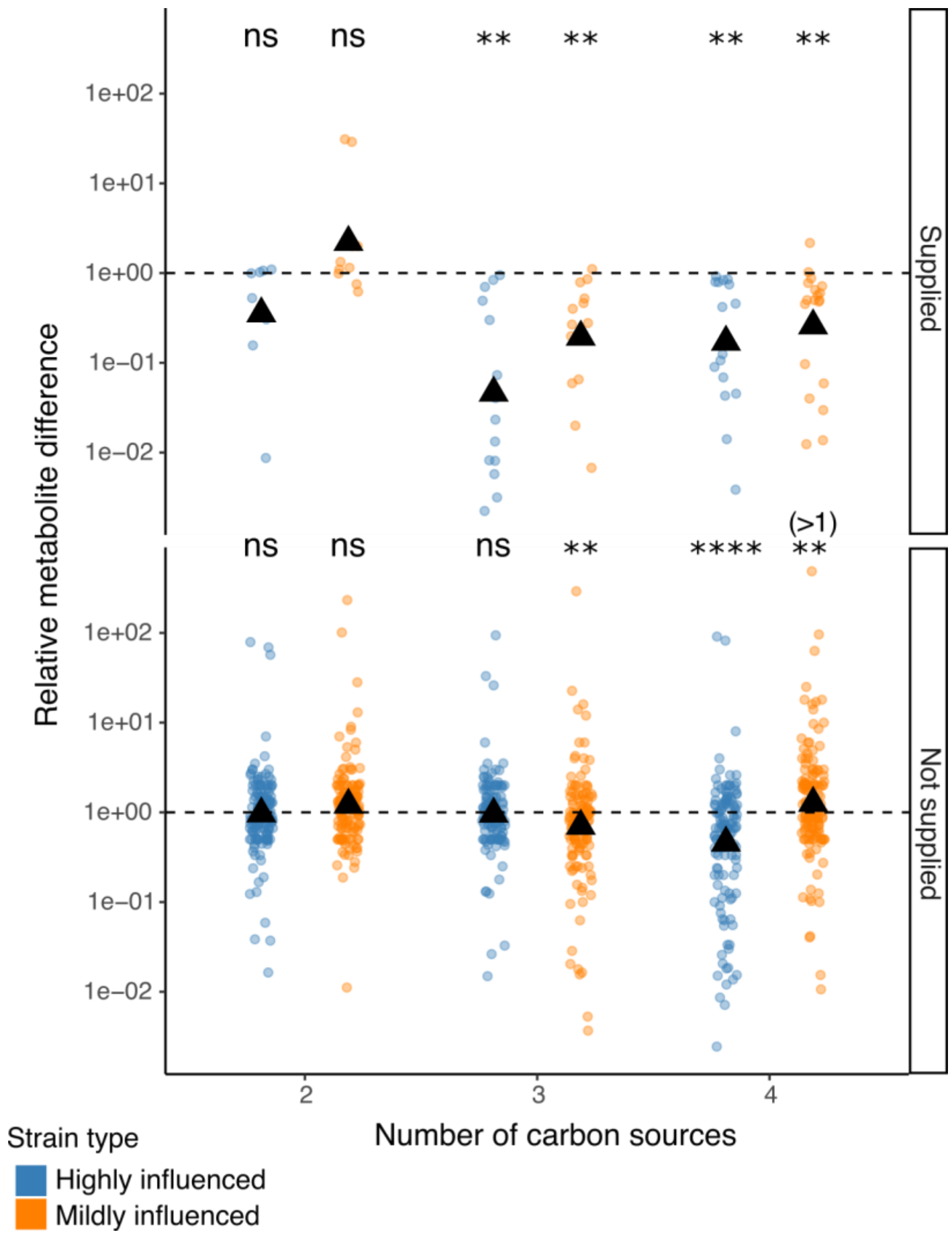


**Supplementary Figure 25: Highly influenced strains show greater metabolic uptake in resource mixtures than in their supernatant mixtures, for both supplied and not supplied resources, with this effect intensifying as resource complexity increases.** Supplementary results for Fig. 4B. Shown is the relative change in metabolite peak area (normalized to an internal control) for 96 identified metabolites, comparing resource mixtures to their matching supernatants. Data are binned by 'supplied' resources (metabolites that were part of the defined media and initially added) and 'not supplied' resources (metabolites likely excreted, as they were not provided in the growth media). Points represent individual metabolites within a specific sample, while triangles indicate the mean. The y-axis is displayed on a $\log_{10}$ scale. A Mann-Whitney U test assessed whether the median relative metabolite difference significantly deviated from 1. For mildly influenced strains with four resources in the 'not supplied' category, values >1 indicate more more produced metabolites detected in the resource mixture (lower uptake), whereas significance otherwise indicates higher uptake. Statistical significance was assessed after Benjamini–Hochberg correction for multiple comparisons, and asterisks denote adjusted p-values (*p_adj < 0.05, **p_adj < 0.01, ***p_adj < 0.001).

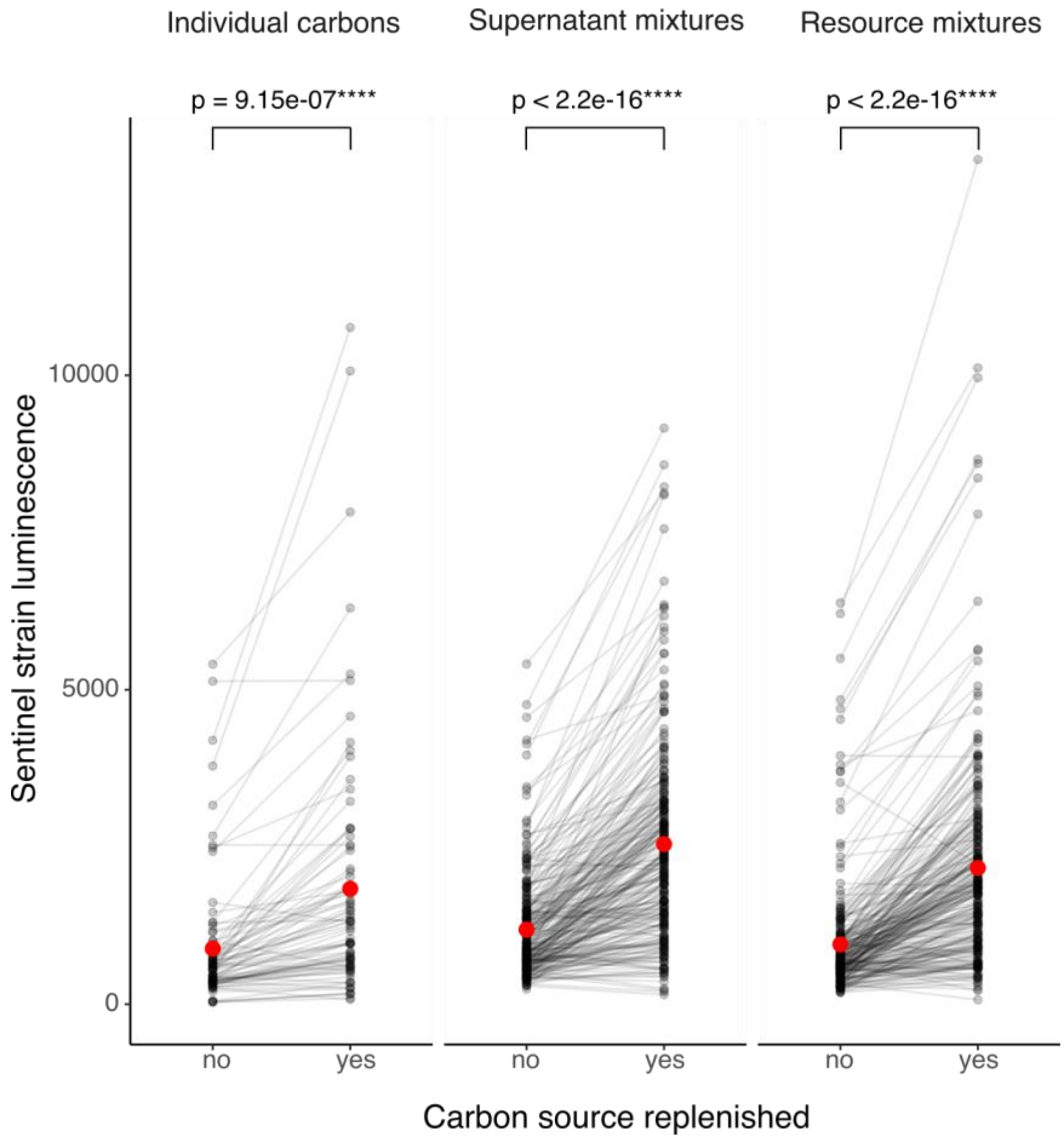


**Supplementary Figure 26: Media replenishment restored normal growth of the sentinel strain in most supernatants, ruling out toxicity as a primary competition driver.** Supplementary results for Fig. 4C. Fresh media containing the matching resources for each sample was added at a 1:10 dilution (9 parts supernatant, 1 part fresh media). The sentinel strain was grown in supernatants with and without replenishment. As shown, replenishment restored growth in most cases. Points represent individual samples (sentinel strain growth in a specific supernatant), with lines connecting the same samples before and after replenishment. The red dot indicates the mean. Replenishment was performed across all three supernatant types—individual carbon sources, supernatant mixtures, and resource mixtures (illustrated in Fig. 4A). Statistical significance was assessed using a Mann-Whitney U test. In total, 84 individual carbon supernatants, 252 supernatant mixtures, and 252 resource mixtures were tested, each with and without replenishment (represented as separate conditions on the x-axis).

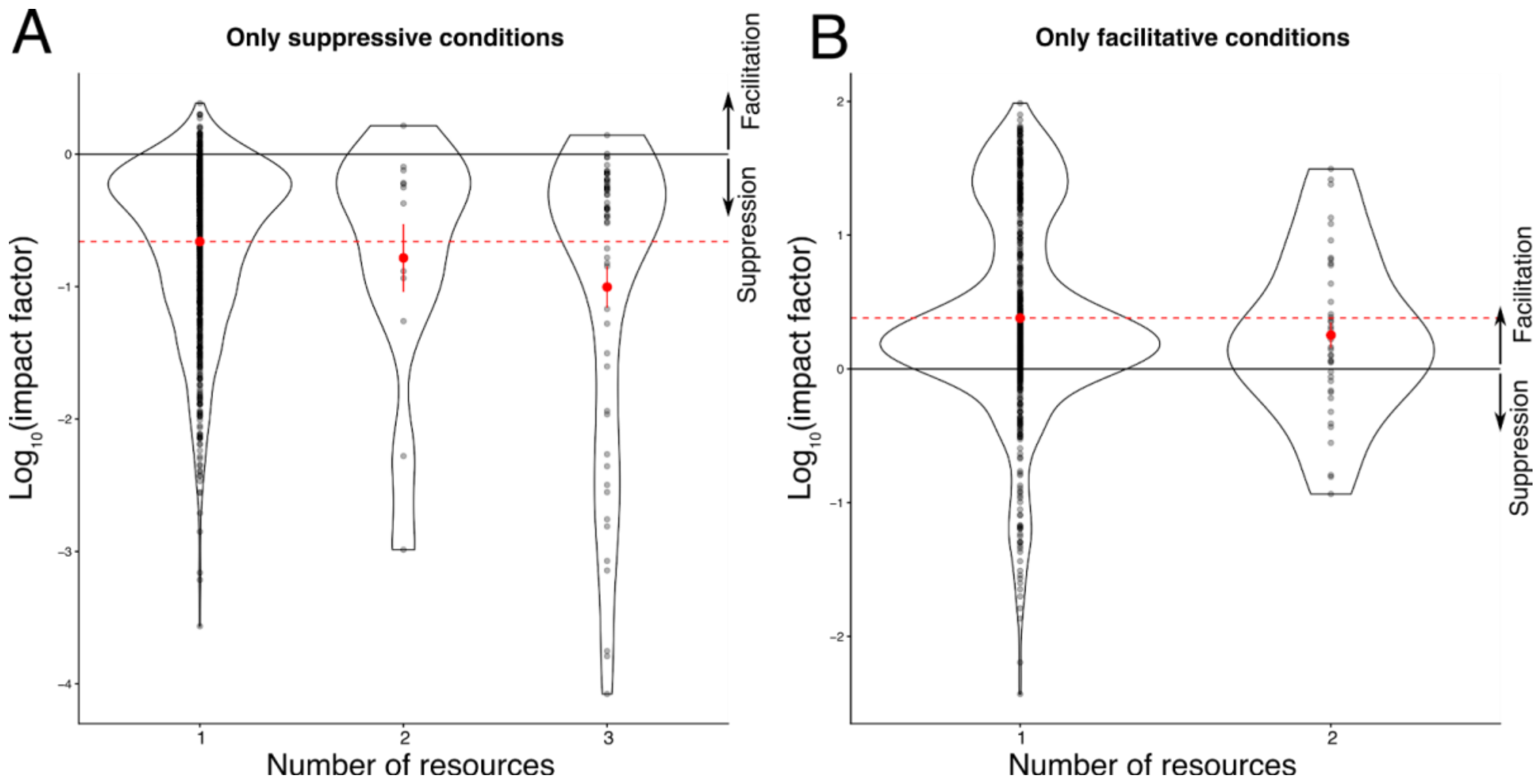


**Supplementary Figure 27: Facilitation declines and suppression intensifies with increasing resource number, even within exclusively facilitative or suppressive subsets.** In both only-suppressive (**A.**) and only-facilitative (**B.**) conditions—defined by strain–resource combinations that predominantly produced suppression or facilitation in single-resource environments (for example, fermentable sugars with facilitative strains versus amino acids with suppressive strains; see Supplementary Fig. 21)—facilitation declined and suppression increased with resource number. Under facilitative conditions, strong facilitation events became increasingly rare as the number of resources grew, evident from the loss of the upper tail in the violin plots (**B.**). Under suppressive conditions, mixtures of three resources produced markedly stronger inhibition than any single-resource environment, with several data points falling below the minimum responses observed for single resources, despite the smaller sample size (**A.**). These patterns mirror the global trends shown in Supplementary Figs. 16 and 18, confirming that both the loss of facilitation and the intensification of suppression arise from the same underlying non-additive process. The dashed red line represents the mean of individual carbon sources, the red dot indicates the average, and the error bars show the standard error of the mean (SEM). Notably, although no resource classes were imposed a priori, only-facilitative conditions (B) predominantly correspond to fermentable sugars, whereas only-suppressive conditions (A) primarily correspond to amino acids, consistent with co-utilization classes described by Wang et al. (2019, Nat. Commun.). Increasing resource number led to reduced facilitation and stronger inhibition, particularly in higher-order mixtures. These patterns are inconsistent with a diauxic-only interpretation, which would predict weaker competition as resource number increases.

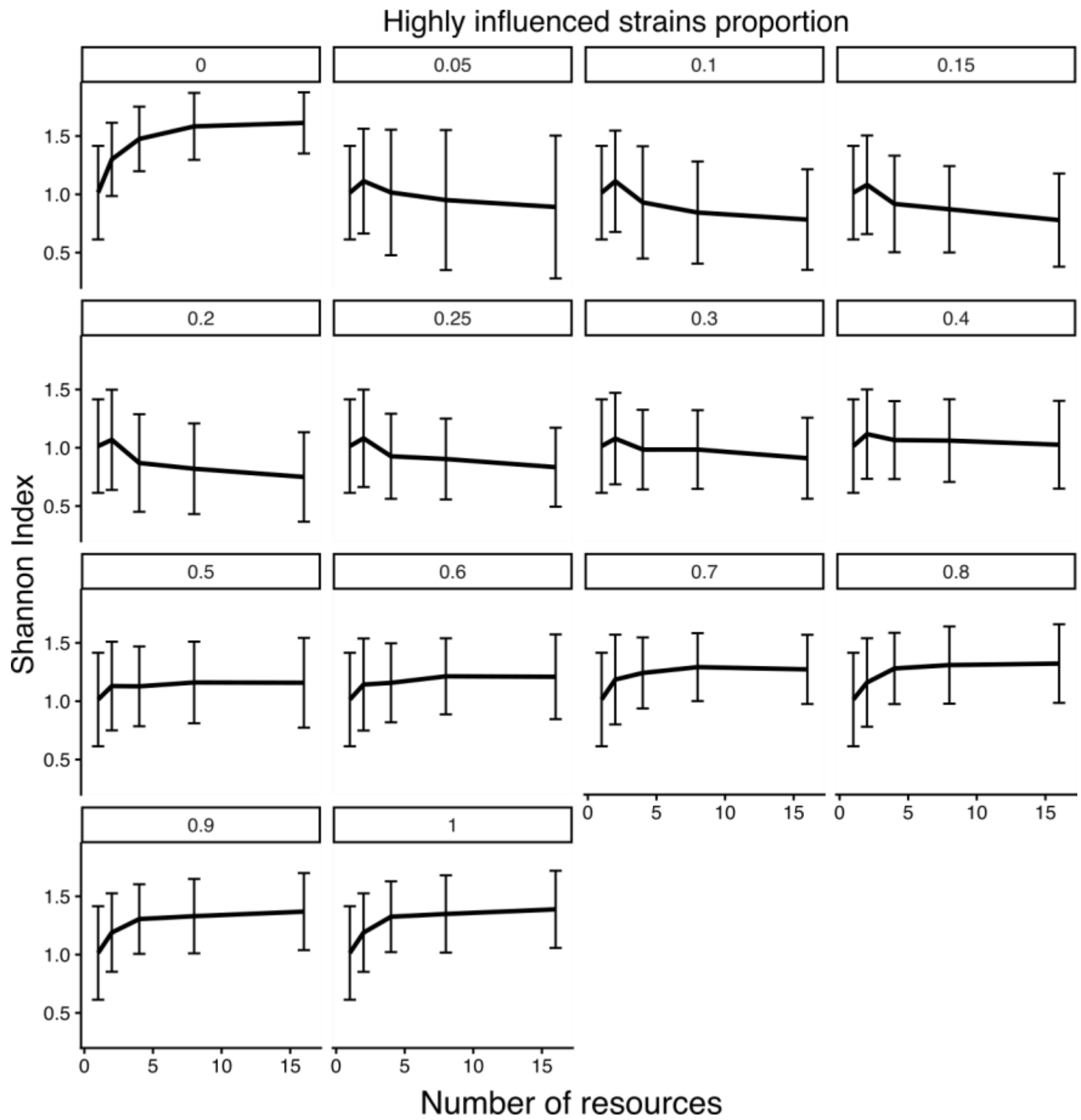


**Supplementary Figure 28: Highly influenced strain behavior can reduce microbial diversity as resource complexity increases in a consumer-resource model.** Supplementary results for Fig. 4E. We simulated communities composed of 20 randomly generated strains, each assigned random uptake and growth rates across 30 resources, across increasing resource complexity (1–16 resources) and a predefined set of highly influenced strain proportions (values shown above each panel). For each proportion, 21 independent communities were simulated (three replicates each) and Shannon diversity was computed as a function of resource number. Trend lines show the mean across communities for each proportion, with error bars indicating ± s.d. The 0% condition corresponds to the default consumer–resource model (as in Fig. 1A). Simulations shown here use $\gamma = 1$. The 0% proportion represents the default consumer-resource model (similar to Fig. 1A). The trend line represents the mean of 20 communities per highly influenced fraction, with error bars indicating ± SD.

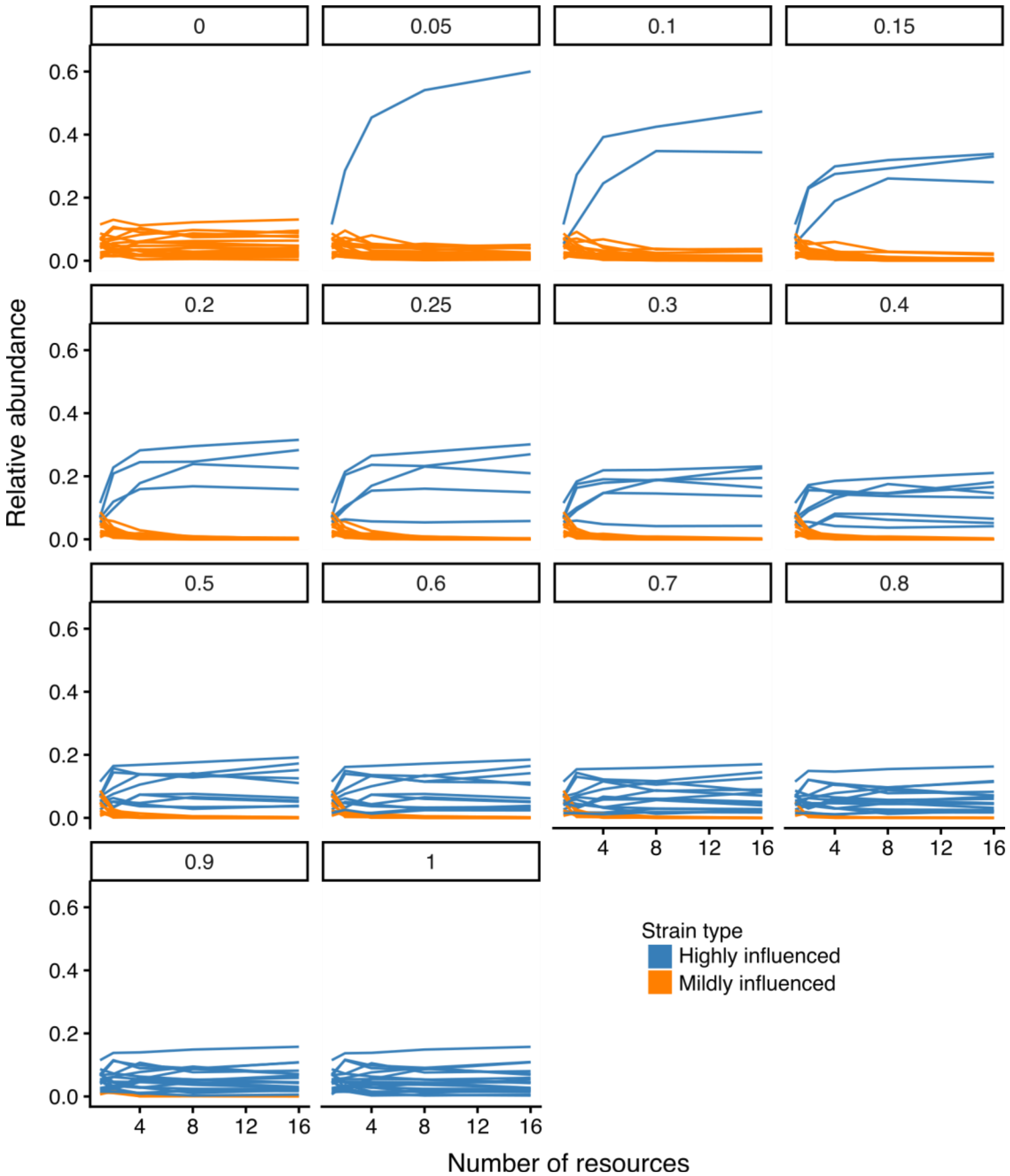


**Supplementary Figure 29: Highly influenced strain behavior reduces microbial diversity as resource complexity increases in a consumer-resource model, with highly influenced strains increasingly dominating.** Shown is the mean community composition across simulated communities for a set of predefined highly influenced strain proportions (values shown above each panel), with

strains categorized as highly or mildly influenced. Averages are taken across independent communities each proportion. Simulations shown here use $\gamma = 1$.

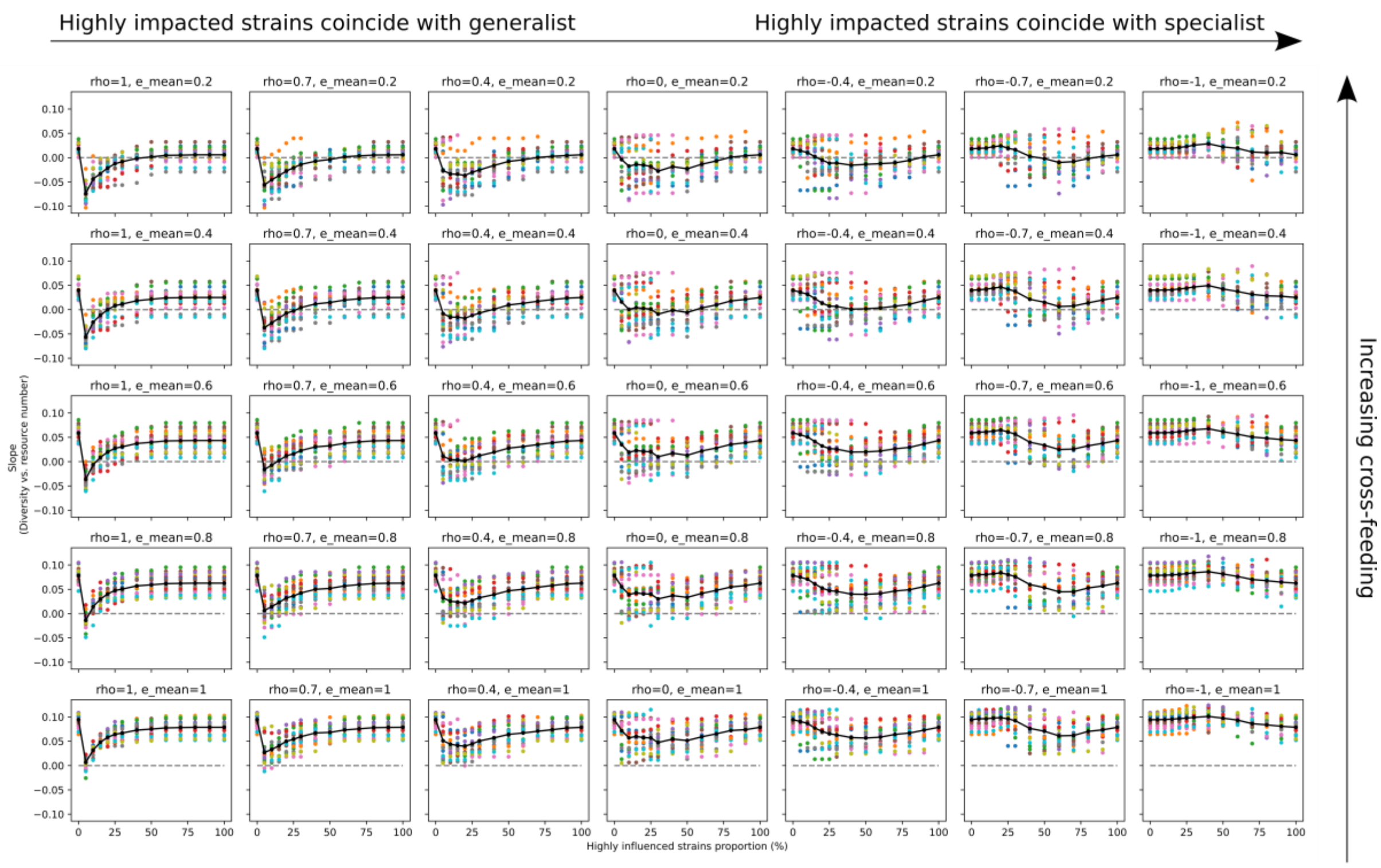


**Supplementary Figure 30: Robustness of microbial diversity-resource complexity slopes across cross-feeding levels and correlation levels between niche width (generalism) and highly influenced behavior.** We evaluated the impact of highly influenced strain behavior on microbial diversity across a broad parameter space within the Consumer-Resource Model (Supplementary Text 1 Section 3.1). Each panel displays the slope ($\beta_1$) from a linear model of Shannon diversity vs. resource number (y-axis) plotted against the proportion of highly influenced strains (f) in the community (x-axis, 0–100%). Points represent individual simulated communities (20 replicates), and the solid black trend line indicates the mean across communities. The dashed horizontal line at zero represents the baseline where microbial diversity is neutral to resource complexity. Trait Correlations (ρ): Columns represent the correlation between a strain's niche breadth (the fraction of resources that it can uptake) and its likelihood to be highly influenced by resource complexity. Positive values (ρ>0, left) indicate that generalist strains are more likely to be highly influenced, while negative values (ρ<0, right) indicate that specialist strains are more likely to be highly influenced. In main text Fig. 4E we used ρ=0.4 in accordance with experimental value 0.36 (Supplementary Figure 22). Cross-feeding (1-e_mean): Rows represent increasing levels of resource assimilation (e_mean) thus decreasing cross-feeding (1-e_mean); as e_mean ranges from 0.2 to 1.0, cross-feeding, or the average fraction of resources secreted back into the environment, ranges from 0.8 (80%) to 0 (0%). In main text Fig. 4E we used e_mean=0.4 in accordance with relative high biodiversity at single carbon source in experiment. Interpretations: The results demonstrate that the transition toward a negative diversity-complexity slope is more likely at an intermediate proportion of highly influenced strains; as the proportion further increases, the competition happens among a growing number of highly impacted strains, maintaining microbial diversity even at high resource complexity. Negative slopes are also more likely at higher cross-feeding rate, because it allows higher biodiversity under low resource complexity thus room for biodiversity loss as resource complexity increases. Finally, slopes become more negative if highly

influenced strains coincide with resource generalists, as they can leverage this competitive advantage across multiple niches to exert a greater impact.

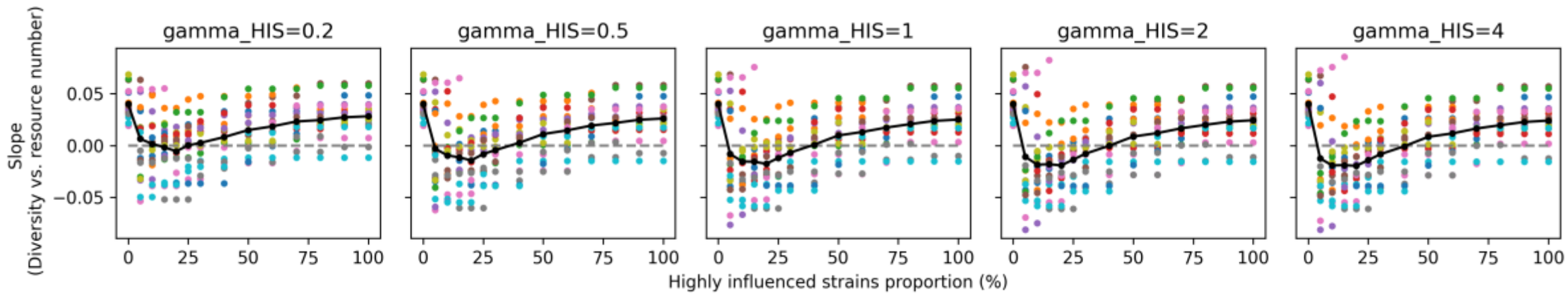


**Supplementary Figure 31: Robustness of microbial diversity-resource complexity slopes across varying levels of highly influenced strain sensitivity (γ).** We evaluated the impact of highly influenced strain behavior on microbial diversity under a range of sensitivity parameter (γ) within the Consumer-Resource Model (Supplementary Text 1 Section 3.2). Each panel displays the slope ($\beta_1$) from a linear model of Shannon diversity vs. resource number (y-axis) plotted against the proportion of highly influenced strains in the community (x-axis, 0–100%). Points represent individual simulated communities (20 replicates), and the solid black trend line indicates the mean across communities. The dashed horizontal line at zero represents the baseline where microbial diversity is neutral to resource complexity. We used resource assimilation e_mean=0.4 and trait correlation ρ=0.4 as in Supplementary Figure 30 and main text Fig. 4E for these simulations. **Strain Sensitivity (γ):** Panels represent the γ of highly influenced strains (ranging from 0.2 to 4, whereas 1 is used for main text Fig. 4E); the γ of mildly influenced strains is kept as 0. **Interpretations:** The results demonstrate that the observation of a negative diversity-complexity slope is robust across a wide range of highly impacted strain sensitivity levels.

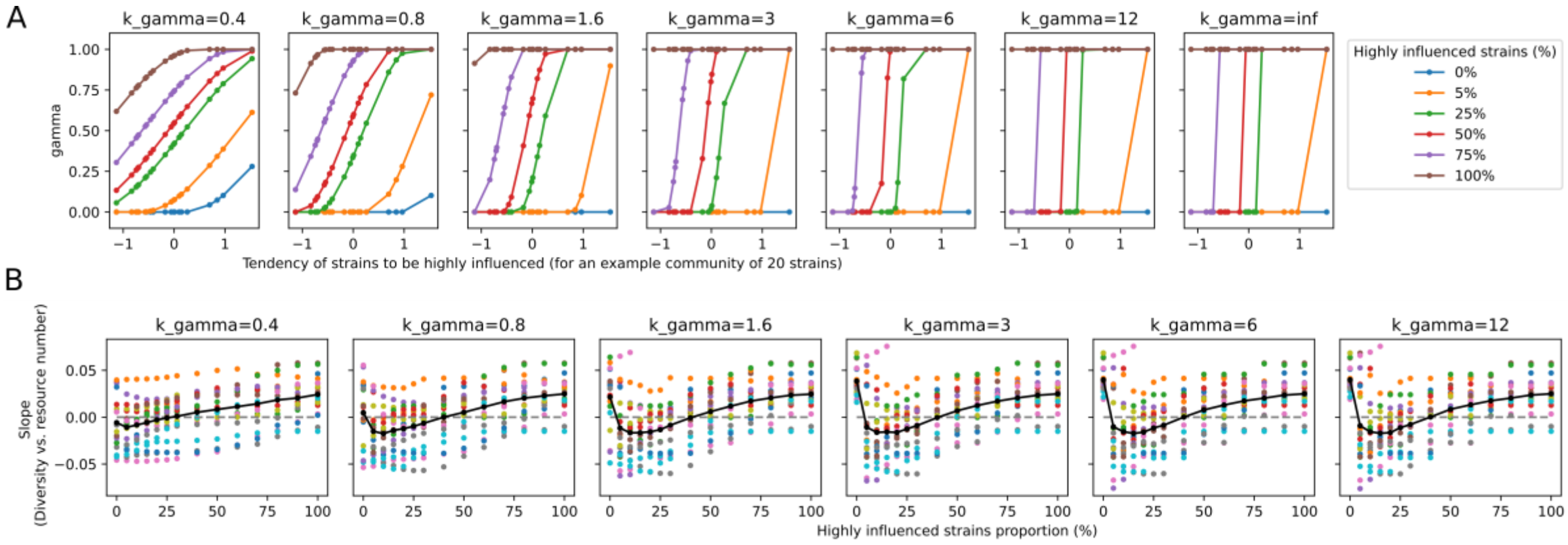


**Supplementary Figure 32: Robustness of microbial diversity-resource complexity slopes across a transition from binary to continuous highly influenced and mildly influenced strain behavior distributions.** In the main text we assumed a binary classification of highly influenced and mildly influenced strains; here we evaluated the impact if such behavior becomes a continuous instead of binary trait (Supplementary Text 1 Section 3.3). We evaluated the impact of highly influenced strain behavior on microbial diversity across various sharpness of the transition (k_gamma) between mildly and highly influenced strains within the Consumer-Resource Model (Supplementary Text 1 Section 3.3). **(A)** Distribution of strain sensitivity to resource complexity (γ) as a function of the underlying tendency to be influenced, which is drawn to be partially correlated with niche width (ρ=0.4). Panels represent the transition from a continuous distribution of sensitivity (k_gamma=0.4) to a near-binary classification (k_gamma=12) as k_gamma increases; whereas the main text Fig. 4E utilizes a strictly binary classification of mildly influenced and highly influenced strains (k_gamma=inf). **(B)** Panels represent the transition from a continuous distribution of sensitivity to a near-binary classification as in (A). Each panel displays the slope ($\beta_1$) from a linear model of Shannon diversity vs. resource number (y-axis) plotted against the proportion of highly influenced strains in the community (x-axis, 0–100%). Points represent individual simulated communities (20 replicates), and the solid black trend line indicates the mean across communities. We used resource assimilation e_mean=0.4 and trait correlation ρ=0.4 as in Supplementary Figure 30 for these simulations. The dashed horizontal line at zero represents the baseline where microbial diversity is neutral to resource complaexity. **Interpretations:** The results demonstrate that the negative diversity–complexity slope is robust, regardless of whether strain sensitivity to resource complexity is treated as a binary classification (mildly vs. highly influenced) or as a continuous trait.

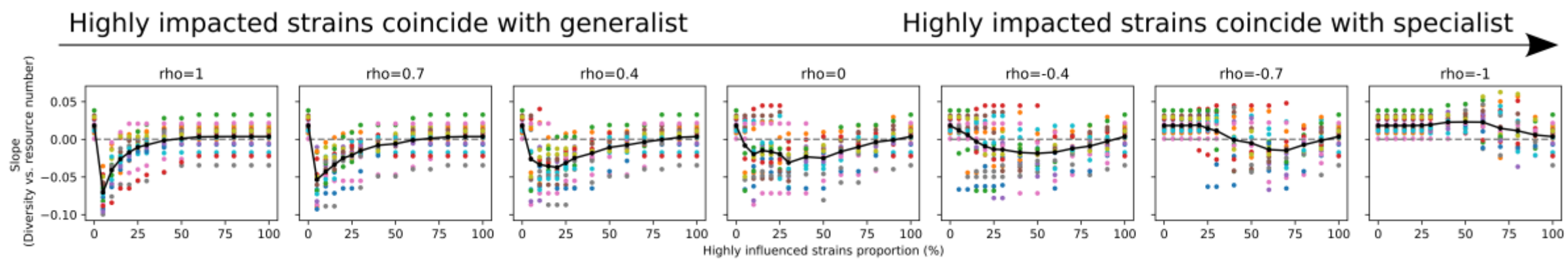


**Supplementary Figure 33: Robustness of microbial diversity-resource complexity slopes when resource complexity impacts highly influenced strain behavior by an alternative mechanism.** We evaluated the impact of substituting the mechanism by which resource complexity affects strain behavior—changing from increasing uptake affinity (i.e. reduced $K_m$) in the main text to increasing resource assimilation efficiency (e) here (Supplementary Text 1 Section 3.4). We set the baseline e_mean to 0.2, and species i's assimilation efficiency of resource α (e_iα) stays constant for mildly influenced strains but gradually increased to 1.0 for highly influenced strains as resource complexity increases (sensitivity γ=1). Each panel displays the slope ($\beta_1$) from a linear model of Shannon diversity vs. resource number (y-axis) plotted against the proportion of highly influenced strains in the community (x-axis, 0–100%). Points represent individual simulated communities (20 replicates), and the solid black trend line indicates the mean across communities. The dashed horizontal line at zero represents the baseline where microbial diversity is neutral to resource complexity. **Trait correlation (ρ):** panels represent different correlations between a strain's niche breadth (the fraction of resources that it can uptake) and its likelihood to be highly influenced by resource complexity, as in Supplementary Figure 30. **Interpretations:** The results demonstrate that a negative diversity-complexity slope emerges robustly even when the influence of resource complexity is mediated through different physiological mechanisms. Similar to the increasing uptake affinity model in the main text, a negative slope is more likely at an intermediate proportion of highly influenced strains and when highly influenced strains partially coincide with resource generalists.

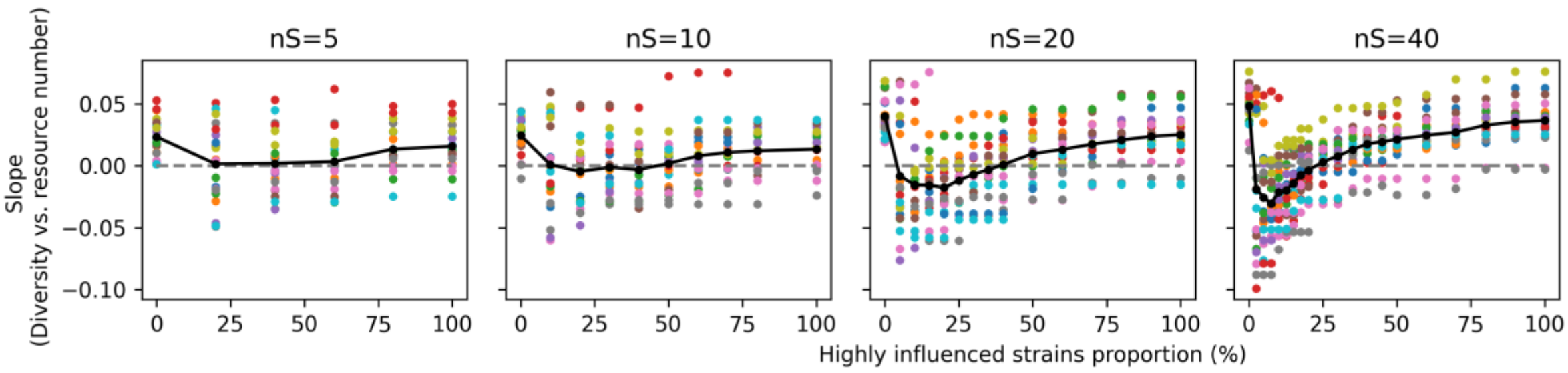


**Supplementary Figure 34: Robustness of microbial diversity-resource complexity slopes across communities with different numbers of initial species (nS).** We evaluated the impact of highly influenced strain behavior on microbial diversity in communities with different number of initial species (nS) within the Consumer-Resource Model (Supplementary Text 1 Section 3.5). Each panel displays the slope ($\beta_1$) from a linear model of Shannon diversity vs. resource number (y-axis) plotted against the proportion of highly influenced strains in the community (x-axis, 0–100%). Points represent individual simulated communities (20 replicates), and the solid black trend line indicates the mean across communities. The dashed horizontal line at zero represents the baseline where microbial diversity is neutral to resource complexity. We used resource assimilation e_mean=0.4 and trait correlation ρ=0.4 as in Supplementary Figure 30 and main text Fig. 4E for these simulations. **Community size (nS):** Panels represent simulations using communities with 5, 10, 20, or 40 initial species respectively (nS=20 is also used for main text Fig. 4E). **Interpretations:** The results demonstrate that the observation of a negative diversity-complexity slope is robust across microbial communities with different number of species. As the community size increases, the slopes tend to be more negative. The likelihood of negative slopes depends on the absolute number of highly impacted strains instead of their proportion, so as communities get bigger, negative slopes occur in a narrower region in terms of the proportion of highly influenced strains.

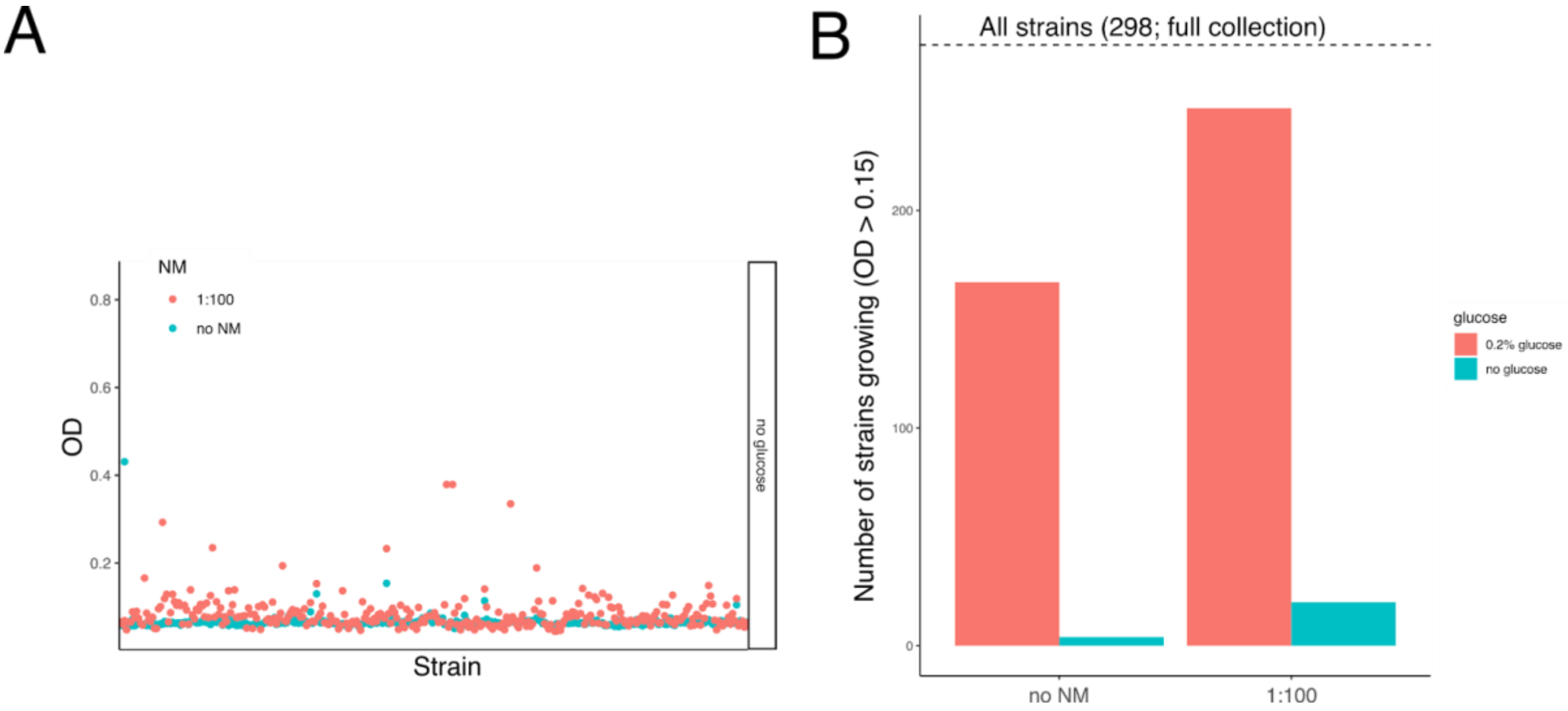


**Supplementary Figure 35: 1:100 dilution of rich medium (NM) promotes synergistic growth by alleviating micronutrient limitations without supplying significant carbon. A.** Growth of 298 individual strains in minimal medium without an added carbon source (M9) with or without supplementation of a 1:100 dilution of nutrient medium (NM). The NM dilution alone, in the absence of glucose, supported only minimal growth. **B.** When 1:100 NM was added together with glucose, the number of strains reaching measurable optical density markedly increased compared with glucose-only conditions. Growth was defined as an optical density at 600 nm ($OD_{600}$) exceeding 0.15—a conservative threshold higher than the maximum OD observed in any control well. The dashed line indicates the total strain collection (all 298 isolates). Measurements were performed after 72 hours of incubation, when cultures had reached carrying capacity.

## Supplementary Tables

**Supplementary Table 1: Sample sizes per condition for Fig. 3c.** Number of independent strain–resource condition observations (n) for each group, defined by the number of carbon sources and strain response category (highly affected vs. not).

| Number of resources | Highly influenced strains | n |
|---|---|---|
| 1 | FALSE | 4158 |
| 1 | TRUE | 2398 |
| 2 | FALSE | 944 |
| 2 | TRUE | 534 |
| 3 | FALSE | 1233 |
| 3 | TRUE | 730 |
| 4 | FALSE | 1213 |
| 4 | TRUE | 725 |
| 5 | FALSE | 1416 |
| 5 | TRUE | 816 |
| 6 | FALSE | 1308 |
| 6 | TRUE | 742 |
| 7 | FALSE | 1548 |
| 7 | TRUE | 928 |
| 8 | FALSE | 1419 |
| 8 | TRUE | 824 |
| 9 | FALSE | 1056 |
| 9 | TRUE | 632 |
| 10 | FALSE | 993 |
| 10 | TRUE | 568 |
| 11 | FALSE | 643 |
| 11 | TRUE | 395 |
| 12 | FALSE | 969 |
| 12 | TRUE | 559 |
| 13 | FALSE | 1057 |
| 13 | TRUE | 625 |
| 14 | FALSE | 952 |
| 14 | TRUE | 560 |
| 15 | FALSE | 1032 |
| 15 | TRUE | 603 |
| 16 | FALSE | 1036 |
| 16 | TRUE | 600 |

**Supplementary Table 2: Statistical analysis for Fig. 4b,c.** Two-sided one-sample Wilcoxon signed-rank tests were performed on log-transformed data (testing deviation from 1). P-values were adjusted for multiple comparisons using the Benjamini–Hochberg method. Direction indicates whether values are significantly higher or lower than the null expectation. Significance levels: ns ($p > 0.05$), * ($p \leq 0.05$), ** ($p \leq 0.01$), *** ($p \leq 0.001$), **** ($p \leq 0.0001$).

**(b) Metabolite depletion analysis**

| Strain category | Number of carbon sources | Median log10 ratio | p-value | Adjusted p-value (BH) | Direction | Significance |
|---|---|---|---|---|---|---|
| Highly affected | 2 | -0.006 | 0.964 | 0.964 | lower | ns |
| Highly affected | 3 | -0.046 | 0.0224 | 0.0336 | lower | * |
| Highly affected | 4 | -0.115 | $1.06 \times 10^{-7}$ | $6.35 \times 10^{-7}$ | lower | **** |
| Mildly affected | 2 | 0.062 | $4.98 \times 10^{-4}$ | $9.96 \times 10^{-4}$ | higher | *** |
| Mildly affected | 3 | -0.079 | $1.52 \times 10^{-4}$ | $4.56 \times 10^{-4}$ | lower | *** |
| Mildly affected | 4 | -0.028 | 0.676 | 0.811 | lower | ns |

**(c) Sentinel strain suppression**

| Strain category | Number of carbon sources | p-value | Adjusted p-value (BH) | Significance |
|---|---|---|---|---|
| Highly affected | 2 | $1.83 \times 10^{-4}$ | $2.75 \times 10^{-4}$ | *** |
| Mildly affected | 2 | 0.826 | 0.826 | ns |
| Highly affected | 3 | $3.23 \times 10^{-10}$ | $6.46 \times 10^{-10}$ | **** |
| Mildly affected | 3 | $6.96 \times 10^{-4}$ | $8.35 \times 10^{-4}$ | *** |
| Highly affected | 4 | $9.68 \times 10^{-23}$ | $5.81 \times 10^{-22}$ | **** |
| Mildly affected | 4 | $5.46 \times 10^{-12}$ | $1.64 \times 10^{-11}$ | **** |

**Supplementary Table 3: Resources used in this study.** A list of carbon compounds categorized by biochemical group, associated metabolic pathway (glycolysis or gluconeogenesis), and vendor information.

| Carbon | Carbon group | Pathway | Vendor |
|---|---|---|---|
| Glucose | Carbohydrate | glycolysis | Sigma-Aldrich |
| Sucrose | Carbohydrate | glycolysis | Sigma-Aldrich |
| Glutamic-acid | Amino-acid | gluconeogenesis | Sigma-Aldrich |
| Glycerol | Sugar-alcohol | both | Sigma-Aldrich |
| Citric acid | Organic-acid | gluconeogenesis | Sigma-Aldrich |
| omnithine | Amino-acid | gluconeogenesis | Sigma-Aldrich |
| Lactose | Carbohydrate | glycolysis | Sigma-Aldrich |
| Maltose | Carbohydrate | glycolysis | Sigma-Aldrich |
| arabinose | Carbohydrate | glycolysis | Sigma-Aldrich |
| Mannose | Carbohydrate | glycolysis | Thermo Fisher Scientific |
| Xylose | Carbohydrate | glycolysis | Sigma-Aldrich |
| Lysine | Amino-acid | gluconeogenesis | Sigma-Aldrich |
| Maleic acid | Organic-acid | gluconeogenesis | Sigma-Aldrich |
| Ribose | Carbohydrate | glycolysis | Sigma-Aldrich |
| Serine | Amino-acid | gluconeogenesis | Merck |
| Proline | Amino-acid | gluconeogenesis | Merck |
| fructose | Carbohydrate | glycolysis | Millipore |
| Threonine | Amino-acid | gluconeogenesis | Merck |
| Valine | Amino-acid | gluconeogenesis | Millipore |
| Histidine | Amino-acid | gluconeogenesis | Merck |
| Aspargine | Amino-acid | gluconeogenesis | Thermo Fisher Scientific |
| Glutumate | Amino-acid | gluconeogenesis | Biowest |

# Supplementary Text

## Supplementary Text 1: Supplementary methods to the Consumer-Resource model and highly influenced strain extensions

## 1 Classic Consumer-Resource Model (Fig. 1B Simulations)

### 1.1 Overview of Consumer and Resource Dynamics.

We consider a community of $n_S$ microbial species competing for $n_R$ resources in a continuous dilution (chemostat-like) environment. Species abundances and resource concentrations change according to the following system of ordinary differential equations:

$$\frac{dN_i}{dt} = \left(\sum_{\alpha=1}^{n_R} e_{i\alpha}\, v_{i\alpha}\right) N_i - \delta N_i, \tag{1}$$

$$\frac{dR_\alpha}{dt} = -\sum_{i=1}^{n_S} v_{i\alpha} N_i + \sum_{i=1}^{n_S}\sum_{\beta=1}^{n_R} b_{i,\beta\to\alpha}\,(1-e_{i\beta})\, v_{i\beta} N_i + \delta\left(R_\alpha^{\text{supp}} - R_\alpha\right). \tag{2}$$

where:

- $N_i$ denotes the biomass of species $i$
- $R_\alpha$ denotes the concentration of resource $\alpha$
- $\delta$ denotes the dilution rate
- $R_\alpha^{\text{supp}}$ denotes a fixed concentration of resource $\alpha$ as supplied in the fresh media

Species grow through uptake of available resources and are removed through dilution. Resources are depleted by consumption, replenished by secretion (cross-feeding), and supplied or washed out by the inflow of fresh medium through dilution. Only a subset of resources is externally supplied.

### 1.2 Resource uptake, biomass conversion, and cross-feeding.

The uptake rate of resource $\alpha$ by species $i$ follows Monod kinetics,

$$v_{i\alpha} = v_{0,i\alpha}\,\frac{R_\alpha}{K_{m,i\alpha} + R_\alpha}, \tag{3}$$

where:

- $v_{0,i\alpha}$ is the maximum uptake rate
- $K_{m,i\alpha}$ is the half-saturation constant, controlling resource affinity

The resources taken up are converted to biomass or other resources/waste. To simplify the equations, we assume that species biomass and resource concentration have been normalized to comparable units, e.g. energy unit, and that the loss of energy when converting resources to biomass is represented by none-usable resources. We further have the following conversion coefficients in equations (1)-(2):

- $e_{i\alpha} \in [0, 1]$ denotes the efficiency with which resource $\alpha$ is converted into biomass by species $i$
- $1 - e_{i\alpha}$ thus represents the cross-feeding rate, or the fraction of resources secreted back into the environment as metabolic byproducts, including waste

- $b_{i,\beta\to\alpha}$ is the secretion matrix and describes the fraction of secreted resource $\beta$ from species $i$ that is converted into resource $\alpha$

For each species $i$ and consumed resource $\beta$,

$$\sum_{\alpha=1}^{n_R} b_{i,\beta\to\alpha} = 1. \tag{4}$$

so that the overall secretion rate is only controlled by $1 - e_{i\beta}$.

### 1.3 Parameter Values

The following table summarizes the parameters used to generate the Consumer-Resource Model communities and their default values or distributions.

| **Parameter** | **Meaning** | **Value / Distribution (Default)** |
|---|---|---|
| *Community and Environmental Parameters* | | |
| $n_S$ | Number of initial species in communities | 20 |
| $n_R$ | Total number of resources | 30 |
| $n_R^{\text{supp}}$ | Number of supplied resources | $\{1, 2, 4, 8, 16\}$ (repeated 3 times) |
| $\delta$ | Dilution rate | 0.1 |
| *Species Trait Parameters* | | |
| $NW_i$ | Niche width (The fraction of resources strain $i$ can uptake) | Uniform distribution $\mathcal{U}(1/n_R, 1)$ |
| $v_{i\alpha}^0$ | Maximum uptake rate | 1 for consumed resources and 0 otherwise. Total number of consumed resource satisfies $\sum_{\alpha=1}^{nR} \mathbb{I}[v_{i\alpha} > 0] = NW_i$. |
| $K_{m,i\alpha}$ | Baseline half-saturation constant | Uniform distribution $\mathcal{U}(0, 1)$ |
| $\bar{e}$ | Mean assimilation efficiency | 0.4 |
| $e_{i\alpha}$ | Assimilation efficiency | $\mathcal{N}(\bar{e}, 0.1^2)$, clipped at 0 |
| $b_{i,\beta\to\alpha}$ | Secretion stoichiometry | $\mathcal{U}(-2, 1)$, clipped at 0 then normalized to 1 for each $i$ and $\beta$ |

## 2 Extended Consumer-Resource Model with Highly Influenced Strains (Fig. 4E Simulations)

The model is extended to incorporate the impact of highly influenced strain behavior, which according to the experimental observation is a mechanism by which resource complexity can benefit some strains more than others. This extension is achieved by introducing a strain-specific sensitivity parameter $\gamma_i$ that modulates strain physiology as a function of the resource complexity in the environment. By default, we assume $\gamma > 0$ allows increasing the resource uptake affinity (decreased $K_{m,i\alpha}$) as resource complexity increases, except in one simulation in section 3.4 where we test an alternative mechanism where resource assimilation rate $e_{i\alpha}$ increases instead.

### 2.1 Strain-Specific Sensitivity to Resource Complexity ($\gamma_i$)

A strain-specific sensitivity parameter, $\gamma_i$, is assigned to each species $i$. By default, strains are classified into two groups: mildly influenced strains (MIS) with $\gamma_i = \gamma_{\text{MIS}}$ and highly influenced strains (HIS) with $\gamma_i = \gamma_{\text{HIS}}$. The binary classification reflects the experimental observation that MIS and HIS form two distinct clusters.

The half-saturation constant is modified from its baseline value $K_{m,i\alpha}$ as in the classic model based on the strain's sensitivity $\gamma_i$ and the *effective* number of supplied resources accessible to species $i$, which serves as a proxy for resource complexity. Rather than using the total number of supplied resources $n_R^{\text{supp}}$, we define a species-specific effective resource complexity

$$n_{R,i}^{\text{eff}} = \sum_{\alpha=1}^{n_R} \mathbb{I}[v_{0,i\alpha} R_\alpha^{\text{supp}} > \varepsilon] \,, \tag{5}$$

where $R_\alpha^{\text{supp}}$ denotes the supply concentration of resource $\alpha$, $v_{0,i\alpha}$ is the maximum uptake rate of species $i$ for resource $\alpha$, $\mathbb{I}[\cdot]$ is the indicator function, and $\varepsilon = 10^{-4}$ is a small positive threshold.

The modification is then applied to get the effective half-saturation constant used in the Monod equation:

$$K_{m,i\alpha}^{\text{eff}} = \frac{K_{m,i\alpha}}{1 + \gamma_i \cdot \max\Big(0,\ n_{R,i}^{\text{eff}} - 1\Big)}. \tag{6}$$

This formulation ensures that $K_{m,i\alpha}^{\text{eff}} = K_{m,i\alpha}$ when only one or less consumable resource is available to species $i$ ($n_{R,i}^{\text{eff}} \leq 1$). For highly influenced strains ($\gamma_i = \gamma_{\text{HIS}} > 0$), increasing effective resource complexity leads to reduced $K_{m,i\alpha}^{\text{eff}}$, corresponding to increased uptake affinity. This mechanism allows highly influenced strains to more efficiently exploit low-concentration resources in complex environments and reflects the experimental observation that such strains can uptake more resources as resource complexity increases.

The resource uptake rate of resource $\alpha$ by species $i$ now becomes

$$v_{i\alpha} = v_{0,i\alpha} \frac{R_\alpha}{\dfrac{K_{m,i\alpha}}{1 + \gamma_i \cdot \max\Big(0,\ n_{R,i}^{\text{eff}} - 1\Big)} + R_\alpha}. \tag{7}$$

and the rest of the equations (1)-(2) stays the same as the classic model.

## 2.2 Assigning Strain-Specific Sensitivity to Resource Complexity ($\gamma_i$)

Sensitivity to resource complexity is conceptually related to resource generalism, and experimental data indicate a weak but nonzero correlation between these traits ($r = 0.36$, Supplementary Figure. 22). To capture this relationship while allowing control of the fraction of highly influenced strains ($f$), we assign species-specific sensitivities to resource complexity ($\gamma_i$) through a two-step procedure. First, we generate a continuous latent variable $\gamma_i^0$ that encodes each species' tendency to be highly influenced, which is correlated with niche width ($NW_i$). Second, we discretize $\gamma_i^0$ into a binary sensitivity parameter by assigning species as mildly or highly influenced, with $\gamma_i = \gamma_{\text{MIS}}$ or $\gamma_i = \gamma_{\text{HIS}}$, respectively, while explicitly controlling the fraction of highly influenced strains in the community.

### 2.2.1 Correlation between niche width ($NW_i$) and tendency to be highly influenced ($\gamma_i^0$).

Let $\mu_{NW}$ and $\sigma_{NW}$ be the mean and standard deviation of niche width $\{NW_i\}$ across all species. We define an intermediate latent variable $\gamma_i^0$ as

$$\gamma_i^0 = \rho_\gamma \frac{NW_i - \mu_{NW}}{\sigma_{NW}} + \sqrt{1 - \rho_\gamma^2}\, \xi_i, \tag{8}$$

where $\rho_\gamma \in [-1, 1]$ controls the correlation between niche width and sensitivity to resource complexity, and $\xi_i \sim \mathcal{N}(0, 1)$ is an independent standard normal random variable. By construction, $\gamma_i^0$ has unit variance and exhibits a Pearson correlation of $\rho_\gamma$ with $NW_i$. Positive values of $\rho_\gamma$ imply that generalist species tend to be more highly influenced by resource complexity, whereas negative values imply that specialists are more highly influenced.

### 2.2.2 Mapping $\gamma_i^0$ to strain sensitivity $\gamma_i$ under different highly influenced strain fractions ($f$).

To reflect the experimental observation that mildly and highly influenced strains form two distinct clusters, we discretize $\gamma_i^0$ into a binary sensitivity parameter $\gamma_i$. Species are first ranked in ascending order of $\gamma_i^0$. For a prescribed fraction $f \in [0, 1]$ of highly influenced strains, we assign

$$n_{\text{HIS}} = \lfloor f \cdot n_S \rfloor, \qquad n_{\text{MIS}} = n_S - n_{\text{HIS}}, \tag{9}$$

where $n_{\text{HIS}}$ and $n_{\text{MIS}}$ denote the number of highly and mildly influenced strains, respectively.

The $n_{\text{MIS}}$ species with the lowest values of $\gamma_i^0$ are assigned a low sensitivity $\gamma_i = \gamma_{\text{MIS}}$, while the remaining $n_{\text{HIS}}$ species are assigned a high sensitivity $\gamma_i = \gamma_{\text{HIS}}$:

$$\gamma_i = \begin{cases} \gamma_{\text{MIS}}, & \text{if } i \in \text{lowest } n_{\text{MIS}} \text{ ranked by } \gamma_i^0, \\ \gamma_{\text{HIS}}, & \text{if } i \in \text{highest } n_{\text{HIS}} \text{ ranked by } \gamma_i^0. \end{cases} \tag{10}$$

This construction allows us to independently tune (i) the correlation between resource generalism and sensitivity to resource complexity ($\rho_\gamma$) and (ii) the fraction of highly influenced strains ($f$), while maintaining a clear separation between mildly and highly influenced strain phenotypes.

## 2.3 Additional Parameter Values

The following table summarizes the new parameters added to the classic model and their default values or distributions.

| Parameter | Meaning | Value / Distribution (Default) |
|---|---|---|
| *Highly Influenced Strain Parameters* | | |
| $f$ | Fraction of highly influenced strains (HIS) | Varied across 14 levels (0 to 1). $f = 0$ corresponds to the classic model. |
| $\rho_\gamma$ | Correlation between $\gamma$ and niche breadth | 0.4 (Experiment value: 0.36) |
| $\gamma_{\text{MIS}}$ | Mildly influenced strain sensitivity to resource complexity | 0 |
| $\gamma_{\text{HIS}}$ | Highly influenced strain sensitivity to resource complexity | 1 |
| $k_\gamma$ | Transition sharpness of $\gamma_i$ from mildly to highly influenced strain | $\infty$ (Step function, binary classification) |

When the fraction of highly influenced strains $f = 0\%$ , all strains are mildly influenced ($\gamma_i = \gamma_{\text{MIS}} = 0$). In this scenario, the uptake affinity $K^{\text{eff}}_{m,i\alpha} \equiv K_{m,i\alpha}$ remains constant regardless of resource complexity, reverting back to the classic Consumer-Resource Model.

# 3 Robustness of the Extended Model to Parameters and Assumptions (Supplementary Figs. Simulations)

To explore the robustness and parameter space of the extended Consumer-Resource Model, a series of simulations were conducted by systematically varying key parameters or model assumptions. The results are shown in Supplementary Figs. 30-34. The default setting for the main text simulations (Fig. 4E) are $n_S = 20$ species, $n_R = 30$ total resources, mean assimilation efficiency $\bar{e} = 0.4$, trait correlation $\rho_\gamma = 0.4$, $\gamma_{\text{MIS}} = 0$, $\gamma_{\text{HIS}} = 1$, a binary classification of mildly vs. highly influenced strains (transition sharpness $k_\gamma = \infty$), and highly influenced strains (HIS) increase their resource uptake affinity (decrease $K_{m,i\alpha}$) as resource complexity ($n_R^{\text{supp}}$) increases. All other simulations in this section are modified upon this default version, and we would describe the changes only.

### 3.1 Supplementary Fig. 30 — Cross-Feeding Rate $(1-\bar{e})$ and Correlation with Generalism $(\rho_\gamma)$

(Data generated by `sim_1219_01.py`)

This simulation explores the combined effects of resource assimilation efficiency (which determines cross-feeding magnitude) and the correlation between a strain's niche breadth (from specialist to generalist) and its tendency to be highly influenced.

- **Cross-feeding $(1-\bar{e})$:** The mean assimilation efficiency $\bar{e}$ was varied across five levels: $\bar{e} \in \{0.2, 0.4, 0.6, 0.8, 1.0\}$. Since the fraction of resource secreted as byproducts is $1-\bar{e}$, this range corresponds to cross-feeding levels from 80% down to 0%.
- **Trait Correlation $(\rho_\gamma)$:** The correlation coefficient $\rho_\gamma$ between niche breadth (number of resources a strain can uptake) and the raw $\gamma$ value was varied across seven levels: $\rho_\gamma \in \{1, 0.7, 0.4, 0, -0.4, -0.7, -1\}$.
  - $\rho_\gamma > 0$: Generalist strains are more likely to be HIS.
  - $\rho_\gamma < 0$: Specialist strains are more likely to be HIS.
  - $\rho_\gamma = 0$: No correlation between niche breadth and HIS status.

### 3.2 Supplementary Fig. 31 — Sensitivity Level of Highly Influenced Strains $(\gamma_{\mathbf{HIS}})$

(Data generated by `sim_1219_04.py`)

This simulation explores the effect of varying the magnitude of the HIS strain's sensitivity to resource complexity.

- **HIS Sensitivity $(\gamma_{\mathbf{HIS}})$:** The sensitivity parameter for highly influenced strains was varied across five levels: $\gamma_{\text{HIS}} \in \{0.2, 0.5, 1, 2, 4\}$. $\gamma_{\text{MIS}}$ was fixed at 0.

### 3.3 Supplementary Fig. 32 — Continuous Transition of the Sensitivity to Resource Complexity $(\gamma_i)$ with Different Transition Sharpness $(k_\gamma)$

(Data generated by `sim_1219_02.py`)

This simulation explores whether the microbial diversity–resource complexity results depend on treating strain sensitivity to resource complexity as a strictly binary trait (mildly vs. highly influenced) or as a continuous trait. As in the main simulations, we first generate a raw latent sensitivity value $\gamma_i^0$ that is correlated with niche breadth ($\rho_\gamma = 0.4$), then map $\gamma_i^0$ into the final sensitivity $\gamma_i \in [\gamma_{\text{MIS}}, \gamma_{\text{HIS}}]$ using a cubic smoothstep transformation whose steepness is controlled by a transition-sharpness parameter $k_\gamma$.

- **Transition sharpness $(k_\gamma)$:** Determines how abruptly strain sensitivity to resource complexity $(\gamma_i)$ transitions from mildly to highly influenced phenotypes. We vary it across six values, $k_\gamma \in \{0.4, 0.8, 1.6, 3, 6, 12\}$, spanning a smooth continuum to a near-binary classification, as detailed below.

**Extending highly influenced strain fraction $f$ to smooth transition middle point $x_0$.** For each community and each prescribed highly influenced fraction $f$, we sort species in ascending order of $\gamma_i^0$, define as in the main simulations

$$n_{\text{HIS}} = \lfloor f \cdot n_S \rfloor, \qquad n_{\text{MIS}} = n_S - n_{\text{HIS}}, \tag{11}$$

and place the transition midpoint $x_0$ at the cutoff between the two groups:

$$x_0 = \begin{cases} \gamma^0_{(n_S)} + \left(\gamma^0_{(n_S)} - \gamma^0_{(n_S-1)}\right), & n_{\text{HIS}} = 0, \\ \gamma^0_{(1)} - \left(\gamma^0_{(2)} - \gamma^0_{(1)}\right), & n_{\text{MIS}} = 0, \\ \dfrac{\gamma^0_{(n_{\text{MIS}})} + \gamma^0_{(n_{\text{MIS}}+1)}}{2}, & \text{otherwise}, \end{cases} \tag{12}$$

where $\gamma^0_{(m)}$ denotes the $m$-th order statistic of $\{\gamma^0_i\}$ (sorted values). The endpoint sensitivities were fixed at $\gamma_{\text{MIS}} = 0$ and $\gamma_{\text{HIS}} = 1$.

**Smoothstep mapping from $\gamma^0_i$ to $\gamma_i$.** To interpolate smoothly between mildly and highly influenced strain sensitivities, we use the standard cubic smoothstep function,

$$S(x) = 3x^2 - 2x^3, \qquad x \in [0, 1], \tag{13}$$

which transitions monotonically from 0 to 1 with zero slope at both endpoints. This function allows a continuous but bounded interpolation between $\gamma_{\text{MIS}}$ and $\gamma_{\text{HIS}}$.

To apply the smoothstep to the raw latent sensitivities $\gamma^0_i$, we first linearly rescale them into a normalized variable $x_{\text{norm},i} \in [0, 1]$ within a finite transition window centered at the cutoff $x_0$. The width of this window is chosen so that the maximum slope of the resulting $\gamma_i(\gamma^0_i)$ curve equals a prescribed sharpness parameter $k_\gamma$. Because the cubic smoothstep has a maximum slope of 1.5 on $[0, 1]$, scaling slope to $k_\gamma$ and height to $\gamma_{\text{HIS}} - \gamma_{\text{MIS}}$ requires a transition width

$$w = \frac{1.5\,(\gamma_{\text{HIS}} - \gamma_{\text{MIS}})}{k_\gamma}. \tag{14}$$

We then define the normalized coordinate (clamped in $[0, 1]$)

$$x_{\text{norm},i} = \text{clip}\left(\frac{\gamma^0_i - x_0}{w} + \frac{1}{2},\, 0,\, 1\right), \tag{15}$$

so that $x_{\text{norm},i} = 0$ and 1 correspond to the lower and upper plateaus, respectively, and $x_{\text{norm},i} = 1/2$ corresponds to the transition midpoint.

Finally, the strain-specific sensitivity to resource complexity is given by

$$\gamma_i = \gamma_{\text{MIS}} + (\gamma_{\text{HIS}} - \gamma_{\text{MIS}})\, S(x_{\text{norm},i}). \tag{16}$$

**Transition sharpness and binary limit.** This smoothstep mapping yields $\gamma_i \approx \gamma_{\text{MIS}}$ for species well below the cutoff, $\gamma_i \approx \gamma_{\text{HIS}}$ for species well above the cutoff, and a continuous transition whose steepness increases with $k_\gamma$, as visualized in Supplementary Fig. 32.

Thus, increasing $k_\gamma$ shifts the sensitivity ($\gamma$) distribution from a smooth continuum (low $k_\gamma$) to a near-binary distribution (high $k_\gamma$); the binary classification mildly vs. highly influenced strain used in main simulations corresponds to the limiting case of an ideal step function ($k_\gamma = +\infty$).

### 3.4 Supplementary Fig. 33 — Alternative Physiological Mechanism for Highly Influenced Behavior

(Data generated by `sim_1219_05.py`)

This simulation tests an alternative physiological mechanism where resource complexity increases mean resource assimilation efficiency $\bar{e}$ instead of increasing uptake affinity (reducing $K_{m,i\alpha}$). Same as in the main simulations, resource complexity is quantified using a effective resource complexity to species $i$: $n^{\text{eff}}_{R,i}$ as defined in equation (5).

The assimilation efficiency is then modified as a function of effective resource complexity:

$$e^{\text{eff}}_{i\alpha} = \min\left(1,\; e_{i\alpha} + \gamma_i \frac{\max\big(0,\; n^{\text{eff}}_{R,i} - 1\big)}{n_R}\right), \tag{17}$$

where $e_{i\alpha}$ is the baseline efficiency under the single-resource condition (sampled given $\bar{e} = 0.2$), $\gamma_i$ is the species-specific sensitivity to resource complexity, and $n_R$ is the total number of resources. The upper bound of 1 enforces the physical constraint that assimilation efficiency cannot exceed complete assimilation (i.e., zero cross-feeding).

This formulation ensures that $e^{\text{eff}}_{i\alpha} = e_{i\alpha}$ when only one or less consumable resource is available to species $i$ ($n^{\text{eff}}_{R,i} \leq 1$). Mildly influenced strains ($\gamma_i = 0$) therefore maintain constant assimilation efficiency, whereas

highly influenced strains ($\gamma_i > 0$) exhibit increased assimilation efficiency as the number of consumable supplied resources increases.

Finally, in all simulations using this alternative mechanism, $e_{i\alpha}$ is replaced by $e^{\text{eff}}_{i\alpha}$ in equations (1)-(2), and uptake affinity was kept fixed by setting $K^{\text{eff}}_{m,i\alpha} = K_{m,i\alpha}$, equivalent to substituting equation (4) instead of equation (7) into equations (1)-(2).

### 3.5 Supplementary Fig. 34 — Number of Initial Species in Communities ($n_S$)

(Data generated by `sim_1219_03.py`)

This simulation examines the impact of the community size ($n_S$, initial number of species in the community) on the observed microbial diversity-resource complexity relationship. We vary the community size across four levels: $n_S \in \{5, 10, 20, 40\}$, and adjust the highly influenced strain fractions $f$ accordingly to ensure a representative sampling. All other parameters are kept at their default values.

**Supplementary Text 2: Inferring interactions matrices from experimental data using the generalized Lotka-Volterra model**

# 1 Data and Methodology

Data from single-species growth, pairwise interactions (Fig. 2 A) and community composition (Fig. 1 D, E and Supplementary Fig. 3) at steady state was used to infer the interaction matrices of the 14 strains comprising the experimental communities. The dataset was divided into three groups based on carbon source richness: 1, 8, and 16 resources. Each group contained multiple nutrient combinations, so we did not assume a fixed interaction strength for a given strain pair across conditions. Instead, we treated interaction strengths as random variables drawn from an unknown distribution and used Bayesian regression to infer their values.

# 2 Generalized Lotka–Volterra Model at Steady State

With the generalized Lotka–Volterra (gLV) model we obtain under steady-state assumptions:

$$\frac{dx}{dt} = k \circ x \circ (1 - Ax) = 0, \tag{1}$$

where $x$ is an $N$-dimensional vector containing steady-state population densities of N interacting species, $A$ is the $N \times N$ interaction matrix, k a N-dimensional vector of growth rates, and $\circ$ the Hadamard product (element-wise multiplication). We take into account only the species that are present in the community in steady state ($x_i > 0$). For these species the equation (1) can only become zero if $(1 - Ax)$ equals zero. Accordingly, equation (1) simplifies to:

$$\mathbf{1}_{\{x_i>0\}} \circ (1 - Ax) = 0, \tag{2}$$

with $\mathbf{1}_{\{x_i>0\}}$ denoting a N-dimensional indicator vector, that has entry 1 if the species i is present at steady state and 0 otherwise.

# 3 Bayesian Regression and MCMC Implementation

Equation (2) was fit using Bayesian regression with Monte Carlo Markov Chain (MCMC) sampling, implemented via the NUTS solver in the Python `pyro` package.

## 3.1 Priors and Likelihood

Priors for the off-diagonal elements ($\alpha_{\text{other}}$) of the interaction matrix A were assumed to be normally distributed with mean 1 and variance 1. Priors for the diagonal elements ($\alpha_{\text{self}}$) of the interaction matrix A were normally distributed with mean equal to the inverse of the carrying capacity (i.e., the steady-state $OD_{600}$ in monoculture, Supplementary Fig. 7) and variance $5 \times 10^{-6}$. With

$$y = \mathbf{1}_{\{x_i>0\}} \circ (1 - Ax) \tag{3}$$

the likelihood for the Bayesian regression was defined as:

$$\mathcal{L}(y|x, A) \sim \mathcal{N}(0, 10^{-5}\mathbf{I}). \tag{4}$$

## 3.2 Posterior Sampling

Posterior sampling was performed using 30 chains, each with 400 warm-up steps and 200 sampling steps. Convergence was assessed by plotting posterior traces over time, and the final interaction matrix values were computed as the mean of the posterior samples. The software code for the Bayesian Regression can be accessed at https://github.com/RatzkeLab .
