# Supplementary material for: Increased nutrient diversity can induce loss of microbial diversity through enhanced resource uptake
Source: Nat Ecol Evol. 2026 Jul 20;10(8):1449–63. doi: 10.1038/s41559-026-03111-4 (PMC13441979; doi:10.1038/s41559-026-03111-4)
Supplement: Supplementary file 4 [file 41559_2026_3111_MOESM3_ESM.pdf]

nature portfolio

Peer Review File

Increased nutrient diversity can induce loss of microbial diversity through enhanced resource uptake

Corresponding Author: Dr Christoph Ratzke

Version 0:

Decision Letter:

10th October 2025

*Please ensure you delete the link to your author homepage in this e-mail if you wish to forward it to your co-authors.

Dear Dr Ratzke,

Thank you for your patience and please accept my apologies again for the delay. Your Article, "Emergent loss of microbial biodiversity with increasing resource diversity" has now been seen by 3 reviewers. You will see from their comments copied below that while they find your work of considerable potential interest, they have raised quite substantial concerns that must be addressed. In light of these comments, we cannot accept the manuscript for publication, but would be very interested in considering a revised version that addresses these serious concerns.

We hope you will find the reviewers' comments useful as you decide how to proceed. If you wish to submit a substantially revised manuscript, please bear in mind that we will be reluctant to approach the reviewers again in the absence of major revisions and, if we do go back to the reviewers, we will need them to be much more convinced at the next stage to consider the manuscript further.

If you choose to revise your manuscript taking into account all reviewer and editor comments, please highlight all changes in the manuscript text file in Microsoft Word format.

We are committed to providing a fair and constructive peer-review process. Please do not hesitate to contact us if there are specific requests from the reviewers that you believe are technically impossible or unlikely to yield a meaningful outcome.

If revising your manuscript:

* Include a “Response to reviewers” document detailing, point-by-point, how you addressed each referee comment. If no action was taken to address a point, you must provide a compelling argument. This response will be sent back to the referees along with the revised manuscript.

* If you have not done so already we suggest that you begin to revise your manuscript so that it conforms to our Article format instructions at http://www.nature.com/natecolevol/info/final-submission. Refer also to any guidelines provided in this letter.

* Please note that our Articles can have up to 6 display items so you could consider additional figures to avoid showing too much in the ones you currently have.

* Include a revised version of any required reporting checklist. It will be available to referees (and, potentially, statisticians) to aid in their evaluation if the manuscript goes back for peer review. A revised checklist is essential for re-review of the paper.

Please use the link below to submit a revised paper:

Link Redacted

Note: This URL links to your confidential home page and associated information about manuscripts you may have submitted, or that you are reviewing for us. If you wish to forward this email to co-authors, please delete the link to your homepage.

If you wish to submit a suitably revised manuscript we would hope to receive it within 6 months. If you cannot send it within

this time, please let us know. We will be happy to consider your revision so long as nothing similar has been accepted for publication at Nature Ecology & Evolution or published elsewhere.

Nature Ecology & Evolution is committed to improving transparency in authorship. As part of our efforts in this direction, we are now requesting that all authors identified as ‘corresponding author’ on published papers create and link their Open Researcher and Contributor Identifier (ORCID) with their account on the Manuscript Tracking System (MTS), prior to acceptance. This applies to primary research papers only. ORCID helps the scientific community achieve unambiguous attribution of all scholarly contributions. You can create and link your ORCID from the home page of the MTS by clicking on ‘Modify my Springer Nature account’. For more information please visit please visit www.springernature.com/orcid.

Please do not hesitate to contact me if you have any questions or would like to discuss the required revisions further.

Thank you for the opportunity to review your work.

[redacted]

Reviewer expertise:

Reviewer #1: Microbial ecology and evolution, ecological theory

Reviewer #2: Microbial ecology, evolution, metabolism

Reviewer #3: Microbial ecology and physiology, bioinformatics

Reviewers’ comments:

Reviewer #1 (Remarks to the Author):

In this manuscript, Shalev et al. present the hypothesis that increases in resource diversity do not always lead to an increase in biodiversity, but instead, counterintuitively, can lead to a decrease in diversity. They show this effect in data from the Earth Microbiome Project (EMP), synthetic communities (SynComs), and attempt to explain it using pairwise interaction and metabolomics experiments. The idea overall is interesting and timely, and the amount of data generated for this project is impressive. Nevertheless, I am not entirely convinced by their conclusions which run counter to intuition and previous experiments, and therefore in my view require clearer evidence and a more careful consideration of the existing literature. Below, I provide several comments about the EMP data analysis, in vitro experiments, and the different models used here. Because of the number of different approaches, I have grouped my comments not by major and minor, but by approach.

Discussion of existing literature:
Regardless of the validity of the results of this manuscript (discussed below), more work is required to put the present work in context with existing literature. As the authors mention, there are few quantitative studies of the effect of increasing resource diversity on species diversity. This makes it even more surprising that, even though the two experiments that previously asked similar questions are cited, this manuscript does not engage with those studies at all. In particular, ref 13 has been quite influential in the field and is certainly well-known to the authors. The results in Ref 13 go against the findings of the present manuscript, but those findings are explained quantitatively by a physiology-based model. Why is Ref 13 not discussed at all? Why is the physiology-based model from Ref 13 not applicable here? The mismatch between Ref 13, Ref 14, and the data presented here needs to be discussed in detail. Perhaps the data from those papers could even be reanalyzed through the lens of the present paper? Since the supplementary data was not available during review, I do not know the taxonomy of the strains used here, but perhaps there is some phylogenetic relatedness between these strains and those in Ref 13/14 that could be used to infer the degree to which the classification into highly/mildly affected strains can be useful there, as well?

EMP data: An analysis of EMP data appears to show that for many communities, there is a negative correlation between the observed number of metabolites and the Shannon index. The authors interpret this as increased competition and competitive exclusion at higher resource diversity, rather than the intuitively expected increase in diversity at higher resource diversity due to the greater number of available niches.

1. I am not convinced that the results from the EMP and the SynCom experiments in this paper can be interpreted together as they are here; in particular, whether the diversity of metabolites in the EMP and the number of input resources in the SynCom experiments can be interpreted at all as having anything in common. In the EMP, the number of metabolites is the result of microbial activity, both production and uptake. The observed metabolites are those that have been produced and not yet consumed; thus, a sample with a low measured metabolite number might not only arise because the diversity of the incoming resources is low but also because it represents a highly active microbial ecosystem where many metabolites are

rapidly consumed; this idea of “missing metabolites” being key in predicting community metabolism was recently used to predict the trophic structure of gut microbiomes (10.1371/journal.pcbi.1007524). For this reason, I find the observation that the correlation between resource diversity and species diversity to be tenuous and potentially explained by a variety of other factors.

2. The GC-MS data in Fig 4 and the discussion around it brings up additional questions about the interpretation of the EMP data: in Fig 4, higher input resource number leads to lower concentration of metabolites (whether the number of metabolites is lower is hard to say, but once concentrations become low enough, they might conceivably be measured as absent). If the authors are correct about the physiological findings (see below), shouldn't the prediction then be that those EMP communities that show low metabolite counts might have arisen from environments with many input resources? This might at least happen in some environments (and perhaps not in others, depending on the input resources and species present). For me, this makes it even more difficult for me to interpret the EMP results.

3. Regardless of the interpretation of the number of observed metabolites, I found the statistical effect in the EMP data to be rather mild, and it seems like generally diversity increases with the number of metabolites (“resources”). In SI Fig 1, after multiple testing correction, only a single ecosystem type (Animal distal gut, non-saline) shows a significant negative correlation, hardly a huge effect. By contrast, the four strongest correlations are all positive.

4. Fig 3E: after identifying highly influenced strains (HIS), the authors map EMP reads to phylogenetically related strains and find that only those strains have a positive correlation with the number of measured metabolites. However, I think a crucial point of this analysis that is currently missing is the potential bias of finding these strains across the different ecosystems, which additionally might each have their own biases for having a high/low number of metabolites and its correlation with diversity. Without a more careful analysis, it is unclear to me that this analysis proves the “ecological relevance” of the experimental results (L245). Additionally, the HIS seem to be at much lower average relative abundance than the MIS, which goes against the experimental finding that the HIS dominate the communities.

Experimental community data: The authors aim to reproduce the trends observed in the EMP by assembling SynComs from 6 or 8 species across a variety of different resources and resource combinations.

1. In Fig 1E, the Shannon index shows some bias towards negative correlations with the resource number. I believe some of this bias may come from the fact that many communities maintain a lot of initial diversity even after 9 transfers (which in itself I found surprising). For example, even at a single resource, the 6-species SynComs still contain on average about 5 species. Translated to the Shannon index, a simple calculation shows that for a random community with uniformly distributed relative abundances, one would expect a Shannon index of 1.6 and 1.9 for 6 and 8 strain SynComs, respectively. Some communities seem to be close to that threshold even for a single resource (e.g., 5, 3), such that they could not possibly have a high positive slope with resource number, while negative slopes are comparatively less constrained.

2. In some cases, the richness is greater than the number of input strains. What are those additional strains, contaminations?

3. In terms of richness, most communities seem to show no correlation with the number of resources. In addition to my point 1 above, while there does seem to be an effect in the Shannon index, this lack of correlation in richness vs resource diversity seems to imply that the most significant change is that a small number of strains are more dominant at higher resource numbers, rather than competitive exclusion, as the authors describe it.

In summary, I am not so convinced that a “large fraction” (L78) of communities showed a diversity decrease, or that there is “widespread biodiversity loss” (L85); those sound like overstatements to me that rely on choosing Shannon diversity over richness, and are potentially confounded by the issues raised above.

Interactions and physiology: The authors aim to explain their SynCom findings with an impressive number of interaction and metabolomics measurements. They find lower self-inhibition, more resource consumptions, and lower metabolite excretion at higher resource number, which increases the proportion of negative interactions.

1. As the authors state, a lower self-inhibition at higher resource number, i.e., a high carrying capacity, is a counterintuitive result – I would expect not all strains to consume all the resources. To arrive at the self-inhibition values, are the carrying capacities averaged over several carbon sources? (This seems to be described in SD3, but this was not available at the time of review – still, this should be described in the text somewhere.) Surely, the carrying capacity is different for each carbon source, including zero in some cases? Given the counterintuitive nature of this result, it needs to be made much clearer that it is not an artefact of the methodology.

2. Minor point: this manuscript likely will be read by many microbiologists, and explicitly providing values of OD for the carrying capacity may make the manuscript more accessible to that audience.

3. SI Fig 10 shows that the strain 79 that drives biodiversity loss with resource number is a clear exception – all other strains appear to be neutral or have a positive impact on biodiversity. To me, this suggest that while there is clearly an effect with this strain, it may be a physiological anomaly; perhaps this strain has a very strong effect on pH or produces antibiotics and

ALSO happens to dominate at high resource numbers (perhaps because it is very metabolically flexible and can consume every resource rapidly). It is therefore perhaps not a coincidence that strain 79 also has by far the largest absolute values of impact factors (SI Fig 11).

4. Sentinel experiments, Fig 3B: It is interesting to know what the strains and resources are that give rise to strong facilitation and strong suppression. This information is contained in SI Fig 17, where it looks like the strongly facilitative (not "facultative") strains are facilitative in sugars and suppressive in acids. In the absence of information about the strains' taxonomy, this is reminiscent of patterns observed between enterobacteria (E) and pseudomonadales (P, like the sentinel strain used here), where E produce organic acids on sugars that P prefers over sugars. Thus, I wonder whether the choice of sentinel strain is crucial here in determining the impact of different strains. Would the results have looked different if the sentinel strain was, say, an enterobacterales strain?

5. There is no discussion for why or how some strains might be highly or mildly influenced by resource number. Overall, the fact that the classification as a highly or mildly influenced strain has such a strong phylogenetic correlation suggests to me that there may be a clear physiological idiosyncrasy that explains the highly or mildly influenced classification, rather than the classification being an emergent feature that can appear across many clades. The data in Fig 3B would offer a possible physiological explanation for the increased suppression at higher resource number that differs from the authors' interpretation: as the number of resources increases, the proportion of resources that allow for strong facilitation decreases (e.g., among the 8 resources, there might only be 4 fermentable sugars). At the same time, there is always competition for the organic and amino acids, leading to overall more suppression at higher resource diversity. Such an explanation would be satisfying if correct and inject some much needed physiological mechanism into the present findings, which I find currently lacking (e.g., in L232, the loss of facilitation is mentioned but no potential mechanism is offered).

6. In addition, the data shows that interactions (unsurprisingly) are context-dependent and can be positive or negative depending on the carbon source. If my understanding is correct that the reported interaction terms in Fig 2 are averaged over multiple carbon sources, this averaging does not seem to be doing the complexity of the dynamics justice and may even be misleading. If I am misunderstanding, then please explain the methodology more clearly.

7. L275ff: I found the idea of mixing supernatants from individual resources and comparing to supernatants from mixed resources to be very elegant. However, I found the discussion around this point to be confusing and initially found myself wondering whether lower residual key nutrients in the mixed-resource supernatants really imply "increased carbon uptake with greater resource diversity" – on a first read, wouldn't this be equally well explained by reduced excretion of metabolites? SI Fig 19 resolves this somewhat, but it would be helpful to know which are the "supplied" metabolites in each case. For the produced metabolites, it seems that only in the 4-resource condition and with the HIS is there any difference in metabolites production. Therefore, it would be useful to split the discussion into supplied and produced metabolites. E.g., in the caption of SI Fig 19, the authors talk of "more metabolites remaining in the non-supplied category", but they cannot be remaining if they were produced during the growth (unless I am misunderstanding). Nevertheless, the finding that the sentinel strain grew less in the high resource supernatants is interesting and aligns with the reduction in metabolite concentrations.

Modeling:

1. The authors use both Lotka-Volterra models and Consumer-resource models here. Although I do see the practical usefulness of LV to interpret coculture outcomes, I find it confusing and potentially even inappropriate to make such heavy use of a model that does not explicitly model resource diversity when resource diversity is the entire point of this manuscript. With all the data they collected, why not try to fit consumer-resource models to the individual and pairwise cocultures? I feel that measuring metabolic capabilities and interpreting their data in light of niche overlaps could be much more mechanistic and accessible.

2. Both the validity and the procedure of fitting the LV model to infer interaction parameters are unclear to me. It makes sense that inverse carrying capacities would represent the a_self and that one could infer the a_other from the pairwise experiments. But several questions remained for me:
a. Is it appropriate to also use the community data, where it is well-known that higher-order effects and dynamic interactions can be hugely important in more complex communities? How are these community data even used during inference?
b. Are the inferred interaction terms for a given pair and condition consistent between the pairwise and community inferences? Are they consistent across individual resources in the single-resource cocultures?
c. Is inferring interactions using Bayesian regression as done here statistically as well-supported as established methods? Why didn't the authors use an established method to infer interactions from the community data, such as SparCC or SpiecEasi? To me, the Bayesian regression procedure used here seems like a new method that requires substantial testing against, e.g., simulated controls.
d. How should a_other values of >10 be interpreted? That seems extremely high to me - could they be artefactual because some carrying capacities are extremely low?
e. In SI Fig 7, it is stated that cases with relative abundance <0.001 were excluded, which is understandable from a technical perspective, but in reality, does this means that all cases of actual competitive exclusion (where one strain goes to extinction) are removed from consideration here? If that understanding is correct, doesn't this introduce a lot of bias?

3. In the LV model simulations, all growth rates are set to 1. However, about 10% of alpha_other are greater than alpha_self, and in those cases the growth rate CAN matter (bistability regime). This should be mentioned, or growth rates should be

randomized.

4. In general, I was surprised to see the off-diagonal elements quite large compared to the diagonals, meaning that interspecies interactions are very strong even at high resource concentrations. It would be good to comment on this result more, especially since the coexistence of many species in the SynComs would suggest small average values of alpha_other.

5. L130: while the interaction matrix inferred for higher resource number reproduces a lower diversity than the one for a lower resource number, this is not evidence that the inferred interactions are valid, as the authors claim (L130-131). It simply means that a higher ratio of other to self interaction strength yields lower diversity outcomes, which is well known.

6. The CRM setup has a number of problems:
a. Adding growth rates from individual carbon sources should not be done as is done here; instead, there are two options: the more "traditional" model simply adds the growth rates without cutting off at a maximum growth rate; alternatively, there is a formula for adding growth rates based on proteome allocation in 10.15252/msb.20145537 (extended to more than two resources, e.g., in 10.1101/2024.06.14.599039).
b. More importantly, the proposed mechanism for the updated CRM in SI text 3 needs to be discussed in more detail. In particular, what possible mechanism would give a decreased Km? Typically, one would assume the Km for a given resource to be fixed and independent of the presence of other resources. A case could be made for reduced carbon catabolite repression when there are several resources around, each with now a lower concentration and therefore weaker potential for CCR, but this would need to be discussed and supported by existing literature.
c. Alternatively, could other mechanisms rather than a decrease in Km explain the results? This would need to be discussed, as well.
d. In the resource dynamics, the c_ia term is not explained completely: what is a_i and what is the meaning of the min(a_i, r_0,i)/a_i term?
e. In eq. 4 in Supp text 3, c_ia is updated with the new k_m expression, but only in the metabolite excretion term, not the uptake term. Side note: it would probably be easier to read if equation numbering was continuous across the supp texts.

7. Fig 4E: why is the slope of diversity vs resource number so noisy overall? It also looks like for up to 50% highly influenced strains, one still gets an increase in diversity, contradicting the experiments. The case of 5% HIS seems to give a particularly strong reduction in slope, but given the overall shape of the curve, this seems artefactual without an explanation.

Minor comments:
- Fig 1: How many species were the simulations initialized with? What are the simulation parameters?
Supplementary data was not available during review but overall, I believe adding some more information to the main text without having to go the SD for every bit of information would be very helpful for readers: how many strains were combined to form the synthetic communities (only in caption so far), what is the phylogeny of the strains (missing), what resources were used for the metabolomics experiments, etc.?

- L43: the consumer resource model in reference 7 describes animal and plant communities, no microbial communities. More recent references to the microbial CRM would be more appropriate. Crucially, as the authors know, crossfeeding plays a huge role but is missing from the list of additional mechanisms (L47) even though it leads to a diversity of dynamically generated resources.

- The authors may be interested in this recent preprint that seems to show some similar findings on interactions in multiple resources: https://www.biorxiv.org/content/10.1101/2025.03.30.646244v1.abstract

- L228: what does it mean to have a "strongly quantitative" response?

- L358 (Discussion): While it is true that nutrient fertilization can reduce diversity, I found this discussion odd. Firstly, because, as the authors say, this is typically because of the increase in concentration not diversity, and it is not clear that the two scenarios are at all related. Second, their examples are not related to microbes, while their entire approach here and the physiological explanations rely on microbial physiology. Therefore, I would caution against trying to forge too close a link between macroscopic ecosystems and microbial ecology in this context.

- Typo: SI Fig 17 caption, 3D -> 3B

- L383: A personal point, but I find the framing of discussions of microbial existence as a "paradox" despite "competition for the same resources" to be outdated – as the authors themselves say, there are so many theoretical explanations for any number of species coexisting on small numbers of supplied resources that, at least to me, there simply is no paradox. However, this does not invalidate the search for patterns in diversity across environments and trying to explain them, including the work in this manuscript, at all – it is simply a matter of framing.

Reviewer #1 (Remarks on code availability):

Code is available, extensive, and well-annotated. However, I did not try to run it.

Reviewer #2 (Remarks to the Author):

This manuscript presents an ambitious combination of modeling, multi-omics, and experimental approaches to study microbial community assembly. The integration of these methodologies is a strength, the data is rich and could potentially yield significant insights. However, I find that the work does not fully substantiate its claims. Specifically, the modeling is underdeveloped, the experimental methods lack critical detail and justification, and while concision is a strength, important theoretical and empirical literature is not discussed.
Below is a list (not exhaustive, but I believe sufficient) of major issues I have identified with this manuscript at its current stage.
Major points
The consumer–resource (CR) framework used here is not developed in sufficient depth. CR models incorporating cross-feeding are now considered state of the art in microbial ecology, yet cross-feeding is not included or discussed.
More broadly, cross-feeding is widely recognized as central to microbial community assembly, yet it is not even mentioned in the manuscript.
Modeling results are not fully unpacked. For instance, the saturating curve in the CR model is presented without explanation.
Recent theoretical developments which might be highly relevant to this work are not or barely discussed (e.g. Cui, Marsland and Mehta PRL 2020; dal Bello 2020 Nat Eco Evo). The discussion of the latter paper in particular, which studied nearly the same question, is minimal. Especially given that the conclusions of that paper differ quite a bit from what is claimed here, yet that contrast is not explored.
Many important parts of the experimental design and approach are either absent, or only concisely specified. Where do the strains come from, why are they suitable for answering this question, how were the 22 resources selected?
The choice to maintain all resources at 0.2% mass/volume is problematic. Different resources differ in molecular weight and carbon content, meaning mixtures vary substantially in total available carbon and per-resource contribution. This affects carrying capacities, which are fundamental for interpreting both monoculture and co-culture dynamics (e.g. $\alpha\_self = 1/K$). The relationship between carrying capacities and interaction coefficients is not addressed.
The computation of the "impact factor" is unclear. The text provides one definition ($2\times\alpha\_self + 2\times\alpha\_others$), while Figure 2 states a different definition based on OD600 values. Line 155 refers to "Methods," but this is absent from the Methods section.
Metabolic strategies, e.g. diauxie/coutilization and resource preferences, should be central to the observations, yet they are poorly discussed. In particular, there are known metabolic determinants of whether resources are sequentially or simultaneously uptaken (e.g. see Wang et al Nat communications 2019). In addition, potential biases derived from using a Pseudomonas sentinel strain are not discussed.
Fig S4 says "As resource number increases, interaction coefficients decrease for both intra-species (diagonal) and inter-species interactions (off-diagonal)" However, whether the figure supports this conclusion is hard to tell visually.
Discussion: "our finding reopens a fundamental question: how is microbial coexistence maintained despite competition for the same resources?" Interesting way to frame it. I think it is hard to reopen a question that was never closed.

Reviewer #3 (Remarks to the Author):

In this manuscript, Shalev et al. use a synthetic microbial community-based experiment to test the hypothesis that diversity increases with resource diversity. They first observe that, based on the Earth Microbiome Project (EMP) data, the assumption that increasing species diversity is associated with resource diversity does not appear to hold true. They then design a laboratory experiment to manipulate resource diversity and species diversity, testing the assumptions of consumer-resource theory. Overall, they find the opposite of what is expected – that species diversity decreases with resource diversity. Specifically, they identified bacterial isolates that exemplified this pattern (highly influential). They conclude that these basic theoretical models fail to capture the specific physiology of microbial systems and that there is limited support for these classical ecological principles in microbial systems.

Overall, I was highly impressed with the experimental aspects of this work. The researchers designed an impressive experiment using liquid handling reports to achieve a remarkable number of manipulations. However, I was not convinced by their main conclusions and was surprised that they did not consider the role of resource generalists in their interpretations. Throughout the manuscript, I felt that there was an incredible lack of information necessary to interpret the results, and instead, important details are buried in the supplemental material. As such, I have many issues with this manuscript in its current form, which I outline below.

Generalists: I think the simple explanation for the experimental observations in this manuscript is that the highly influential isolates were resource generalists. The basic theoretical predictions used in this manuscript assume that species are specializing on unique resources and can therefore partition niche space as resource diversity increases. But there was no measure of resource niche breadth or discussion of why specific resources were used in the study (no mention of specific resources in the main text at all, nor of the identity of isolates). Likewise, the media used in the experiment contained dilute undefined media, which includes an enormous amount of resource diversity and therefore complicates any interpretation. Evidence that the results support the role of generalists appears throughout the manuscript (Fig. 1E, L145, L165, L174, Fig. 2D & E, L235, L298, L315). The emergent physiological trait is clearly resource generalism (L317).

Resources and Media: How were the resources in this study chosen? Were there any a priori expectations? Most of these are simple sugars and amino acids. I would predict that many species are generalists in terms of these resources. Further, in

the experiments, why were the cells not washed to remove any carryover undefined media?

Strains: I was unable to find any information on the strains used in this study. Furthermore, I do not see any evidence to support that the highly influential strains were phylogenetically conserved (not even a statistical support for this). Fig. 3B does not provide sufficient support for these conclusions, as the clustering method used in the heat map is unknown, and the identities of the strains are unclear. “Ordered by core-genome phylogeny” is not that meaningful (L258).

Resource uptake: In the abstract, the authors claim that they observed higher resource uptake in more complex resource environments. I do not see how this statement is supported by any of the data provided. In fact, I’m overall surprised by the media conditions. For a study such as this, I would have expected resources to be discussed in molarity and that the effect of molarity was accounted for as resource diversity increased (L294). The observation that strains grew less in mixed-resource supernatants seems obvious, given that the concentration was conserved as additional resources were added.

Lack of Literature: Overall, I found that the authors build their arguments on a minimal selection of the literature. For example, they claim that there is little support for resource diversity driving microbial diversity, citing two publications. However, even a brief Google Scholar search yields numerous examples and a diverse body of literature on this topic. Even one of their main references, Dal Bello et al, does a remarkable job demonstrating the relationship. Therefore, I would not claim that there is “scarce” support for this theoretical prediction.

Complex Environments: When interpreting complex ecological systems, it is perhaps naive to consider resources as the only niche axes that species are dividing, especially in soils, guts, and other complex environments that can maintain diversity due to large variability in environmental conditions.

Figure 1: The legend of Figure 1 references “Animal Distal Gut,” but the figure actually shows “Water”.

The central premise of the paper is that the EMP data provide more support for a negative relationship. In fact, this claim is based on the evidence presented in Figure 1. However, the number of points in the negative relationship, “Water”, is far fewer than the points for “Sediment”. Based on this figure alone, I would conclude that there is more support for the positive relationship. Perhaps I’m missing something, though.

Methods: Overall, I found that there were plenty of details for the bioinformatics section, but the methods were lacking in other sections. For example, the generalized Lotka-Volterra section was not informative at all and merely referred to the supplemental material. More information is needed because this was such a critical part of this manuscript.

Minor:
1. The section titled “Resource diversity fuels competition, which reduced coexistence” is a discussion, not results.
2. Is there a difference between resource diversity and resource complexity (L219)
3. Fig 3B: I cannot see gray in this figure. Perhaps it is my color vision.
4. Why were the sequences not aligned to a reference database (L503)?
5. What is “Predicted microbially derived metabolite richness”? I do not see any mention of this in the Shaffer et al manuscript. They do not use any tool to predict microbially derived metabolites; instead, they use observed metabolites.

Reviewer #3 (Remarks on code availability):

I have done a minor review of the code. From my assessment, the simulation code and the code for some analyses are present. However, it does not appear that this includes the code for all analyses in the manuscript.

Version 1:

Decision Letter:

2nd March 2026

*Please ensure you delete the link to your author homepage in this e-mail if you wish to forward it to your co-authors.

Dear Dr Ratzke,

Your manuscript entitled "Emergent loss of microbial biodiversity with increasing resource diversity" has now been seen by 3 reviewers, whose comments are attached. The reviewers have raised a number of concerns which will need to be addressed before we can offer publication in Nature Ecology & Evolution. We will therefore need to see your responses to the criticisms raised and to some editorial concerns, along with a revised manuscript, before we can reach a final decision regarding publication.

In particular, it is clear from the latest reviewers' comments that there is overstatement in the manuscript and that toning down and further transparency is needed, along with additional methodological and statistical details. We therefore invite you to revise your manuscript taking into account all reviewer comments. In addition, we also think that the first and last lines of the abstract need toning down, and we would like you to reconsider the use of 'emergent' in the title and/or whether the

title can be made more specific.

Please highlight all changes in the manuscript text file in Microsoft Word format.

We are committed to providing a fair and constructive peer-review process. Do not hesitate to contact us if there are specific requests from the reviewers that you believe are technically impossible or unlikely to yield a meaningful outcome.

When revising your manuscript:

* Include a “Response to reviewers” document detailing, point-by-point, how you addressed each reviewer comment. If no action was taken to address a point, you must provide a compelling argument. This response will be sent back to the reviewers along with the revised manuscript.

* If you have not done so already please begin to revise your manuscript so that it conforms to our Article format instructions at http://www.nature.com/natecolevol/info/final-submission. Refer also to any guidelines provided in this letter.

* Include a revised version of any required reporting checklist. It will be available to referees (and, potentially, statisticians) to aid in their evaluation if the manuscript goes back for peer review. A revised checklist is essential for re-review of the paper.

* Extended Data Figures - please ensure that any supplementary figures and tables that are crucial to the manuscript’s conclusions are converted into Extended Data figures and tables to increase visibility of these data. Extended Data figures and tables are online-only (present in the online PDF and full-text HTML versions of the paper), peer-reviewed display items that provide essential background to the article but are not included in the main article due to space constraints. A maximum of ten Extended Data display items (figures and tables) is permitted.

Please use the link below to submit your revised manuscript and related files:

Link Redacted

Note: This URL links to your confidential home page and associated information about manuscripts you may have submitted, or that you are reviewing for us. If you wish to forward this email to co-authors, please delete the link to your homepage.

We hope to receive your revised manuscript within four to eight weeks. If you cannot send it within this time, please let us know. We will be happy to consider your revision so long as nothing similar has been accepted for publication at Nature Ecology & Evolution or published elsewhere.

Nature Ecology & Evolution is committed to improving transparency in authorship. As part of our efforts in this direction, we are now requesting that all authors identified as ‘corresponding author’ on published papers create and link their Open Researcher and Contributor Identifier (ORCID) with their account on the Manuscript Tracking System (MTS), prior to acceptance. ORCID helps the scientific community achieve unambiguous attribution of all scholarly contributions. You can create and link your ORCID from the home page of the MTS by clicking on ‘Modify my Springer Nature account’. For more information please visit please visit www.springernature.com/orcid.

Please do not hesitate to contact me if you have any questions or would like to discuss these revisions further.

We look forward to seeing the revised manuscript and thank you for the opportunity to review your work.

[redacted]

Reviewer expertise:

Reviewers' comments:

Reviewer #1 (Remarks to the Author):

The authors have for the most part done a great job addressing my concerns. The additions to the text have vastly improved it, and the tempering of some claims make the findings more convincing. The additional simulations, such as including additional physiological mechanisms, are very helpful and enrich the paper.
I nevertheless have a few remaining concerns and suggestions:

Title and abstract: I understand the desire to make a provocative claim about biodiversity, but from the rebuttal and the data, my interpretation is that there is generally no positive relationship between biodiversity and resource diversity, rather than that there is generally a negative relationship. Therefore, “Surprisingly, we observe the opposite: microbial biodiversity often declines as resource diversity increases.” in abstract in my eyes does not capture the spirit of the paper and should be

replaced by something more nuanced. For example, I found this statement from the introduction much more fitting: "We found that resource diversity does not consistently correlate with biodiversity (Fig. 1C; Supplementary Fig. 1C-D): in some ecosystems biodiversity increases with higher resource diversity, while in others, it decreases."

Statistics: there are many instances of statistical tests performed across a large number of experimental or simulated conditions, but I can't find in the methods whether and if so, how, multiple testing correction was performed. (e.g., SI Fig 3, but there are many examples)

In the rebuttal, the authors write: "While strain 79 shows the most pronounced competitive response in pairwise experiments, several other strains display similar—though weaker—trends. As shown in Supplementary Fig. 13, strains 95, 81, and 27 also exhibit negative slopes in pairwise settings, with strain 95 in particular showing relatively large absolute impact factors (Supplementary Fig. 11). Thus, 4 out of the 14 tested strains show a consistent negative relationship between resource number and biodiversity in pairwise experiments, indicating that the effect is not exclusive to strain 79." - Without proper statistics, I find this difficult to judge. Strains 79 and 81 are the only strains that show a consistent trend with the number of resources, 95 and 27 show a non-monotonic relationship. Are these slopes significant? Given that there are 14 strains, this makes me wonder whether such behaviors are not expected by chance; 7 of the 14 strains show monotonically positive slopes (though it's not clear whether those slopes are significant). Perhaps the authors could highlight more strongly that strain 79 is an example of a highly competitive strain at high resource diversity and discuss its relationship to the strains in the sentinel experiments? E.g., is 79 closely related to the facilitative or the suppressive HIS?

In the rebuttal, the authors write: "In our corrected results, we now observe that as the fraction of Highly Impacted Strains (HIS) increases, the slope drops sharply from positive to negative, then gradually recovers back to positive/neutral." – This seems to make a clear prediction, namely that the fraction of HIS determines whether the slope is positive or negative. Can the authors reinterpret their results from Fig 1 in light of this finding?

L193ff: I found the new description of the impact factor confusing. "which equals the growth of a strain in the presence of an interaction partner divided by the growth without it. This metric quantifies how the population of one species affects that of another and thus characterizes the influence of a given species." Is the impact factor defined for the species being impacted or for the species having the impact? I believe it is the latter, but it is not clear. Are the impact factors reported in Fig 2D the average of the impact factors measured for one strain across all the pairs it is involved in? Does an IF<1 mean that when that strain is present, other strains tend to have relatively low relative abundance in pairs? Please explain more clearly, perhaps with an example.

L314ff: "These findings suggest that highly influenced strains not only become more competitive in the lab, but also tend to dominate natural ecosystems as resource complexity increases—reinforcing the ecological relevance of our experimental results." – I feel this is an overstatement. HIS are very low abundance indeed. They do become more abundant, yes, but they are not dominant in the environment, unlike in their experiments. I suggest tempering this claim to something akin to "but they tend to become more abundant in natural ecosystems"
Related to this, in Supplementary Fig 23: "Despite finer resolution, the overall pattern recapitulates Fig. 3E: the relative abundance of highly influenced strains increases with resource complexity, whereas mildly influenced and unmapped reads do not." I don't see this in the data: of 8 environments, 3 show a significant (though multiple testing correction may change this number) increase of MIS, 3 show a significant increase of HIS; 2 show a significant decrease of MIS, 1 shows a significant decrease in HIS. Therefore, I would not say that this the trend of HIS increasing with resource complexity "is generally consistent across environments" (l314 in main text). I do wonder how the data in A and B are related. There are three EMPO3 categories for animals (top row), where most points seem to be in the distal gut category, which shows a significant DEcrease of HIS. How then is the overall relationship positive in the EMPO2 animal category?

Minor comments:
L106: "robust to contamination" this is probably unclear without consulting the supplementary figure, perhaps "remained when controlling for potential contamination"?
L155: typo, interactionS
L157: the equation is not displayed correctly
L601: incomplete sentence

Reviewer #1 (Remarks on code availability):

The code looks complete now, though I have not attempted to run it. There is a readme file for each type of analysis/simulation.

Reviewer #2 (Remarks to the Author):

Even if I consider the metabolomics work linking physiology to ecology quite impressive and rare in the field, I am

unfortunately not fully convinced with the more ecological part. Many claims seem overstated to me, and the level of evidence (effect sizes etc) still does often not fully match the ambition of the claims.

1. L302 header: “Increased microbial competitiveness with resource diversity is widespread”. Despite the impressive size of the experiment, I still don’t buy that this trait of being “highly influenced” will be conserved if the same experiment was tried with other phylogenetically different sentinel strains. As evidence for this, we can see at Fig S13, depicting the impact factor of each strain against the other 13, where variation is massive (SEM is plotted as errorbar, and standard deviation, which would be appropriate to describe the data, will be sqrt(13)=3.6 times larger in all cases.
2. The added paragraph L256-262 represents only anecdotal evidence (just one strain), does not disprove Pacheco et al. Indeed, when authors do it later on for a larger number of highly influenced strains, they find that generalism is enriched, but they diminish it (only moderately enriched). Then they say “ this mild correlation was insufficient to explain the pronounced increase in suppression”, but how do they know that it is insufficient? Moreover, later on the authors themselves show that highly influenced strains uptake more resources as more resources become available. Isn’t this exactly the definition of generalism?
3. The explorations of parameter space in the CR model in the last section of results identifies a rather narrow set of conditions in which biodiversity declines with resource availability. This is OK, and is actually a very valuable result, but it should be more transparently acknowledged.
4. Regarding Fig 4E, I am wondering how did the pattern become so qualitatively different from the same figure in the previous version of the manuscript.

Regarding the framing and discussion of the background, I still disagree on some points:

1. Authors claim they are testing whether resource diversity - species diversity theory holds, which is an ambitious claim. The theory has been tested already (as mentioned, dal bello et al, and Pacheco et al). Regarding dal Bello, I do not fully agree that top down community assembly reflects only environmental filtering. While the loss of many species does happen because of environmental filtering, the remaining community does reflect interactions among the species (which of course might be different in a synthetic bottom up assembly scenario). I don't agree they are masked, they can be very strong indeed (see e.g. Goldford et al 2018).
2. The natural relevance of this synthetic community scenario could be also questioned. How many natural communities are assembled in this way? Also, despite the initial claim that the chosen species are phylogenetically diverse, there are still substantial biases which can influence the results, and I could imagine that easily with another set of species one could find a different result.

Note that I might sound harsh, but I am not saying this work is without value. In fact I appreciate, and actually like this type of experiments and approach, I do think the amount of work is impressive and the results are valuable. I am only still a bit bothered by overstatement.

Some additional minor points:

L33: “has been remaining” sounds unnatural in english
L61 - question mark is unnecessary
Supplement, caption new Fig. S6 should be S7
I think Fig. 2E is not a reanalysis of Fig. 1C as stated, but 1E?

Reviewer #3 (Remarks to the Author):

In this revised manuscript, Shalev et al. address the major concerns raised by three reviewers in the original submission. Overall, the manuscript presents an impressive experimental test of the hypothesis that biodiversity increases with resource diversity. Using their synthetic communities and an analysis of the Earth Microbiome Project data, the authors find mixed support for this hypothesis. In the first round of review, it is interesting that all reviewers had very similar concerns about the original manuscript. Among these concerns are the strength of interpretations and the role of generalists. It is striking how similar the reviewers’ comments are. In the revised manuscript, the authors provide a detailed response, major revisions to the original text, and a very lengthy supplement (56 pages and 35 supplemental figures). Overall, the authors have incorporated substantial feedback but ultimately want to maintain their original conclusions.

After reviewing the revised manuscript and the complete response to reviewers, I am pleased to see how much the authors have incorporated feedback. While I still do not fully agree with their conclusions, they have provided detailed justifications for each point. My only major concern now is the strength of some of their statements. I firmly believe they are overinterpreting their results and making them too generalizable. I think their results apply to their simplified system, but I am still not convinced they are applicable to microbial communities writ large. As such, I would ask the authors to provide another revision and tone down some of the comments:

L 27: “Overturn classical ecological principles” – that is way way too strong
L93: “Often” – I believe your results indicate that resource diversity “can” reduce biodiversity. For example, you only observed this in 7 of 14 communities.
L218: It seems that most of your conclusions are the result of a single strain (79). I wouldn’t say that is generalizable at all.

Also, I think you need to provide more details about how you are calculating niche breadth
L268: Increased competitiveness with resource diversity is “Widespread”. As you state in your response to reviewers, this evidence is mixed and a bit complicated to interpret.
L297: Why is $r = 0.36$ $p < 0.001$ only moderate? You interpret other correlations at this level as significant.
L300: Why is this mild correlation insufficient? This is not justified.
L467: You need to modify “often reduces biodiversity”. This is not a justified strong conclusion based on your results. I suggest you tone down this type of language here and elsewhere.

********************END********************

Version 2:

Decision Letter:

9th April 2026

Dear Dr. Ratzke,

Thank you for submitting your revised manuscript "Enhanced resource uptake drives microbial biodiversity loss as resource diversity increases" (NATECOLEVOL-25072385B). It has now been seen again by the original reviewers and their comments are below. The reviewers find that the paper has improved in revision, and therefore we'll be happy in principle to publish it in Nature Ecology & Evolution, pending minor revisions to satisfy the reviewers' final requests and to comply with our editorial and formatting guidelines.

If the current version of your manuscript is in a PDF format, please email us a copy of the file in an editable format (Microsoft Word or LaTex)-- we can not proceed with PDFs at this stage.

If you have not done so already, please ensure that you also email us a completed copy of the Reporting summary :

Reporting summary: https://www.nature.com/documents/nr-reporting-summary.pdf

We are now performing detailed checks on your paper and will send you a checklist detailing our editorial and formatting requirements in about a week. Please do not upload the final materials and make any revisions until you receive this additional information from us.

Thank you again for your interest in Nature Ecology & Evolution. Please do not hesitate to contact me if you have any questions.

[redacted]

Reviewer #1 (Remarks to the Author):

The authors have addressed all my remaining concerns.

Reviewer #2 (Remarks to the Author):

I thank the authors for addressing my concerns in a largely satisfactory manner. In particular, I think that the discussion about difference between generalist and highly-influenced strains is now much clearer, as it seems, in a previous version I did not understand what the authors meant. While I still disagree with some parts (e.g. the discussion on environmental filtering and interactions), this is a matter of discussion. Overall I do find now that broadly speaking the results are more transparent, and the tone and ambition of the claims are much better adjusted to the results.

Minor - Please re-read and fix several typos e.g. L514 "our results has important implications"

Open Access This Peer Review File is licensed under a Creative Commons Attribution 4.0 International License, which permits use, sharing, adaptation, distribution and reproduction in any medium or format, as long as you give appropriate credit to the original author(s) and the source, provide a link to the Creative Commons license, and indicate if changes were made.
In cases where reviewers are anonymous, credit should be given to 'Anonymous Referee' and the source.
The images or other third party material in this Peer Review File are included in the article's Creative Commons license, unless indicated otherwise in a credit line to the material. If material is not included in the article's Creative Commons license and your intended use is not permitted by statutory regulation or exceeds the permitted use, you will need to obtain permission directly from the copyright holder.
To view a copy of this license, visit https://creativecommons.org/licenses/by/4.0/

Reviewer #1 (Remarks to the Author):

In this manuscript, Shalev et al. present the hypothesis that increases in resource diversity do not always lead to an increase in biodiversity, but instead, counterintuitively, can lead to a decrease in diversity. They show this effect in data from the Earth Microbiome Project (EMP), synthetic communities (SynComs), and attempt to explain it using pairwise interaction and metabolomics experiments. The idea overall is interesting and timely, and the amount of data generated for this project is impressive. Nevertheless, I am not entirely convinced by their conclusions which run counter to intuition and previous experiments, and therefore in my view require clearer evidence and a more careful consideration of the existing literature. Below, I provide several comments about the EMP data analysis, in vitro experiments, and the different models used here. Because of the number of different approaches, I have grouped my comments not by major and minor, but by approach.

Discussion of existing literature:
Regardless of the validity of the results of this manuscript (discussed below), more work is required to put the present work in context with existing literature. As the authors mention, there are few quantitative studies of the effect of increasing resource diversity on species diversity. This makes it even more surprising that, even though the two experiments that previously asked similar questions are cited, this manuscript does not engage with those studies at all. In particular, ref 13 has been quite influential in the field and is certainly well-known to the authors. The results in Ref 13 go against the findings of the present manuscript, but those findings are explained quantitatively by a physiology-based model. Why is Ref 13 not discussed at all?

We fully agree that Dal Bello et al. (formerly Ref 13, now Ref 16) is a highly relevant contribution, but its approach differs fundamentally from controlled synthetic-community experiments such as those of Pacheco et al. and our own. Dal Bello et al. analyzed natural soil microcosms containing thousands of taxa, where community outcomes primarily reflect environmental filtering from a vast initial species pool rather than interactions among defined isolates. In contrast, Pacheco et al. (and our approach) used a fixed and well-characterized strain set, minimizing such filtering and isolating the effects of resource-driven interactions on biodiversity.

We have clarified this distinction in the revised text (attached), explicitly noting that while Dal Bello et al. provide compelling ecological evidence that resource heterogeneity shapes community composition, their approach and findings differ from those of Pacheco et al. This revision better emphasizes the scarcity of systematic tests in defined synthetic consortia—the gap our study aims to address.

Attached is our revised version:
"Dal Bello et al.[15] found a positive relationship between resource and microbial diversity in microcosms. However, these experiments involved a single, complex, and undefined soil-derived community containing thousands of taxa. When transferred to laboratory conditions, such diverse natural communities often collapse to a few dominant species, as observed in this study, indicating strong environmental filtering that may mask microbial interactions. In contrast, only one study—by Pacheco et al.[16]— examined how increasing resource diversity affects diversity in a defined microbial community, finding only a weak biodiversity increase, much weaker than that observed by Dal Bello et al. or predicted by classical consumer–resource models. However, as in Dal Bello et al., the use of a single community limits the generalizability of these findings."

Why is the physiology-based model from Ref 13 not applicable here?

The physiology-based model from Dal Bello et al. is not applicable in our case because it follows a fundamentally different modeling philosophy. Specifically, Dal Bello et al. employs a consumer–resource framework in which the metabolite conversion matrix is inferred from a genome-scale model, while other parameters are fixed at arbitrary values. The model further relies on two key assumptions that impose a direct correlation between metabolite and species diversity: (1) each species preferentially consumes only one metabolite, thereby isolating each metabolite into its own ecological niche, and (2) each metabolite is consumed by at least one species, among which exactly one species survives due to competitive exclusion.

While this framework may provide valuable conceptual insights, its assumptions are not supported by experimental evidence and differ from empirical observations such as the

co-utilization of metabolites by multiple species. As a result, the model can reproduce certain observed patterns but does not necessarily represent the underlying ecological mechanisms in experimental systems. More broadly, this reflects a common challenge in ecological modeling: models may successfully reproduce observed patterns, yet the underlying mechanisms generating these patterns in simulations may differ from those operating in real systems.

For this reason, we sought to uncover the underlying mechanisms of our observations as directly as possible through experiments, and to complement these findings with models parameterized on the resulting experimental data. This approach ensures that the mechanisms we propose are firmly grounded in empirical evidence. Accordingly, we focused on models whose parameters can be directly measured or reliably inferred from experiments—an approach that is not feasible for the model described in Ref. 13.

The mismatch between Ref 13, Ref 14, and the data presented here needs to be discussed in detail.

First, we now made the key difference between the experimental designs clearer in the revised main text in the introduction part, as we detailed in our first response above.

Moreover, we added now in the revised discussion a direct comparison with the results of previous studies by Dal Bello et al. and Pacheco et al. (formerly Refs. 13, 14; now 16,17 respectivley). Specifically, we note that while both studies reported weaker-than-expected diversity increases with additional resources, they did not find a decline. We further note the comparison with Pacheco et al., who used a very similar experimental design and found an increase that was much lower than expected, already indicating that the positive relationship between resource diversity and biodiversity is not as strong or as consistent with classical consumer–resource theory. Lastly, by including multiple communities, our study revealed a broader range of outcomes, including consistent diversity reductions, emphasizing that this relationship depends strongly on community composition and interaction structure.

Attached is our revised version:

"Our findings show that, contrary to ecological intuition and widely used models, increasing resource diversity in microbial communities often reduces biodiversity. This extends previous experimental work, which reported weaker-than-expected diversity increases with additional resources but not a decline[15,16]. Compared with Pacheco et al.16, who used a similar defined-community design, our inclusion of multiple

communities uncovered a broader range of outcomes, including consistent diversity reductions. The much lower-than-expected increase observed by Pacheco et al. already hinted that the relationship between microbial biodiversity and resource diversity is not as simple or positive as classical consumer–resource theory suggests. The progression from weakly positive to negative responses observed here underscores that this relationship is not universal but instead depends strongly on community composition and interaction structure. Our results thus highlight that resource enrichment can intensify competitive exclusion, challenging the assumption of a general positive link between resource and species diversity."

Perhaps the data from those papers could even be reanalyzed through the lens of the present paper? Since the supplementary data was not available during review, I do not know the taxonomy of the strains used here, but perhaps there is some phylogenetic relatedness between these strains and those in Ref 13/14 that could be used to infer the degree to which the classification into highly/mildly affected strains can be useful there, as well?

We agree this would be an interesting comparison, but differences in data generation limit its interpretability. Our study uses complete genomes for all 298 strains, whereas these references rely on 16S amplicon sequencing, which lacks strain-level resolution. Because even closely related strains can show markedly different effects, a direct mapping could be unreliable. A meaningful comparison would require complete genomes for all strains, as in our EMP analysis.

Nonetheless, to provide a mechanistic point of comparison, we examined whether our results are broadly consistent with the physiological mechanism proposed by Pacheco et al. We find only limited agreement: generalists are modestly enriched among highly influenced strains, but the correlation is weak and cannot explain the strong suppression we observe (Supplementary Fig. 22; attached here). At the community level, the strain with the strongest biodiversity effect (strain 79) shows only moderate niche breadth compared with the other strains, indicating that its effect cannot be attributed to generalism alone (Supplementary Fig. 15; attached here). Together, these analyses demonstrate that the emergent decrease in biodiversity with increasing resource number cannot be explained by generalism *per se*. Instead, our findings support the mechanistic explanation advanced in the manuscript: synergistic competitiveness, particularly in highly influenced strains, arising from synergistic resource uptake.

We also ran new complementary simulations to test how biodiversity patterns change when resource generalism is more or less correlated with being highly influenced by resource diversity, added in Supplementary Fig. 30 (Attached here). When highly

influenced strains are generalists, biodiversity loss is strongest. As this correlation weakens or becomes negative, biodiversity loss becomes less severe, but still occurs across a wider range of conditions (Supplementary Fig. 30; compare across columns). This is thus consistent with the empirical results.

These new supplementary figures and the revised text have been added and are included as attachments to this rebuttal.

Text revisions:

1) Highly- and mildly-influeced strains analyses:

   "*Because a previous study (Pacheco et al.[17]) linked resource generalism to higher competitiveness under increasing resource diversity, we next examined whether generalism could underlie the enhanced competitiveness of highly influenced strains. Generalist strains were moderately enriched among highly influenced types ($r = 0.36$, $p < 0.001$; $\kappa = 0.36$; Jaccard = 0.43; Supplementary Fig. 22), suggesting that broad niche breadth may facilitate competitive dominance at higher resource numbers. However, this mild correlation was insufficient to explain the pronounced increase in suppression observed under higher resource diversity, indicating that additional mechanisms must drive the stronger competitive responses of highly influenced strains.*"

2) *Community generalism analysis, focusing on strain 79:*

   "We tested whether the mechanism proposed by Pacheco et al.[17] could map onto our results. Their work suggested that higher resource generalism leads to stronger competitive advantage and dominance at elevated resource diversity, reducing coexistence through increased niche overlap. Strain 79, despite its strong biodiversity impact, exhibited only moderate niche breadth compared with the other community members, indicating that its dominance cannot be explained by generalism alone (Supplementary Fig. 15)."

Supplementary figures:

1) *Supplementary Fig 22:*

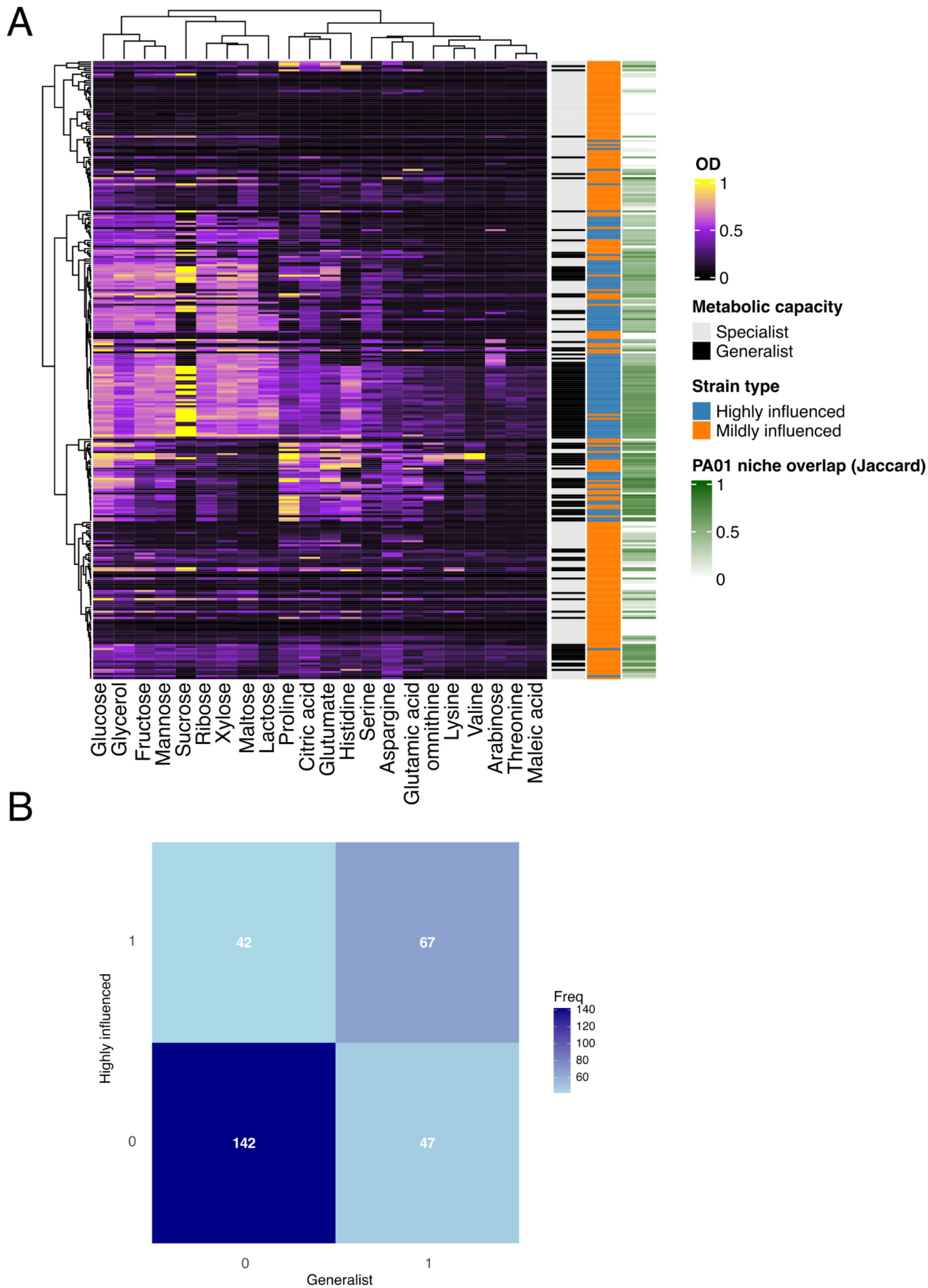
A
Glucose
Glycerol
Fructose
Mannose
Sucrose
Ribose
Xylose
Maltose
Lactose
Proline
Citric acid
Glutumate
Histidine
Serine
Aspargine
Glutamic acid
ornithine
Lysine
Valine
Arabinose
Threonine
Maleic acid
OD
1
0.5
0
Metabolic capacity
Specialist
Generalist
Strain type
Highly influenced
Mildly influenced
PA01 niche overlap (Jaccard)
1
0.5
0
B
Highly influenced
1
0
42
67
142
47
Generalist
0
1
Freq
140
120
100
80
60

2) Supplementary Fig. 15:

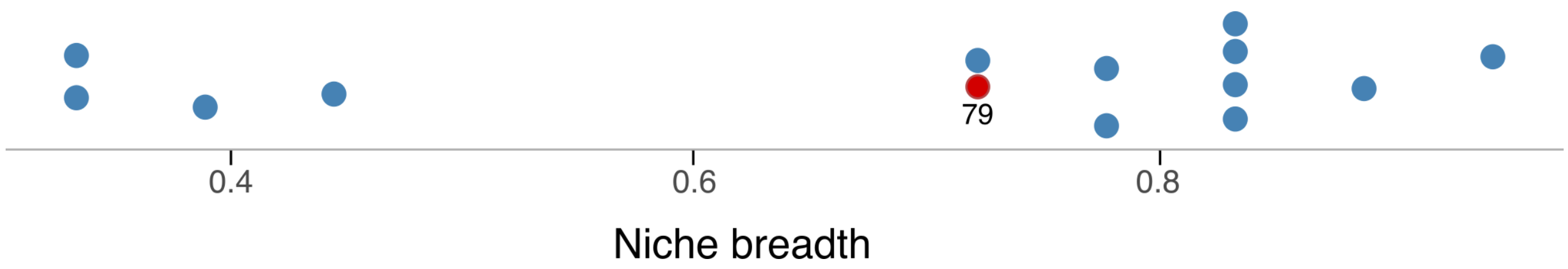

3) Supplementary Fig. 30:

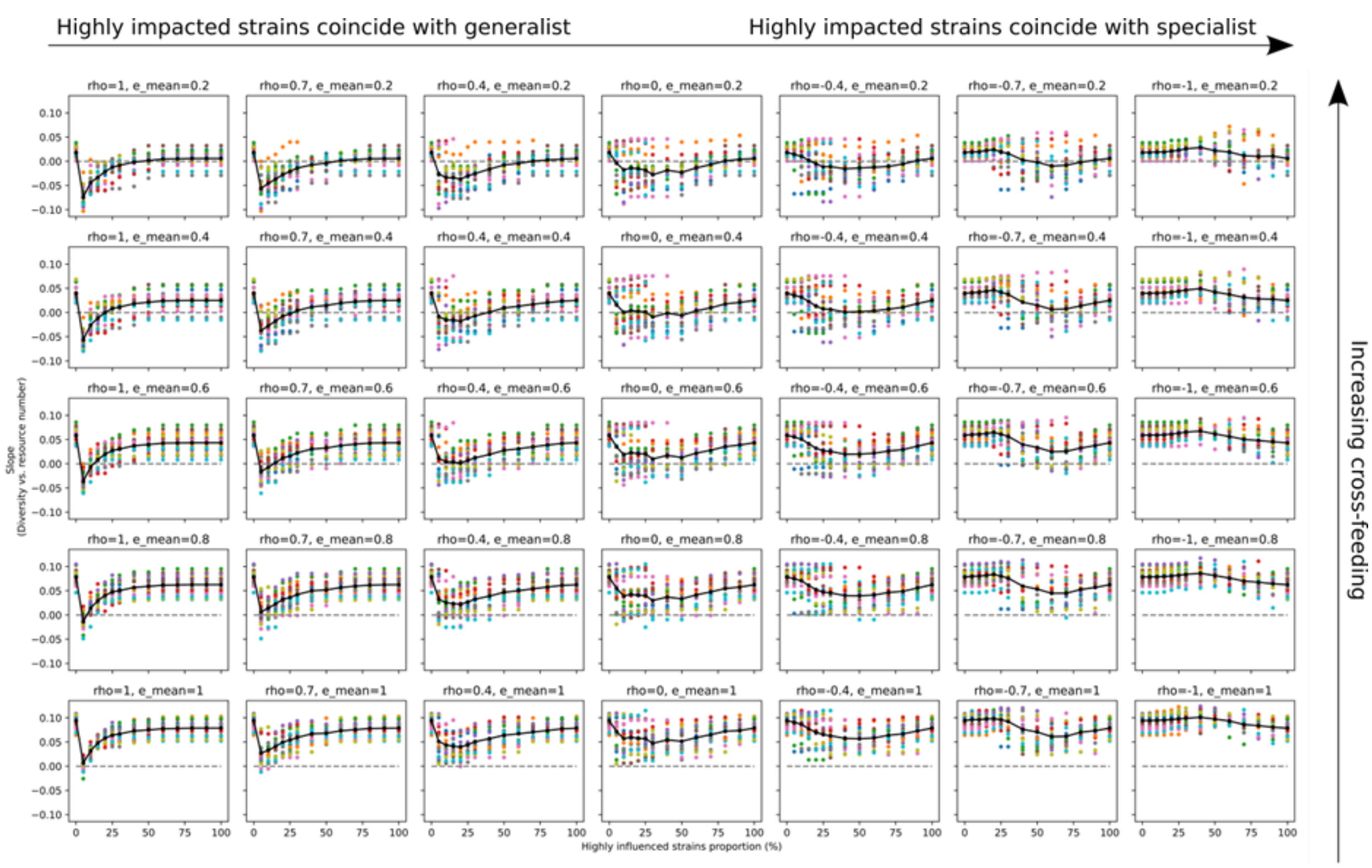

EMP data: An analysis of EMP data appears to show that for many communities, there is a negative correlation between the observed number of metabolites and the Shannon index. The authors interpret this as increased competition and competitive exclusion at higher resource diversity, rather than the intuitively expected increase in diversity at higher resource diversity due to the greater number of available niches.

1. I am not convinced that the results from the EMP and the SynCom experiments in this paper can be interpreted together as they are here; in particular, whether the diversity of metabolites in the EMP and the number of input resources in the SynCom experiments can be interpreted at all as having anything in common. In the EMP, the number of metabolites is the result of microbial activity, both production and uptake. The observed metabolites are those that have been produced and not yet consumed; thus, a sample with a low measured metabolite number might not only arise because the diversity of the incoming resources is low but also because it represents a highly active microbial ecosystem where many metabolites are rapidly consumed; this idea of "missing metabolites" being key in predicting community metabolism was recently used to predict the trophic structure of gut microbiomes (10.1371/journal.pcbi.1007524). For this reason, I find the observation that the correlation between resource diversity and species diversity to be tenuous and potentially explained by a variety of other factors.

We thank the reviewer for pointing out this important tension. We present the EMP analysis primarily as context and motivation for our controlled experiments, as ecological datasets are best suited for hypothesis generation. Because natural microbial ecosystems are inherently uncontrolled and confounded by many unmeasured factors, we performed controlled experiments that allow direct testing of causality under defined conditions. Nonetheless, as this point may not have been sufficiently clear, we have revised the text to clarify it and further softened the language to emphasize that the EMP results are motivational rather than conclusive. Moreover, we fully agree that, in the EMP data, the detected metabolites reflect both supplementation (by microbes or the environment) and consumption processes rather than only external resource inputs, and we have now clarified this as a key limitation in the main text.

Revised text is now:
"We found that resource diversity does not consistently correlate with biodiversity (Fig. 1C; Supplementary Fig. 1C-D): in some ecosystems biodiversity increases with higher resource diversity, while in others, it decreases. But, interpreting these data is difficult, as it is not clear whether the detected nutrients were supplied by the environment or synthesized by the bacteria, complicating efforts to link species composition to nutrient richness. More broadly, natural systems are inherently uncontrolled and confounded by multiple unmeasured factors, which makes data interpretation challenging and asks for controlled lab experiments."

And also:

“Motivated by the difficulty of drawing causal conclusions from the EMP patterns and by the limitations of previous laboratory studies, we systematically tested multiple defined microbial communities…”

2. The GC-MS data in Fig 4 and the discussion around it brings up additional questions about the interpretation of the EMP data: in Fig 4, higher input resource number leads to lower concentration of metabolites (whether the number of metabolites is lower is hard to say, but once concentrations become low enough, they might conceivably be measured as absent). If the authors are correct about the physiological findings (see below), shouldn’t the prediction then be that those EMP communities that show low metabolite counts might have arisen from environments with many input resources? This might at least happen in some environments (and perhaps not in others, depending on the input resources and species present). For me, this makes it even more difficult for me to interpret the EMP results.

We agree that extrapolation from EMP data is limited and have clarified that the EMP analysis is purely contextual (see our seponse above). Specifically, regarding the reviewer’s question, the key distinction lies in the nature of the systems: our experimental setup is a closed system, where a defined set of carbon sources is supplied once, the community grows to carrying capacity, and measurements are taken after stabilization. In contrast, the EMP samples represent open, continuously dynamic systems with ongoing external inputs (e.g., host diet, plant exudates, or diffusion in aquatic environments). The metabolite profiles in such systems, therefore, capture a snapshot of transient metabolic activity, not a defined input–output balance as in our GC-MS experiments. Consequently, expectations derived from our controlled GC-MS data cannot be directly applied to the ecological EMP data. Nonetheless, we view the EMP analysis as valuable contextual evidence that motivated our controlled experiments, rather than a dataset from which mechanistic conclusions can be drawn.

3. Regardless of the interpretation of the number of observed metabolites, I found the statistical effect in the EMP data to be rather mild, and it seems like generally diversity increases with the number of metabolites (“resources”). In SI Fig 1, after multiple testing correction, only a single ecosystem type (Animal distal gut, non-saline) shows a significant negative correlation, hardly a huge effect. By contrast, the four strongest correlations are all positive.

We agree that the statistical effect observed in the EMP data is relatively mild. This likely reflects both the limited sample size within individual environments and the inherent variability of ecological datasets, where strong or consistent relationships are not necessarily expected. However, the overall trend is not generally positive, which is

our main contextual point. We have refined the text to clarify this. The relevant revised section now reads:

"...Yet despite decades of theoretical support, empirical evidence for this link remains scarce[15,16], raising the question of whether this long-standing theory truly holds in microbial ecosystems? As a first step toward exploring this question, we re-analyzed data from the Earth Microbiome Project[17] that contained both species diversity (measured by DNA sequencing) and nutrient diversity (measured by mass spectrometry) for the same samples, allowing us to examine the connection between both."

We note that, contextually, we frame these results in the Results section not to emphasize a decrease, but rather the lack of evidence for a general increase in biodiversity:

"We found that resource diversity does not consistently correlate with biodiversity (Fig. 1C; Supplementary Fig. 1C–D): in some ecosystems biodiversity increases with higher resource diversity, while in others, it decreases."

To further clarify this point, we would like to draw the reviewer's attention to the following observations: (1) the environment with the largest sample size—Animal Distal Gut (non-saline)—shows a significant negative slope, and (2) across all environments, positive and negative slopes are evenly split (six positive, including two close to zero, and six negative; main Fig. 1C; Supplementary Figs. 1C–D), with only four of twelve showing a significant positive relationship (conistent with the reviewer's observation).

Thus, our intention is not to argue that the EMP data provide strong evidence for negative relationships, but rather to highlight the absence of a widespread positive trend, as would be expected from classical consumer–resource theory (Fig. 1A–B vs. 1C).

4. Fig 3E: after identifying highly influenced strains (HIS), the authors map EMP reads to phylogenetically related strains and find that only those strains have a positive correlation with the number of measured metabolites. However, I think a crucial point of this analysis that is currently missing is the potential bias of finding these strains across the different ecosystems, which additionally might each have their own biases for having a high/low number of metabolites and its correlation with diversity. Without a more careful analysis, it is unclear to me that this analysis proves the "ecological relevance" of the experimental results (L245).

We addressed potential ecosystem-level bias using a linear mixed-effects model that accounts for environmental variation (relative abundance ~ metabolite richness × strain

type + (1 + metabolite richness | environment); n = 1,224). The model confirmed a significant interaction between metabolite richness and strain type ($p = 2.5 \times 10^{-9}$), with HIC strains showing the strongest positive association (slope = 0.165 ± 0.051), exceeding both MIC strains (0.073 ± 0.051; $p = 0.0011$) and strains of unknown type (0.001 ± 0.051; $p < 0.0001$); MIC strains also differed modestly from unknown strains ($p = 0.016$). Random-effect variances for intercepts (SD = 0.12) and slopes (SD = 0.11) were small relative to the residual variance (SD = 0.37), indicating that these trends—particularly for HIC strains—are consistent across environments rather than driven by any single ecosystem. This analysis is included in the caption of new Supplementary Fig. 23 (attached here), which also shows fine-scale results within individual EMP environments, and is now referenced in the revised text. We now refer to it in the revised text: *"This trend is generally consistent across environments (Supplementary Fig. 23)"*.

Specifically, Supplementary Fig. 23A shows, using the EMPO3 classification employed throughout the manuscript and in the mixed-effects model, that HIC strains exhibit positive slopes in 7 of 8 environments (3 significantly positive, 1 significantly negative), whereas MIC strains show less consistent trends (positive in 5 of 8 environments; 3 significantly positive, 2 significantly negative). For completeness, we also include a coarser EMPO2-based analysis (Supplementary Fig. 23B), which yields the same overall pattern, with only HIC strains displaying significant positive slopes across all three environments.

A

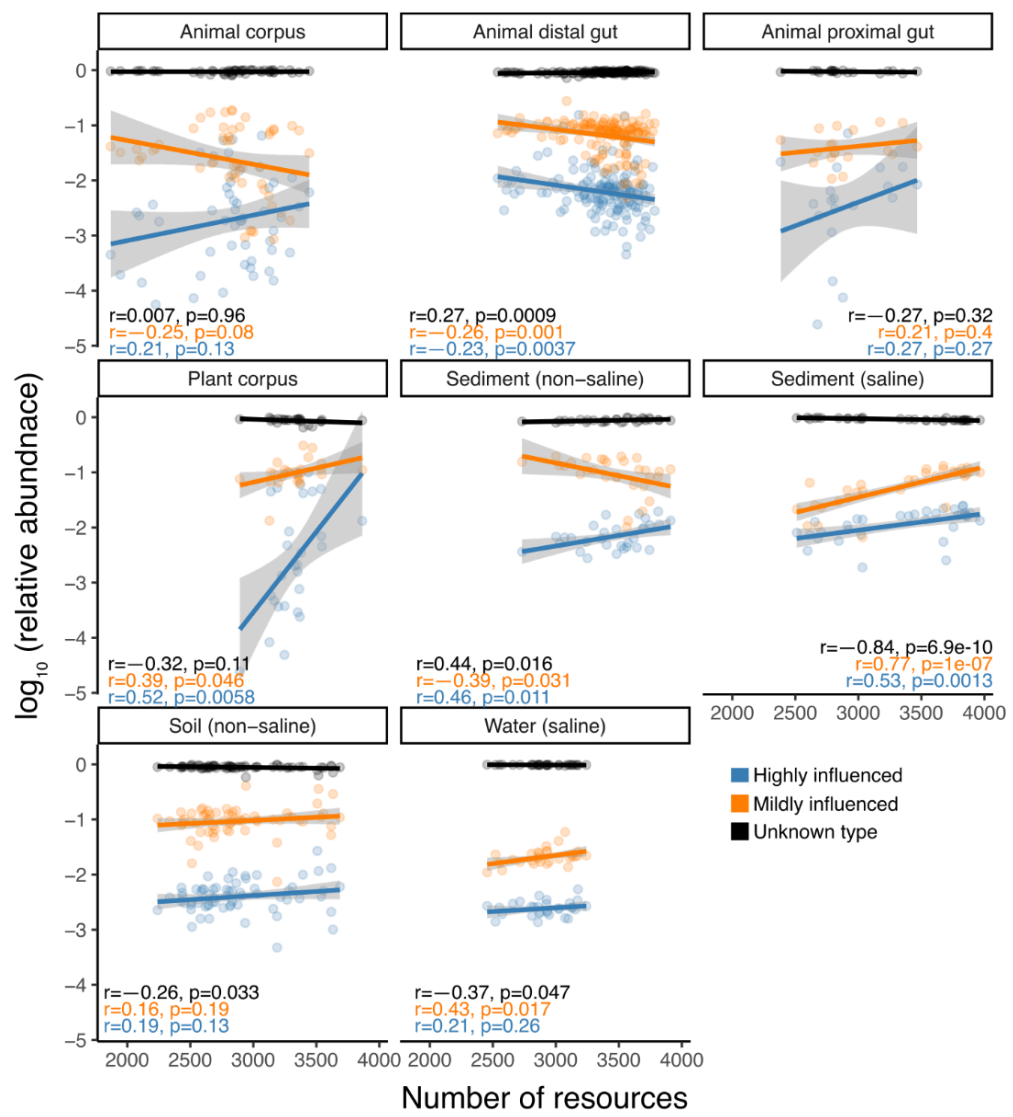

B

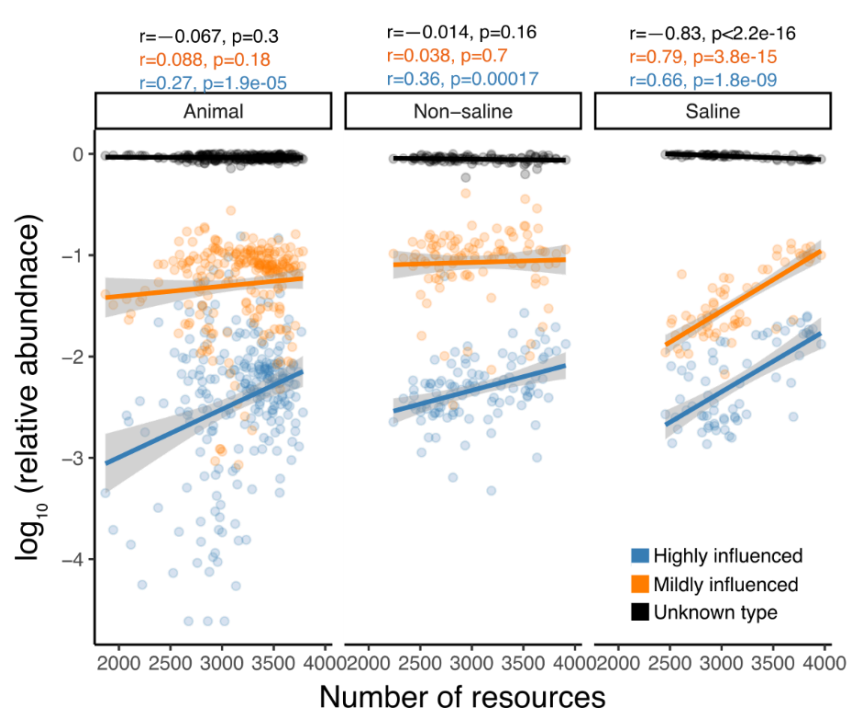

Additionally, the HIS seem to be at much lower average relative abundance than the MIS, which goes against the experimental finding that the HIS dominate the communities.

The apparent difference in relative abundance between HIC and MIC strains likely reflects technical limitations, including the smaller number of HIC genomes in our reference database (109 of 298) and the large fraction of environmental reads that remain unmapped (which may derive from either group). In addition, natural microbial communities are shaped by many interacting ecological and abiotic factors, making them substantially more complex than our controlled experimental system and potentially obscuring dominance patterns in the field. Despite these constraints, the positive association between metabolite richness and HIC abundance is consistently observed across environments, supporting the ecological relevance of our experimental (Fig. 3) and modeling results (Supplementary Fig. 29).

Experimental community data: The authors aim to reproduce the trends observed in the EMP by assembling SynComs from 6 or 8 species across a variety of different resources and resource combinations.

1. In Fig 1E, the Shannon index shows some bias towards negative correlations with the resource number. I believe some of this bias may come from the fact that many communities maintain a lot of initial diversity even after 9 transfers (which in itself I found surprising). For example, even at a single resource, the 6-species SynComs still contain on average about 5 species. Translated to the Shannon index, a simple calculation shows that for a random community with uniformly distributed relative abundances, one would expect a Shannon index of 1.6 and 1.9 for 6 and 8 strain SynComs, respectively. Some communities seem to be close to that threshold even for a single resource (e.g., 5, 3), such that they could not possibly have a high positive slope with resource number, while negative slopes are comparatively less constrained.

We thank the reviewer for this insightful observation. We agree that the relatively high biodiversity observed under single-resource conditions can constrain the maximum attainable positive slope with increasing resource number. Rather than reflecting an experimental artifact, we view this as a biologically meaningful result: many microbial communities maintain substantial coexistence even at low resource diversity. This is consistent with our model inference that self-inhibition typically exceeds interspecies inhibition ($\alpha\_self > \alpha\_other$), enabling stable coexistence of multiple strains (Fig. 2B), as well as with the prevalence of cross-feeding interactions, which are strongest under single-resource conditions (Supplementary Fig. 16).

We also note that the absence of shaking in our setup may promote micro-niche formation, further supporting coexistence.

To directly assess whether high diversity under single-resource conditions could mathematically bias slopes toward negative values, we performed new consumer–resource model simulations spanning a range of cross-feeding levels. As shown in Supplementary Fig. 30 (attached), increasing cross-feeding elevates diversity under a single resource and reduces slope magnitudes; however, neutral or negative slopes remain highly unlikely unless highly impacted strains are present in the community. Thus, even when single-resource diversity is high, the default model does not produce the negative slopes observed in Fig. 1. This indicates that the bias toward negative slopes reflects biological interactions—specifically the presence of highly impacted strains—rather than a mathematical constraint imposed by high baseline diversity.

Supplementary Fig. 30:

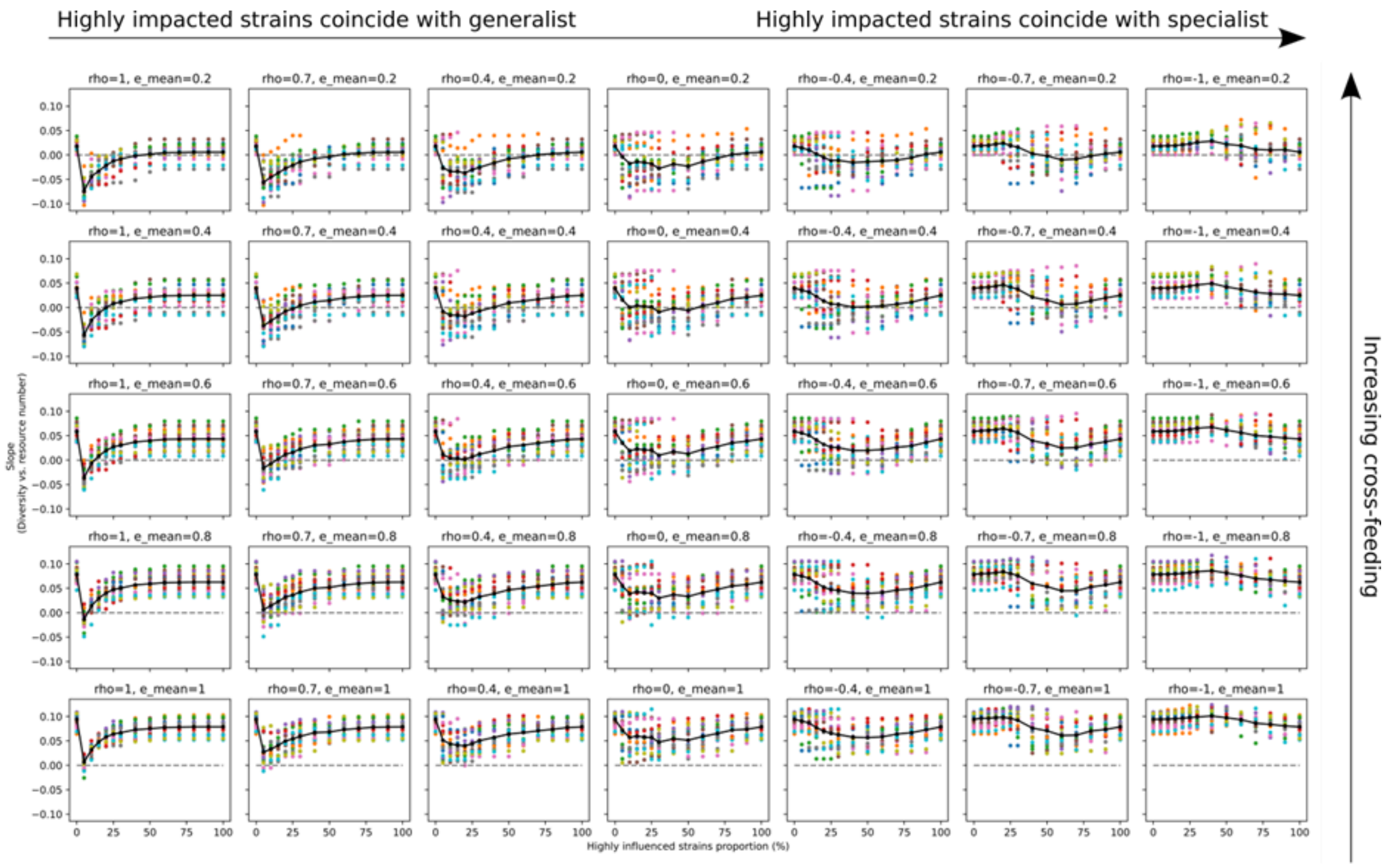

2. In some cases, the richness is greater than the number of input strains. What are those additional strains, contaminations?

We thank the reviewer for raising this important point. Occasional low-level contaminants can indeed explain the few cases in which richness marginally exceeded the number of inoculated strains. Most samples showed no detectable contaminants, and when present, these taxa typically accounted for ~0.1% relative abundance. All SynComs were assembled from a common 14-strain pool (used to construct the 6- and 8-member communities), and reads were mapped against the complete 14-strain reference database. Any additional taxa detected are therefore likely true low-level contaminants or artefacts introduced during PCR, library preparation, sequencing, or mapping, despite extensive quality-control steps (including chimera removal and parameter optimization).

To ensure robustness, richness was calculated using a 0.05% relative-abundance threshold, excluding extremely low-abundance taxa. The results are robust to this choice: reanalyses using no cutoff (0%) or a stricter 1% threshold yield the same qualitative trends. For transparency, we now include these analyses in the new Supplementary Fig. 3A–B (attached), which visualize contamination levels and confirm that contamination has negligible impact on the observed patterns.

We now clarify this point in the main text as follows: “Contrary to predictions from the Consumer–Resource Model (Fig. 1B), microbial biodiversity declined in a large fraction of communities as resource diversity increased (7 of 14 communities showed declines in both Shannon diversity and richness), while increases in biodiversity were rare and typically modest (Fig. 1E; Supplementary Figs. 1E–F; Supplementary Figs. 3–4).”

We also explicitly state the abundance threshold in the revised Supplementary Fig. 1 caption: “Richness was calculated using a 0.05% relative-abundance threshold to exclude sequencing noise and extremely low-abundance taxa. Results are robust to this cutoff choice, yielding similar qualitative trends when using no threshold (0%) or a higher threshold (1%; Supplementary Fig. 3).”

Supplementary Fig. 3A-B:

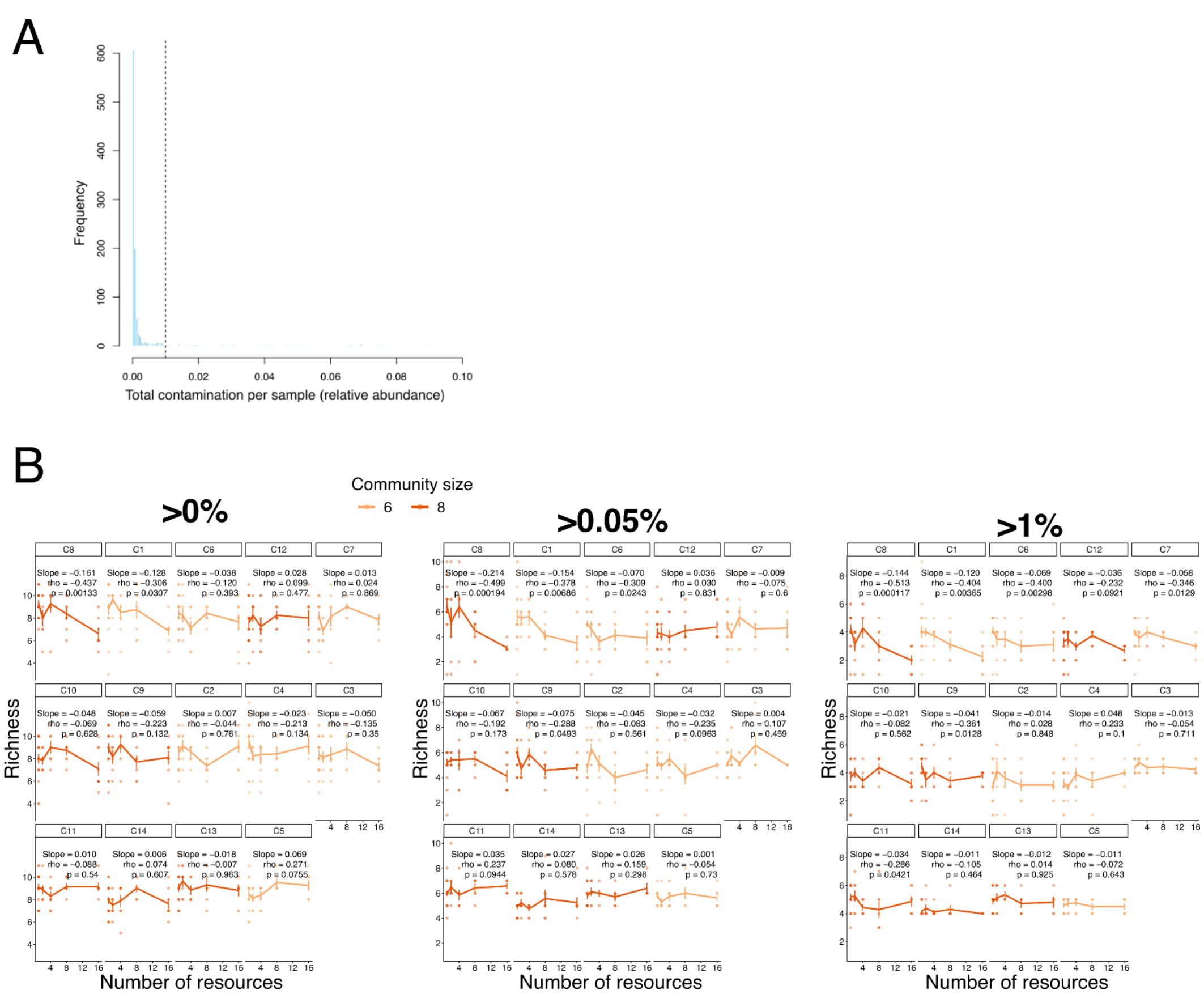

3. In terms of richness, most communities seem to show no correlation with the number of resources.

We have now added the correlation values between richness and the number of resources in the new Supplementary Fig. 3B (attached above), including Spearman correlation coefficients and their corresponding p-values. Using a richness threshold of 0.05% relative abundance (consistent with Fig. 1 and all Supplementary Figures), we find that 4 out of 14 communities show statistically significant correlations, all of which are negative. However, while the majority of communities do not exhibit significant correlations, this primarily reflects the absence of strong monotonic relationships rather than mixed directional trends. Consistent with this, the estimated slopes (shown in Supplementary Fig. 3) are negative in 8 out of 14 communities, mirroring the overall pattern observed for Shannon diversity (a detailed comparison between Shannon diversity and richness is provided below).

In addition to my point 1 above, while there does seem to be an effect in the Shannon index, this lack of correlation in richness vs resource diversity seems to imply that the most significant change is that a small number of strains are more dominant at higher resource numbers, rather than competitive exclusion, as the authors describe it.

First, we have to point out that we used the term “competitive exclusion” in our manuscript mainly in the context of paiwise interactions and in particular the effect of alpha_self vs alpha_other on coexistence in pairs. We did not claim that this is the major driver of biodiversity loss in communties. However the reviewer raises here an important point about the type of biodiversity loss (species loss vs eveness loss) in communities.
To address this point in more depth, we have now directly compared the slopes of Shannon diversity and richness as a function of resource number and find them to be highly correlated (Supplementary Fig. 3C; attached above). Specifically, 7 out of 14 communities show a decrease in diversity with increasing resource number for both Shannon and richness indices, and only two communities display opposing trends. Overall, 12 out of 14 communities show the same directional response for both metrics when using the 0.05% relative abundance threshold applied throughout Fig. 1.

These results indicate that Shannon diversity and richness capture consistent underlying trends, even though richness alone shows weaker statistical significance with respect to resource number. Importantly, the observation that richness decreases in many communities implies that taxa are lost entirely as resource number increases, which is indicative of competitive exclusion rather than solely increased dominance of a small number of strains.

This interpretation is further supported by our pairwise experiments, which provide direct evidence for competitive exclusion. Thus, the observed patterns cannot be explained exclusively by the enhanced growth of a few dominant species. That said, we

agree with the reviewer that the two diversity indices differ in sensitivity: in some communities, Shannon diversity may decrease more strongly than richness, potentially reflecting increased dominance while low-abundance taxa persist. However, given the strong correlation between the slopes of Shannon diversity and richness across communities, we find no evidence that dominance without exclusion is the primary driver. Instead, the data indicate that competitive exclusion remains the predominant mechanism shaping community diversity as resource number increases (Supplementary Fig. 3C).

Supplementary Fig. 3C:

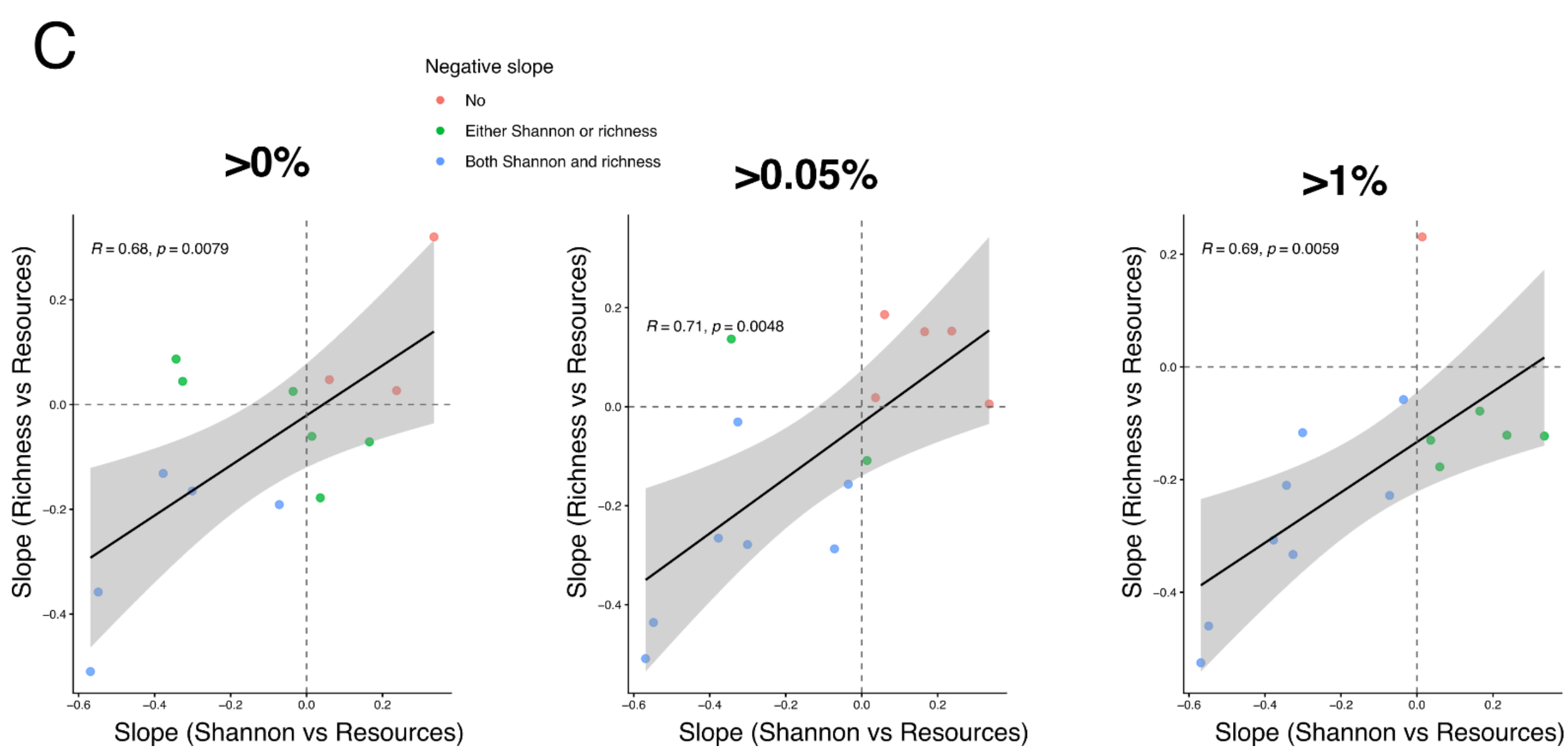

In summary, I am not so convinced that a “large fraction” (L78) of communities showed a diversity decrease, or that there is “widespread biodiversity loss” (L85); those sound like overstatements to me that rely on choosing Shannon diversity over richness, and are potentially confounded by the issues raised above.

Regarding the phrasing (“large fraction” and “widespread biodiversity loss”), we agree that these statements should be supported carefully. As detailed above, our data support a consistent decrease in diversity when considering overall trends across both Shannon diversity and richness. Specifically, 7 out of 14 communities exhibit negative slopes with increasing resource number consistently across **both** indices, based on per-environment correlation coefficients (Supplementary Fig. 3C, attached above). In addition, the magnitudes of negative correlations (|r|) are generally larger than those of positive correlations, indicating that diversity decreases are not isolated cases (Supplementary Fig. 3C).

To improve clarity and avoid overstatement, we have revised the text to explicitly state that “7 of 14 communities showed declines in both Shannon diversity and richness” (Supplementary Fig. 2C). In parallel, we have softened the language by replacing “a large fraction” with “a notable fraction,” and we no longer refer to the effect as “widespread biodiversity loss.” These changes better reflect the quantitative scope of the observed trends while preserving the central conclusion.

Interactions and physiology: The authors aim to explain their SynCom findings with an impressive number of interaction and metabolomics measurements. They find lower self-inhibition, more resource consumptions, and lower metabolite excretion at higher resource number, which increases the proportion of negative interactions.

1. As the authors state, a lower self-inhibition at higher resource number, i.e., a high carrying capacity, is a counterintuitive result – I would expect not all strains to consume all the resources. To arrive at the self-inhibition values, are the carrying capacities averaged over several carbon sources? (This seems to be described in SD3, but this was not available at the time of review – still, this should be described in the text somewhere.) Surely, the carrying capacity is different for each carbon source, including zero in some cases? Given the counterintuitive nature of this result, it needs to be made much clearer that it is not an artefact of the methodology.

The result is surprising and at the core of our work. Indeed the carrying capacity (1/alpha_self) is expected to vary with the exact carbon source conditions. To take this variation into account, we fitted the general Lotka-Volterra model with a Bayesian regression to the data. Bayesian regression treats parameters naturally as random variables and returns their underlying probability distributions (or samples thereof). The values shown in Fig. 2B are the mean of these samples. All of this is stated in Supplementary Text 2.

To make this more transparent in the main text we extended the main text as following:

“Interaction matrices were inferred by a Bayesian regression of a generalized Lotka–Volterra model and the mean interaction coefficients for 1, 8 and 16 carbon sources were calculated (Supplementary Fig. 4, Supplementary Text 2), allowing comparison of intra-species (αself) and inter-species (αothers) interactions across resource levels (panel B).”

To further highlight this point, we have now included data from measurements of several of our strains grown at different nutrient diversities (1, 8, and 16 nutrients, with the same total nutrient concentration). These results likewise show an increase in carrying capacity with increasing nutrient diversity. This information is included in the revised

Supplementary Fig. 5 (attached here) and referenced in the main text (*"We also measured how each strain grew in isolation in each environment (Supplementary Fig 5)."*. We will discuss this interesting phenomenon in detail in a separate publication.

Supplementary Fig. 5:

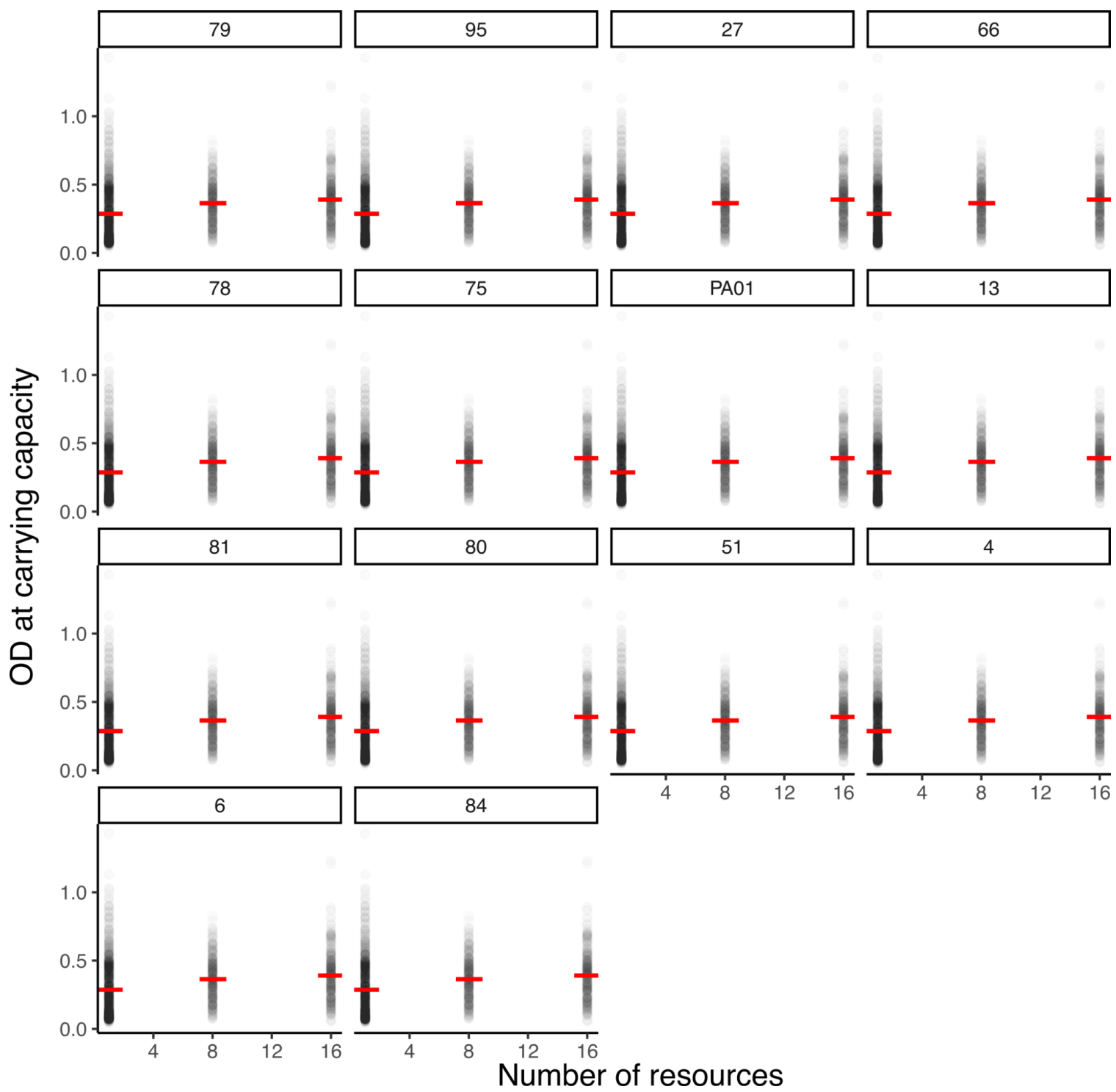

2. Minor point: this manuscript likely will be read by many microbiologists, and explicitly providing values of OD for the carrying capacity may make the manuscript more accessible to that audience.

We thank the reviewer for pointing this out, please see our comment above.

3. SI Fig 10 shows that the strain 79 that drives biodiversity loss with resource number is a clear exception – all other strains appear to be neutral or have a positive impact on biodiversity. To me, this suggest that while there is clearly an effect with this strain, it may be a physiological anomaly; perhaps this strain has a very strong effect on pH or produces antibiotics and ALSO happens to dominate at high resource numbers (perhaps because it is very metabolically flexible and can consume every resource rapidly). It is therefore perhaps not a coincidence that strain 79 also has by far the largest absolute values of impact factors (SI Fig 11).

Thank you for this insightful comment. We agree that strain 79 exhibits a particularly strong effect on biodiversity as resource number increases. However, our data indicate that it is not a physiological outlier in terms of niche breadth (new Supplementary Fig. 15; attached here), nor is its effect unique.

While strain 79 shows the most pronounced competitive response in pairwise experiments, several other strains display similar—though weaker—trends. As shown in Supplementary Fig. 13, strains 95, 81, and 27 also exhibit negative slopes in pairwise settings, with strain 95 in particular showing relatively large absolute impact factors (Supplementary Fig. 11). Thus, 4 out of the 14 tested strains show a consistent negative relationship between resource number and biodiversity in pairwise experiments, indicating that the effect is not exclusive to strain 79.

The apparent prominence of strain 79 in community-level analyses is partly attributable to experimental design: it was present in 8 of the 14 randomly assembled communities, increasing statistical power to detect its effect. To assess whether the observed biodiversity loss reflects a more general mechanism, we therefore turned to our experimentally grounded modeling framework (Fig. 2B; Supplementary Figs. 6–11). These analyses demonstrate that biodiversity loss emerges when self-inhibition (α_{self}) decreases more strongly with resource number than inter-strain inhibition (α_{others}), providing a mechanistic explanation that does not rely on a single strain.

This conclusion is further supported by the supernatant experiments, which reveal non-additive increases in resource uptake across species under mixed-resource conditions (Fig. 4B; Supplementary Figs. 24–25), consistent with reduced self-inhibition. Importantly, replenishing fresh media restores growth of the sentinel strain (Supplementary Fig. 26), arguing against toxicity (e.g., antibiotics or pH effects) as the primary driver.

Taken together, these results indicate that while strain 79 represents the strongest observed example, the underlying pattern reflects a general ecological mechanism—enhanced competitiveness under higher resource numbers—rather than a strain-specific physiological anomaly.

Supplementary Fig. 15:

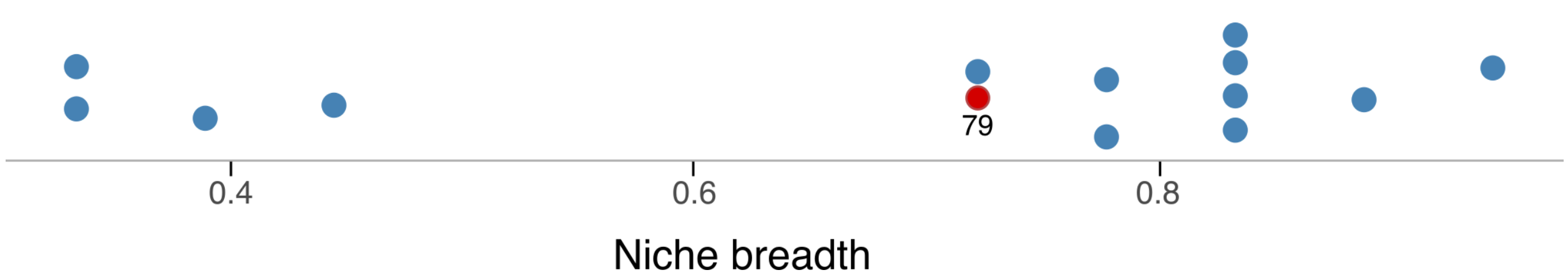

4. Sentinel experiments, Fig 3B: It is interesting to know what the strains and resources are that give rise to strong facilitation and strong suppression. This information is contained in SI Fig 17, where it looks like the strongly facilitative (not "facultative") strains are facilitative in sugars and suppressive in acids. In the absence of information about the strains' taxonomy, this is reminiscent of patterns observed between enterobacteria (E) and pseudomonadales (P, like the sentinel strain used here), where E produce organic acids on sugars that P prefers over sugars. Thus, I wonder whether the choice of sentinel strain is crucial here in determining the impact of different strains. Would the results have looked different if the sentinel strain was, say, an enterobacterales strain?

We thank the reviewer for raising the question of whether the choice of *Pseudomonas aeruginosa* PAO1 as the sentinel strain could influence our conclusions, and for emphasizing the importance of providing detailed taxonomic information. To address the latter, we added full taxonomic annotations for all strains in Supplementary Data 5 and visualized them at the order level—together with the HIC/MIC classification and their effects on the sentinel—in the new Supplementary Fig. 19 (attached here). This is now referenced in the main text: "Both types were phylogenetically conserved (Fig. 3B, top; Mantel test, r = 0.145, p = 0.0001, 9,999 permutations; For taxonomic information see Supplementary Fig. 19 and Supplementary Data 5)". As the reviewer correctly inferred, *Enterobacterales* are enriched among facilitative strains, likely by fermenting sugars that PAO1 cannot directly use into metabolites accessible to PAO1.

We agree that any single sentinel strain introduces some bias. We selected *P. aeruginosa* PAO1 because it allows stable chromosomal integration of the *lux* reporter via the *tn7* system, ensuring consistent single-copy expression; it is a metabolic generalist with broad niche breadth; and it grows robustly at 30 °C, matching the growth range of our strain collection. These practical and biological properties make PAO1 a suitable and reliable sentinel for quantifying competitive balance across diverse resource conditions. We now refer to this rationale directly in the Methods text:
"*Sentinel strain selection rationale*

We selected Pseudomonas aeruginosa PAO1 as the sentinel because it supports stable, single-copy chromosomal integration of the luxCDABE reporter via the mini-Tn7 system—ensuring consistent reporter dosage, avoiding plasmid-borne variability, and yielding a strong, reliable bioluminescence signal compatible with our plate-reader workflow. PAO1 grows robustly at 30 °C—matching the growth range of our strain collection—and exhibits broad metabolic niche breadth (generalist physiology), so its growth is sensitive to a wide range of changes in metabolic space. These practical and biological properties make PAO1 a reliable readout strain for quantifying competitive

balance across diverse resource conditions. We note that any single sentinel may introduce bias."

Importantly, the trends observed for the 298 strains using PAO1 are generally independent of sentinel identity, supported by two lines of evidence. First, GC–MS supernatant experiments—conducted without a sentinel—recapitulate the increased resource uptake and stronger competitive environment under mixed-resource conditions, with a stronger effect among highly influenced (HIC) strains. This confirms that the HIC/MIC distinction identified via PAO1 reflects a general biological property rather than a sentinel-specific artifact. We now refer to this in the revised main text:
"As both the sentinel-independent (GC–MS results; Fig. 4B) and sentinel-dependent (suppression assays; Fig. 4C) analyses yield consistent results, this indicates that our key findings (Fig. 4C; Supplementary Figs. 16–23) are generally sentinel-independent."

Second, even when considering sentinel-specific phenomena such as facilitation and suppression, the number of resources remains the main driver of the shift toward greater competition and suppression, as shown by new analyses focusing on PAO1's responses under exclusively facilitative or suppressive conditions (Supplementary Fig. 27; attached here), which demonstrate that facilitation declines and suppression increases with resource number. We now refer to this in the revised main text as follows: "Moreover, increasing resource number weakened facilitative effects and intensified suppressive effects, regardless of whether communities were composed exclusively of facilitative or suppressive interactions (Supplementary Fig. 27)."

Together, these results demonstrate that the responses of the 298 strains to increasing resource diversity are largely independent of any properties unique to PAO1.

We therefore hypothesize that using a different sentinel strain with a large enough niche breadth (e.g., an *Enterobacterales* isolate) would yield qualitatively similar results, showing the same distinction between highly and mildly influenced strains (HIC/MIC) and the same non-additive responses, with minor differences that cannot be ruled out.

Supplementary Fig. 19:

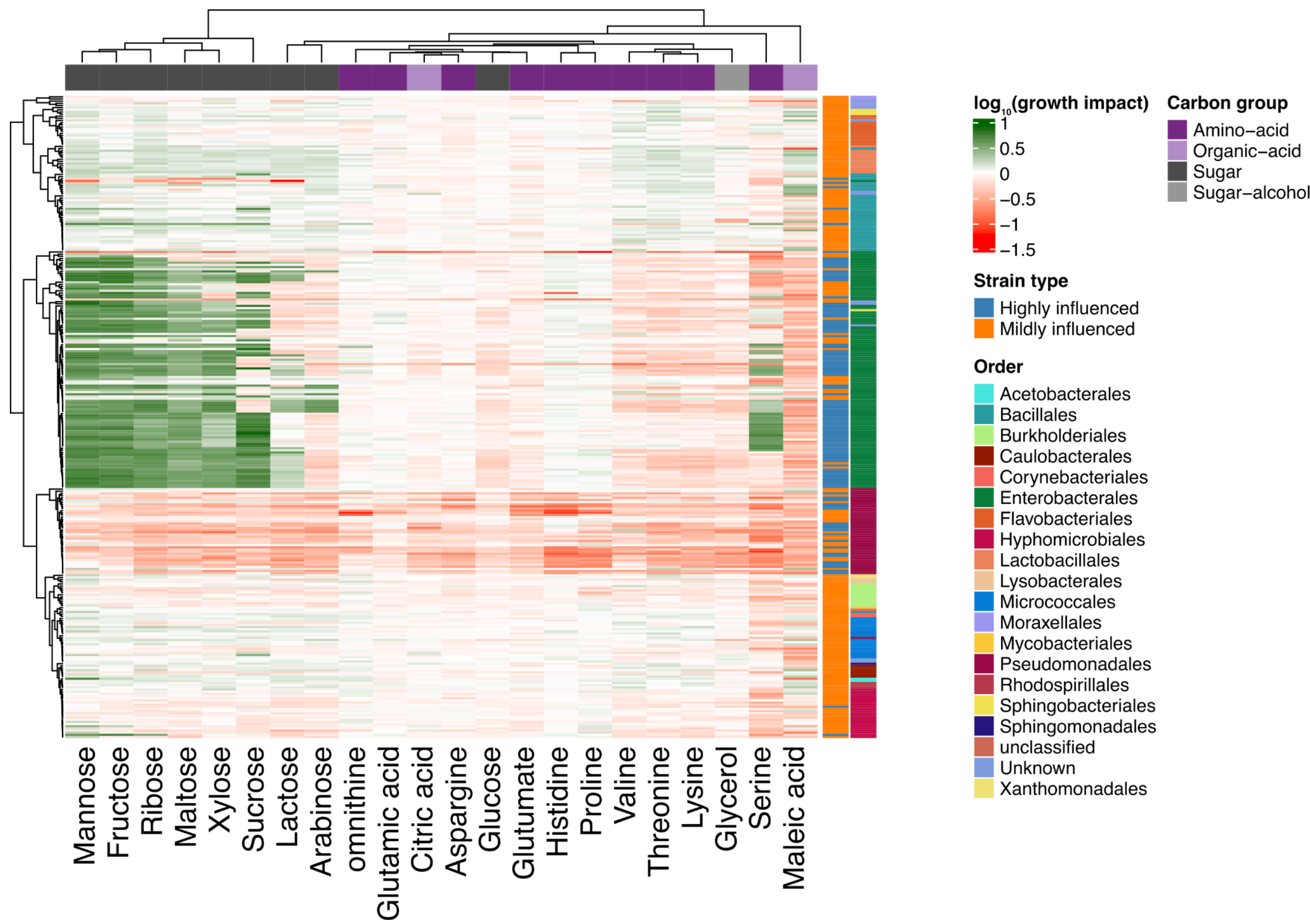

Supplementary Fig. 27:

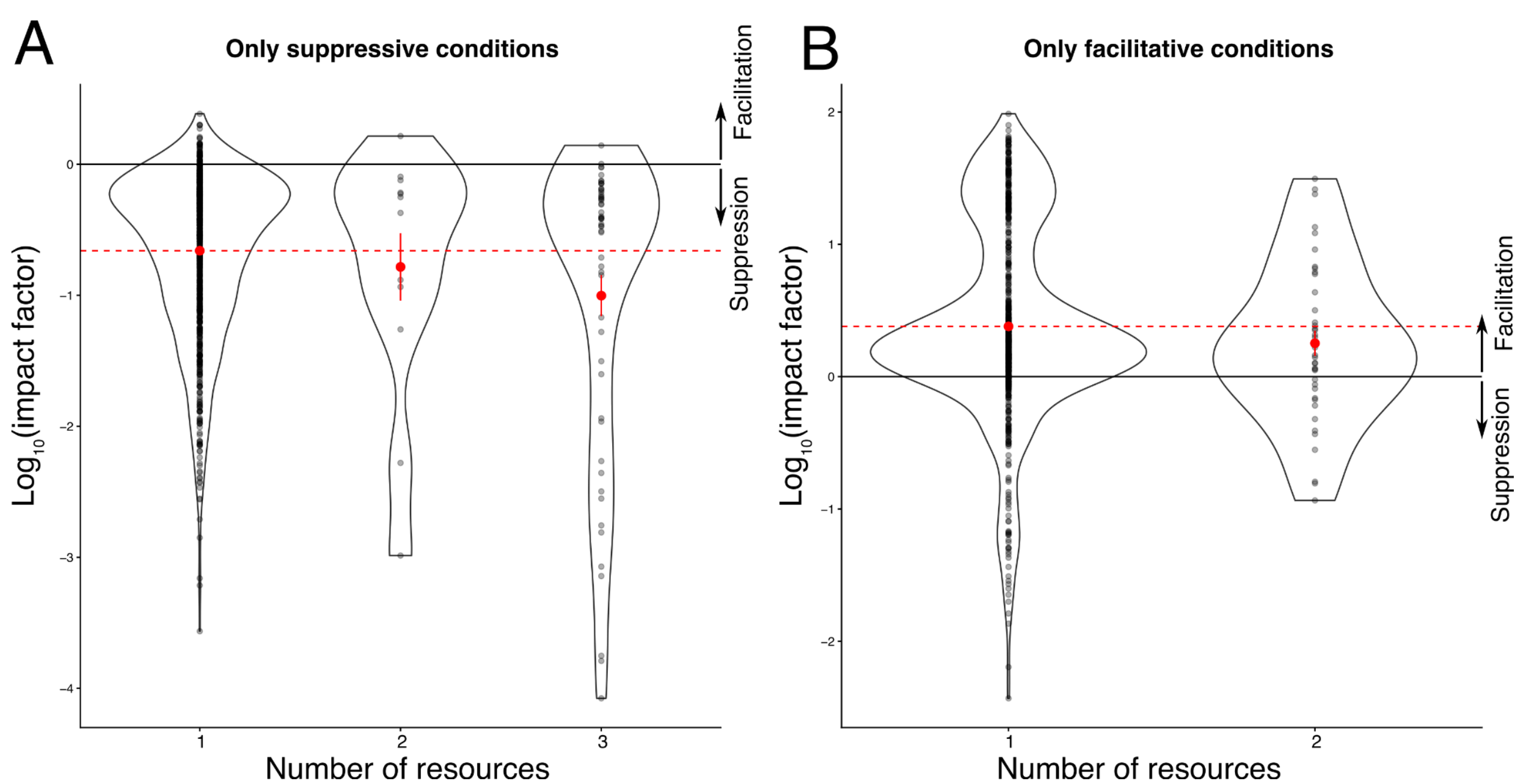

5. There is no discussion for why or how some strains might be highly or mildly influenced by resource number. Overall, the fact that the classification as a highly or mildly influenced strain has such a strong phylogenetic correlation suggests to me that there may be a clear physiological idiosyncrasy that explains the highly or mildly influenced classification, rather than the classification being an emergent feature that can appear across many clades. The data in Fig. 3B would offer a possible physiological explanation for the increased suppression at higher resource number that differs from the authors' interpretation: as the number of resources increases, the proportion of resources that allow for strong facilitation decreases (e.g., among the 8 resources, there might only be 4 fermentable sugars). At the same time, there is always competition for the organic and amino acids, leading to overall more suppression at higher resource diversity. Such an explanation would be satisfying if correct and inject some much needed physiological mechanism into the present findings, which I find currently lacking (e.g., in L232, the loss of facilitation is mentioned but no potential mechanism is offered).

We thank the reviewer for this constructive comment and for encouraging a deeper discussion of potential physiological mechanisms underlying the distinction between highly influenced (HIC) and mildly influenced (MIC) strains. While we agree that the strong phylogenetic signal suggests shared physiological traits, our data indicate that the observed patterns are best explained by non-additive, emergent effects of mixed-resource environments rather than by fixed, clade-specific physiological idiosyncrasies.

As shown in Fig. 3B, the sentinel strain's response in mixed-resource environments deviates strongly from the additive expectation based on single-resource effects (right panel). If increasing suppression were driven solely by compositional effects—such as a declining fraction of fermentable sugars with increasing resource number—responses in mixtures would approximate the mean of the corresponding single-resource conditions. Instead, we observe a systematic intensification of suppression with resource number, indicating non-additive interactions among resources and strains.

To test this directly, we separately analyzed subsets of exclusively facilitative or exclusively suppressive conditions in the new Supplementary Fig. 27 (attached in our previous comment). Even within these restricted subsets, facilitation declined and suppression increased with resource number, mirroring the global trends (Supplementary Figs. 16 and 18). This shows that both effects arise from the same underlying, non-additive process.

Mechanistically, our results support a unifying explanation: increasing resource diversity causes a synergistic, non-additive increase in total resource uptake, leaving fewer

metabolites available in the environment. The distinction between HIC and MIC strains thus reflects quantitative variation in the strength of this shared mechanism rather than fundamentally different physiological strategies.

Beyond the scope of the present study, we now explicitly discuss a potential molecular basis in the revised Discussion text:

"consistent with mounting evidence: microbes exhibit markedly different behavior in mixed-resource environments, including diauxic shifts and lag-phase dynamics[30], and distinct, unexpected gene expression profiles[31]. We speculate that the observed non-additive effects in mixed-resource environments may arise from such enhanced activation of genes involved in nutrient catabolism under mixed resource conditions, a hypothesis we plan to investigate in future work."

6. In addition, the data shows that interactions (unsurprisingly) are context-dependent and can be positive or negative depending on the carbon source. If my understanding is correct that the reported interaction terms in Fig 2 are averaged over multiple carbon sources, this averaging does not seem to be doing the complexity of the dynamics justice and may even be misleading. If I am misunderstanding, then please explain the methodology more clearly.

There are two types of "interaction terms" in Fig.2 (i) interaction coefficients based on fitting (the steady state solution of a) generalized Lotka Volterra equation to our experimental data and (ii) the impact factors. The impact factors are calculated for each strain in each interaction (e.g. there is no global fitting). However, in order to show what we care about (the change of interaction with nutrient diversity) the impact factors for a given number of nutrients is averaged. Showing all of them individually would be quite incomprehensible (3,398 datapoints). This does not oversimplify any dynamics because averaging happens after extracting these values individually.

The interaction coefficients (alpha) are indeed obtained from globally fitting generalized Lotka Volterra equations (respectively their steady state solutions) to community data at 1,8 and 16 carbon sources, whereas each of these "bins" contains different carbon source mixtures. Therefore, we used a Bayesian regression that treats parameters (and variables) as random values of an underlying probability distributions. Bayesian frameworks therefore naturally integrate variability across different single samples - each with different carbon sources, and this is exactly the reason why we used this framework. The returned results are then the averages obtained from the obtained probability distributions (posteriors). Bayesian frameworks allow therefore on the one hand to obtain mean parameters for rather simple models (LotkaVolterra) but allow also

their variation naturally integrating deviations from those simple models as caused by higher dynamical complexity.

With all that being said, we have to point out that the major point of this modelling approach is not to obtain models that simulate the underyling real systems in all detail, but that allow us to observe how interactions shift qualitatively as resource diversity increases. For this approach simple models are not only suitable, they are necessary to be able to present the results (alphas) in a comprehensible way, which would not be possible for highly nonlinear models like neural networks for example.

7. L275: I found the idea of mixing supernatants from individual resources and comparing to supernatants from mixed resources to be very elegant. However, I found the discussion around this point to be confusing and initially found myself wondering whether lower residual key nutrients in the mixed-resource supernatants really imply "increased carbon uptake with greater resource diversity" – on a first read, wouldn't this be equally well explained by reduced excretion of metabolites?

We thank the reviewer for this thoughtful comment. We agree that lower metabolite concentrations in mixed-resource supernatants could reflect either increased substrate uptake or reduced metabolite excretion. In our data, we observed decreases in both supplied and produced metabolites under mixed-resource conditions, indicating more efficient internal metabolism, reduced metabolic overflow, and greater carbon assimilation. Nonetheless, we cannot fully rule out alternative explanations related to the two processes separately (uptake of supplied carbon and metabolism of intermediates). Therefore, for clarity and completeness, we have adjusted the original sentence to distinguish between these processes, which now reads: "*indicating increased carbon uptake and reduced excretion of metabolic by-products*"

SI Fig 19 resolves this somewhat, but it would be helpful to know which are the "supplied" metabolites in each case.

Regarding which metabolites were supplied in each mixture, this information is provided in Supplementary Data 8. Because the experimental design involved many randomized carbon-source combinations, we focus our interpretation on universal patterns across mixtures rather than on each metabolite on its own. For clarity, and following the reviewer's later comments, we have also added a brief description of the set of resources used in this experiment in the corresponding Methods section:

"Cultures were dispensed into 384-well plates containing M9 medium supplemented with either individual or mixed carbon sources, specifically: sucrose, glutamic acid, citric

acid, maltose, mannose, xylose, lysine, ribose, proline, fructose, histidine, and asparagine"

For the produced metabolites, it seems that only in the 4-resource condition and with the HIS is there any difference in metabolites production. Therefore, it would be useful to split the discussion into supplied and produced metabolites. E.g., in the caption of SI Fig 19, the authors talk of "more metabolites remaining in the non-supplied category", but they cannot be remaining if they were produced during the growth (unless I am misunderstanding). Nevertheless, the finding that the sentinel strain grew less in the high resource supernatants is interesting and aligns with the reduction in metabolite concentrations.

We thank the reviewer for recommending a clearer separation between supplied and produced metabolites. To address this, we have revised the text to explicitly distinguish between these two categories and to clarify that "supplied" metabolites refer to the initial resources, whereas "produced" metabolites correspond to compounds secreted during growth: *"This effect was particularly pronounced in highly influenced strains and was observed for both supplied metabolites that were taken up during growth and produced metabolites that were secreted as by-products (Supplementary Fig. 19)."*

In addition, we replaced the ambiguous phrase *"more metabolites remaining"* with *"more produced metabolites detected"* which more accurately reflects the data (caption of Supplementary Fig. 25).

Overall, the key point is that the sentinel strain grew less in supernatants from mixed-resource cultures, indicating that less residual carbon remained available after growth under these conditions—consistent with enhanced overall carbon uptake and more complete substrate degradation.

Modeling:

1. The authors use both Lotka-Volterra models and Consumer-resource models here. Although I do see the practical usefulness of LV to interpret coculture outcomes, I find it confusing and potentially even inappropriate to make such heavy use of a model that does not explicitly model resource diversity when resource diversity is the entire point of this manuscript. With all the data they collected, why not try to fit consumer-resource models to the individual and pairwise cocultures? I feel that measuring metabolic capabilities and interpreting their data in light of niche overlaps could be much more mechanistic and accessible.

We fit a gLV model to our data to obtain insights into the interaction in the community. This close integration of data and model is key in our work. For this purpose gLV is the

perfect choice because its interaction parameters (alphas) can be directly interpreted as interactions (with direction and strenght). As we show in Supplementary Fig. 5 "re-simulating" communities with the obtained interaction coefficients reproduces the experimentally observed results, showing that his modelling approach is able to capture the basic behavior of the studied microbial communities. From these fits we learn that alphas in particular alphas self go down. This may simplify and maybe even oversimplify underlying dynamics, but we are not interested in models that allow quantitative simulations, but use them as a tool to understand qualitative changes of interactions in the system.

On the other hand, no comparable information about interactions could be directly obtained from consumer resource models. Moreover, parameterizing consumer resource models requires measurements of metabolite fluxes in and our of cells and conversion ratios between metabolites within the cells. These parameters are experimentally very hard to obtain and to the best of our knowledge there is no case where a CR model was fully parameterized on actual data.

2. Both the validity and the procedure of fitting the LV model to infer interaction parameters are unclear to me. It makes sense that inverse carrying capacities would represent the a_self and that one could infer the a_other from the pairwise experiments. But several questions remained for me:
a. Is it appropriate to also use the community data, where it is well-known that higher-order effects and dynamic interactions can be hugely important in more complex communities? How are these community data even used during inference?

First, we would like to clarify that "higher-order" interactions can also arise in one- and two-species systems—for instance, when density-dependent growth or nonlinear interactions cannot be adequately described by purely linear models and therefore require higher-order terms.

Second, as described in the manuscript, all available data (single-species, pairwise, and community-level measurements) were indeed used in the Bayesian regression to infer the interaction matrices (α). Several studies have successfully parameterized gLV or related linear models directly from community data, including those using Bayesian approaches, such as:

- Bucci et al., *Genome Biology* (2016), "MDSINE: Microbial Dynamical Systems INference Engine for microbiome time-series analyses."

- Kuntal et al., *Frontiers in Microbiology* (2019), “Web-gLV: A web-based platform for Lotka–Volterra modeling and simulation of microbial populations.”
- Stein et al., *PLOS Computational Biology* (2013), “Ecological modeling from time-series inference: Insight into dynamics and stability of intestinal microbiota.”

The regression was performed on the steady-state values of the communities (as detailed in Supplementary Text 2), where system dynamics have ceased. While fitting a linear model to inherently nonlinear data inevitably simplifies those nonlinearities, our goal here was not to fully capture the complex dynamics of the underlying system. Instead, we aimed to identify broad trends in how interactions change—something a simple linear framework can effectively reveal.

Importantly, we also estimated the interaction coefficients using the analytical steady-state solution of the gLV model applied to single-species and pairwise outcomes. This approach produced comparable results (see Supplementary Fig. 7). Both methods consistently showed the same overall trends.

b. Are the inferred interaction terms for a given pair and condition consistent between the pairwise and community inferences? Are they consistent across individual resources in the single-resource cocultures?

It is not possible to obtain fitted parameters for a gLV model describing a single interaction under just one experimental condition, as the resulting system of equations would be underdetermined—one cannot reliably fit an entire model to a single data point. For this reason, we treated the variation across experimental conditions as reflecting variability in the model parameters, which we captured through Bayesian regression. This approach was chosen because Bayesian methods naturally account for parameter heterogeneity by modeling parameters as probability distributions rather than fixed values.

c. Is inferring interactions using Bayesian regression as done here statistically as well-supported as established methods? Why didn’t the authors use an established method to infer interactions from the community data, such as SparCC or SpiecEasi? To me, the Bayesian regression procedure used here seems like a new method that requires substantial testing against, e.g., simulated controls.

Bayesian regression is a very well established and mathematically well founded method reaching back to the early 19th century. It was also used in the context of microbial interactions on several occasions especially when dealing with intrinsic uncertainties (for example:

- Shafiei, M., Dunn, K.A., Boon, E. et al. BioMiCo: a supervised Bayesian model for inference of microbial community structure. Microbiome 3, 8 (2015);
- *Veronica Vinciotti, Pariya Behrouzi, Reza Mohammadi*, Bayesian Structural Learning with Parametric Marginals for Count Data: An Application to Microbiota Systems, 25(339):1−26, 2024;
- Neal S. Grantham, Brian J. Reich, Elizabeth T. Borer, Kevin Gross, MIMIX: a Bayesian Mixed-Effects Model for Microbiome Data from Designed Experiments, arxiv, 2017,

To make it clearer that we follow here an established traditions we added some of those citations to the main text:

"Therefore, we used ato a generalized Lotka–Volterra (gLV) model using Bayesian regression that naturally allows for its parameters to be distributed (Methods, Supplementary Text 2), in line with similar previous approaches[19,20]."

SparCC returns correlation between species (not interactions) and SpiecEasi returns conditional dependence within a community. Both approaches do not allow to compare self vs other interaction, which is crucial for our analysis.

d. How should a_other values of >10 be interpreted? That seems extremely high to me - could they be artefactual because some carrying capacities are extremely low?

For the *α*_other values obtained using Bayesian regression, no coefficients exceed 10. In contrast, when the interaction coefficients are derived by analytically solving the steady-state gLV equations, higher values can indeed appear (see what is now Supplementary Fig. 9). As explained in the caption of Supplementary Fig. 9, these large values arise from small estimated carrying capacities in those particular cases as the reviewer pointed out correctly:

"A. Distribution of α_{self} values for 1, 8, and 16 carbon sources. Low population densities in single-resource conditions occasionally produced large, noise-sensitive α_{self} estimates. B. This issue was more pronounced for α_{other} values, where small denominators led to extreme values and a broad distribution."

This is one of the reasons why we used a Bayesian Framework for obtain the interaction coefficients.

e. In SI Fig 7, it is stated that cases with relative abundance <0.001 were excluded, which is understandable from a technical perspective, but in reality, does this means that all cases of actual competitive exclusion (where one strain goes to extinction) are removed from consideration here? If that understanding is correct, doesn't this introduce a lot of bias?

The need to remove data points corresponding to near-zero strain abundances is primarily technical in nature, though not absolute. It is not possible to make meaningful statements about strains that are effectively absent from a community. Accordingly, the removal of very low-abundance species is standard practice in sequencing analyses, as extremely low read counts can arise from sequencing noise or statistical artifacts in data processing pipelines—issues that cannot be completely ruled out. This has been discussed, for example, by Chakraborty *et al.* (*Trends in Microbiology*, 2025). In this context, applying a relative abundance cutoff of < 0.001 is quite common.

To acknowledge that such a cutoff can itself introduce bias, we have now added the phrase "which may introduce some bias into the data" to the caption of Supplementary Fig. 7.

Finally, we note that the large variability observed in the α values—largely driven by low species abundances in several samples—was one of the motivations for adopting a Bayesian regression approach. This framework naturally incorporates uncertainty by treating parameters as probability distributions and, importantly, does not require excluding data with low-abundance species.

3. In the LV model simulations, all growth rates are set to 1. However, about 10% of alpha_other are greater than alpha_self, and in those cases the growth rate CAN matter (bistability regime). This should be mentioned, or growth rates should be randomized.

Thanks for pointing this out. We now mentioned this limitation in our revised methods section by adding:
"Since the growth rate may decide for multistable cases in which state the system ends up, this choice may result in different steady states compared to experimentally parametrized or randomized growth rates."

4. In general, I was surprised to see the off-diagonal elements quite large compared to the diagonals, meaning that interspecies interactions are very strong even at high

resource concentrations. It would be good to comment on this result more, especially since the coexistence of many species in the SynComs would suggest small average values of alpha_other.

As pointed out in the manuscript what decides about coexistence of interacting pairs is the conditions alpha_self>alpha_other and not their absolute value. We further found a correlation between both alpha self and other in the sense that alpha other tend to be smaller than the alpha self of the same interaction, which allows for a quite high number of coexistence compared to randomly assigned alpha self and alpha other. We will discuss this trend and its consequences for coexistence in another manuscript.

5. L130: while the interaction matrix inferred for higher resource number reproduces a lower diversity than the one for a lower resource number, this is not evidence that the inferred interactions are valid, as the authors claim (L130-131). It simply means that a higher ratio of other to self interaction strength yields lower diversity outcomes, which is well known.

Supplementary Fig. 5 was intended as a sanity check to confirm that the inferred α values qualitatively reproduce the overall community behavior. We agree that this does not imply the α values are quantitatively exact, but rather that they capture the correct general trends. To reflect this more accurately, we have softened the wording in the main text to read: “suggesting the validity of the inferred interactions” (Instead of “supporting”).

6. The CRM setup has a number of problems:
a. Adding growth rates from individual carbon sources should not be done as is done here; instead, there are two options: the more “traditional” model simply adds the growth rates without cutting off at a maximum growth rate; alternatively, there is a formula for adding growth rates based on proteome allocation in 10.15252/msb.20145537 (extended to more than two resources, e.g., in 10.1101/2024.06.14.599039).

Thank you for this helpful suggestion. Our current model is indeed an adaptation of the traditional model by adding the maximum growth rate. We capped the growth rate by a maximum value to capture scenarios where species growth is no longer limited by resource availability but rather by other factors, such as enzyme reaction rate. However, we agree with the reviewer that adding such a maximum growth rate makes it harder to interpret the dynamics in the model, thus we revised the model to remove this restriction (thus switching back to the “traditional” model) and rerun the simulations. This modification doesn’t lead to noticeable differences as the simulated dynamics almost

always operate in the regime where species' growth rates are limited by resource concentrations.

b. More importantly, the proposed mechanism for the updated CRM in SI text 3 needs to be discussed in more detail. In particular, what possible mechanism would give a decreased Km? Typically, one would assume the Km for a given resource to be fixed and independent of the presence of other resources. A case could be made for reduced carbon catabolite repression when there are several resources around, each with now a lower concentration and therefore weaker potential for CCR, but this would need to be discussed and supported by existing literature.
c. Alternatively, could other mechanisms rather than a decrease in Km explain the results? This would need to be discussed, as well.

The reviewer is absolutely right that a reduced *K*□ is not the only mechanism by which increased resource diversity could benefit highly influenced strains. We do not yet know which specific physiological changes occur in these strains under our experimental conditions, and identifying these mechanisms is an important direction for future work. However, our conclusions regarding biodiversity are rooted in the empirical observation that some strains (highly influenced strains) benefit strongly from increased resource diversity, whereas others (mildly influenced strains) benefit much less, and that this distinction is largely independent of the specific physiological mechanism underlying it.

To demonstrate this, we examined an alternative scenario in which increased resource diversity benefits highly influenced strains by increasing resource assimilation efficiency (*e*), rather than resource affinity (*K*□). The resulting biodiversity patterns remained qualitatively unchanged (Sup Fig. 33, as compared to Sup Fig. 30; also attached here).

Supplementary Fig. 33:

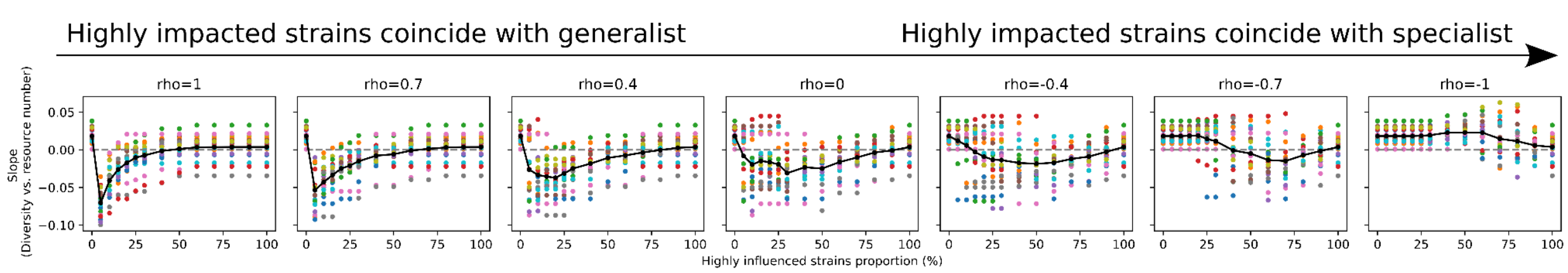

We now explicitly mention this in the main text:
"the results were robust to how strongly and how heterogeneously strains responded to increasing nutrient diversity (Supplementary Figs. 31 and 32), to changes in the size of the species pool (Supplementary Fig. 34), and to the specific physiological mechanism by which resource diversity benefits highly affected strains—whether by allowing access to lower resource concentrations (reduced K□; Supplementary Fig. 30) or by increasing

resource-use efficiency (Supplementary Fig. 33). The latter reflects our limited knowledge of the exact mechanism by which these strains achieve higher carbon uptake; however, our results demonstrate that regardless of the underlying mechanism, the ecological outcome remains the same."

d. In the resource dynamics, the c_ia term is not explained completely: what is a_i and what is the meaning of the min(a_i, r_0,i)/a_i term?

Sorry for missing the definition of a_i, thanks for pointing this out. In our revised model this term is no longer needed because we no longer capped the growth rate with r_0i, but we will offer a brief explanation below.
In our original model where we capped growth rate to a maximum value, the min(a_i, r_0,i)/a_i term is used to correct for resource uptake in scenarios where growth rate of species is no longer limited by resource availability but by the maximum growth rate cap. This requires scaling down resource uptake from the uptake as if the growth rate were not limited by other factors. Here a_i = sum_α(v_iα) is the sum of resource uptake rate if it were not limited by non-resource factors, in which v_iα=v0_iα * R_α / (Km_iα + R_α).

e. In eq. 4 in Supp text 3, c_ia is updated with the new k_m expression, but only in the metabolite excretion term, not the uptake term. Side note: it would probably be easier to read if equation numbering was continuous across the supp texts.

Thanks for pointing out this typo. We indeed updated c_ia in both excretion term and the uptake term. We have modified the equation in supplementary text to correct for that.

Regarding equation numbering, we followed the reviewer's suggestion and revised the Supplementary Material so that all consumer–resource model equations are now consolidated in Supplementary Text 1 and numbered continuously across sections. We believe this change improves readability and clarity.

7. Fig 4E: why is the slope of diversity vs resource number so noisy overall? It also looks like for up to 50% highly influenced strains, one still gets an increase in diversity, contradicting the experiments. The case of 5% HIS seems to give a particularly strong reduction in slope, but given the overall shape of the curve, this seems artefactual without an explanation.

Thanks for the insightful comments. After careful examination of our simulations, we realized that we made a mistake with numerical methods - the error tolerance we set for the ode solver was not enough to guarantee convergence, leading to unbounded growth

and other wrong results. The curve is noisy in our original plot partially due to the above numerical error, and partially because we draw different communities for different fractions of highly impacted strains, this creates high baseline variance in biodiversity among communities, which indeed makes it hard to interpret the results. We now fixed these numerical issues by reducing `atol` (absolute error tolerance) from 1e-3 to 1e-6, and using the same 20 communities for simulations under different conditions. We also made slight modifications of the model according to other comments, and added thorough analyses and discussions of how different factors impact the slope. These changes lead to a different Fig. 4E (attached here) and several new supplementary figures (Supplementary Figs. 28-34).

In our corrected results, we now observe that as the fraction of Highly Impacted Strains (HIS) increases, the slope drops sharply from positive to negative, then gradually recovers back to positive/neutral. This is because as the HIS fraction increases, the competition gradually shifts from among all strains to among highly impacted strains, and when there are only a few highly impacted strains, this competition scope shrinks from all species to a handful of highly impacted strains, leading to the biggest drop in slope. Thus, despite the mistake we made, we expect the strongest drop of slope to happen at a small but non-zero HIS fraction.

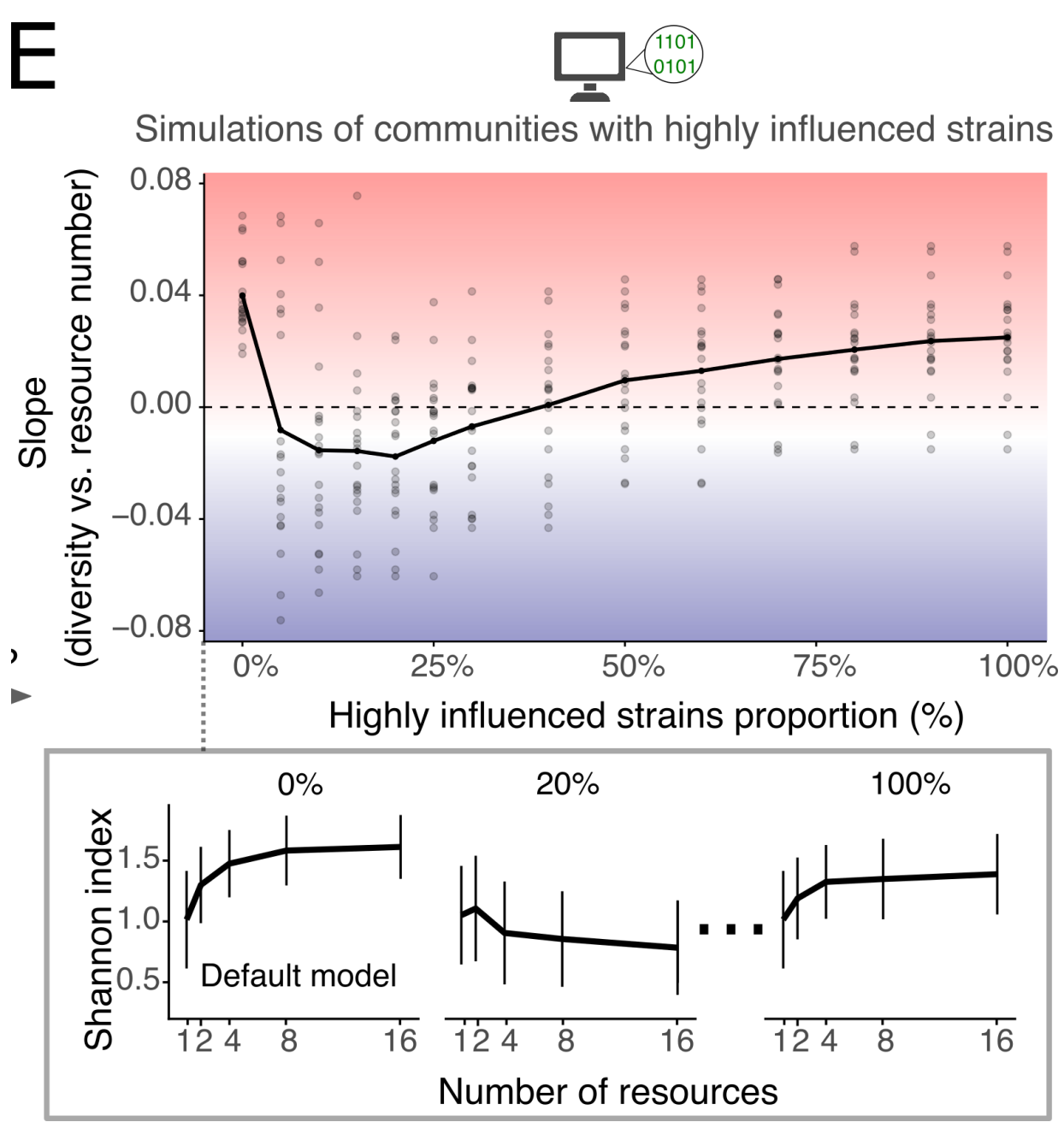

Minor comments:

- Fig 1: How many species were the simulations initialized with? What are the simulation parameters?

We now added more information in Methods for convenience, and extended Supplementary Text to include all the details such as parameters (Supplementary Text 1.3 and 2.3). We also modified Methods in main text and referred to more information about Supplementary Methods and the full code:

"**Consumer-Resource model**
For the classic Consumer-Resource model, we consider a community of 20 microbial species competing for 30 resources in a continuous dilution (chemostat-like) environment. Species take up available resources and either turn them into biomass or transform them into secreted metabolic byproducts (cross-feeding), and die through dilution. Resources are depleted through species uptake, replenished through secretion from species, and are subject to dilution - which replenishes supplied resources and washes out others. For each of 20 independently parameterized communities, we varied resource complexity across 1, 2, 4, 8, 16 supplied resources (equal total supply split evenly among the chosen resources). Initial abundances were uniform (N_0 = 1/20 per species). Simulations used `scipy.integrate.solve_ivp` v1.16.3 in Python v3.14.0 as ODE solver (rtol=1e-3, atol=1e-6, method="RK45"), over t = 0–800 (a.u.) with dilution = 0.1. Cross-feeding is modelled as a metabolic transformation tensor ($b_{i,\beta\to\alpha}$) that allows a fraction of consumed resource β to be secreted as resource α by species i, thereby generating new metabolites available to other species. This formulation follows standard extensions of MacArthur-type models incorporating metabolic by-product exchange and saturating resource uptake kinetics[8,16,51]. For full details, see **Supplementary Text 1 Section 1** and the full code (under 'CR_model_simulations').
For the highly influenced strains modified model version see **Supplementary Text 1 Section 2**. In this updated model, we introduce a species-specific sensitivity parameter γ. When γ>0, the species increases its resource uptake affinity (i.e., reduces K□) as resource diversity increases. We set γ=1 for highly influenced strains (HIS) and γ=0 for mildly influenced strains (MIS) by default. For each of the 20 independently parameterized communities, we vary the highly influenced strain (HIS) fraction f = 0, 0.05, …, 1.0 (14 fractions)(**Fig. 4E**; **Supplementary Figs. 28-34**). To assign the species as either HIS or MIS, we first assigned a tendency to be highly influenced for each species i, which is correlated with resource generalism. We then rank the species by their tendency to be highly influenced, and assign the ones with highest values as HIS according to the preassigned fraction f, and the rest as MIS. We simulated the 20 communities under 14 HIS fractions, across the same five resource complexities (1–16) with equal total supply, t = 0–800, dilution = 0.1, and uniform initial abundances.
We further performed robustness tests by systematically varying key parameters and assumptions. These include (i) cross-feeding rate; (ii) correlation between highly influenced behavior and resource generalism; (iii) γ value for HISs; (iv) a continuous instead of binary distribution of MIS and HIS behavior; (v) an alternative mechanism

where resource complexity influences HISs by increasing assimilation efficiency instead of resource affinity; and (vi) the number of initial species for each community. For full details see **Supplementary Text 1 Section 3, Supplementary Figures 30-34,** and the full code (https://github.com/orshalevsk/Emergent_Biodiv_loss/)**.**"

Supplementary data was not available during review but overall, I believe adding some more information to the main text without having to go the SD for every bit of information would be very helpful for readers: how many strains were combined to form the synthetic communities (only in caption so far), what is the phylogeny of the strains (missing), what resources were used for the metabolomics experiments, etc.?

To avoid redundancy, we added methodological details that were previously in figure captions to the Methods section. The number of strains or communities used in each experiment remains specified in the figure captions, while the Methods section now focuses on the relevant technical procedures. Regarding phylogeny, we have added the complete classification in Supplementary Data 5 and visualized it at the order level in Supplementary Figure 19. As for the carbon sources used in the metabolomics (supernatant) experiments, we now list them briefly in the Methods section: *"Cultures were dispensed into 384-well plates containing M9 medium supplemented with either individual or mixed carbon sources, specifically: sucrose, glutamic acid, citric acid, maltose, mannose, xylose, lysine, ribose, proline, fructose, histidine, and asparagine"*

- L43: the consumer resource model in reference 7 describes animal and plant communities, no microbial communities. More recent references to the microbial CRM would be more appropriate. Crucially, as the authors know, crossfeeding plays a huge role but is missing from the list of additional mechanisms (L47) even though it leads to a diversity of dynamically generated resources.

This is a helpful note. We have now added a citation to the foundational formulation of the consumer–resource model (MacArthur, R. "Species packing and competitive equilibrium for many species." Theor. Popul. Biol. 1, 1–11, 1970; L40), as well as a reference to a more recent extension of the model that explicitly incorporates cross-feeding in microbial systems (Goldford et al., Science, 361, 469–474, 2018; L40). Moreover, we extended the Introduction and Methods sections in the context of cross-feeding, while also acknowledging important works relevant to our conceptual framework:

Intro:
"When species are assigned random metabolic traits and uptake efficiencies, using a realistic framework that also allows metabolic by-product secretion (cross-feeding), the

model predicts that increasing resource diversity allows more species to persist—as naively expected and previously shown by others"

Methods:

"Simulations used `scipy.integrate.solve_ivp` v1.16.3 in Python v3.14.0 as ODE solver (rtol=1e-3, atol=1e-6, method="RK45"), over t = 0–800 (a.u.) with dilution = 0.1. Cross-feeding is modelled as a metabolic transformation tensor ($b_{i,}\beta\rightarrow\alpha$) that allows a fraction of consumed resource β to be secreted as resource α by species i, thereby generating new metabolites available to other species. This formulation follows standard extensions of MacArthur-type models incorporating metabolic by-product exchange and saturating resource uptake kinetics[8,16,51]. For full details, see Supplementary Text 1 Section 1 and the full code (under 'CR_model_simulations')."

- The authors may be interested in this recent preprint that seems to show some similar findings on interactions in multiple resources: https://www.biorxiv.org/content/10.1101/2025.03.30.646244v1.abstract

This is really interesting indeed! Always great to see that ones results are recapitulated. We thank the reviewer for pointing our attention, and also now cite this work and refer to it in our main text: "This interpretation is further supported by a recent study showing that increasing resource diversity synergistically intensify competition among microbes[22]."

- L228: what does it mean to have a "strongly quantitative" response?

The next sentence clarifies the intended meaning by referring to Fig. 3C. To improve the connection, we changed the punctuation from a period to a colon, hopefully making the transition smoother and the meaning clearer:

"The responses of highly and mildly influenced strains were strongly quantitative, scaling with the number of available resources: highly influenced strains started out less suppressive than mildly influenced strains under simple conditions, but became more suppressive as resource complexity increased (Fig. 3C)."

- L358 (Discussion): While it is true that nutrient fertilization can reduce diversity, I found this discussion odd. Firstly, because, as the authors say, this is typically because of the increase in concentration not diversity, and it is not clear that the two scenarios are at all related. Second, their examples are not related to microbes, while their entire approach here and the physiological explanations rely on microbial physiology. Therefore, I would caution against trying to forge too close a link between macroscopic ecosystems and microbial ecology in this context.

This is a helpful critique. First, We have clarified in the revised text that while nutrient fertilization typically increases the concentration of existing resources, in some cases it can also introduce new nutrient types, effectively increasing resource diversity—depending on the soil composition and the fertilizer used. We now make this distinction explicit in the main text: *"More broadly, biodiversity loss due to nutrient enrichment (e.g., fertilization) is frequently observed in forest[23,24], agricultural[25], and aquatic ecosystems[26,27], though these cases may involve increases in nutrient quantity rather than diversity—depending on soil composition and fertilizer type."*

Moreover, we agree that mechanistic parallels between macroscopic and microbial ecosystems should be drawn with caution. Accordingly, we added a clear disclaimer noting that this analogy is meant only as a conceptual comparison, and nevertheless decided to retain this parallel, as it may help stimulate new hypotheses and ideas regarding macroscopic environments: "*This demonstrates that resource complexity itself can be sufficient to drive higher competition, although whether such a mechanism operates in macroscopic ecosystems remains unknown."*

- Typo: SI Fig 17 caption, 3D -> 3B
Thank you!

- L383: A personal point, but I find the framing of discussions of microbial existence as a "paradox" despite "competition for the same resources" to be outdated – as the authors themselves say, there are so many theoretical explanations for any number of species coexisting on small numbers of supplied resources that, at least to me, there simply is no paradox. However, this does not invalidate the search for patterns in diversity across environments and trying to explain them, including the work in this manuscript, at all – it is simply a matter of framing.

Agreed. Changed to a neutral "*question*" instead of "*paradox*".
Reviewer #1 (Remarks on code availability):

Code is available, extensive, and well-annotated. However, I did not try to run it.

Reviewer #2 (Remarks to the Author):

This manuscript presents an ambitious combination of modeling, multi-omics, and experimental approaches to study microbial community assembly. The integration of these methodologies is a strength, the data is rich and could potentially yield significant

insights. However, I find that the work does not fully substantiate its claims. Specifically, the modeling is underdeveloped, the experimental methods lack critical detail and justification, and while concision is a strength, important theoretical and empirical literature is not discussed.

Below is a list (not exhaustive, but I believe sufficient) of major issues I have identified with this manuscript at its current stage.

Major points

The consumer–resource (CR) framework used here is not developed in sufficient depth. CR models incorporating cross-feeding are now considered state of the art in microbial ecology, yet cross-feeding is not included or discussed.

More broadly, cross-feeding is widely recognized as central to microbial community assembly, yet it is not even mentioned in the manuscript.

Thanks for the insightful comment! We apologize for providing an outdated and incomplete “Parameter Values” section in supplementary methods, which wrongly stated “By default we set e=1 so that resources R are fully converted to biomass N, and there is no cross-feeding”. In reality, we draw e - the fraction of consumed carbon source converting to biomass - from [0, 1], and the rest (1-e) are secreted as alternative metabolites that may cross feed other species, resulting in a cross feeding rate between 100%-0%. More specifically, we modelled cross-feeding via the resource transformation tensor (*b*, see Supplementary Text 1), which allows a fraction of consumed resources (1-e) to be secreted as alternative metabolites available to other species. This formulation is conceptually similar to those used in recent cross-feeding models (e.g., Posfai et al., PNAS 2017; Marsland et al., PNAS 2019; now cited).

In fact, without cross-feeding, low resource diversity can only maintain low biodiversity, leaving little room for biodiversity to drop as resource diversity increases and making it unlikely to observe negative slopes. To recognize this important role cross-feeding plays, we now added additional simulations where we examined biodiversity patterns under different average cross-feeding rates (0, 20%, 40%, 60% and 80%) and showed that cross-feeding makes it more likely to observe biodiversity loss as resource diversity increases (Supplementary Fig. 30; attached here).

We have revised the Methods and Supplementary Text 1 to reflect the correct parameter values used in the simulations, to include the additional simulations, and to clarify that cross-feeding was explicitly incorporated into the model:

“For the classic Consumer-Resource model, we consider a community of 20 microbial species competing for 30 resources in a continuous dilution (chemostat-like) environment. Species take up available resources and either turn them into biomass or

transform them into secreted metabolic byproducts (cross-feeding), and die through dilution. Resources are depleted through species uptake, replenished through secretion from species, and are subject to dilution - which replenishes supplied resources and washes out others. For each of 20 independently parameterized communities, we varied resource complexity across 1, 2, 4, 8, 16 supplied resources (equal total supply split evenly among the chosen resources). Initial abundances were uniform (N_0 = 1/20 per species). Simulations used `scipy.integrate.solve_ivp` v1.16.3 in Python v3.14.0 as ODE solver (rtol=1e-3, atol=1e-6, method="RK45"), over t = 0–800 (a.u.) with dilution = 0.1. Cross-feeding is modelled as a metabolic transformation tensor ($b_{i,\beta\rightarrow\alpha}$) that allows a fraction of consumed resource β to be secreted as resource α by species i, thereby generating new metabolites available to other species. This formulation follows standard extensions of MacArthur-type models incorporating metabolic by-product exchange and saturating resource uptake kinetics[8,16,51]. For full details, see ***Supplementary Text 1 Section 1*** *and the full code (under 'CR_model_simulations')."*

Also, to avoid overinterpretation of our cartoon in Fig. 1A, we have revised the figure caption to clarify its schematic nature. *"This schematic is simplified for illustrative purposes; in reality, microbial communities also exhibit cross-feeding interactions and varying niche breadths, which are not depicted here."*

We agree that cross-feeding should be mentioned in the main text, as it is a central process in microbial community assembly, and have therefore revised the main text to acknowledge it:

#1:

"When species are assigned random metabolic traits and uptake efficiencies, using a realistic framework that also allows metabolic by-product secretion (cross-feeding), the model predicts that increasing resource diversity allows more species to persist—as naively expected and previously shown by others"

#2:

"It is further promoted when the cross-feeding rate is high and communities already sustain high diversity under a single resource (Supplementary Fig. 30, compare across rows)"

Supplementary Figure 30:

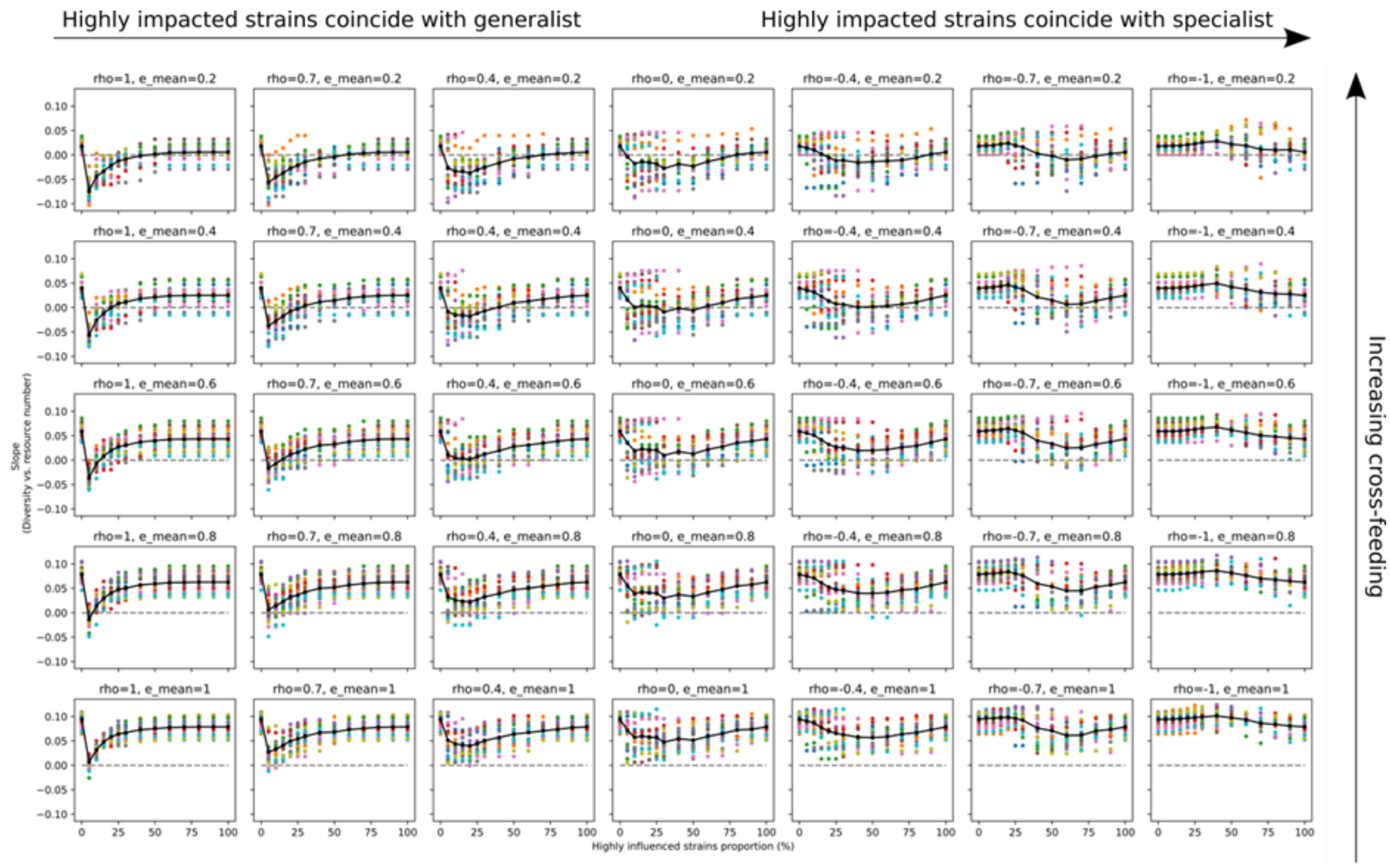

Modeling results are not fully unpacked. For instance, the saturating curve in the CR model is presented without explanation.

We thank the reviewer for pointing this out, and we completely agree. In line with this and other comments, we added new simulations to systematically explore the behavior of the consumer–resource model across a wide range of conditions and identify when increasing resource diversity leads to biodiversity loss (Supplementary Figs. 30-34).

Specifically regarding the saturating curve, the Shannon diversity of species does not saturate with increasing resource diversity; rather, the curve appears to plateau because of diminishing marginal returns at high resource numbers. The reviewer is correct that in the consumer–resource model, each additional resource could, in principle, always open a new niche and permit one more coexisting species. However, when many resources and species are already present, two effects make such gains in biodiversity increasingly rare and small. First, the probability that a newly added resource constitutes a real independent limiting axis is low—existing species can also exploit the new niche and a new species must be the best competitor for this niche to be able to invade. Second, even if a new specialist does invade, the new resource contributes only a small fraction of the total resource supply, so the invader's equilibrium abundance is small. Because Shannon diversity H=-sum_i(pi*ln(p_i)) weights species

by their relative abundances, adding a rare species increases it only slightly. These two forces—lower chance of creating a novel limiting niche and the minimal abundance share of any species that does—produce an apparent plateau without implying a hard ceiling on diversity. That said, a hard ceiling does exist as our simulated communities contain only 20 species, thus a theoretical maximum of H=-ln(1/50)=2.99, the apparent plateau we observed is around 1.5-1.7 and below this hard limit. We now explicitly explain in the main text why the diversity–resource relationship saturates in the Consumer–Resource model: *"the model predicts that increasing resource diversity allows more species to persist—as naively expected and previously shown by others (Fig. 1B; Supplementary Fig. 1A-B). This relationship saturates at high resource numbers because increasing niche overlap limits how many species can stably coexist."*

And added the following discussion about model findings:
*"To identify when increasing resource diversity leads to biodiversity loss, we systematically explored the behavior of the consumer–resource model across a wide range of conditions. This analysis revealed three main factors that strongly shape the likelihood of biodiversity decline. Biodiversity loss is most likely when only a small but non-zero fraction of strains disproportionately benefits from nutrient diversity, allowing these strains to dominate at high resource complexity (**Fig. 4E**; **Supplementary Fig. 30**). It is further promoted when the cross-feeding rate is high and communities already sustain high diversity under a single resource (**Supplementary Fig. 30**, compare across rows), and when strains that benefit most from nutrient diversity are ecological generalists rather than specialists (**Supplementary Fig. 30**, compare across columns). In contrast, many other model assumptions had little qualitative effect on the biodiversity pattern: the results were robust to how strongly and how heterogeneously strains responded to increasing nutrient diversity (Supplementary Figs. 31 and 32), to changes in the size of the species pool (Supplementary Fig. 34), and to the specific physiological mechanism by which resource diversity benefits highly affected strains—whether by allowing access to lower resource concentrations (reduced K□; Supplementary Fig. 30) or by increasing resource-use efficiency (Supplementary Fig. 33). The latter reflects our limited knowledge of the exact mechanism by which these strains achieve higher carbon uptake; however, our results demonstrate that regardless of the underlying mechanism, the ecological outcome remains the same. Larger species pools mainly narrowed the conditions under which biodiversity loss occurred, without altering the underlying mechanism."*

Recent theoretical developments which might be highly relevant to this work are not or barely discussed (e.g. Cui, Marsland and Mehta PRL 2020; dal Bello 2020 Nat Eco Evo).

We thank the reviewer for this valuable comment. We have now added references to recent theoretical developments by Cui, Marsland, and Mehta (Phys. Rev. Lett., 2020) and Dal Bello et al. (Nat. Ecol. Evol., 2020) in the revised manuscript, specifically in the Methods section where we discuss cross-feeding dynamics:

“Simulations used `scipy.integrate.solve_ivp` v1.16.3 in Python v3.14.0 as ODE solver (rtol=1e-3, atol=1e-6, method=”RK45”), over t = 0–800 (a.u.) with dilution = 0.1. Cross-feeding is modelled as a metabolic transformation tensor ($b_{i,\beta\rightarrow\alpha}$) that allows a fraction of consumed resource β to be secreted as resource α by species i, thereby generating new metabolites available to other species. This formulation follows standard extensions of MacArthur-type models incorporating metabolic by-product exchange and saturating resource uptake kinetics[8,16,51]. For full details, see Supplementary Text 1 Section 1 and the full code (under ‘CR_model_simulations’).”

Regarding more specific theoretical developments, we now emphasize in the revised text and analyses the work of Pacheco *et al.* on consumer–resource model extensions addressing generalism. We also reanalyzed our data in light of their model predictions and included corresponding new analyses. We consider this framework most closely related to ours, as it provides a theoretical explanation for the weaker-than-expected increase in biodiversity with the number of resources—closely aligning with our own focus on cases where biodiversity actually decreases.

The revised text now reads:

“*We tested whether the mechanism proposed by Pacheco et al.[16] could map onto our results. Their work suggested that higher resource generalism leads to stronger competitive advantage and dominance at elevated resource diversity, reducing coexistence through increased niche overlap. Strain 79, despite its strong biodiversity impact, exhibited only moderate niche breadth compared with the other community members, indicating that its dominance cannot be explained by generalism alone (Supplementary Fig. 15).”*

And also:
“*Because a previous study (Pacheco et al.[16]) linked resource generalism to higher competitiveness under increasing resource diversity, we next examined whether generalism could underlie the enhanced competitiveness of highly influenced strains. Generalist strains were moderately enriched among highly influenced types ($r = 0.36$, $p < 0.001$; $\kappa = 0.36$; Jaccard = 0.43; Supplementary Fig. 22), suggesting that broad niche breadth may facilitate competitive dominance at higher resource numbers. However, this mild correlation was insufficient to explain the pronounced increase in suppression*

observed under higher resource diversity, indicating that additional mechanisms must drive the stronger competitive responses of highly influenced strains."

Lastly, we examined how strain generalism interacts with highly influenced strains to affect biodiversity loss in our simulations, as shown in the new Supplementary Fig. 30.

The discussion of the latter paper in particular, which studied nearly the same question, is minimal. Especially given that the conclusions of that paper differ quite a bit from what is claimed here, yet that contrast is not explored.

We fully agree that Dal Bello et al. is a highly relevant contribution, but its approach differs fundamentally from controlled synthetic-community experiments such as those of Pacheco et al. and our own. Dal Bello et al. analyzed natural soil microcosms containing thousands of taxa, where community outcomes primarily reflect environmental filtering from a vast initial species pool rather than interactions among defined isolates. In contrast, Pacheco et al. (and our approach) used a fixed and well-characterized strain set, minimizing such filtering and isolating the effects of resource-driven interactions on biodiversity.

We have clarified this distinction in the revised text (attached), explicitly noting that while Dal Bello et al. provide compelling ecological evidence that resource heterogeneity shapes community composition, their approach and findings differ from those of Pacheco et al. This revision better emphasizes the scarcity of systematic tests in defined synthetic consortia—the gap our study aims to address.

Attached is our revised version (Introduction):
"Dal Bello et al.[15] found a positive relationship between resource and microbial diversity in microcosms. However, these experiments involved a single, complex, and undefined soil-derived community containing thousands of taxa. When transferred to laboratory conditions, such diverse natural communities often collapse to a few dominant species, as observed in this study, indicating strong environmental filtering that may mask microbial interactions. In contrast, only one study—by Pacheco et al.[16]— examined how increasing resource diversity affects diversity in a defined microbial community, finding only a weak biodiversity increase, much weaker than that observed by Dal Bello et al. or predicted by classical consumer–resource models. However, as in Dal Bello et al., the use of a single community limits the generalizability of these findings."

And also (Discussion):

"This extends previous experimental work, which reported weaker-than-expected diversity increases with additional resources but not a decline[16,17]. Compared with Pacheco *et al.*[17], who used a similar defined-community design, our inclusion of multiple

communities uncovered a broader range of outcomes, including consistent diversity reductions. The much lower-than-expected increase observed by Pacheco et al. already hinted that the relationship between microbial biodiversity and resource diversity is not as simple or positive as classical consumer–resource theory suggests. The progression from weakly positive to negative responses observed here underscores that this relationship is not universal but instead depends strongly on community composition and interaction structure. Our results thus highlight that resource enrichment can intensify competitive exclusion, challenging the assumption of a general positive link between resource and species diversity. This interpretation is further supported by a recent study showing that increasing resource diversity synergistically intensify competition among microbes[22]."

Many important parts of the experimental design and approach are either absent, or only concisely specified. Where do the strains come from, why are they suitable for answering this question, how were the 22 resources selected?

We thank the reviewer for this important comment and have expanded the Methods section to clarify the origin and rationale of our strain collection as well as the rationale for choosing the 22 resources:

"Resources and their Mixtures
*A total of 22 individual resources were used (**Supplementary Table 1**). These resources were selected to encompass a broad spectrum of microbial growth substrates, including sugars, amino acids, organic acids and alcohol-sugars, thereby capturing the main metabolic classes that distinguish "sugar specialists" and "acid specialists" commonly observed among heterotrophic bacteria[33]. Resources were dispensed into 384-well plates containing M9 media without carbon sources using an acoustic liquid handler (Echo 525, Beckman) prior to microbial inoculation. For all resource conditions (individual or mixtures), a final concentration of 0.2% (mass/volume) was maintained. We used concentration (mass/volume) rather than molarity to keep the total available energy comparable across resources of different molecular weights, consistent with previous resource-diversity studies[15,16,34]. Mixture conditions ranged from 2 to 16 resources and were assigned randomly."*

"Strain collection in sentinel strain experiments
For the sentinel strain experiments, we used 298 bacterial strains isolated from the gut of Caenorhabditis elegans collected from diverse natural environments, including rotting fruits, soil, and compost (Supplementary Data 5). Strains were provided by Dr. Christoph Ratzke and Prof. Hinrich Schulenburg. This collection spans a broad range of bacterial taxa (Supplementary Data 5; Supplementary Fig. 19) and represents a highly

diverse and ecologically relevant set of C. elegans–associated microbes suitable for controlled experimental studies."

As for why the strains in our collection are suitable to address the research question, the 298 bacterial strains used span a broad range of taxa, covering major phylogenetic lineages: new taxonomic analyses now provided in Supplementary Fig. 19 (attached here), and in Supplementary Data 5. While no experimental system can capture the full diversity of natural microbiomes, we believe that this collection represents an exceptional level of phylogenetic and ecological diversity for controlled experiments, thereby providing strong ecological relevance.

Supplementary Fig. 19 (order-level taxonomy):

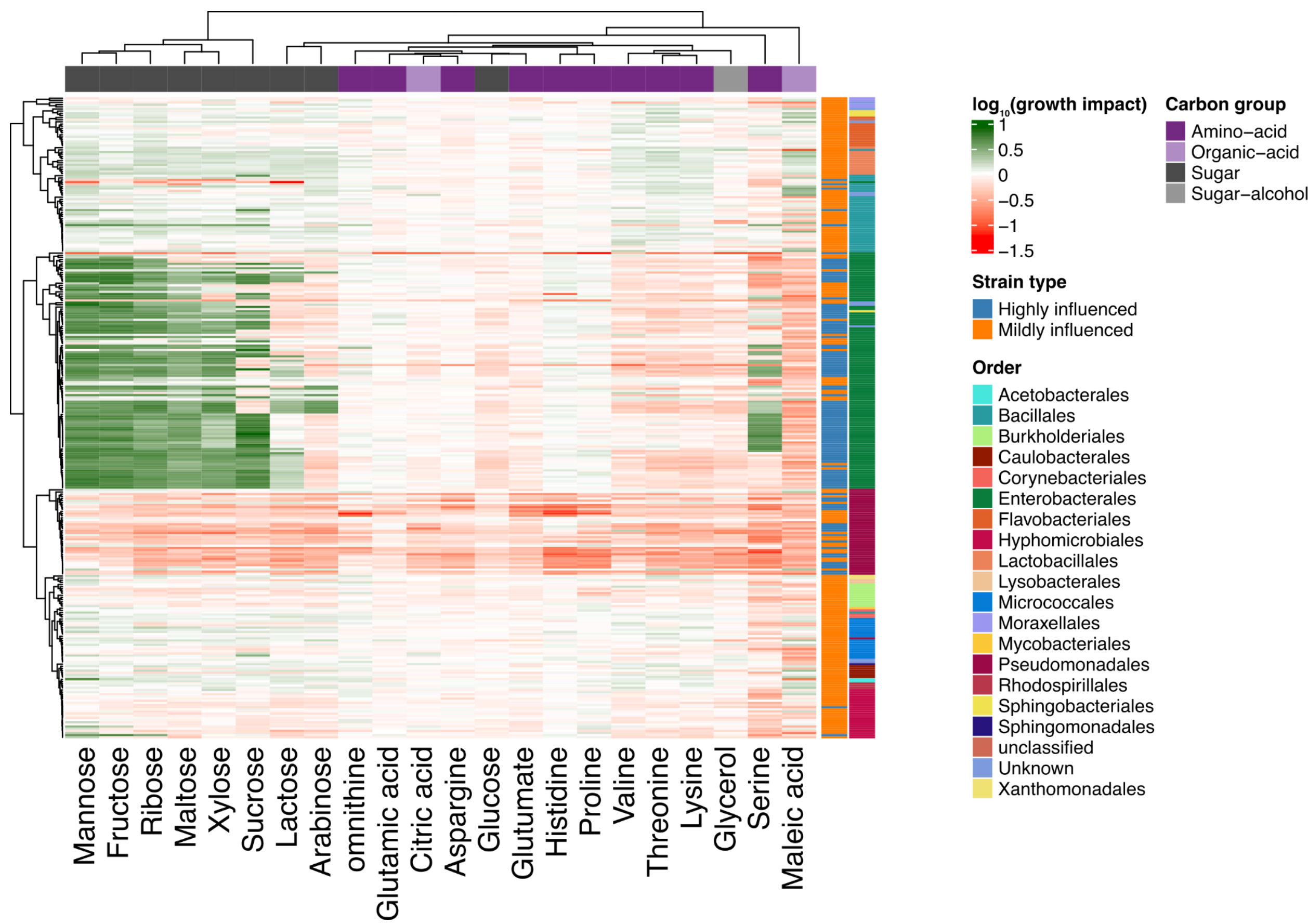

The choice to maintain all resources at 0.2% mass/volume is problematic. Different resources differ in molecular weight and carbon content, meaning mixtures vary substantially in total available carbon and per-resource contribution. This affects carrying capacities, which are fundamental for interpreting both monoculture and co-culture dynamics (e.g. $\alpha\_self = 1/K$).

We thank the reviewer for this helpful comment and agree that this is an important point that should be clarified in the text. We have revised the Methods section accordingly:

"We used concentration (mass/volume) rather than molarity to keep the total available energy comparable across resources of different molecular weights, consistent with previous resource-diversity studies[16,17,36]."

We respectfully note that maintaining all resources at 0.2% (w/v) is not problematic but an intentional normalization strategy. Microbial growth is primarily constrained by the total mass of available carbon, rather than the number of substrate molecules. For example, the same number of sucrose molecules provides roughly twice the energy of glucose molecules due to their higher molecular weight. By normalizing carbon input by mass, we ensured that each condition—single or mixed—contained a comparable potential energy supply.

This approach follows previous resource-diversity experiments that compared environments of differing resource complexity using fixed weight concentrations (*Dal Bello et al.*, *Nat Ecol Evol* 2021; *Pacheco et al.*, *Nat Commun* 2021) and aligns with other high-throughput microbial community studies employing similar normalization (*Kehe et al.*, *Science Advances* 2021).

For the sake of the rebuttal, we also calculated the Gibbs energy for each molecule at 0.2% in 40 ul volume, to ensure that it is indeed similar, using eQuilibrator (https://equilibrator.weizmann.ac.il/). As can be seen, the approximated Gibbs energy for 2 g/L (0.2%) in 40 ul among the resources is very similar:

Compound	Formula	Carbons	Molecular weight (g/mol)	Moles in 40 µL at 2 g/L	Approx ΔG° for 40 µL amount (kJ)
Asparagine	C4H8N2O3	4	132.12	6,06E-04	-1,04E+00
Glutamic acid (Glutamate)	C5H9NO4	5	147.13	5,44E-04	-1,17E+00
Histidine	C6H9N3O2	6	155.15	5,16E-04	-1,33E+00
Lysine	C6H14N2O2	6	146.19	5,47E-04	-1,41E+00
Ornithine	C5H12N2O2	5	132.16	6,05E-04	-1,30E+00
Proline	C5H9NO2	5	115.13	6,95E-04	-1,49E+00
Serine	C3H7NO3	3	105.09	7,61E-04	-9,82E-01
Threonine	C4H9NO3	4	119.12	6,72E-04	-1,16E+00
Valine	C5H11NO2	5	117.15	6,83E-04	-1,47E+00
Arabinose	C5H10O5	5	150.13	5,33E-04	-1,27E+00
Citric acid	C6H8O7	6	192.12	4,16E-04	-1,19E+00
Fructose	C6H12O6	6	180.16	4,44E-04	-1,27E+00
Glucose	C6H12O6	6	180.16	4,44E-04	-1,27E+00
Glycerol	C3H8O3	3	92.09	8,69E-04	-1,25E+00
Lactose	C12H22O11	12	342.3	2,34E-04	-1,34E+00
Maleic acid	C4H4O4	4	116.07	6,89E-04	-1,32E+00
Maltose	C12H22O11	12	342.3	2,34E-04	-1,34E+00
Mannose	C6H12O6	6	180.16	4,44E-04	-1,27E+00
Ribose	C5H10O5	5	150.13	5,33E-04	-1,27E+00
Sucrose	C12H22O11	12	342.3	2,34E-04	-1,34E+00
Xylose	C5H10O5	5	150.13	5,33E-04	-1,27E+00

Importantly, we would like to clarify that our analysis does not focus on the absolute nutrient amounts themselves, but rather on how the community's behavior changes when different nutrients are mixed. For example, we compare growth on 1 unit of nutrient A and 1 unit of nutrient B to growth on a 1:1 mixture containing 0.5 units of A and 0.5 units of B. In this context, the specific unit of measurement is not critical—what matters is that each nutrient amount of each nutrient is halved when combined in a 1:1 mix.

The relationship between carrying capacities and interaction coefficients is not addressed.

We thank the reviewer for this useful observation and have clarified this point in the text. In our generalized Lotka–Volterra formulation (Supplementary Text 2), the self-interaction coefficient (α_self) corresponds to the inverse of the carrying capacity (K). This formulation directly links experimentally observed monoculture growth limits (K) to the model's intraspecific inhibition term (α_self), following a standard approach in implementations of the gLV model (MacArthur & Levins, Theor. Popul. Biol. 1970; Venturelli et al., Nat Commun 2018).

We now explicitly state this relationship in the revised Methods section and refer the reader to Supplementary Text 2 for the detailed model formulation. For completeness we add the fully revised section of the gLV, marking this specific additon:

"Inferring interaction matrices from experimental data using the generalized Lotka–Volterra model

Data from single-species growth (Supplementary Fig. 7Xxx), pairwise interactions (Fig. 2A) and community compositions (Fig. 1D, E and Supplementery Fig. 3) at steady state were used to infer Lotka-Volterra interaction matrices of the 14 strains comprising these communities. Interaction matrices were constructed for three datasets corresponding to 1, 8, and 16 available carbon sources. Since each of these three datasets contains data for multiple different nutrient compositions, we treated interaction strength as a random variable by using Bayesian regression to infer their values. The steady state of the generalized Lotka–Volterra system is obtained by setting the equations in Eq. 1 (main text) to zero, taking into account only those species whose population densities remain non-zero.The obtained equations where fit to the data with Bayesian regression (Monte Carlo Markov Chain sampling, NUTS solver in pyro, python). As priors we used Gaussian distributions with a mean of 1 for the off-diagonal entries and a mean equal to the inverse carrying capacity for the diagonal entries. <u>This is based on the fact that the self-interaction coefficient (α_self) corresponds to the inverse of the carrying capacity (K), a standard notation of generalized Lotka-Volterra models</u>[31,52]. Posterior sampling was performed with 30 chains, 400 warm up steps and 200 sampling steps. For full

details, see Supplementary Text 2. Note that error bars for αself in Figure 2B (middle) may be overestimated as the interaction values are not independent (see Supplementary Fig. 6A)."

The computation of the "impact factor" is unclear. The text provides one definition (2×α_self + 2×α_others), while Figure 2 states a different definition based on OD600 values. Line 155 refers to "Methods," but this is absent from the Methods section.

We apologize for the confusion and agree that the definition of the "impact factor" required clarification.

The "impact factor" used in Figure 2 is based on experimentally measured OD_{600} values (population densities at steady state) and the relative abundances obtained from 16S sequencing of pairwise interactions, and quantifies the total inhibitory effect on the sentinel strain as the relative reduction in growth compared with monoculture controls (as illustrated in Figure 2A). With the expression *(2 × α_self + 2 × α_others)* we intended to indicate that the impact factor depends on four interaction terms (α), representing two self-inhibiting and two mutually inhibiting effects between the two interaction partners. However, we agree that this notation was confusing. We therefore calculated the exact expression for this impact within a generalized Lotka–Volterra (gLV) framework and added this information to the main text and Methods as follows:

Main: *"To identify which strains drive biodiversity loss, we defined an 'impact factor'—which equals the growth of a strain in the presence of an interaction partner divided by the growth without it. In context of a Lotka Volterra framework this impact factors depends on all the four alphas of a pairwise interaction (two α_{self} and two α_{others})."*

Methods: *"The impact factor was computed from experimental OD_{600} data as an empirical measure of total inhibition acting on the sentinel strain. It was defined as the relative reduction in OD_{600} compared to monoculture controls (Figure 2A). In the framework of generalized Lotka-Volterra model this impact factor corresponds to the steady state population density of species 2 in the presence of species 1 divided by the steady state population density of species 2 grown alone, which equals* $\alpha22(\alpha11 - \alpha21)/(\alpha11\alpha22 - \alpha12\alpha21)$*"*

Metabolic strategies, e.g. diauxie/coutilization and resource preferences, should be central to the observations, yet they are poorly discussed. In particular, there are known metabolic determinants of whether resources are sequentially or simultaneously uptaken (e.g. see Wang et al Nat communications 2019).

We thank the reviewer for highlighting the importance of metabolic strategies such as diauxie and co-utilization. Both experimental and theoretical studies have shown that diauxic shifts can promote coexistence by creating temporal niche partitioning (Bloxham et al., 2022, *Mol Syst Biol*; Bloxham et al., 2024, *PLoS Comput Biol*). However, diauxic shifts occur only when multiple nutrient sources are available, and thus would be expected to enhance biodiversity as resource richness increases, beyond the niche-expansion effect alone. This makes our finding—that biodiversity commonly decreases with increasing resource diversity—even more surprising. To highlight this, we added the following sentence to the main text, in the context of Introduction:

“This relationship saturates at high resource numbers because increasing niche overlap limits how many species can stably coexist. The positive relationship between resource diversity and biodiversity may be further reinforced by diauxic shifts, which promote species coexistence in mixed-resource environments[10].”

And in the context of our discussion, regarding the physiology of the effects we obsereved:

“consistent with mounting evidence: microbes exhibit markedly different behavior in mixed-resource environments, <u>including diauxic shifts and lag-phase dynamics</u>[30], and distinct, unexpected gene expression profiles[31].”

The major findings of Wang *et al.* (2019, *Nat Commun*) showed that (1) when combining two resources from the sugar-generating group (Group A), diauxic shifts are likely to occur; (2) when combining two resources from the amino acid–generating group (Group B), both diauxic shifts and co-utilization can occur; and (3) when combining resources from Groups A and B, co-utilization tends to dominate. Connecting this to our study, increasing resource diversity would thus be expected to favor co-utilization over strictly sequential (diauxic) uptake. Because co-utilization produces synergistic, non-additive effects among resources, whereas diauxic shifts lead to primarily additive outcomes, it is plausible that our observed non-additive increase in resource uptake with greater resource diversity reflects a higher prevalence of co-utilization.

To test this hypothesis, we re-analyzed interactions with the sentinel strain in the supernatnat experiment, focusing only on these two groups which is now added in Supplementary Figure 27 (attached here). We found strong evidence that interaction strength became more inhibitory within Group A (sugars; ‘only facilitative conditions’) as resource number increased—an unexpected result given that diauxic shifts should reduce competitiveness (Supplementary Figure 27B). In contrast, we lacked sufficient data to robustly assess Group B (amino acids; ‘only suppressive conditions’;

Supplementary Figure 27A), as filtering for the relevant data left us with only mixtures of two resources. These did not differ from individual conditions, but as also seen for Group A (Supplementary Figure 27B), effects emerged only in 4-resource mixtures, not in pairs.

Although we cannot conclusively distinguish between co-utilization and diauxic shifts within the framework of Wang et al. (2019, Nat Commun) due to insufficient data for Group B, our results rule out the expectation that diauxic shifts would reduce competition. Our results are largely composed of mixtures combining Groups A and B. In such cases, co-utilization is likely predominant (concluded by Wang et al.) and could, in principle, explain part of the observed non-synergism. However, since we observe clear synergistic effects within Group A alone, co-utilization alone is unlikely to be the main explanation for the patterns we report.

We added our analysis to the figure caption of Supplementary Figure 27:

"Notably, although no resource classes were imposed a priori, only-facilitative conditions (B) predominantly correspond to fermentable sugars, whereas only-suppressive conditions (A) primarily correspond to amino acids, consistent with co-utilization classes described by Wang et al. (2019, Nat. Commun.). Increasing resource number led to reduced facilitation and stronger inhibition, particularly in higher-order mixtures. These patterns are inconsistent with a diauxic-only interpretation, which would predict weaker competition as resource number increases"

Supplementary Figure 27:

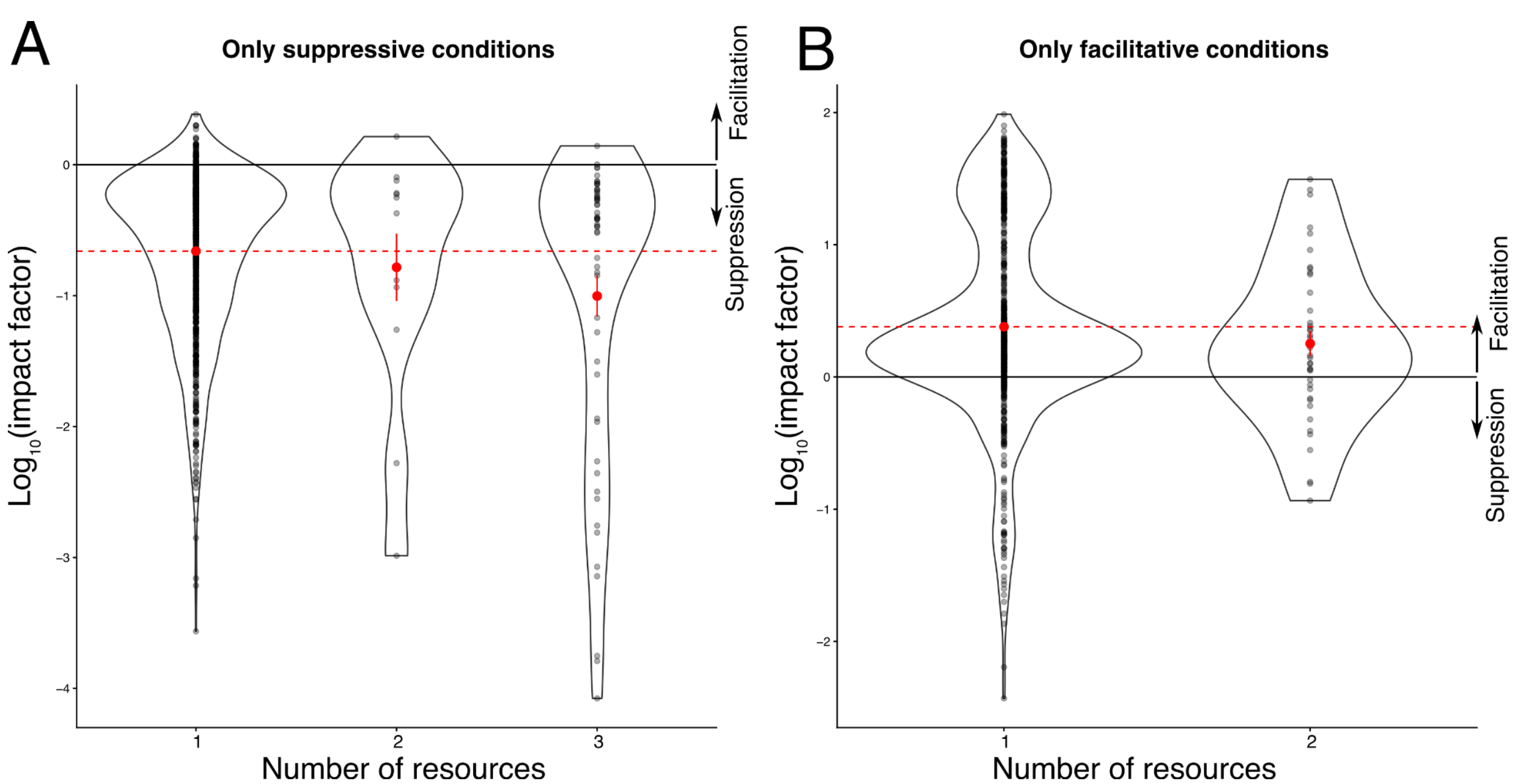

In addition, potential biases derived from using a Pseudomonas sentinel strain are not discussed.

We thank the reviewer for raising the question of whether the choice of *Pseudomonas aeruginosa* PAO1 as the sentinel strain could influence our conclusions.

We agree that any single sentinel strain introduces some bias. We selected *P. aeruginosa* PAO1 because it allows stable chromosomal integration of the *lux* reporter via the *tn7* system, ensuring consistent single-copy expression; it is a metabolic generalist with broad niche breadth; and it grows robustly at 30 °C, matching the growth range of our strain collection. These practical and biological properties make PAO1 a suitable and reliable sentinel for quantifying competitive balance across diverse resource conditions. We now refer to this rationale directly in the Methods text:
"*Sentinel strain selection rationale*

We selected Pseudomonas aeruginosa PAO1 as the sentinel because it supports stable, single-copy chromosomal integration of the luxCDABE reporter via the mini-Tn7 system—ensuring consistent reporter dosage, avoiding plasmid-borne variability, and yielding a strong, reliable bioluminescence signal compatible with our plate-reader workflow. PAO1 grows robustly at 30 °C—matching the growth range of our strain collection—and exhibits broad metabolic niche breadth (generalist physiology), so its growth is sensitive to a wide range of changes in metabolic space. These practical and biological properties make PAO1 a reliable readout strain for quantifying competitive balance across diverse resource conditions. We note that any single sentinel may introduce bias."

Importantly, the trends observed for the 298 strains using PAO1 are generally independent of sentinel identity, supported by two lines of evidence. First, GC–MS supernatant experiments—conducted without a sentinel—recapitulate the increased resource uptake and stronger competitive environment under mixed-resource conditions, with a stronger effect among highly influenced (HIC) strains. This confirms that the HIC/MIC distinction identified via PAO1 reflects a general biological property rather than a sentinel-specific artifact. We now refer to this in the revised main text:
"As both the sentinel-independent (GC–MS results; Fig. 4B) and sentinel-dependent (suppression assays; Fig. 4C) analyses yield consistent results, this indicates that our key findings (Fig. 4C; Supplementary Figs. 16–23) are generally sentinel-independent."

Together, these results demonstrate that the responses of the 298 strains to increasing resource diversity are largely independent of any properties unique to PAO1.

Fig S4 says "As resource number increases, interaction coefficients decrease for both intra-species (diagonal) and inter-species interactions (off-diagonal)" However, whether the figure supports this conclusion is hard to tell visually.

We agree that the change is somewhat difficult to discern in the heatmaps. However, the shift from yellow to blue tones indicates a decrease in interaction coefficients. We would nevertheless like to retain Supplementary Fig. 4, as it illustrates the overall structure of the interaction matrix. The change in interaction strength is shown more clearly in Fig. 2B and Supplementary Fig. 6. We have extended the caption of Supplementary Fig. 4 to include references to these figures for clarity:

"As resource number increases, interaction coefficients decrease for both intra-species (diagonal) and inter-species interactions (off-diagonal). See also Fig. 2B and Supplementary Fig. 6."

Discussion: "our finding reopens a fundamental question: how is microbial coexistence maintained despite competition for the same resources?" Interesting way to frame it. I think it is hard to reopen a question that was never closed.

This is a good point. We re-framed it as "revisit" rather than "reopens".

Reviewer #3 (Remarks to the Author):

In this manuscript, Shalev et al. use a synthetic microbial community-based experiment to test the hypothesis that diversity increases with resource diversity. They first observe that, based on the Earth Microbiome Project (EMP) data, the assumption that increasing species diversity is associated with resource diversity does not appear to hold true. They then design a laboratory experiment to manipulate resource diversity and species diversity, testing the assumptions of consumer-resource theory. Overall, they find the opposite of what is expected – that species diversity decreases with resource diversity. Specifically, they identified bacterial isolates that exemplified this pattern (highly influential). They conclude that these basic theoretical models fail to capture the specific physiology of microbial systems and that there is limited support for these classical ecological principles in microbial systems.

Overall, I was highly impressed with the experimental aspects of this work. The researchers designed an impressive experiment using liquid handling reports to achieve a remarkable number of manipulations. However, I was not convinced by their main conclusions and was surprised that they did not consider the role of resource generalists in their interpretations. Throughout the manuscript, I felt that there was an

incredible lack of information necessary to interpret the results, and instead, important details are buried in the supplemental material. As such, I have many issues with this manuscript in its current form, which I outline below.

We appreciate this feedback and agree that key information should be accessible from the main text. Accordingly, we expanded the main text (including figure captions), and substantially revised and expanded the Methods section to improve clarity and accessibility.

Generalists: I think the simple explanation for the experimental observations in this manuscript is that the highly influential isolates were resource generalists. The basic theoretical predictions used in this manuscript assume that species are specializing on unique resources and can therefore partition niche space as resource diversity increases. But there was no measure of resource niche breadth…

We thank the reviewer for this valuable comment. To clarify the relationship between non-additive competition (i.e., highly influenced strains) and generalism (niche breadth), we performed new analyses using our full 298-strain collection across the 22 resources used throughout the study. Specifically, we examined whether strain generalism correlates with our empirically defined classification of highly influenced versus mildly influenced strains. We find that generalists are somewhat enriched among highly influenced strains; however, this correlation is modest and insufficient to account for the pronounced increase in suppression observed with increasing resource diversity (Supplementary Fig. 22; attached here). We have revised the manuscript accordingly, placing this result in the context of prior work on generalism and competition under resource diversity (e.g., Pacheco et al.).

Main text revisions:

"*Because a previous study (Pacheco et al.*[17]*) linked resource generalism to higher competitiveness under increasing resource diversity, we next examined whether generalism could underlie the enhanced competitiveness of highly influenced strains. Generalist strains were moderately enriched among highly influenced types ($r = 0.36$, $p < 0.001$; $\kappa = 0.36$; Jaccard = 0.43; Supplementary Fig. 22), suggesting that broad niche breadth may facilitate competitive dominance at higher resource numbers. However, this mild correlation was insufficient to explain the pronounced increase in suppression observed under higher resource diversity, indicating that additional mechanisms must drive the stronger competitive responses of highly influenced strains.*"

We also ran new complementary simulations to test how biodiversity patterns change when resource generalism is more or less correlated with being highly influenced by

resource diversity, added in Supplementary Fig. 30 (Attached here). When highly influenced strains are generalists, biodiversity loss is strongest. As this correlation weakens or becomes negative, biodiversity loss becomes less severe, but still occurs across a wider range of conditions (Supplementary Fig. 30; compare across columns). This is thus consistent with the empirical results.

Supplementary Fig. 22:

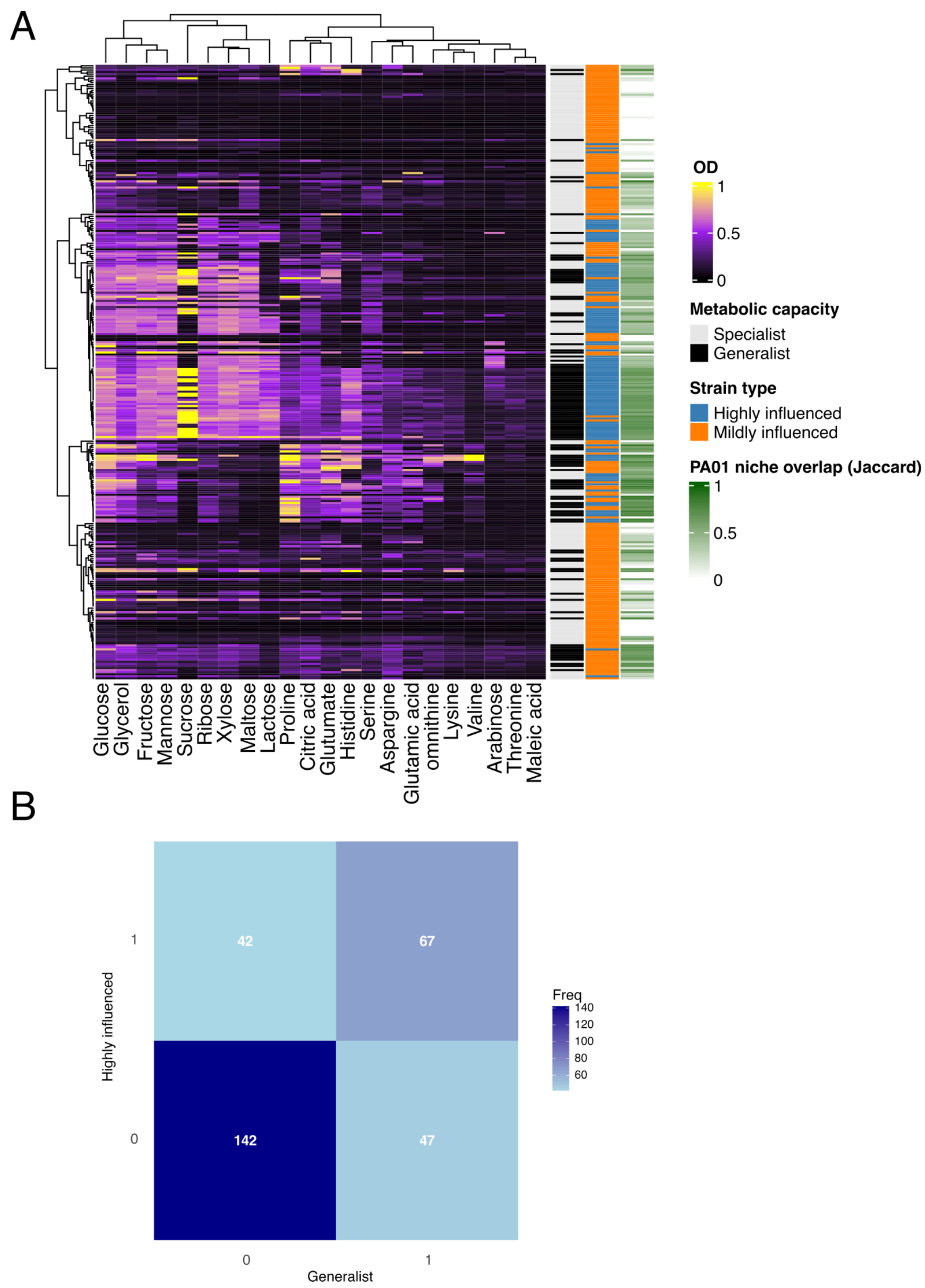

Supplementary Fig. 30:

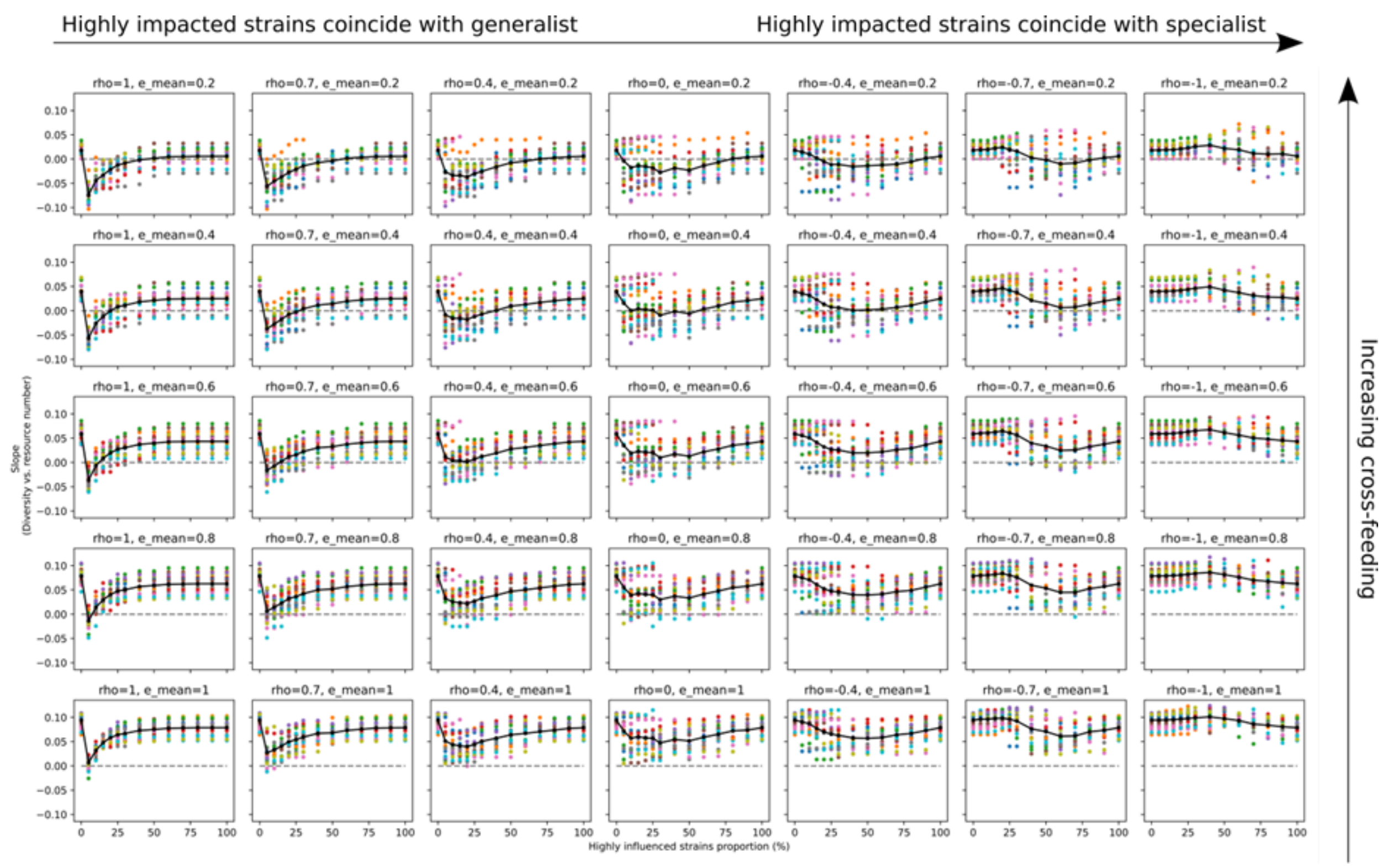

…or discussion of why specific resources were used in the study (no mention of specific resources in the main text at all, nor of the identity of isolates).

We thank the reviewer for this important comment and have expanded the Methods section to clarify the origin and rationale of our strain collection, as well as the rationale for choosing the 22 resources.

Revised text now reads:

"Resources and their Mixtures

*A total of 22 individual resources were used (**Supplementary Table 1**). These resources were selected to encompass a broad spectrum of microbial growth substrates, including sugars, amino acids, organic acids and alcohol-sugars, thereby capturing the main metabolic classes that distinguish "sugar specialists" and "acid specialists" commonly observed among heterotrophic bacteria[35]. Resources were dispensed into 384-well plates containing M9 media without carbon sources using an acoustic liquid handler (Echo 525, Beckman) prior to microbial inoculation. For all resource conditions (individual or mixtures), a final concentration of 0.2% (mass/volume) was maintained. We used concentration (mass/volume) rather than molarity to keep the total available energy comparable across resources of different*

molecular weights, consistent with previous resource-diversity studies[16,17,36]. Mixture conditions ranged from 2 to 16 resources and were assigned randomly.

"Strain collection in sentinel strain experiments
For the sentinel strain experiments, we used 298 bacterial strains isolated from the gut of Caenorhabditis elegans collected from diverse natural environments, including rotting fruits, soil, and compost (Supplementary Data 5). Strains were provided by Dr. Christoph Ratzke and Prof. Hinrich Schulenburg. This collection spans a broad range of bacterial taxa (Supplementary Data 5; Supplementary Fig. 19) and represents a highly diverse and ecologically relevant set of C. elegans–associated microbes suitable for controlled experimental studies."

The 298 bacterial strains used in our study span a broad range of taxa, covering major phylogenetic lineages; Taxonomic analyses now provided in Supplementary Fig. 19 (attached here) and in Supplementary Data 5. While no experimental system can capture the full diversity of natural microbiomes, we believe that this collection represents an exceptional level of phylogenetic and ecological diversity for controlled experiments, thereby providing strong ecological relevance.

Supplementary Fig. 19 (order-level taxonomy, mapped to interactions results):

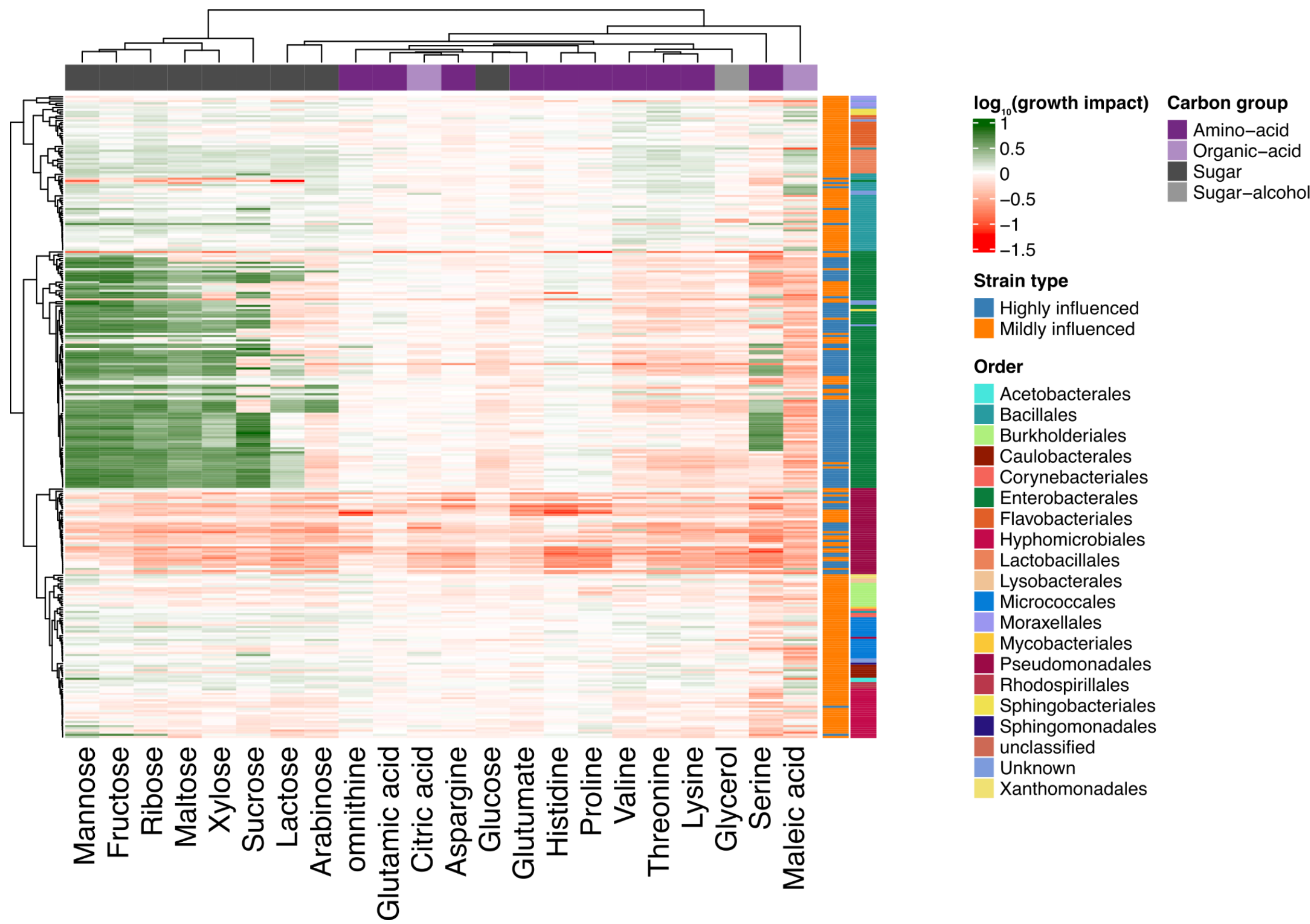

Likewise, the media used in the experiment contained dilute undefined media, which includes an enormous amount of resource diversity and therefore complicates any interpretation.

We agree that the media composition warrants clarification. The Methods section has been revised accordingly and now includes supporting data - Supplementary Fig. 35 (attached here).

Before the main experiments, we optimized the medium composition. Adding a 1:100 dilution of rich medium together with glucose substantially increased the number of strains reaching measurable optical density compared with glucose alone (Supplementary Fig. 35B), whereas the dilution by itself—without an added carbon source—supported only minimal growth (Supplementary Fig. 35A). This indicates that the rich medium alleviates micronutrient limitations without contributing significant carbon.

Revised Methods text:

“The NM supplement was included based on preliminary tests (Supplementary Fig. 35), which showed that this small addition substantially increased the number of strains

reaching measurable optical density, while only a few strains exhibited limited growth on the NM supplement alone in the absence of added carbon sources. This indicates a synergistic effect with defined carbon sources, likely through the alleviation of micronutrient or other abiotic limitations that would otherwise restrict growth."

Supplementary Fig. 35:

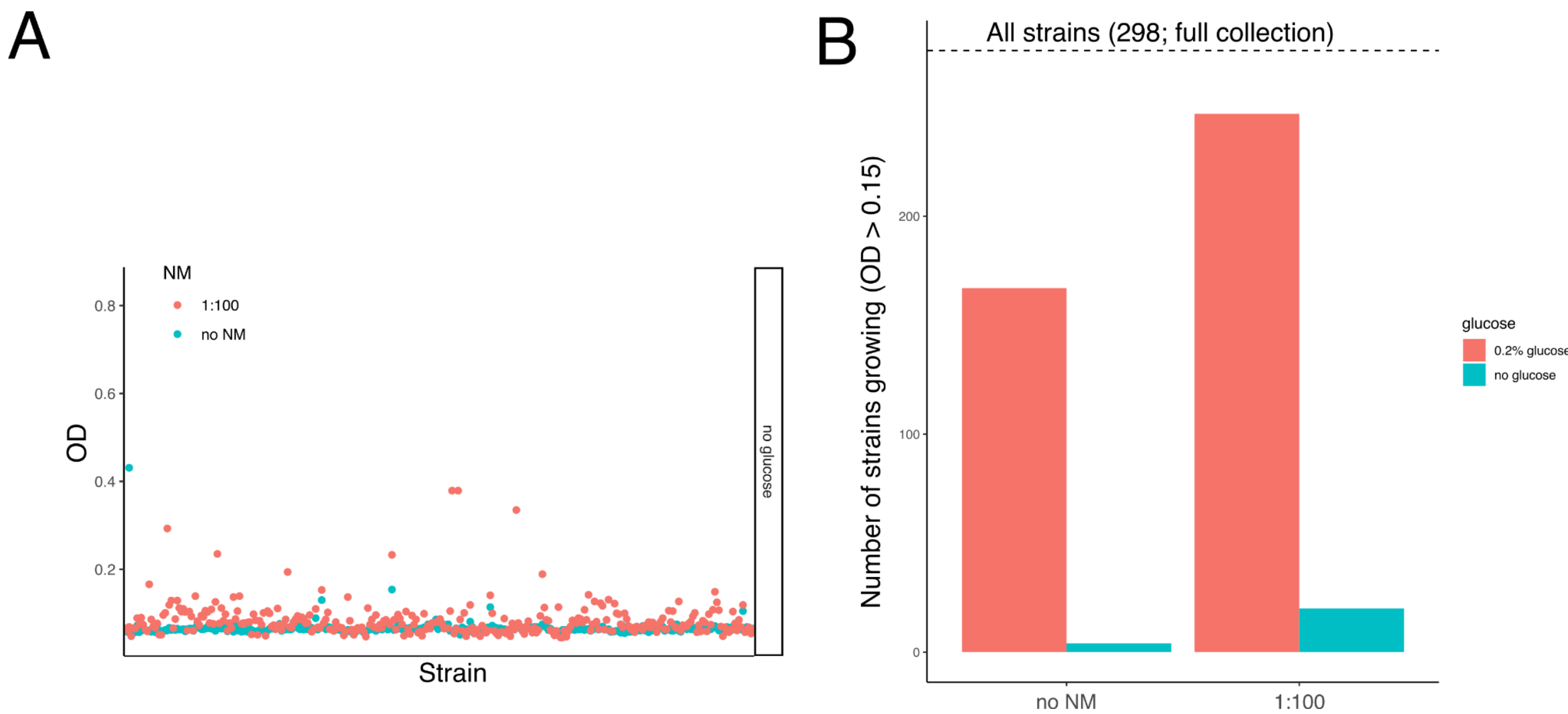

Evidence that the results support the role of generalists appears throughout the manuscript (Fig. 1E, L145, L165, L174, Fig. 2D & E, L235, L298, L315).

We note that part of this general criticism has already been addressed above in the context of highly influenced strains, generalism, and their effects on biodiversity, both empirically and through simulations. Here, we further address this issue specifically in relation to Fig. 1E and Fig. 2D–E. To demonstrate that generalism alone cannot explain the observed reduction in biodiversity with increasing resource number in Fig. 1E and Fig. 2D–E, we provide additional evidence using strain 79 as a case study. Although strain 79 has the strongest impact on biodiversity, we find that it is not exceptionally generalist compared with the other 13 strains tested (Supplementary Fig. 15; attached). This indicates that its pronounced effect on biodiversity cannot be explained by niche breadth alone. We have added this clarification to the main text as follows:

"We tested whether the mechanism proposed by Pacheco et al.[17] could map onto our results. Their work suggested that higher resource generalism leads to stronger competitive advantage and dominance at elevated resource diversity, reducing coexistence through increased niche overlap. Strain 79, despite its strong biodiversity impact, exhibited only moderate niche breadth compared with the other community members, indicating that its dominance cannot be explained by generalism alone (Supplementary Fig. 15)."

Supplementary Fig. 15:

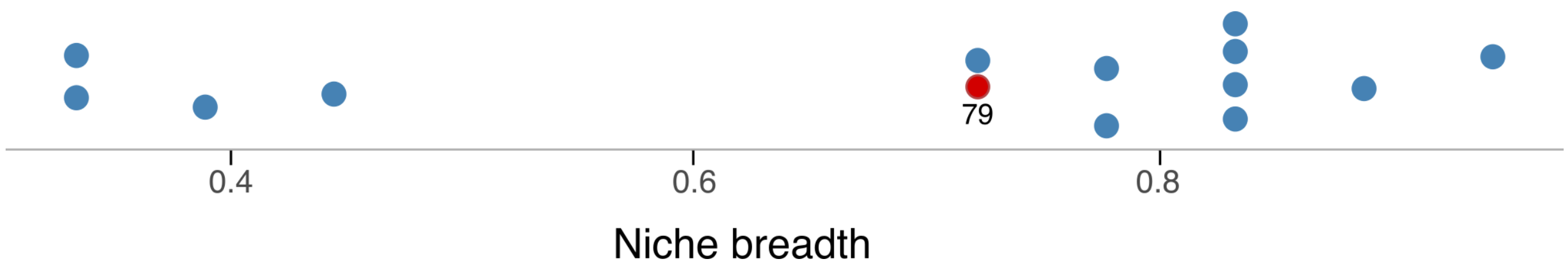

The emergent physiological trait is clearly resource generalism (L317).

The reviewer refers to the synergistic increase in resource uptake observed for highly influenced strains. We agree that this phenomenon can be described as an emergent physiological trait related to resource use. However, our data do not support emergent *resource generalism* in the strict sense—that is, a categorical expansion of niche breadth whereby strains gain the ability to utilize novel carbon sources they previously could not.

Instead, our results indicate a quantitative shift within existing niches: highly influenced strains show increased uptake or growth on resources they could already utilize to some extent (Supplementary Fig. 24). We therefore distinguish between *breadth-based resource generalism*, as commonly defined by the ability to grow across a wider range of substrates, and *emergent resource uptake*, referring to enhanced quantitative use of resources already accessible to a strain. Our findings support the latter interpretation.

To clarify this distinction, we have added data visualizing this conclusion more explicitly in the rebuttal (Rebuttal Fig. 1; attached).

Rebuttal Fig. 1 (caption below):

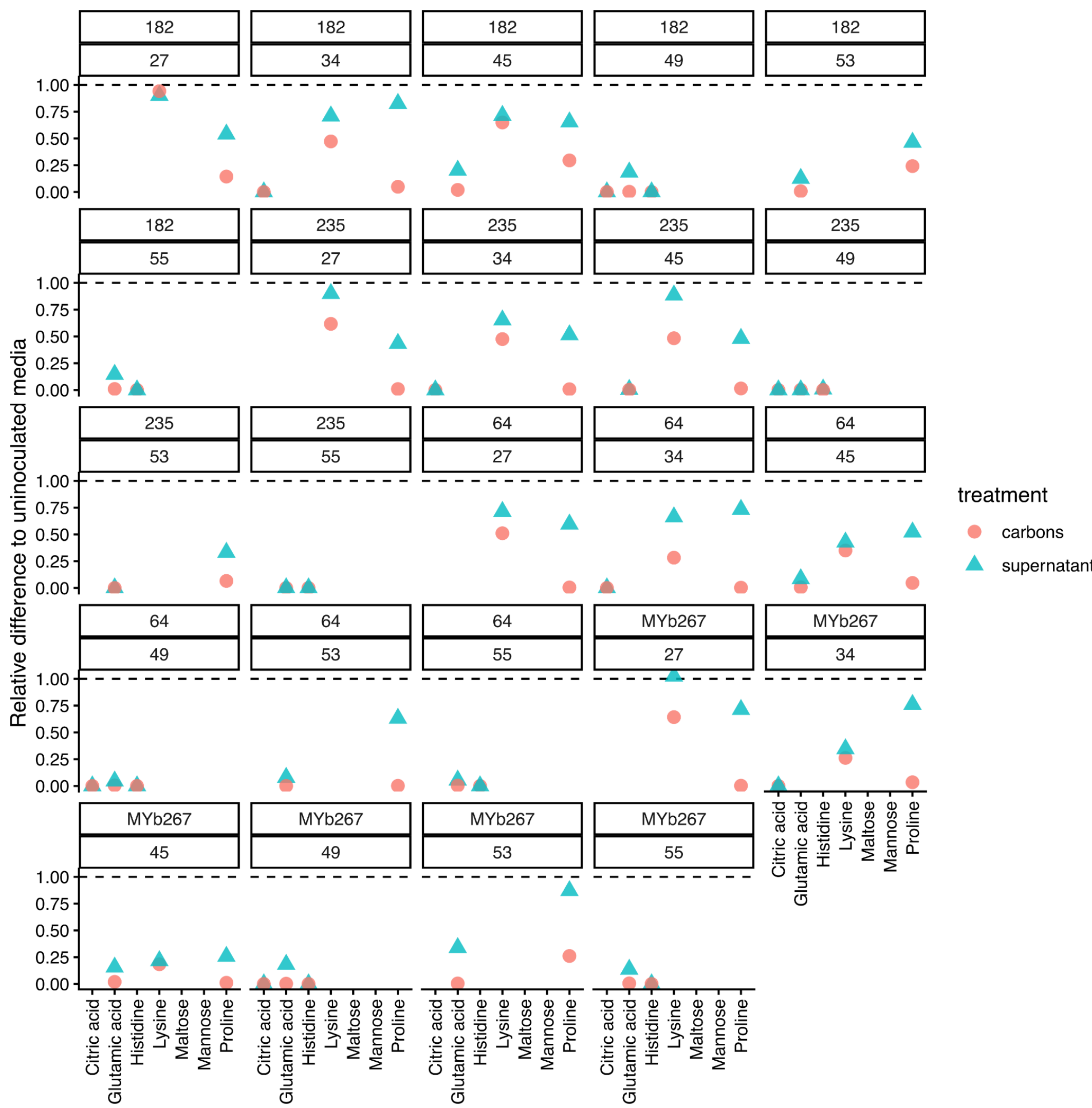

Rebuttal Fig. 1: Strains utilize existing niches more efficiently in mixed-carbon conditions but do not exploit new ones (no emergent resource generalism). Shown are raw GC–MS results, expressed as the relative difference in peak area (AUC) between post-growth samples and the corresponding supplied carbon peaks in uninoculated controls. Each panel shows results for an individual strain (upper facet) across different carbon mixtures (lower facet). Blue points represent supernatant mixtures of individual carbons, and red points represent direct carbon mixtures (design illustrated in Fig. 4A). The dashed line indicates a value of 1, corresponding to no difference between inoculated and uninoculated samples. Except for a single case, lysine uptake by strain MYb267 in mixture 27, mixed-carbon conditions never resulted in the appearance of new uptake peaks relative to single-carbon conditions. This exception is likely an artefact, as the same strain uptakes lysine in both supernatant and carbon mixtures in mixture 34. Overall, our results support improved use of existing niches but no emergent ability to utilize new carbon sources.

Resources and Media: How were the resources in this study chosen? Were there any a priori expectations? Most of these are simple sugars and amino acids.

Please see our response above for the rationale behind selecting the 22 resources (as also revised in the main text).

Regarding *a priori* expectations, we aimed to span major, well-characterized metabolic classes commonly utilized by bacteria, using simple sugars and amino acids that are broadly accessible across taxa. This approach was inspired by prior work (e.g., Pacheco et al. 2021, Nat. Commun.), which similarly used simple, canonical carbon sources to systematically vary resource richness while maintaining comparability across strains. As a general guideline, and informed by classifications of “sugar specialists” and “acid specialists” (Gralka *et al.*, 2023, Nat. Microbiol.), we selected approximately equal numbers of sugar- and acid-based resources to broadly cover this metabolic space.

I would predict that many species are generalists in terms of these resources.

This can indeed be seen in our new Supplementary Fig. 22 (attached in our response above), where the majority of strains display generalist behavior. However, this is not surprising, as many natural microbial communities are dominated by taxa with broad ecological or environmental niche breadths (Dutilh et al., 2023, Nat. Ecol. Evol.), making the prevalence of generalist strains in our collection ecologically realistic and relevant. We do not, however, consider this directly relevant to our conclusions, which focus on emergent interactions driven by emergent resource uptake in mixed-resource environments—processes that categorically occur regardless of generalism patterns, as supported by our revised analyses (as discussed in our responses above).

Further, in the experiments, why were the cells not washed to remove any carryover undefined media?

Thank you for pointing out this point of clarification. We used an acoustic liquid handler to transfer 25 nL of 1:5 diluted pre-culture into 40 µL of assay medium (Methods), corresponding to an effective dilution of approximately 1:8,000. We added this notion in the revised Methods text, which now reads: *“Inoculation was performed using an acoustic liquid handler (Echo 525, Beckman Coulter) by transferring 25 nL of 1:5 diluted culture into 40 µL of media per well (corresponding to a of dilution 1:8,000)”*

At this level, any potential carryover of undefined medium is negligible and unlikely to influence the results. The use of an acoustic dispenser enabled high-throughput culturing in 384-well plates, where individual washing of cells would be technically impractical, time-consuming, and would substantially reduce experimental throughput.

Strains: I was unable to find any information on the strains used in this study.

We apologize for the lack of information regarding the strains used in our study. We have revised the Methods section to clearly describe how the strain collection was established and now include the full taxonomic classification for all strains in Supplementary Data 5. This information is also visualized at the order level—together with the mapping of highly and mildly influenced strains and their interaction outcomes—in the new Supplementary Fig.19 (attached in our response above).

Our revised Methods now reads:

"Strain collection in sentinel strain experiments

For the sentinel strain experiments, we used 298 bacterial strains isolated from the gut of Caenorhabditis elegans collected from diverse natural environments, including rotting fruits, soil, and compost (Supplementary Data 5). Strains were provided by Dr. Christoph Ratzke and Prof. Hinrich Schulenburg. This collection spans a broad range of bacterial taxa (Supplementary Data 5; Supplementary Fig. 19) and represents a highly diverse and ecologically relevant set of C. elegans–associated microbes suitable for controlled experimental studies."

Furthermore, I do not see any evidence to support that the highly influential strains were phylogenetically conserved (not even a statistical support for this).

This is true. We therefore added a new analysis to support this statement. To quantify whether highly influenced strains are phylogenetically conserved, we performed a Mantel test comparing pairwise phylogenetic distances (derived from the core-genome phylogeny) with pairwise similarities in their influence profiles. The analysis used Spearman correlation with 9,999 permutations and tested whether phylogenetically closer strains exhibit more similar influence patterns. The Mantel test yielded a significant positive correlation ($r = 0.145$, $p = 0.0001$), indicating that closely related strains tend to exert similar effects. We have added this analysis to the revised manuscript, along with new taxonomic analyses (as detailed in our previous response): "*Both types were phylogenetically conserved (Fig. 3B, top; Mantel test, r = 0.145, p = 0.0001, 9,999 permutations; for taxonomic information see Supplementary Fig. 19 and Supplementary Data 5*)."

Fig. 3B does not provide sufficient support for these conclusions, as the clustering method used in the heat map is unknown, and the identities of the strains are unclear. "Ordered by core-genome phylogeny" is not that meaningful (L258).

We thank the reviewer for this comment.

The heatmap in Fig. 3B is ordered according to the core-genome phylogeny of the strain collection, constructed in panX using default bacterial parameters with a 95% core-gene threshold. The tree was inferred by maximum-likelihood from concatenated core-gene alignments. We now revised our text to be explicit and clear about how this was done. First, we revised the caption of Fig. 3B to add this information: *"Strains are ordered by core-genome phylogeny inferred with panX (95 % core-gene threshold; see Methods for details).".* Moreover, we have revised our Methods which now includes a new section titled '*Strain phylogeny calculation*', which reads: *"Core-genome phylogeny was constructed using panX[41], which clusters orthologous genes, aligns core gene families, and infers a maximum-likelihood tree from the concatenated alignment. We used default bacterial parameters and defined core genes as those present in ≥ 95 % of strains."*

Resource uptake: In the abstract, the authors claim that they observed higher resource uptake in more complex resource environments. I do not see how this statement is supported by any of the data provided.

We refer to main findings of our study, shown in Fig. 4B and Supplementary Figs. 24 and 25, which are based on the supernatant experiments.

As described in the main text and illustrated in Fig. 4A, we compared two conditions: (i) microbes grown separately on individual resources and their supernatants subsequently mixed, and (ii) microbes grown directly on the mixed carbon sources. Importantly, the proportions of each resource were kept identical between the two conditions (e.g., in a 1:1 mixture of carbon A and B, each was halved compared to the single-resource condition). This design provides a direct test for additive versus non-additive resource uptake. If uptake were additive, we would expect a microbe to consume carbon A and B in the mixture at the same combined rate as when grown on each separately. However, our data clearly show deviations from this additive expectation, indicating that microbes take up more total carbon in more complex resource environments.

Because this result arises directly from a direct comparison of uptake in isolation and in mixtures, and is supported by multiple lines of evidence with strong statistical significance (Fig. 4B and Supplementary Figs. 24 and 25 provide clear "smoking-gun" evidence), we consider it a main finding of our study and refer to it in the abstract.

In fact, I'm overall surprised by the media conditions. For a study such as this, I would have expected resources to be discussed in molarity and that the effect of molarity was accounted for as resource diversity increased (L294).

We thank the reviewer for this helpful comment and agree that this is an important point that should be clarified in the text. We have revised the Methods section accordingly:

"We used concentration (mass/volume) rather than molarity to keep the total available energy comparable across resources of different molecular weights, consistent with previous resource-diversity studies[15,16,34]"

We respectfully note that maintaining all resources at 0.2% (w/v) is not problematic but an intentional normalization strategy. Microbial growth is primarily constrained by the total mass of available carbon, rather than the number of substrate molecules. For example, the same number of sucrose molecules provides roughly twice the energy of glucose molecules due to their higher molecular weight. By normalizing carbon input by mass, we ensured that each condition—single or mixed—contained a comparable potential energy supply.

This approach follows previous resource-diversity experiments that compared environments of differing resource complexity using fixed weight concentrations (*Dal Bello et al.*, *Nat Ecol Evol* 2021; *Pacheco et al.*, *Nat Commun* 2021) and aligns with other high-throughput microbial community studies employing similar normalization (*Kehe et al.*, *Nat Microbiol* 2021).

For the sake of the rebuttal, we also calculated the Gibbs energy for each molecule at 0.2% in 40 ul volume, to ensure that it is indeed similar, using eQuilibrator (https://equilibrator.weizmann.ac.il/). As can be seen, the approximated Gibbs energy for 2 g/L (0.2%) in 40 ul among the resources is very similar:

Compound	Formula	Carbons	Molecular weight (g/mol)	Moles in 40 µL at 2 g/L	Approx ΔG⁰ for 40 µL amount (kJ)
Asparagine	C4H8N2O3	4	132.12	6,06E-04	-1,04E+00
Glutamic acid (Glutamate)	C5H9NO4	5	147.13	5,44E-04	-1,17E+00
Histidine	C6H9N3O2	6	155.15	5,16E-04	-1,33E+00
Lysine	C6H14N2O2	6	146.19	5,47E-04	-1,41E+00
Ornithine	C5H12N2O2	5	132.16	6,05E-04	-1,30E+00
Proline	C5H9NO2	5	115.13	6,95E-04	-1,49E+00
Serine	C3H7NO3	3	105.09	7,61E-04	-9,82E-01
Threonine	C4H9NO3	4	119.12	6,72E-04	-1,16E+00
Valine	C5H11NO2	5	117.15	6,83E-04	-1,47E+00
Arabinose	C5H10O5	5	150.13	5,33E-04	-1,27E+00
Citric acid	C6H8O7	6	192.12	4,16E-04	-1,19E+00
Fructose	C6H12O6	6	180.16	4,44E-04	-1,27E+00
Glucose	C6H12O6	6	180.16	4,44E-04	-1,27E+00
Glycerol	C3H8O3	3	92.09	8,69E-04	-1,25E+00
Lactose	C12H22O11	12	342.3	2,34E-04	-1,34E+00
Maleic acid	C4H4O4	4	116.07	6,89E-04	-1,32E+00
Maltose	C12H22O11	12	342.3	2,34E-04	-1,34E+00
Mannose	C6H12O6	6	180.16	4,44E-04	-1,27E+00
Ribose	C5H10O5	5	150.13	5,33E-04	-1,27E+00
Sucrose	C12H22O11	12	342.3	2,34E-04	-1,34E+00
Xylose	C5H10O5	5	150.13	5,33E-04	-1,27E+00

The observation that strains grew less in mixed-resource supernatants seems obvious, given that the concentration was conserved as additional resources were added.

We thank the reviewer for this comment and would like to clarify why the observation that PAO1 grew less in mixed-resource supernatants is not obvious. The experiment in Fig. 4A–C was designed to test whether growth on mixtures of resources produces a different metabolic outcome than expected from the sum of single-resource effects.

If a bacterium is provided with X mg of carbon source A or B separately, linearity predicts that supplying 0.5X of each together would result in total uptake equal to the linear combination of their individual effects. Instead, we observed greater total carbon consumption in mixtures, indicating synergistic, non-additive metabolism. The comparison between supernatants from mixed-resource cultures and mixtures of single-resource supernatants directly tests this principle: both contain the same total carbon and the same diversity of carbon sources (identical profiles), differing only in whether the resources were encountered simultaneously or separately by the tested bacteria. The CR model predict identical outcomes in this scenario (see figure attached to this rebuttal). Yet, in our results, true mixtures showed stronger metabolite depletion (Fig. 4B) and reduced sentinel growth (Fig. 4C).

This result is therefore unexpected, contradicting the linear metabolic assumption of the established consumer–resource theory and, to our knowledge, has not been demonstrated experimentally or discussed before.

Lack of Literature: Overall, I found that the authors build their arguments on a minimal selection of the literature. For example, they claim that there is little support for resource diversity driving microbial diversity, citing two publications. However, even a brief Google Scholar search yields numerous examples and a diverse body of literature on this topic. Even one of their main references, Dal Bello et al, does a remarkable job demonstrating the relationship. Therefore, I would not claim that there is “scarce” support for this theoretical prediction.

We thank the reviewer for raising this point. Our statement regarding the limited empirical evidence for resource diversity driving microbial diversity referred specifically to controlled experimental studies in which the number of supplied carbon sources was systematically varied. To our knowledge, only two studies have tested this question in a controlled manner; among them, only one—*Pacheco et al.*—used both a defined number of resources and a defined microbial community. *Dal Bello et al.* (*Nat Ecol Evol* 2020) also varied the number of supplied resources but did so using complex, undefined soil microcosms rather than controlled consortia. We now revised our text in Intorduction to make it explicit and clear:

"*Nonetheless, only a few controlled experiments have directly addressed this question[15,16]. Dal Bello et al.[15] found a positive relationship between resource and microbial diversity in microcosms, although the increase in biodiversity was weaker than expected. However, these experiments involved a single, complex, and undefined soil-derived community containing thousands of taxa. When transferred to laboratory conditions, such diverse natural communities often collapse to a few dominant species, as observed in this study, because the majority of natural bacteria cannot survive under lab conditions. This strong environmental filtering may mask the effect of microbial interactions. In contrast, Pacheco et al.[16] examined how increasing resource diversity influences diversity in a defined microbial community—the only study to do so to date. Here, the biodiversity increase was substantially weaker than predicted by classical consumer–resource models and even smaller than that observed by Dal Bello et al., a result that was central to their analysis. Similarly to Dal Bello et al., the use of a single community constrains the generality of their conclusions.*"

This revision better emphasizes the scarcity of systematic tests in defined synthetic consortia—the gap our study aims to address. We acknowledge that despite our extensive search, additional relevant works may exist, and if the reviewer can point us to specific studies fitting these criteria, we would be very happy to include and discuss them in our revision.

Regarding the more general concern about a lack of supporting literature, we have expanded the reference list from 42 to 51 citations, adding additional references throughout the manuscript to better support our claims.

Complex Environments: When interpreting complex ecological systems, it is perhaps naive to consider resources as the only niche axes that species are dividing, especially in soils, guts, and other complex environments that can maintain diversity due to large variability in environmental conditions.

We thank the reviewer for pointing out this important tension. We present the EMP analysis primarily as context and motivation for our controlled experiments, as ecological datasets are best suited for hypothesis generation. Because natural microbial ecosystems are inherently uncontrolled and confounded by many unmeasured factors, we performed controlled experiments that allow direct testing of causality under defined conditions. Nonetheless, as this point may not have been sufficiently clear, we have revised the text to clarify it and further softened the language to emphasize that the EMP results are motivational rather than conclusive.

Revised text is now:

"We found that resource diversity does not consistently correlate with biodiversity (Fig. 1C; Supplementary Fig. 1C-D): in some ecosystems biodiversity increases with higher resource diversity, while in others, it decreases. But, interpreting these data is difficult, as it is not clear whether the detected nutrients were supplied by the environment or synthesized by the bacteria, complicating efforts to link species composition to nutrient richness. More broadly, natural systems are inherently uncontrolled and confounded by multiple unmeasured factors, which makes data interpretation challenging and asks for controlled lab experiments."

And also:

"Motivated by the difficulty of drawing causal conclusions from the EMP patterns and by the limitations of previous laboratory studies, we systematically tested multiple defined microbial communities…"

Figure 1: The legend of Figure 1 references "Animal Distal Gut," but the figure actually shows "Water".

We thank the reviewer for catching this inconsistency and apologize for the confusion caused by our phrasing. Our intent was to refer to the 'Animal Distal Gut (non-saline)' environment, which contains the largest number of samples in the EMP dataset (Supplementary Fig. 1D). However, in the text, this reference could be misinterpreted as referring to the main figure panel showing the 'Water' environment, which illustrates the most extreme slope rather than the largest dataset (refering to the main Figure 1C). We have now rephrased in the figure legend to make this distinction clear: "*Notably, the Animal Distal Gut (non-saline) environment contains the largest number of samples in the EMP dataset and shows a significant negative correlation (Supplementary Fig. 1D)."*

The central premise of the paper is that the EMP data provide more support for a negative relationship.

Importantly, we do not argue that the EMP data provide stronger evidence for negative relationships, but rather highlight the absence of a widespread positive trend, as would be expected from classical consumer–resource theory (Fig. 1A–B vs. 1C):

"We found that resource diversity does not consistently correlate with biodiversity (Fig. 1C; Supplementary Fig. 1C–D): in some ecosystems biodiversity increases with higher resource diversity, while in others, it decreases."

In fact, this claim is based on the evidence presented in Figure 1. However, the number of points in the negative relationship, "Water", is far fewer than the points for "Sediment".

Based on this figure alone, I would conclude that there is more support for the positive relationship. Perhaps I'm missing something, though.

We thank the reviewer for this comment and are happy to clarify this point. The 'Water' and 'Sediment' examples in Figure 1 were included only to illustrate the two extremes of the diversity–resource number slopes and to demonstrate how these slopes were derived for the overall analysis shown in the main Figure 1C. These examples were chosen for clarity, demonstrating how the slope was derived, rather than to represent an overall trend.

In the full dataset (main Fig. 1C; Supplementary Figs. 1C–D), six environments exhibit positive slopes (two of which are close to zero) and six show negative slopes. When statistical significance is considered, only four out of twelve environments display a significant positive relationship.

Thus, the EMP data reveal a surprisingly small number of positive relationships—contrary to the null expectation, based on both ecological consensus and classical consumer–resource theory, that higher resource diversity should promote higher microbial diversity. This observation motivated our work, but given the inherent inconclusiveness of large-scale ecological datasets, we treated the EMP results as contextual and suggestive rather than mechanistic, motivating our controlled experimental analysis.

Methods: Overall, I found that there were plenty of details for the bioinformatics section, but the methods were lacking in other sections. For example, the generalized Lotka-Volterra section was not informative at all and merely referred to the supplemental material. More information is needed because this was such a critical part of this manuscript.

We thank the reviewer for pointing out this important issue.

We have now extensively expanded the Methods section and Supplementary Texts in this revised manuscript to improve clarity and completeness. In particular, we have substantially extended the modeling subsection to include detailed descriptions of the model formulation, parameterization, and implementation.

For example, the discussed gLV section now reads:

"Data from single-species growth (Supplementary Fig. 9), pairwise interactions (Fig. 2A) and community compositions (Fig. 1D, E and Supplementery Fig. 3) at steady state were used to infer Lotka-Volterra interaction matrices of the 14 strains comprising these communities. Interaction matrices were constructed for three datasets corresponding to

1, 8, and 16 available carbon sources. Since each of these three datasets contains data for multiple different nutrient compositions, we treated interaction strength as a random variable by using Bayesian regression to infer their values.

*The steady state of the generalized Lotka–Volterra system is obtained by setting the equations in Eq. 1 (main text) to zero, taking into account only those species whose population densities remain non-zero.The obtained equations where fit to the data with Bayesian regression (Monte Carlo Markov Chain sampling, NUTS solver in pyro, python). As priors we used Gaussian distributions with a mean of 1 for the off-diagonal entries and a mean equal to the inverse carrying capacity for the diagonal entries. This is based on the fact that the self-interaction coefficient (α_self) corresponds to the inverse of the carrying capacity (K), a standard notatio[CR1] n generalized Lotka-Volterra models[31,52]. Posterior sampling was performed with 30 chains, 400 warm up steps and 200 sampling steps. For full details, see **Supplementary Text 2**. Note that error bars for α_{self} in Figure 2B (middle) may be overestimated as the interaction values are not independent (see Supplementary Fig. 6A).”*

And the expanded methods of the CR model now reads:

“For the classic Consumer-Resource model, we consider a community of 20 microbial species competing for 30 resources in a continuous dilution (chemostat-like) environment. Species take up available resources and either turn them into biomass or transform them into secreted metabolic byproducts (cross-feeding), and die through dilution. Resources are depleted through species uptake, replenished through secretion from species, and are subject to dilution - which replenishes supplied resources and washes out others. For each of 20 independently parameterized communities, we varied resource complexity across 1, 2, 4, 8, 16 supplied resources (equal total supply split evenly among the chosen resources). Initial abundances were uniform (N_0 = 1/20 per species). Simulations used `scipy.integrate.solve_ivp` v1.16.3 in Python v3.14.0 as ODE solver (rtol=1e-3, atol=1e-6, method=”RK45”), over t = 0–800 (a.u.) with dilution = 0.1. Cross-feeding is modelled as a metabolic transformation tensor ($b_{i,\beta\rightarrow\alpha}$) that allows a fraction of consumed resource β to be secreted as resource α by species i, thereby generating new metabolites available to other species. This formulation follows standard extensions of MacArthur-type models incorporating metabolic by-product exchange and saturating resource uptake kinetics[8,16,51]. For full details, see Supplementary Text 1 Section 1 and the full code (under ‘CR_model_simulations’).
For the highly influenced strains modified model version see Supplementary Text 1 Section 2. In this updated model, we introduce a species-specific sensitivity parameter γ. When γ>0, the species increases its resource uptake affinity (i.e., reduces K⍰) as resource diversity increases. We set γ=1 for highly influenced strains (HIS) and γ=0 for

mildly influenced strains (MIS) by default. For each of the 20 independently parameterized communities, we vary the highly influenced strain (HIS) fraction f = 0, 0.05, …, 1.0 (14 fractions)(Fig. 4E; Supplementary Figs. 28-34). To assign the species as either HIS or MIS, we first assigned a tendency to be highly influence for each species i, which is correlated with resource generalism. We then rank the species by their tendency to be highly influenced, and assign the ones with highest values as HIS according to the preassigned fraction f, and the rest as MIS. We simulated the 20 communities under 14 HIS fractions, across the same five resource complexities (1–16) with equal total supply, t = 0–800, dilution = 0.1, and uniform initial abundances.
We further performed robustness test by systematically varying key parameters and assumptions. These include (i) cross-feeding rate; (ii) correlation between highly influenced behavior and resource generalism; (iii) γ value for HISs; (iv) a continuous instead of binary distribution of MIS and HIS behavior; (v) an alternative mechanism where resource complexity influences HISs by increasing assimilation efficiency instead of resource affinity; and (vi) the number of initial species for each community. For full details see Supplementary Text 1 Section 3, Supplementary Figures 30-34, and the full code (https://github.com/orshalevsk/Emergent_Biodiv_loss/).”

Minor:
1. The section titled “Resource diversity fuels competition, which reduced coexistence” is a discussion, not results.

We thank the reviewer for this comment. This section summarizes the experimental and modeling results in a concise, integrative manner. Specifically, Figure 2B–D shows that increasing resource diversity leads to stronger competitive interactions, and Figure 2C, E–F demonstrate that this heightened competition reduces coexistence. We therefore maintain this section within the Results, as it directly presents empirical findings rather than post hoc interpretation.

2. Is there a difference between resource diversity and resource complexity (L219)

We thank the reviewer for this comment. In our study, resource diversity and resource complexity are used synonymously. We have added a brief clarification in the text to make this explicit: “*hereafter, resource ‘diversity’ is used interchangeably with resource ‘number’ in this manuscript”*

3. Fig 3B: I cannot see gray in this figure. Perhaps it is my color vision.

We thank the reviewer for this comment and apologize if the grey elements in Fig. 3B are difficult to distinguish. The figure indeed contains a large amount of data and color

information. We conducted a small informal test among lab members, who were generally able to identify the grey elements successfully. Given the richness of the data, we were unable to find a clearer way to present all elements without losing important information. Nonetheless, we are happy to follow any specific suggestions from the editorial board regarding alternative visualizations.

4. Why were the sequences not aligned to a reference database (L503)?

If the reviewer is referring to line 503 (chimera removal), we used the same custom database employed for mapping with vsearch --uchime_ref, as it accurately represents the biological sequences present in our system. Using a broad public database (e.g., SILVA or Greengenes) would not improve chimera detection and could instead introduce false positives from unrelated taxa. We have slightly revised the text to clarify that the same custom database was used for chimera removal: "*Chimeric reads were removed using vsearch (v2.21.1)45 with --uchime_ref against this custom reference database.*"

5. What is "Predicted microbially derived metabolite richness"? I do not see any mention of this in the Shaffer et al manuscript. They do not use any tool to predict microbially derived metabolites; instead, they use observed metabolites.

The concept of "predicted microbially derived metabolite richness" is described in the Shaffer et al. manuscript. It is expalined in the Methods section under "Metabolite intensities reveal habitat-specific distributions", and also discussed throughout the main text. For example, the authors state: *"We present an analysis of microbially related metabolites and microbe–metabolite co-occurrences across Earth's environments (Extended Data Fig. 1)."* and also *"We then refined that dataset to include only putative, microbially related metabolites (that is, defined as being produced, modified by, or otherwise associated with a microbe), resulting in 6,588 metabolites across all samples (12.55% of all metabolites)."*

Moreover, Shaffer et al. describe how they identified these metabolites: *"To establish putative microbially related secondary metabolites, we collected annotations from spectral library matching and the DEREPLICATOR+ tools and queried them against the largest microbial metabolite reference databases (Natural Products Atlas and MIBiG). Molecular networking was then used to propagate the annotation of microbially related secondary metabolites throughout all molecular families (that is, the network component)."*

Reviewer #3 (Remarks on code availability):

I have done a minor review of the code. From my assessment, the simulation code and the code for some analyses are present. However, it does not appear that this includes the code for all analyses in the manuscript.

Reviewer expertise:

Reviewers' comments:

Reviewer #1 (Remarks to the Author):

The authors have for the most part done a great job addressing my concerns. The additions to the text have vastly improved it, and the tempering of some claims make the findings more convincing. The additional simulations, such as including additional physiological mechanisms, are very helpful and enrich the paper.
I nevertheless have a few remaining concerns and suggestions:

Title and abstract: I understand the desire to make a provocative claim about biodiversity, but from the rebuttal and the data, my interpretation is that there is generally no positive relationship between biodiversity and resource diversity, rather than that there is generally a negative relationship. Therefore, “Surprisingly, we observe the opposite: microbial biodiversity often declines as resource diversity increases.” in abstract in my eyes does not capture the spirit of the paper and should be replaced by something more nuanced. For example, I found this statement from the introduction much more fitting: “We found that resource diversity does not consistently correlate with biodiversity (Fig. 1C; Supplementary Fig. 1C-D): in some ecosystems biodiversity increases with higher resource diversity, while in others, it decreases.”

We thank the reviewer for this thoughtful comment and agree that the original phrasing in the abstract may have overstated the generality of a negative relationship between resource diversity and biodiversity. We have therefore revised the abstract to more accurately reflect the context-dependent nature of our findings. The sentence now reads: “Contrary to this expectation, we find that the relationship between resource diversity and microbial biodiversity is not consistently positive and can even decline with increasing resource diversity.” In addition, we have toned down the opening and closing statements of the abstract and revised the title to better reflect the mechanistic focus of the study, which is now: “Enhanced resource uptake drives microbial biodiversity loss as resource diversity increases”.

Statistics: there are many instances of statistical tests performed across a large number of experimental or simulated conditions, but I can't find in the methods whether and if so, how, multiple testing correction was performed. (e.g., SI Fig 3, but there are many examples)

We thank the reviewer for raising this important point. We carefully re-examined all main and supplementary figures to ensure consistent treatment of multiple hypothesis testing.

We identified three cases in which multiple statistical tests were performed across panels without formal correction: the multi-panel correlation plots in Supplementary Figures 1, 3, and 23. In addition, Figure 3E contains three parallel correlation analyses. Although the number of comparisons there is small, for full consistency we have now applied multiple testing correction in this figure as well. Specifically, we applied the Benjamini–Hochberg false discovery rate (FDR) procedure within each multi-panel figure, treating the tests shown in each figure as a single family. We retain the raw p-values in each panel (as they correspond to the reported correlation coefficients), but statistical significance (asterisks) now reflects FDR-adjusted p-values. This is explicitly indicated in the updated figure captions.

Importantly, our conclusions do not rely on counting the number of individually significant environments, but rather on the overall trends and directionality of the correlations across conditions. Applying FDR correction does not alter the conclusions of the manuscript. Notably, even when restricting the analysis to relationships that remain statistically significant after FDR correction, the qualitative conclusions remain unchanged.

For Supplementary Figure 25, Benjamini–Hochberg correction had already been applied to the reported p-values; however, this was not explicitly stated in the original caption. We have now clarified this in the revised manuscript to ensure transparency.

For the simulation analyses, some parameters and community compositions are randomly drawn, resulting in stochastic variability across replicate simulations. However, we do not perform formal hypothesis testing on simulation outputs and therefore do not report p-values for these analyses. Instead, simulation results are summarized descriptively (e.g., mean ± SEM across replicate runs) and trends are evaluated across parameter sweeps. Because no inferential statistical tests are applied to the simulation results, multiple testing correction is not relevant for these analyses.

In the rebuttal, the authors write: "While strain 79 shows the most pronounced competitive response in pairwise experiments, several other strains display similar—though weaker—trends. As shown in Supplementary Fig. 13, strains 95, 81, and 27 also exhibit negative slopes in pairwise settings, with strain 95 in particular showing relatively large absolute impact factors (Supplementary Fig. 11). Thus, 4 out of the 14 tested strains show a consistent negative relationship between resource number and biodiversity in pairwise experiments, indicating that the effect is not exclusive to strain 79." - Without proper statistics, I find this difficult to judge. Strains 79 and 81 are the only strains that show a consistent trend with the number of resources, 95 and 27 show a non-monotonic relationship. Are these slopes significant? Given that there are 14 strains, this makes me wonder whether such behaviors are not expected by chance; 7 of the 14 strains show monotonically positive slopes (though it's not clear whether those slopes are significant).

We thank the reviewer for this careful assessment. We clarify that, in the pairwise analysis, the slope for each strain was referred to as the change in biodiversity between the single-resource condition and the multi-resource conditions (8 and 16 resources). Importantly, the experiment includes only three discrete resource levels (1, 8, and 16), rather than a continuous gradient from 1 to 16 resources. Accordingly, we do not test for strict monotonic trends across many intermediate levels, but instead assess the directional shift from single- to multi-resource environments. Because only a subset of possible multi-resource combinations was experimentally sampled, responses at 8 and 16 resources may not always appear strictly monotonic.

Regarding statistical significance, we confirm that the negative slope is statistically significant for strain 79. For strains 95 and 27, while the overall change relative to the single-resource condition is negative, the slope does not reach statistical significance, largely due to non-monotonic intermediate values.

Importantly, our conclusions do not rely on the statistical significance of the effect of any single strain in a pairwise interaction experiment. Rather, the central result concerns the aggregate pattern involving independent experiments and simulations: the change of average impact factor of strains with increasing resource number in pairwise settings predicts their influence on biodiversity trends in community contexts (see Supplementary Fig. 12), and this relationship is further supported and generalized by our simulation results.

The pairwise and community experiments were conducted independently and involve different resource mixtures, yet the predictive relationship between pairwise impact and community-level biodiversity response remains evident (Supplementary Fig. 12). While Fig. 2E–F highlights strain 79 as a representative example with a particularly strong effect, the broader trend is not limited to this strain.

Due to experimental constraints, it was not feasible to exhaustively measure all pairwise interactions across all possible multi-resource environments. The design therefore includes three discrete resource levels (1, 8, and 16) and a limited subset of resource combinations. In addition, the number of experimentally assembled communities was necessarily limited, and many included strain 79. To address these limitations and assess generality, we complemented the experiments with Lotka–Volterra simulations.

These simulations allowed us to explore a broader range of community compositions and resource contexts and demonstrate that the mechanistic relationship between competitive impact and biodiversity response extends beyond specific experimental realizations. Thus, although individual strain impact factor in pairwise interactions may vary and occasionally appear non-monotonic, the overall mechanistic pattern linking competitive impact and biodiversity response is robust and generalizable.

Perhaps the authors could highlight more strongly that strain 79 is an example of a highly competitive strain at high resource diversity and discuss its relationship to the strains in the sentinel experiments? E.g., is 79 closely related to the facilitative or the suppressive HIS?

We have clarified this point in the revised manuscript. Strain 79 is classified as Pseudomonas based on 16S rRNA sequence analysis, and this is now stated explicitly. Members of the genus Pseudomonas are well represented among suppressive highly influenced strains (Supplementary Fig. 19), consistent with the strong competitive behavior observed for strain 79.

The phylogenetic conservation analysis shown in Fig. 3B is based on a genome-derived phylogeny for the 298 strains included in the sentinel assay. As strain 79 is taxonomically assigned based on 16S rather than a full genome sequence, we refer to its genus-level classification for contextual comparison with suppressive highly influenced strains.

In the rebuttal, the authors write: “In our corrected results, we now observe that as the fraction of Highly Impacted Strains (HIS) increases, the slope drops sharply from positive to negative, then gradually recovers back to positive/neutral.” – This seems to make a clear prediction, namely that the fraction of HIS determines whether the slope is positive or negative. Can the authors reinterpret their results from Fig 1 in light of this finding?

We agree that the resource–consumer model generates a clear qualitative prediction. Specifically, it predicts a non-monotonic relationship between the fraction of highly influenced strains (HIS) and the biodiversity–resource slope, with the strongest negative slopes occurring at intermediate HIS frequencies and recovery toward neutral or positive slopes at higher fractions.

However, our current experimental dataset does not allow a direct quantitative reinterpretation of Fig. 1 in terms of HIS fraction. The experimental system includes 14 strains and a limited number of assembled communities, many of

which contain strain 79, and therefore does not span a sufficiently broad or balanced range of HIS fractions to evaluate whether such non-monotonic behavior is observable experimentally.

In addition, the HIS classification was derived from the sentinel assay, and we do not have independent HIS-type assignments for all strains within the specific communities shown in Fig. 1.

Importantly, the resource–consumer model is mechanistic and parameterized using experimentally derived impact factors, allowing us to explore a broader range of community compositions than is experimentally feasible. While the qualitative pattern is robust across parameter regimes, the precise location of the minimum slope (i.e., the fraction at which biodiversity reduction is strongest) depends on model parameterization and should not be interpreted as a fixed quantitative threshold.

We therefore view the model as providing a mechanistic explanation and a hypothesis for how competitive composition can reshape biodiversity–resource relationships, rather than as a framework for a direct quantitative reinterpretation of Fig. 1 with the current data. A rigorous test of this prediction would require a larger, systematically designed experiment in which communities are assembled with controlled and validated proportions of highly and mildly influenced strains. Such targeted experiments would allow explicit testing of the predicted non-monotonic dependence on competitive composition and represent a direction for future work.

L193ff: I found the new description of the impact factor confusing. "which equals the growth of a strain in the presence of an interaction partner divided by the growth without it. This metric quantifies how the population of one species affects that of another and thus characterizes the influence of a given species." Is the impact factor defined for the species being impacted or for the species having the impact? I believe it is the latter, but it is not clear.

We thank the reviewer for pointing out this ambiguity. The impact factor is defined for the strain exerting the effect (i.e., the species having the impact), and we have clarified this explicitly in the revised text: "*This metric quantifies how the population of one species affects that of another and thus characterizes the influence of a given species,* ***and is therefore defined for the strain exerting the effect.***"

Are the impact factors reported in Fig 2D the average of the impact factors measured for one strain across all the pairs it is involved in?

Yes, the impact factors shown in Fig. 2D represent the average impact factor of each strain across all pairwise interaction partners for each resource condition. We have clarified this explicitly in the figure caption: "*Strain '79' showed the steepest increase in suppressiveness among experimentally tested strains. For each strain,* ***impact factors were averaged across all pairwise interaction partners for each resource condition****, and we fitted log_{10}(impact factor)...*"

Does an IF<1 mean that when that strain is present, other strains tend to have relatively low relative abundance in pairs? Please explain more clearly, perhaps with an example.

The impact factor is calculated based on the absolute abundance of the affected strain in co-culture relative to its abundance in monoculture, not its relative abundance within the community. Absolute abundance was estimated using OD_{600} measurements together with 16S rRNA sequencing data. As illustrated in Fig. 2A and stated in the text ("a drop from 6 to 2 yields an impact factor of 0.33"), IF < 1 indicates suppression (reduced absolute abundance in co-culture), whereas IF > 1 indicates facilitation. Also mentioned in main text: *"An impact factor of 1 indicates neutrality, values below 1 indicate suppression, and values above 1 indicate facilitation."*

We have now clarified in the figure caption of Fig. 2A that the impact factor is calculated using absolute abundance (not relative abundance) of the affected strain in co-culture versus monoculture: "*To assess strain-level contributions to diversity loss, we defined an impact factor based on the change in a strain's partner's* ***absolute abundance (estimated from OD_{600} and 16S data)*** *in co-culture versus monoculture. As exemplified, a drop from 6 to 2 yields an impact factor of 0.33."*

L314ff: “These findings suggest that highly influenced strains not only become more competitive in the lab, but also tend to dominate natural ecosystems as resource complexity increases—reinforcing the ecological relevance of our experimental results.” – I feel this is an overstatement. HIS are very low abundance indeed. They do become more abundant, yes, but they are not dominant in the environment, unlike in their experiments. I suggest tempering this claim to something akin to “but they tend to become more abundant in natural ecosystems”

We agree and have tempered the statement accordingly. The sentence now reads: “*These findings suggest that highly influenced strains not only become more competitive in the lab, but also tend to* ***become more abundant*** *in natural ecosystems as resource complexity increases—**suggesting** the ecological relevance of our experimental results.”*

Related to this, in Supplementary Fig 23: “Despite finer resolution, the overall pattern recapitulates Fig. 3E: the relative abundance of highly influenced strains increases with resource complexity, whereas mildly influenced and unmapped reads do not.” I don’t see this in the data: of 8 environments, 3 show a significant (though multiple testing correction may change this number) increase of MIS, 3 show a significant increase of HIS; 2 show a significant decrease of MIS, 1 shows a significant decrease in HIS. Therefore, I would not say that this the trend of HIS increasing with resource complexity “is generally consistent across environments” (l314 in main text).

We thank the reviewer for this careful assessment. In the EMPO3 breakdown, 7 of 8 environments show positive directional trends for HIS, whereas 1 shows a negative trend. For MIS, 5 of 8 show positive trends and 3 show negative trends. When restricting the analysis to statistically significant relationships after multiple-testing correction, the numbers decrease, but the directional tendency remains similar (HIS: 3 positive, 1 negative; MIS: 2 positive, 1 negative). Our intention in stating that the trend is “generally consistent” was to refer to this directional bias rather than uniform statistical significance across all environments. However, to avoid overstatement, we have revised the wording in the manuscript to more accurately reflect the variability across environments: *“A similar directional tendency is observed in most environments (Supplementary Fig. 23), albeit with variability in strength and significance.”*

I do wonder how the data in A and B are related. There are three EMPO3 categories for animals (top row), where most points seem to be in the distal gut category, which shows a significant DEcrease of HIS. How then is the overall relationship positive in the EMPO2 animal category?

EMPO2 and EMPO3 are predefined hierarchical categories from the Earth Microbiome Project classification, which we used as provided. The EMPO2 analysis is based on a pooled regression across all samples within the EMPO2 category and is not derived from averaging the EMPO3 slopes. Therefore, it is possible for a specific EMPO3 subgroup (such as distal gut) to show a negative association while the overall EMPO2 animal category shows a positive trend when all samples are analyzed together. This occurs because EMPO3 subcategories differ in sample size and covariate structure, and pooling across all animal-associated samples in EMPO2—where the dataset contains many observations—can yield a different overall slope than any single subgroup.

Minor comments:
L106: “robust to contamination” this is probably unclear without consulting the supplementary figure, perhaps “remained when controlling for potential contamination”?
Thanks, this is much better. Changed accordingly.

L155: typo, interactionS
Thanks. Fixed.
L157: the equation is not displayed correctly
Thanks for catching this, we corrected the formula.

L601: incomplete sentence
We found this as the corresponding sentence in the manuscript we sent:
Taxonomic classification of bacterial strains

Taxonomic classification of all bacterial strains was performed based on their assembled genomes using a similarity-based approach against the AnnoTree 602 reference database (v2021).

Reviewer #1 (Remarks on code availability):

The code looks complete now, though I have not attempted to run it. There is a readme file for each type of analysis/simulation.

Reviewer #2 (Remarks to the Author):

Even if I consider the metabolomics work linking physiology to ecology quite impressive and rare in the field, I am unfortunately not fully convinced with the more ecological part. Many claims seem overstated to me, and the level of evidence (effect sizes etc) still does often not fully match the ambition of the claims.

1. L302 header: "Increased microbial competitiveness with resource diversity is widespread". Despite the impressive size of the experiment, I still don't buy that this trait of being "highly influenced" will be conserved if the same experiment was tried with other phylogenetically different sentinel strains. As evidence for this, we can see at Fig S13, depicting the impact factor of each strain against the other 13, where variation is massive (SEM is plotted as errorbar, and standard deviation, which would be appropriate to describe the data, will be sqrt(13)=3.6 times larger in all cases.

We first note that we revised the section title to be more cautious about generalization: *"Increased microbial competitiveness with resource diversity is widespread across our strain collection."*

We agree that interaction strengths vary substantially across strain pairs, as shown in Supplementary Fig. 13, and therefore we cannot rule out that the quantitative classification of strains could depend to some extent on the choice of sentinel strain. However, several observations suggest that the phenomenon we report is not specific to a particular sentinel strain.

First, despite the high variability in pairwise interactions, consistent trends emerge when interactions are aggregated across partners. In particular, strains 79, 95, 27, and 81 show a negative trend in their impact factor with increasing carbon source diversity (Supplementary Fig. 12). Interestingly, these strains also fall into phylogenetic groups enriched for highly influenced strains: strain 79 belongs to *Pseudomonales*, a lineage enriched for highly influenced strains, and strains 27 and 95 belong to *Enterobacteriales*, the most enriched order (see supplementary Fig. 19). Strain 81 belongs to *Sphingomonadales*, which is represented by only a single strain in our 298-strain collection and does not itself show the highly influenced phenotype. Thus, although individual pairwise interactions vary substantially, the aggregated signals are broadly consistent with the phylogenetic structure observed across the larger strain collection in the sentinel strain experiment.

Second, our metabolite–supernatant experiments provide an independent line of evidence that does not rely on the sentinel strain assay used in Fig. 2. In these experiments, metabolic environments generated by highly and mildly influenced strains were characterized using GC–MS under independently randomized resource mixtures, and these metabolic profiles produced distinct physiological responses consistent with the HIS/MIS classification.

Finally, the sentinel assay enabled us to probe a much broader strain collection (298 strains, >30,000 measurements), allowing us to detect large-scale trends in how competitiveness changes with resource diversity. While using a single sentinel strain represents a trade-off between throughput and generality, the observed patterns are supported by multiple independent datasets, including the pairwise experiments, metabolomic analyses, and environmental metagenomic data from the Earth Microbiome Project.

We therefore interpret the "highly influenced strain" phenomenon as a broadly observed trend rather than a property tied to a specific sentinel strain, while acknowledging that the precise quantitative classification may depend on the interaction context.

2. The added paragraph L256-262 represents only anecdotal evidence (just one strain), does not disprove Pacheco et al. Indeed, when authors do it later on for a larger number of highly influenced strains, they find that generalism is enriched, but they diminish it (only moderately enriched). Then they say " this mild correlation was insufficient to explain the pronounced increase in suppression", but how do they know that it is insufficient?

We thank the reviewer for pointing this out. Our intention was not to argue that the relationship is statistically weak—indeed the correlation is highly significant—but rather that generalism alone does not explain the observed pattern. While generalists are enriched among highly influenced strains ($r = 0.36$, $p < 0.001$), the overlap between the two classifications is only partial. This is evident from the confusion matrix shown in Supplementary Fig. 22, as well as from the moderate agreement metrics (Cohen's $\kappa = 0.36$; Jaccard = 0.43). Specifically, only 67 strains are both generalists and highly influenced, whereas 89 strains (42 + 47) are exclusively generalist or exclusively highly influenced. This partial overlap indicates that generalism alone cannot explain the emergence of the highly influenced strain phenotype.

We have therefore revised the text to emphasize this incomplete correspondence rather than the strength of the correlation: *"However, the overlap between generalist strains and highly influenced strains is only partial. For example, only 67 strains are both generalists and highly influenced, whereas 89 strains fall exclusively into one category or the other (Supplementary Fig. 22). This incomplete correspondence indicates that generalism alone cannot explain the pronounced increase in suppression observed under higher resource diversity, suggesting that additional mechanisms must contribute to the stronger competitive responses of highly influenced strains."*

We agree that the community experiment highlights one strain (strain 79) with a particularly strong effect, and that this observation alone is anecdotal. However, the purpose of this example is simply to illustrate that the strain with the strongest impact factor does not correspond to exceptional generalism, which contradicts the mechanism proposed by Pacheco et al. The large effect of strain 79 partly reflects the size of the community experiment, which included only 14 strains and 14 assembled communities, with strain 79 present in 8 of them. Importantly, our conclusions do not rely on this single observation. We complemented the experimental results with LV simulations that reproduced the observed biodiversity patterns when strains with high impact factors were included.

Notably, our approach closely follows the framework introduced by Pacheco et al., where a limited experimental strain set (13 strains) was used to identify the phenomenon, and simulations were then employed to explore its consequences across broader community compositions and parameter regimes. In addition, their experimental set contained few non-generalist strains, with most strains behaving as generalists on the relevant resources (see Supplementary Fig. 9B in Pacheco et al.). In this sense, both studies begin from limited experimental observations and rely on simulations to assess the generality of the phenomenon.

Finally, we complemented these analyses with metabolite–supernatant experiments that were independent of the sentinel assay and the resource mixtures used there. These physiological experiments reproduced the distinctions between highly and mildly influenced strains, providing additional support that the observed phenomenon is not solely attributable to generalism.

Moreover, later on the authors themselves show that highly influenced strains uptake more resources as more resources become available. Isn't this exactly the definition of generalism?

We respectfully disagree that this observation corresponds to generalism. In microbial ecology, generalism refers to niche breadth, i.e., the number of distinct resources on which a microbe can grow. In contrast, the phenomenon we observe concerns the uptake intensity of resources that the organism already utilizes, not the expansion of the number of usable resources.

To illustrate this distinction: consider a strain that can grow on 9 out of 10 tested carbon sources (a generalist) and another that can grow on only 2 out of 10 (a specialist). Increasing resource diversity in the environment does not cause the specialist to gain the ability to utilize additional resources (e.g., from 2/10 to 3/10). Instead, what we observe is that strains increase the uptake of the same resources they were already able to consume, particularly for highly influenced strains.

We tested this explicitly in our dataset and found no evidence that highly influenced strains expand their resource niche breadth with increasing resource diversity. Rather, the data show that the same strains consume more of the same resources they could already utilize. This distinction explains why the phenomenon we observe cannot be explained by generalism alone. This analysis was previously presented in the last rebuttal and attached here as well (Rebuttal Fig. 1).

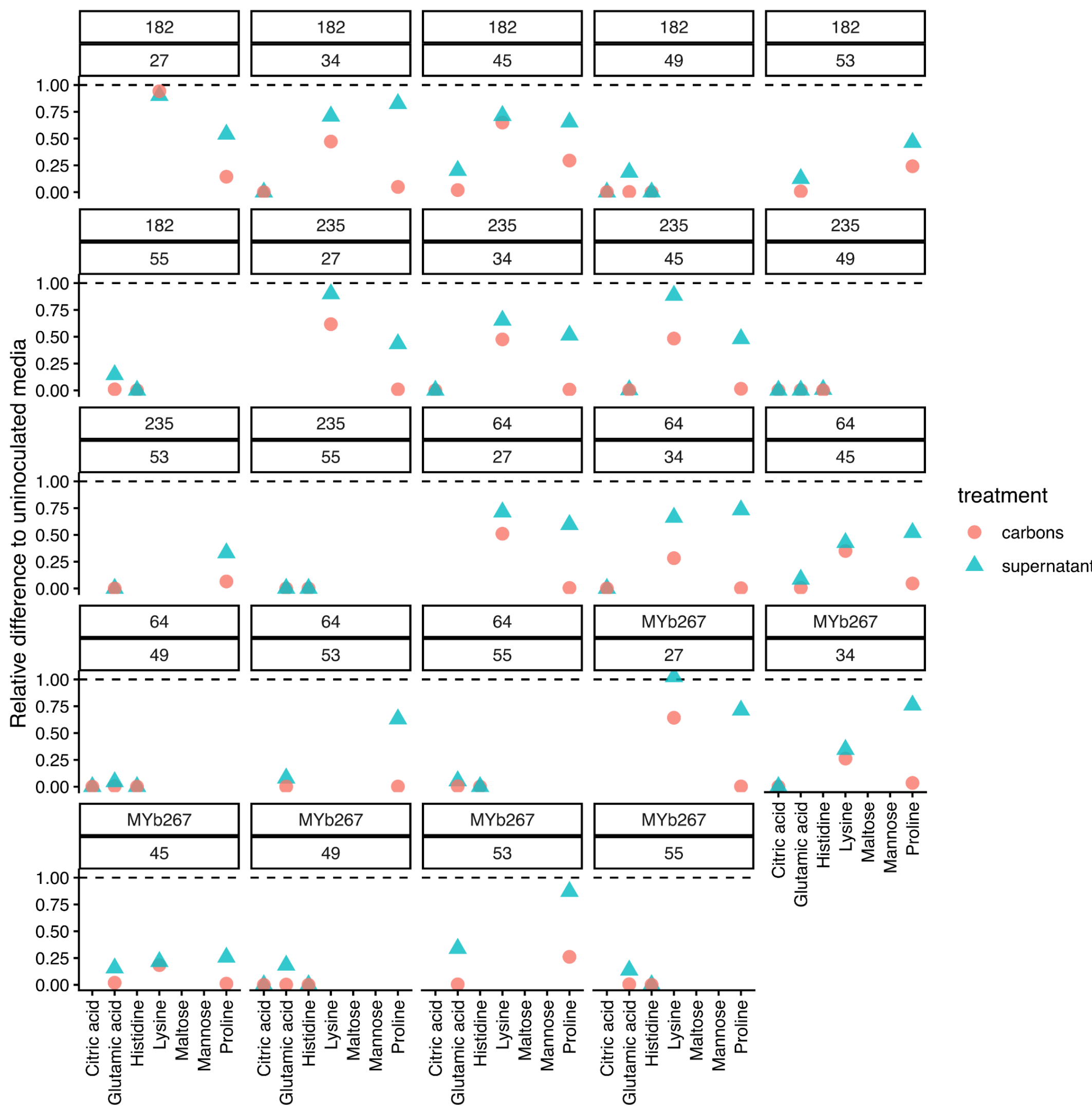

Rebuttal Fig. 1: Strains utilize existing niches more efficiently in mixed-carbon conditions but do not exploit new ones (no emergent resource generalism). Shown are raw GC–MS results, expressed as the relative difference in peak area (AUC) between post-growth samples and the corresponding supplied carbon peaks in uninoculated controls. Each panel shows results for an individual strain (upper facet) across different carbon mixtures (lower facet). Blue points represent supernatant mixtures of individual carbons, and red points represent direct carbon mixtures (design illustrated in Fig. 4A). The dashed line indicates a value of 1, corresponding to no difference between inoculated and uninoculated samples. Except for a single case, lysine uptake by strain MYb267 in mixture 27, mixed-carbon conditions never resulted in the appearance of new uptake peaks relative to single-carbon conditions. This exception is likely an artefact, as the same strain uptakes lysine in both supernatant and carbon

mixtures in mixture 34. Overall, our results support improved use of existing niches but no emergent ability to utilize new carbon sources.

3. The explorations of parameter space in the CR model in the last section of results identifies a rather narrow set of conditions in which biodiversity declines with resource availability. This is OK, and is actually a very valuable result, but it should be more transparently acknowledged.

We agree that this point should be stated more explicitly. While the text already describes the conditions that promote biodiversity decline, we now clarify in the Results that biodiversity loss is not the generic outcome of the model but emerges under specific ecological conditions, which we then identify through the parameter exploration (Supplementary Figs. 30–34): *“Biodiversity loss does not occur across all parameter combinations, but emerges under specific ecological conditions that we identify below.”*

4. Regarding Fig 4E, I am wondering how did the pattern become so qualitatively different from the same figure in the previous version of the manuscript.

The qualitative difference arises because we identified and corrected a numerical issue in the simulations used in the previous version. Specifically, the error tolerance of the ODE solver was not sufficiently strict, which occasionally led to unbounded growth of some species. In such cases, the fastest-growing strain could dominate by several orders of magnitude, artificially reducing Shannon diversity to near zero. This artifact occurred more frequently under conditions favorable to highly competitive strains and therefore produced an artificially sharp decline in biodiversity at high HIS fractions in the previous version of Fig. 4E. In the revised manuscript we corrected this issue by tightening the solver tolerance (atol reduced from 1e-3 to 1e-6) and rerunning all simulations. We also standardized the simulated community sets across conditions to reduce baseline variance. The corrected simulations produce the revised Fig. 4E and associated supplementary analyses. We also noted this correction in our previous rebuttal letter when submitting the revised version of the manuscript.

Regarding the framing and discussion of the background, I still disagree on some points:

1. Authors claim they are testing whether resource diversity - species diversity theory holds, which is an ambitious claim. The theory has been tested already (as mentioned, dal bello et al, and Pacheco et al). Regarding dal Bello, I do not fully agree that top down community assembly reflects only environmental filtering. While the loss of many species does happen because of environmental filtering, the remaining community does reflect interactions among the species (which of course might be different in a synthetic bottom up assembly scenario). I don't agree they are masked, they can be very strong indeed (see e.g. Goldford et al 2018).

We agree that interactions can remain strong in top-down community assembly experiments such as Dal Bello et al., and that environmental filtering does not eliminate them. Our point was not that interactions are absent in these systems, but that strong initial environmental filtering can make it difficult to disentangle how interactions themselves change as resource diversity increases. In the Dal Bello et al. dataset, the initial soil communities contained thousands of taxa (~3,500 ASVs), yet after laboratory transfer only a small subset reached appreciable abundance (on average ~20 ASVs above 0.1% relative abundance; we confirmed this by re-analyzing their published data and include the corresponding plot as an attachment to this rebuttal).

Importantly, the theoretical framework used by Dal Bello et al. does not fully disentangle the relative contributions of environmental filtering and species interactions. Their consumer–resource model incorporates cross-feeding—a genuine biotic interaction in which metabolites produced by one taxon serve as resources for another—but it cannot separate the diversity contribution of these interactions from the diversity contribution of the directly supplied nutrients. As more carbon sources are added experimentally, both effects increase simultaneously: the supplied resource pool expands, and the cross-feeding network seeded by those resources also grows. The model recapitulates the observed linear diversity increase but cannot attribute it specifically to interaction-mediated coexistence versus direct resource partitioning. Moreover, the specific mechanisms proposed by the model are not directly tested experimentally, making it difficult to determine which processes drive the empirical observations.

Similar concerns have also been raised in the discussion of the Goldford et al. study (see the eLetter comment below their paper).

In contrast, our bottom-up synthetic community approach allows us to directly track how interaction strengths and competitive outcomes shift as resource diversity changes. We have clarified this point in the revised Introduction and replaced our original wording with the following sentence: “Such strong environmental filtering can make it difficult to disentangle how species interactions change with increasing resource diversity.”

Reanalysis of Dal Bello et. al final relative ASVs relative abundances:

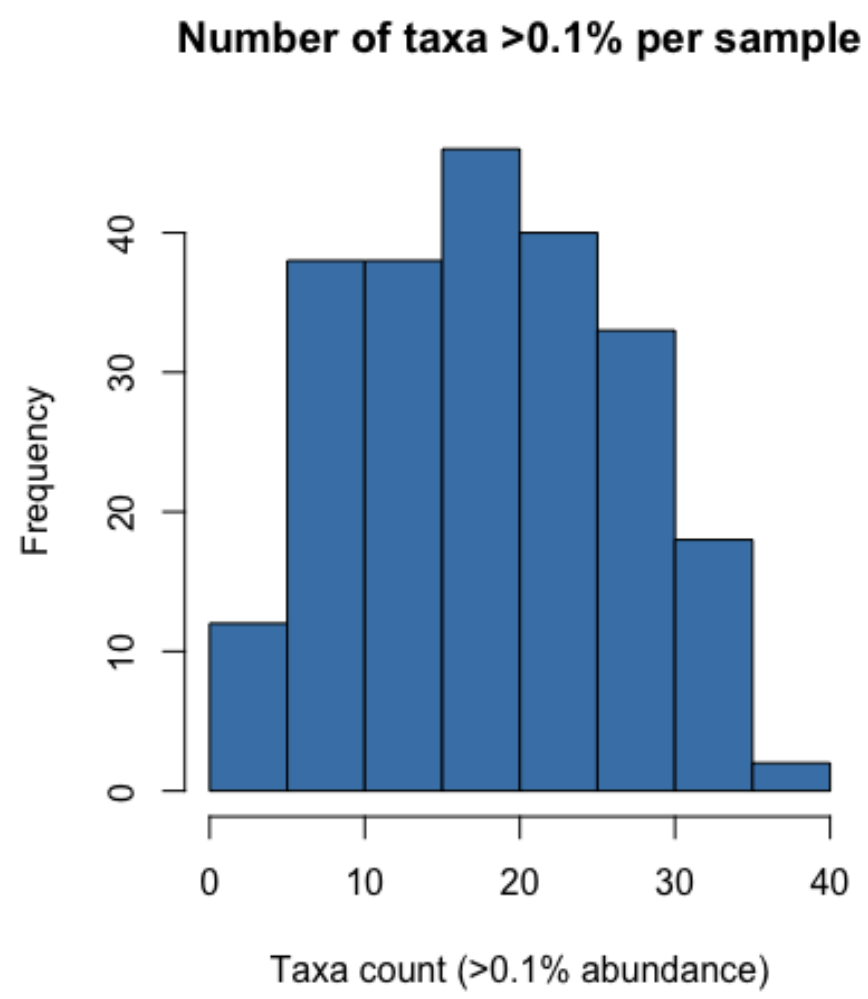

2. The natural relevance of this synthetic community scenario could be also questioned. How many natural communities are assembled in this way? Also, despite the initial claim that the chosen species are phylogenetically diverse, there are still substantial biases which can influence the results, and I could imagine that easily with another set of species one could find a different result.

We agree that synthetic communities are simplified representations of natural ecosystems and cannot capture their full complexity. However, reductionist laboratory systems are widely used in microbial ecology precisely because they allow controlled testing of ecological mechanisms that are difficult to isolate in natural communities. Previous studies addressing the relationship between resource diversity and microbial biodiversity have relied on similar approaches, including both defined communities (e.g., Pacheco et al.) and top-down assemblies from natural communities (e.g., Dal Bello et al.).

In this context, our study substantially expands the experimental scope of defined community systems. While Pacheco et al. examined 13 strains and a single community configuration, our experiments involved 298 strains in the sentinel assays and multiple assembled communities in the community experiments. These strains were collected from diverse environments, by two different reserach groups and from two continents, and span a wide range of phylogenetic groups (Supplementary Fig. 19), allowing us to examine whether the observed competitive response is broadly distributed across taxa.

We agree that any specific strain collection may influence quantitative outcomes, and that different strain sets could in principle produce different results. However, this limitation applies to all controlled microbial community experiments. Our goal was therefore not to claim universal generality, but to identify and mechanistically explain a reproducible ecological pattern across a large and phylogenetically diverse strain collection. To clarify this point, we have added the following sentence to the Discussion: *“While it is inherently difficult to generalize from laboratory experiments to*

all natural microbial communities, our results emerge from a large and phylogenetically diverse collection of isolates, suggesting that the mechanism we identify is not restricted to a particular species combination."

Note that I might sound harsh, but I am not saying this work is without value. In fact I appreciate, and actually like this type of experiments and approach, I do think the amount of work is impressive and the results are valuable. I am only still a bit bothered by overstatement.

We appreciate the reviewer's thoughtful feedback and positive assessment of the work. We agree that careful framing is important, and throughout the revision we have worked to moderate several statements to avoid overgeneralization and better clarify the scope and interpretation of our results.

Some additional minor points:

L33: "has been remaining" sounds unnatural in english
Thanks. Changed to "remained"
L61 - question mark is unnecessary
Removed.
Supplement, caption new Fig. S6 should be S7
Thanks - changed.
I think Fig. 2E is not a reanalysis of Fig. 1C as stated, but 1E?
True! Thanks.

Reviewer #3 (Remarks to the Author):

In this revised manuscript, Shalev et al. address the major concerns raised by three reviewers in the original submission. Overall, the manuscript presents an impressive experimental test of the hypothesis that biodiversity increases with resource diversity. Using their synthetic communities and an analysis of the Earth Microbiome Project data, the authors find mixed support for this hypothesis. In the first round of review, it is interesting that all reviewers had very similar concerns about the original manuscript. Among these concerns are the strength of interpretations and the role of generalists. It is striking how similar the reviewers' comments are. In the revised manuscript, the authors provide a detailed response, major revisions to the original text, and a very lengthy supplement (56 pages and 35 supplemental figures). Overall, the authors have incorporated substantial feedback but ultimately want to maintain their original conclusions.

After reviewing the revised manuscript and the complete response to reviewers, I am pleased to see how much the authors have incorporated feedback. While I still do not fully agree with their conclusions, they have provided detailed justifications for each point. My only major concern now is the strength of some of their statements. I firmly believe they are overinterpreting their results and making them too generalizable. I think their results apply to their simplified system, but I am still not convinced they are applicable to microbial communities writ large. As such, I would ask the authors to provide another revision and tone down some of the comments:

L 27: "Overturn classical ecological principles" – that is way way too strong.

Agreed. Toned down to the whole sentence to *"Our findings suggest that physiological changes at the individual level can substantially alter predicted diversity patterns and scale up to influence community structure."*

L93: "Often" – I believe your results indicate that resource diversity "can" reduce biodiversity. For example, you only observed this in 7 of 14 communities.

Changed to "can".

L218: It seems that most of your conclusions are the result of a single strain (79). I wouldn't say that is generalizable at all.

We agree that strain 79 shows the strongest effect in the community experiments. This partly reflects the limited size of the community experiment, which included 14 strains and 14 randomly assembled communities, with strain 79 present in 8 of them. This limitation is also acknowledged in the manuscript when discussing the simulations, which were introduced specifically to generalize beyond the limited statistical power of the experimental dataset ("This approach allowed us to generalize beyond the limited statistical power of the experimental dataset."). However, our conclusions do not rely solely on this strain.

First, the community experiment was designed primarily to illustrate how strong pairwise competitive effects can scale up to community-level biodiversity loss.

Second, to generalize beyond this specific example, we used LV simulations parameterized based on the experimentally measured interaction patterns. This approach follows a similar logic to that used by Pacheco et al., where limited experimental observations were complemented with simulations to explore the consequences across broader community compositions.

Third, the broader pattern of increased competitiveness with resource diversity is supported by the sentinel strain experiments across 298 strains, which show that stronger suppressive responses under higher resource diversity are common across our strain collection.

We therefore use strain 79 as a representative example to illustrate the mechanism, rather than as the sole basis for the conclusions.

Also, I think you need to provide more details about how you are calculating niche breadth

We thank the reviewer for this suggestion. To make this clearer for readers, we now clarify in the main text how niche breadth was calculated when introducing the analysis of generalism: "We defined niche breadth as the proportion of tested carbon resources on which a strain showed measurable growth (OD_{600} > 0.15 after 72 h)."

This definition was already provided in the captions of the relevant supplementary figures. For example, the caption of Supplementary Fig. 15 states: "Niche breadth was calculated as the proportion of resources on which each strain showed growth above an OD_{600} of 0.15—a conservative threshold exceeding the maximum OD observed in any control well." Similarly, Supplementary Fig. 22 notes: "The full collection of 298 strains used in this study was grown across the 22 carbon resources included throughout the work. OD_{600} was measured after 72 h (carrying capacity). Results are shown as a heatmap, with strains classified as generalists (niche breadth > 0.5) or specialists (niche breadth ≤ 0.5). Niche breadth was calculated as the number of resources supporting growth above control level (OD_{600} > 0.15; this conservative threshold exceeds the maximum OD_{600} observed in any control well). PAO1 niche overlap was calculated using the Jaccard distance."

L268: Increased competitiveness with resource diversity is "Widespread". As you state in your response to reviewers, this evidence is mixed and a bit complicated to interpret.

We changed the title to be more cautious about generalization: *"Increased microbial competitiveness with resource diversity is widespread across our strain collection."*

L297: Why is $r = 0.36$ $p < 0.001$ only moderate? You interpret other correlations at this level as significant.
L300: Why is this mild correlation insufficient? This is not justified.

We thank the reviewer for pointing this out. Our intention was not to argue that the relationship is statistically weak—indeed the correlation is highly significant—but rather that generalism alone does not explain the observed pattern. While generalists are enriched among highly influenced strains ($r = 0.36$, $p < 0.001$), the overlap between the two classifications is only partial. This is evident from the confusion matrix shown in Supplementary Fig. 22, as well

as from the moderate agreement metrics (Cohen’s κ = 0.36; Jaccard = 0.43). Specifically, only 67 strains are both generalists and highly influenced, whereas 89 strains (42 + 47) are exclusively generalist or exclusively highly influenced. This partial overlap indicates that generalism alone cannot explain the emergence of the highly influenced strain phenotype.

We have therefore revised the text to emphasize this incomplete correspondence rather than the strength of the correlation: *“However, the overlap between generalist strains and highly influenced strains is only partial. For example, only 67 strains are both generalists and highly influenced, whereas 89 strains fall exclusively into one category or the other (Supplementary Fig. 22). This incomplete correspondence indicates that generalism alone cannot explain the pronounced increase in suppression observed under higher resource diversity, suggesting that additional mechanisms must contribute to the stronger competitive responses of highly influenced strains.”*

L467: You need to modify “often reduces biodiversity”. This is not a justified strong conclusion based on your results. I suggest you tone down this type of language here and elsewhere.

Changed to “can”.

********************END********************

Reviewer #1:
Remarks to the Author:
The authors have addressed all my remaining concerns.

Thank you.

Reviewer #2:
Remarks to the Author:
I thank the authors for addressing my concerns in a largely satisfactory manner. In particular, I think that the discussion about difference between generalist and highly-influenced strains is now much clearer, as it seems, in a previous version I did not understand what the authors meant. While I still disagree with some parts (e.g. the discussion on environmental filtering and interactions), this is a matter of discussion. Overall I do find now that broadly speaking the results are more transparent, and the tone and ambition of the claims are much better adjusted to the results.

Minor - Please re-read and fix several typos e.g. L514 "our results has important implications"
Thank you for your attention. We scanned the manuscript thoroughly and fixed all typos, including L514.
